# Toward a pan-SARS-CoV-2 vaccine targeting conserved epitopes on spike and non-spike proteins for potent, broad and durable immune responses

**DOI:** 10.1371/journal.ppat.1010870

**Published:** 2023-04-20

**Authors:** Chang Yi Wang, Wen-Jiun Peng, Be-Sheng Kuo, Yu-Hsin Ho, Min-Sheng Wang, Ya-Ting Yang, Po-Yen Chang, Yea-Huei Shen, Kao-Pin Hwang

**Affiliations:** 1 UBI Asia, Hsinchu, Taiwan; 2 StatPlus, Inc., Taipei, Taiwan; 3 Division of Infectious Diseases, China Medical University Children’s Hospital, China Medical University, Taichung, Taiwan; 4 School of Medicine, College of Medicine, China Medical University, Taichung, Taiwan; Icahn School of Medicine at Mount Sinai, UNITED STATES

## Abstract

**Background:**

The SARS-CoV-2 non-Spike (S) structural protein targets on nucleocapsid (N), membrane (M) and envelope (E), critical in the host cell interferon response and memory T-cell immunity, are grossly overlooked in COVID vaccine development. The current Spike-only vaccines bear an intrinsic shortfall for promotion of a fuller T cell immunity. Vaccines designed to target conserved epitopes could elicit strong cellular immune responses that would synergize with B cell responses and lead to long-term vaccine success. We pursue a universal (pan-SARS-CoV-2) vaccine against Delta, Omicrons and ever-emergent new mutants.

**Methods and findings:**

We explored booster immunogenicity of UB-612, a multitope-vaccine that contains S1-RBD-sFc protein and sequence-conserved promiscuous Th and CTL epitope peptides on the Sarbecovirus N, M and S2 proteins. To a subpopulation (N = 1,478) of infection-free participants (aged 18–85 years) involved in a two-dose Phase-2 trial, a UB-612 booster (third dose) was administered 6–8 months after the second dose. The immunogenicity was evaluated at 14 days post-booster with overall safety monitored until the end of study. The booster induced high viral-neutralizing antibodies against live Wuhan WT (VNT_50_, 1,711) and Delta (VNT_50_, 1,282); and against pseudovirus WT (pVNT_50,_ 11,167) vs. Omicron BA.1/BA.2/BA.5 variants (pVNT_50_, 2,314/1,890/854), respectively. The lower primary neutralizing antibodies in the elderly were uplifted upon boosting to approximately the same high level in young adults. UB-612 also induced potent, durable Th1-oriented (IFN-γ^+^-) responses (peak/pre-boost/post-boost SFU/10^6^ PBMCs, 374/261/444) along with robust presence of cytotoxic CD8^+^ T cells (peak/pre-boost/post-boost CD107a^+^-Granzyme B^+^, 3.6%/1.8%/1.8%). This UB-612 booster vaccination is safe and well tolerated without SAEs.

**Conclusions:**

By targeting conserved epitopes on viral S2, M and N proteins, UB-612 could provide potent, broad and long-lasting B-cell and T-cell memory immunity and offers the potential as a universal vaccine to fend off Omicrons and new VoCs without resorting to Omicron-specific immunogens.

**Trial registration:**

ClinicalTrials.gov ID: NCT04773067; ClinicalTrials.gov ID: NCT05293665; ClinicalTrials.gov ID: NCT05541861.

## Author summary

The Omicron variant of SARS-CoV-2 has swept the globe with a rapid succession of dominating sublineages from BA.1, BA.2, to the current BA.5 with increasing infectivity and antibody evasion. Concerningly, the non-Spike structure proteins that promote T-cell immunity have been grossly overlooked in vaccine development. Looking beyond short-interval boosters and Omicron-updated vaccines, a pragmatic approach to curbing ever-emergent new mutants would be “universal (pan-SARS-CoV-2) vaccines” as exemplified by UB-612, a multitope-vaccine armed with Spike (S1-RBD and S2) and non-Spike (Nucleocapsid N and Membrane M) targets, allowing booster vaccination to elicit broadly-recognizing and durable B-/T-cell memory immunity. Sequence-conserved Th/CTL epitope peptides were designed from S2, N and M proteins to synergistically enhance memory helper and cytotoxic T-cell immunity and RBD targeted B-cell immunity.

## Introduction

The SARS-CoV-2 Omicron lineage has swept the globe with a rapid succession of dominating subvariants from BA.1, BA.2 and to the current BA.5 that makes up more than 90% of infection cases with overriding edges in transmissibility and neutralizing antibody escape [1–7]. Relative to Delta variant, Omicron BA.1 does not require fusion co-receptor TMPRSS2 for cell entry; instead, it enters the cells through endosomes [8,9]. It infects upper bronchial cells and proliferates much more efficiently; it does not foster cell syncytia nor erode lung-alveolar tissues [8–14], thus causing lesser disease severity.

Omicron BA.1 is heavily mutated (Table 1), including more than 35 changes in S protein [8,15–17]. Compared to 2 mutations associated with Delta at S1 receptor binding domain (S1-RBD, residues 319–541), BA.1 and BA.2 share 12 mutations, with BA.1 and BA.2 each having additional 3 and 4 unique ones, respectively, that confers BA.2 a higher immune evasion. BA.4 and BA.5 have identical Spike protein. They differ from BA.2 by having additional mutations at 69-70del, L452R, F486V and wild type amino acid at position Q493 [18] (Table 1) within the Spike protein, contributing to their higher degree of immune escape than BA.2.

**Table 1:** The mutation sites on SARS-CoV-2 Spike (S), Envelope (E), Membrane (M), and Nucleocapsid (N) proteins on Delta and Omicrons^a^.

VoC^b^	Spike (S1-RBD residues at 319–541)	E	M	N
**Delta**	T19R, G142D, Δ156–157, R158G, $\underline{L452R}$, T478K, D614G, P681R, & D950N	T9I	I82T	D63G, R203M, & D377Y
**Omicron** **(BA.1)**	A67V, Δ69–70, T95I, G142D, Δ143, Y144del, Δ145, Δ211, L212I, +214EPE, G339D, R346K, S371L, S373P, S375F, K417N, N440K, G446S, S477N, T478K, E484A, Q493R, G496S, Q498R, N501Y, Y505H, T547K, D614G, H655Y, N679K, N764K, D796Y, N856K, and Q954H, L969K, & $\underline{L981F}$	T9I	D3G, Q19E, & A63T	P13L, Δ31–33, R203K, & G204R
**Omicron** **(BA.2)**	T19I, L24S, Δ25–27, G142D, V213G, G339D, S371F, S373P, S375F, T,376A, D405N, R408S, K417N, N440K, S477N, T478K, E484A, $\underline{Q493R}$, Q498R, N501Y, Y505H, D614G, H655Y, N679K, N764K, D796Y, N856K, Q954H, & L969K	T9I	Q19E & A63T	P13L, Δ31–33, R203K, G204R, & S413R
**Omicron** **(BA.4)**	T19I, L24S, Δ25–27, Δ69–70, G142D, V213G, G339D, S371F, S373P, S375F, T,376A, D405N, R408S, K417N, N440K, L452R, S477N, T478K, E484A, L486V, Q493, Q498R, N501Y, Y505H, D614G, H655Y, N679K, N764K, D796Y, N856K, and Q954H, & L969K	T9I	Q19E & A63T	P13L, Δ31–33, P151S, R203K, G204R, & S413R
**Omicron** **(BA.5)**	T19I, L24S, Δ25–27, Δ69–70, G142D, V213G, G339D, S371F, S373P, S375F, T,376A, D405N, R408S, K417N, N440K, L452R, S477N, T478K, E484A, L486V, Q493, Q498R, N501Y, Y505H, D614G, H655Y, N679K, N764K, D796Y, N856K, and Q954H, & L969K	T9I	D3N, Q19E, & A63T	P13L, Δ31–33, R203K, G204R, & S413R

^a^ Reported mutation sites on Spike, E, M, and N proteins [Refs: 15–18 and 44–47].

^b^ Omicron BA.4 and BA.5 have identical mutation site profile on Spike protein, which are more related to BA.2 than BA.1. Among BA.2, BA.4 and BA.5, the between-variant differences in mutation sites on S, E, M, and N proteins are marked in red.

^c^ Except for N969K (on BA.1 through BA.5) and L981F (on BA.1) within S_957-984_ peptide on the S2 spike protein, none of the other four designer epitope peptides (Table 2) for UB-612 vaccine has an aa-residue that overlaps with the reported mutation sites on Spike, M, and N proteins.

BA.2 exhibits a 1.3- to 1.5-fold higher transmissibility and a 1.3-fold immune evasion than BA.1 [16,19], consistent with the finding that BA.1-immune sera neutralizes BA.2 with lower titers by a factor of 1.3 to 1.4 [20] and that BA.2 reinfection can occur after BA.1 [21]. BA.4/BA.5 are more transmissible and resistant to BA.1/BA.2-immunity and monoclonal antibodies [1,2]. While BA.2 vs. BA.1 [19] and BA.4/BA.5 vs. BA.2 [22] display greater cell-to-cell fusion, they do not make infected people sicker nor change the fundamental pandemic dynamics.

Amongst double-vaccinated adults, the booster (third dose)-induced neutralization titers against BA.4/BA.5 are notably lower than those against BA.1/BA.2 [3–5]; and, amongst BA.1-infected adults, the immune sera can potently neutralize BA.1/BA.2, but showing weaker responses against BA.4/BA.5 [6,22,23]. These suggest that booster vaccination or BA.1/BA.2 infection may not achieve sufficient immunity to protect against BA.4/BA.5 while reinfection would not be uncommon.

Regardless of vaccination status or hybrid immunity, each reinfection would add risks of mortality, hospitalization and other health hazards including burden of long COVID [24]. The long COVID associated with Omicron [25–28] and pre-Omicron VoCs [26] has loomed large from infections that didn’t require hospitalization [28]. Immunization with current EUA approved vaccines could present only limited benefits to relieve long COVID [29,30].

While a booster 3^rd^-dose of mRNA vaccines could compensate Omicron (BA.1)-induced decrease in serum neutralizing antibodies (20- to 30-fold reduction), along with rates of hospitalization and severe disease (80–90% protection) [31–36], they offer less effective protection against mild and asymptomatic infections (40–50% protection) [36]. Breakthrough infections identified with high viral loads are common even after the fourth jab (2^nd^ booster to adults aged 18 and older) [37].

While development of composition-updated (variant-specific) vaccines has been strongly advocated [38,39], a better strategy of “universal coronavirus vaccines” would be more urgently needed [40] for robust, broad, and durable immunity. The currently authorized Spike-only vaccines do not incorporate SARS-CoV-2’s non-Spike structure proteins of envelope (E), membrane (M) and nucleocapsid (N), the regions critically involved in the host cell interferon response and T-cell memory [41–43]. Oversight of non-Spike proteins as targets could lead to an intrinsic shortfall for promotion of a fuller T cell immunity. Viral mutations are also known to occur in E, M and N (Table 1) [15–18,44–47], the structure proteins that are beyond recognition by current EUA-approved vaccines.

In the present Phase-2 extension study, we affirm that a booster vaccination (third dose) by UB-612, a multitope-vaccine which contains S1-RBD-sFc fusion protein enriched with five rationally-designed promiscuous peptides representing sequence-conserved Th and CTL epitopes on the Sarbecovirus N, M and S2 proteins across all VoCs (Table 2); and a sixth idealized universal Th peptide which serves as a catalyst in T cell activation [48] can induce potent, broadly-recognizing, durable antibodies and T-cell immunity that offers potential as a universal vaccine to fend off Omicrons and new mutants.

**Table 2:** Rationally-designed sequence-conserved Th/CTL epitope peptides on M, N, and S2 proteins across all SARS-CoV-2 Variants of Concern (VoCs)^a^.

Wild type & VoCs	M protein SARS-CoV-2 M_101-156_ (CTL epitopes)	N protein SARS-CoV-2 N_305-331_ (Th/CTL epitopes)	S2 protein^b^^,^^c^ SARS-CoV-2 S_957-984_ (Th/CTL epitopes)	S2 Protein SARS-CoV-2 S_891-917_ (Th epitope)	S2 Protein SARS-CoV-2 S_996-1028_ (Th/CTL epitope)
**Wuhan** **(Original)**	GLMWLSYFIASFRLFARTRSMWS	AQFAPSASAFFGMSRIGMEVTPSGTWL	QALNTLVKQLSSNFGAISSVLNDILSRL	GAALQIPFAMQMAYRFNGIGVTQNVLY	LITGRLQSLQTVVTQLIRAAEIRASANLAATK
**Alpha, Beta, & Gamma**	GLMWLSYFIASFRLFARTRSMWS	AQFAPSASAFFGMSRIGMEVTPSGTWL	QALNTLVKQLSSNFGAISSVLNDILSRL	GAALQIPFAMQMAYRFNGIGVTQNVLY	LITGRLQSLQTVVTQLIRAAEIRASANLAATK
**Delta**	GLMWLSYFIASFRLFARTRSMWS	AQFAPSASAFFGMSRIGMEVTPSGTWL	QALNTLVKQLSSNFGAISSVLNDILSRL	GAALQIPFAMQMAYRFNGIGVTQNVLY	LITGRLQSLQTVVTQLIRAAEIRASANLAATK
**Omicron** ^**c**^ **(BA.1)**	GLMWLSYFIASFRLFARTRSMWS	AQFAPSASAFFGMSRIGMEVTPSGTWL	QALNTLVKQLSS**K**FGAISSVLNDI**F**SRL	GAALQIPFAMQMAYRFN GIGVTQNVLY	LITGRLQSLQTVVTQLIRAAEIRASANLAATK
**Omicron** ^**c**^ **(BA.2/BA.4/ BA.5)**	GLMWLSYFIASFRLFARTRSMWS	AQFAPSASAFFGMSRIGMEVTPSGTWL	QALNTLVKQLSS**K**FGAISSVLNDILSRL	GAALQIPFAMQMAYRFN GIGVTQNVLY	LITGRLQSLQTVVTQLIRAAEIRASANLAATK

^a^ The presence of T cell epitopes is critical for the induction of B and T cell memory responses against viral antigens. SARS-CoV-2 CTL and Th epitopes, validated by HLA binding and T cell functional assays, are highly conserved between SARS-CoV-2 and SARS-CoV-1 viruses, with minor between-variant differences seen only at S_957-984_. The Wuhan wild-type peptides (M, N and S2x3) are employed for precision-design of UB-612 vaccine against COVID-19 [Ref. 90]. Identification of T cell epitopes on SARS-CoV-1 (2003), determined using HLA-binding assays, were used to determine corresponding T cell epitopes in SARS-CoV-2 (2019) by sequence alignment.

^b^ Except for N969K (on BA.1 through BA.5) and L981F (on BA.1) within S_957-984_ peptide on the S2 spike protein, none of the other four designer epitope peptides for UB-612 vaccine has an aa-residue that overlaps with the reported mutation sites on Spike, M, and N proteins (Table 1).

^c^ At S957-984, there are minor sequence differences between Omicron BA.1 and BA.2/BA.4/BA.5, marked in red.

## Methods

### Ethics statement

The study was conducted according to Study Protocol V-205 approved by Institutional Review Board (IRB) at: China Medical University Hospital, Taipei Medical University, Far Eastern Memorial Hospital, National Cheng Kung University Hospital, Chang Gung Medical Foundation, Kaohsiung Medical University Chung-Ho Memorial Hospital, Tri-Service General Hospital, Taipei Veterans General Hospital, Kaohsiung Veterans General Hospital, Changhua Christian Hospital, and Taichung Veterans General Hospital. Written Informed Consent was obtained from all the study participants. The Study Protocol, IRB approval letters, and Informed Consent Form are provided in **Supporting Information** (S1–S3 Appendices).

### Design of Phase-2 extension booster trial and oversight

#### Booster 3^rd^-dose following the Phase-2 trial primary 2-dose series

We conducted a booster vaccination study (n = 1,478) which was an extension arm of the Phase-2, placebo-controlled, randomized, observer-blind, multi-center primary 2-dose study (S1A Fig) [ClinicalTrials.gov ID: NCT04773067] in Taiwan with 3,844 healthy male or female adults aged >18 to 85 years (S1B Fig) who received two intramuscular doses (28 days apart) of 100 μg UB-612 or saline placebo. The objectives of the third-dose extension study were to determine the booster-induced safety and immunogenicity after unblinding, 6 to 8 months after the second dose.

The Principal Investigators at the study sites agreed to conduct the study according to the specifics of the study protocol and the principles of Good Clinical Practice (GCP); and all the investigators assured accuracy and completeness of the data and analyses presented. The protocol was approved by the ethics committee at the sites and all participants provided written informed consent. Full details of the booster trial design, inclusion and exclusion criteria, conduct, oversight, and statistical analysis plan are available in the study protocol.

### Trial procedures of safety and immunogenicity

#### Reactogenicity in the primary and booster series

The primary safety endpoints of the Phase-2 primary series (Days 1–365) and extension booster trial (recorded until 14 days post-booster and followed up study end) were to evaluate safety and tolerability. Vital signs were assessed before and after each injection. After each injection, participants had to record solicited local and systemic AEs in their self-evaluation e-diary for up to seven days while skin allergic reactions were recorded in their e-diary for up to fourteen days. Safety endpoints include unsolicited AEs reported for Days 1 to 57 in primary series and Days 1 to 14 in the booster phase. The overall safety was followed until the end of this study. Complete details for solicited reactions are provided in the study protocol.

#### Scope of immunogenicity investigation

The primary immunogenicity endpoints were the geometric mean titers (GMT) of neutralizing antibodies against SARS-CoV-2 wild-type (WT, Wuhan strain), Delta, Omicron BA.1, BA.2 and BA.5 variants were explored. For WT and Delta strains, viral-neutralizing antibody titers that neutralize 50% (VNT50) of live SARS-CoV-2 WT and Delta variant were measured by a cytopathic effect (CPE)-based assay using Vero-E6 (ATCC CRL-1586) cells challenged with SARS-CoV-2-Taiwan-CDC#4 (Wuhan strain) and SARS-CoV-2-Taiwan-CDC#1144 (B.1.617.2; Delta variant). The replicating virus neutralization test conducted at Academia Sinica was fully validated using internal reference controls and results expressed as VNT_50_. For WT, Omicron BA.1, BA.2, and BA.4/BA.5 strains, 50% of pseudovirus neutralization titers (pVNT_50_) were measured by neutralization assay using HEK-293T-ACE2 cells challenged with SARS-CoV-2 pseudovirus variants expressing the Spike protein of WT, BA.1, BA.2, or BA.4/BA.5 variants.

The secondary immunogenicity endpoints include anti-S1-RBD IgG antibody, inhibitory titers against ACE2:RBDWT interaction, and T-cell responses assayed by ELISpot and Intracellular Staining. The RBD IgG ELISA was fully validated using internal reference controls and results expressed in end-point titers. A panel of 20 human convalescent serum samples from hospitalized Taiwan COVID-19 patients aged 20 to 55 years were also tested for comparison with those in the vaccinees. Human peripheral blood mononuclear cells (PBMCs) were used for monitoring T cell responses (ELISpot and ICS). The constructs of the UB-612 vaccine product, all bioassay methods and statistics are detailed in the **Supporting Information** (S1–S8 Methods).

## Results

### Booster trial population

After unblinding of Phase-2 trial, 1,478 of the 3,844 healthy study participants who completed the 2-dose primary vaccine series (28 days apart) of 100-μg UB-612 (S1A Fig) were enrolled to receive an additional 100-μg booster 3^rd^-dose at 6 to 8 months after the second shot. The booster vaccinees were followed for 14 days to evaluate safety and immunogenicity. The vast majority of participants were of Taiwanese origin, with two groups aged 18–65 years (76%) and 65–85 years (24%) (S1B Fig).

### Reactogenicity and safety

No vaccine-related serious adverse events (SAEs) were recorded; the most common solicited AEs were injection site pain and fatigue, mostly mild and transient (S2 Fig). The incidence of solicited local AEs slightly increased at post-booster (S2A Fig), mostly pain at the injection site (mild, 54%; moderate, 7%). The incidence of skin allergic reaction at post-booster was similar to post-dose 2 reported earlier [48] (S2B Fig). Fatigue/tiredness, muscle pain, and headaches that belonged to solicited systemic AEs were mostly mild (S2C Fig). Overall, no safety concerns were identified with UB-612 booster across age groups.

### Durable Th1 cell responses by ELISpot

**Fig 1:**
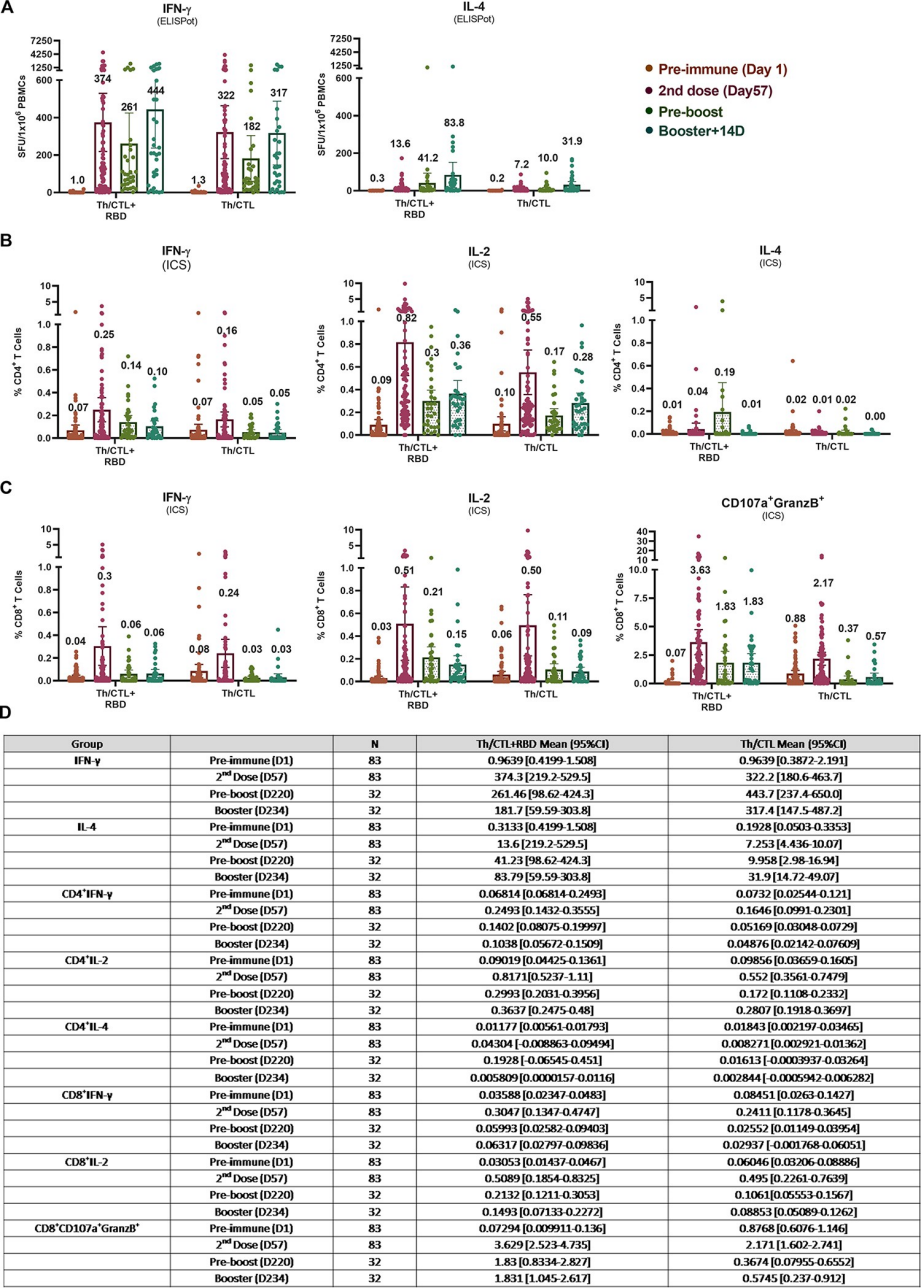
UB-612 induced T cell responses measured by ELISpot and ICS analyses. T-cell responses to stimulation by epitope peptides (RBD + Th/CTL or Th/CTL alone) were analysed with PBMCs collected from 83 vaccinees from Immunogenicity group (n = 83) on Day 57 (28 days after 2^nd^ dose); and from 32 vaccinees from the Immunogenicity (n = 18) or Safety groups (n = 14) who joined the Phase-2 extension booster study to evaluate the T-cell responses in PBMCs on Days 197 to 242 (pre-boosting days) and Days 211 to 256 (14 days post-booster third dose). T-cell responses were measured by (A) ELISpot at 10-μg/mL per stimulator, in which the Spot-forming units (SFU) per 1×10^6^ PBMCs producing IFN-γ, IL-2 and IL-4 after stimulation with the RBD + Th/CTL peptide pool or the Th/CTL peptide pool are expressed. The PBMC samples stimulated with Th/CTL+RBD were also evaluated for T cell responses by (B) Intracellular Staining (ICS), by which the %CD4^+^ T cells producing IFN-γ, IL-2 and IL-4; and (C) %CD8^+^ T cells producing IFN-γ, IL-2 and CD107a^+^Granzyme B^+^ in response to the stimulation by RBD+Th/CTL peptide pool or the Th/CTL peptide pool are shown. (D) Summary of mean and 95% CI are presented for plots as shown in panels (A) to (C). Horizontal bars indicate mean with 95% CI.

Vaccinees’ peripheral blood mononuclear cells (PBMCs) were collected for evaluation of Interferon-γ^+^ (IFN-γ^+^)-ELISpot. On Day 57 (28 days post-2^nd^ dose), IFN-γ SFU (Spot Forming Unit)/10^6^ cells under stimulation with RBD+Th/CTL peptide pool (Fig 1A) increased from the baseline 1.0 to a high peak at 374 SFU/10^6^ cells [48], which maintained robust at 261 (70%) at pre-boosting (6–8 months post-2^nd^ dose) and rose to 444 SFU 14 days post-booster. Together with the insignificant low levels of the IL-4^+^ ELISpot responses in the primary 2-dose series observed earlier [48] and post-booster in the present report, UB-612 vaccination as both primary series and homologous-boosting could induce pronounced Th1-predominant immunity.

Similar IFN-γ profiles were observed for those stimulated with Th/CTL peptide pool alone (Fig 1A), which increased from the baseline 1.3 to a high peak at 322 SFU/10^6^ cells on Day 57 [48], maintained at 182 SFU/10^6^ cells (~57%) at pre-boosting and remained strong at 317 SFU/10^6^ cells 14 days post-booster. T cell responses persisted robustly (60–70% of the high peak at Day 57) long over 6–8 months.

These results indicate that UB-612 can induce a strong and durable IFN-γ^+^ T cell immunity in the primary series, prompt a high level of memory recall upon boosting, and the fact that the presence of Th/CTL peptides is essential and principally responsible for the bulk of the T cell responses, while S1-RBD domain plays a minor role.

### Robust CD4^+^ and CD8^+^ T cell activities by Intracellular Cytokine Staining (ICS)

Along with high levels of ELISpot-based T cell responses, ICS analyses revealed again substantial Th1-dominant %CD4^+^ T cells producing IFN-γ and IL-2, versus low level of IL-4 (Fig 1B). Similar robust pattern was notable for %CD8^+^ T cells producing IFN-γ and IL-2 (Fig 1C).

Vaccine recipients also showed cytotoxic T-cell responses, including CD8^+^ T cells expressing cytotoxic markers CD107a and Granzyme B (Fig 1C) as observed in the primary series, accounting for a remarkable 3.6% of circulating CD8^+^ T cells after re-stimulation with S1-RBD + Th/CTL peptide pool, which persisted at a substantial 1.8% upon booster vaccination. Apparently, CD8^+^ T cell responses persisted robustly (50% of the high peak at Day 57) over 6–8 months as well. This suggests a potential of robust cytotoxic CD8^+^ T responses in favor of viral clearance once infection occurs.

### Overview of B cell immunogenicity on antigenic and functional levels

Of the 871 Phase-2 study participants designated for Immunogenicity investigation, 302 participants had their serum samples collected at pre-boosting and 14 days post-booster for antigenic assay by anti-S1-RBD IgG ELISA, and functional assays by ACE2:RBD_WT_ binding inhibition ELISA and by neutralization against live SARS-CoV-2 wild-type Wuhan strain (WT) by cell-based CPE method (S3 Fig). The results showed pronounced booster-induced increase of antibody titers that bound to RBD and inhibit/neutralize ACE2 interaction by respective 16- to 45-folds. These indicate that UB-612 booster vaccination could profoundly enhance both antigenic and functional activities.

### Neutralizing antibodies against WT, Delta, Omicron BA.1 and BA.2

**Fig 2:**
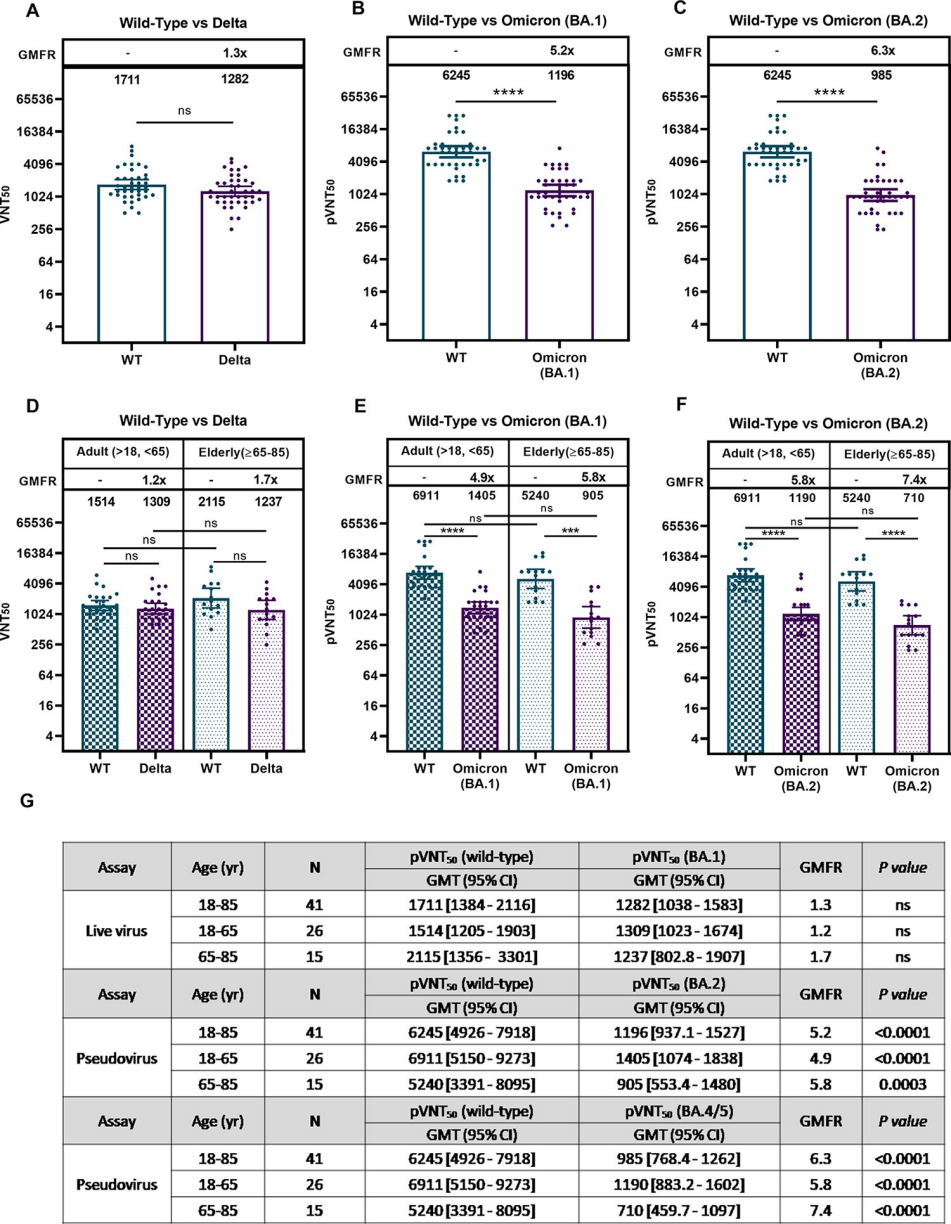
Viral-neutralization effects of UB-612 booster vaccination against wild type, Delta, Omicron BA.1 and BA.2 variants. Viral-neutralizing titers against SARS-CoV-2 wild-type, Delta, Omicron BA.1, and BA.2 variants were investigated during the infection pandemic that BA.1/BA.2 dominated. Serum samples from 41 participants (n = 27 for 18–65 years; n = 14 for 65–85 years) collected at 14 days post-booster were subjected to a live virus or pseudovirus-luciferase neutralization assay. (A) Live virus assay for Wuhan wild type WT vs. Delta for all ages and (B) live virus assay for WT vs. Delta for young adults and the elderly. (C) Pseudovirus assay for WT vs. BA.1 for all ages and (D) Pseudovirus assay WT vs. BA.1 for young adults and the elderly. (E) Pseudovirus assay for WT vs. BA.2 for all ages and (F) Pseudovirus assay WT vs. BA.2 for young adults and the elderly. The 50% viral-neutralizing antibody geometric mean titers (GMT, 95% CI) were measured, VNT_50_ for live virus and pVNT_50_. Statistical analysis was performed by the Student’s t-test (ns, *p*>0.05; ****, *p*<0.0001). No significant difference is notable between the two age groups in neutralization effect against WT, Delta, BA.1, and BA.2.

Functional blockade was further investigated comparatively on the occasion when Omicrons BA.1 and BA.2 dominate the pandemic scene. First, with limited available, affordable sources of viral variants, we investigated immune sera from 41 study participants across all age groups (18–65 years, n = 26; 65–85 years, n = 15). Neutralization measured using live virus, UB-612 booster elicited a neutralizing titer (VNT_50_) against WT at 1,711 versus Delta variant at 1,282 (Fig 2A), representing a 1.3-fold reduction (GMFR, Geomean Fold Reduction). There was no significant age-dependent neutralization effect between young adults (18–65 yrs.) and the elderly (65–85 yrs.) with respect to either anti-WT or anti-Delta VNT_50_ levels (Fig 2B), with a modest 1.2- to 1.7-fold GMFR of anti-Delta relative to anti-WT level.

As to Omicron BA.1 and BA.2 subvariants, when they sequentially dominated the pandemic scene, neutralization effects were measured by using pseudovirus for WT and Omicron subvariants. UB-612 booster elicited high neutralizing titers against WT at pVNT_50_ of 6,245; versus that against BA.1 at 1,196, representing a 5.2-fold reduction (Fig 2C); and versus against BA.2 at pVNT_50_ of 985, representing a 6.3-fold reduction (Fig 2E). There was no significant age-dependent booster-induced neutralization effect between young adults (18–65 yrs.) and the elderly (65–85 years) with respect to either anti-WT or anti-Omicron pVNT_50_ level (Fig 2D and 2F). Both age groups showed a 5.0- to 7.6-fold reduction for anti-BA.2 relative to anti-WT. By all accounts compared with BA.1, booster vaccination exhibits only a minor 1.2-fold lower neutralizing activity against BA.2.

### Neutralizing antibodies against WT, Omicron BA.1, BA.2, and BA.5

**Fig 3:**
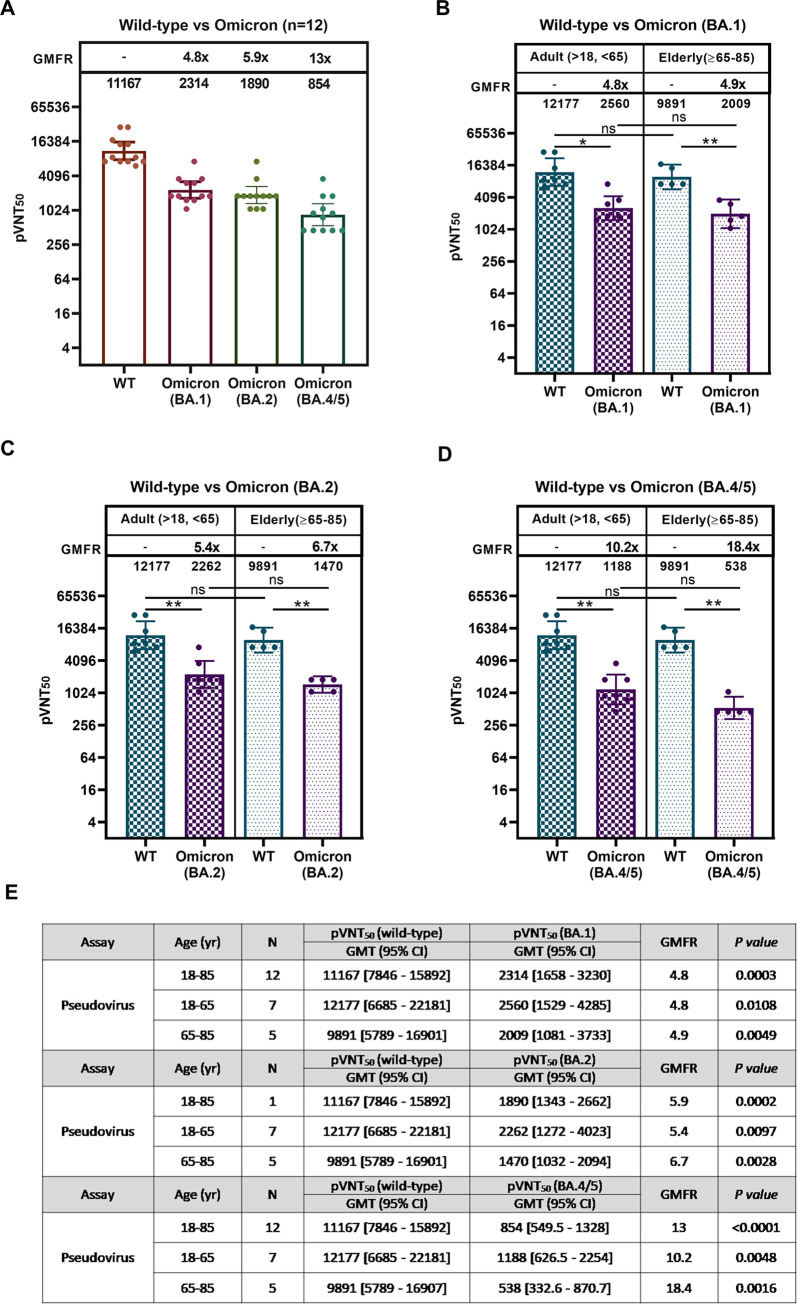
Comparative viral-neutralization effects against wild type strain, Omicron BA.1, BA.2, and BA.5 variants. At the SARS-CoV-2 pandemic when the Omicron BA.5 variant dominates, viral-neutralizing titers against wild-type, Omicron BA.1, BA.2, and BA.5 variants were measured using pseudovirus assay for comparison. Serum samples from 12 participants (n = 7 for 18–65 years; n = 5 for 65–85 years) collected at 14 days post-booster were subjected to pseudovirus-luciferase neutralization assay. (A) Wuhan wild type WT, BA.1, BA.2, and BA.5 for study participants of all ages, (B) WT vs. BA.1 for young adults and the elderly, (C) WT vs. BA.2 for young adults and the elderly, and (D) WT vs. BA.5 for young adults and the elderly. The 50% viral-neutralizing antibody geometric mean titers (GMT, 95% CI) were measured, pVNT_50_. Statistical analysis was performed by the Student’s t-test (ns, *p*>0.05; ****, *p*<0.0001). No significant difference is notable between the two age groups in neutralization effect against WT, BA.1, BA.2, and BA.5.

During the time when the pandemic was dominated by the ongoing BA.5 subvariant, we tested samples from 12 participants from the two groups (18–65 years, n = 7; 65–85 years, n = 5), with neutralization titers measured using pseudovirus. UB-612 booster elicited neutralizing titers of pVNT_50_ against WT/BA.1/BA.2/BA.5 at 11,167/2,314/1,890/854 (Fig 3A), representing a 4.8-/5.9-/13-fold reduction, respectively, relative to the anti-WT level. There was no statistically significant difference in age-dependent neutralization effect between young adults and the elderly within each of anti-WT/-BA.1/-BA.2/-BA.5 pVNT_50_ levels (Fig 3B–3D). By all accounts compared with BA.2, booster vaccination exhibits only a modest 2-fold lower neutralizing activity against BA.5.

### Potent, durable and correlative viral-neutralization and ACE2:RBD_WT_ binding inhibition

**Fig 4:**
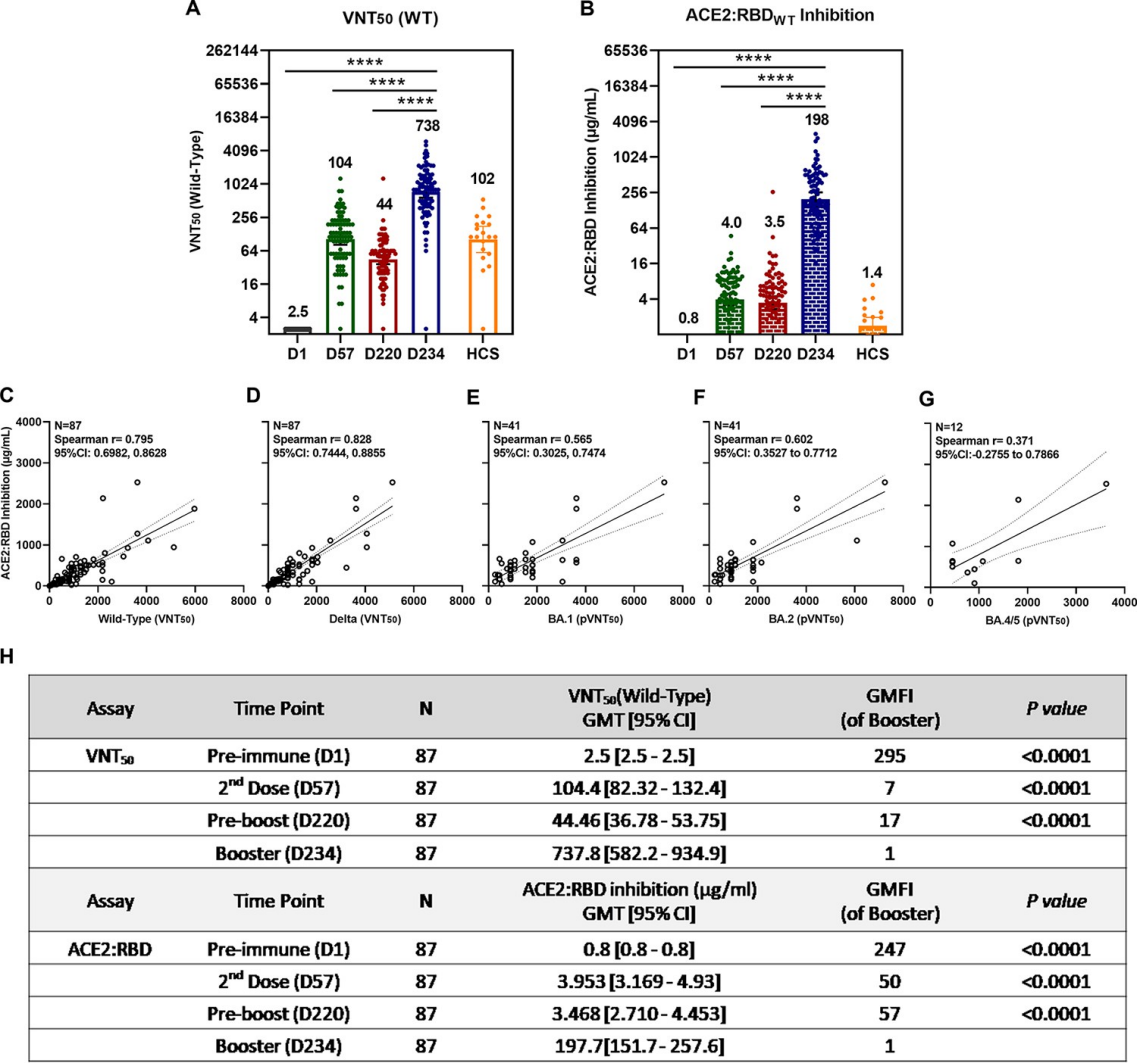
Functional correlations between ACE2:RBD_WT_ binding inhibition and viral-neutralization against Delta, Omicron BA.1, BA.2, and BA.5. Of 871 participants enrolled in the Phase-2 primary 2-dose series and grouped for Immunogenicity investigation, serum samples from 87 participants who had received a booster 3^rd^-dose of 100 μg UB-612 were collected at Day 1 (pre-dose 1), Day 57 (28 days post-dose 2), Day 220 (pre-booster between Days 197 to 242), Day 234 (14 days post-booster between Days 211 to 256). HCS from 20 SARS-CoV-2 infected individuals were also included for comparative testing by two functional assays: (A) viral-neutralizing titer (VNT_50_) against live wild-type Wuhan strain (WT) by CPE method; and (B) the antibody concentration calibrated with an internal standard for ACE2:RBD_WT_ binding inhibition by ELISA. The correlations are explored between the two function assays, i.e., ACE2:RBD_WT_ binding inhibition ELISA and the viral-neutralizing titers against the live virus (VNT_50_ for original wild-type and Delta strains) or the psuedovirus (pVNT_50_ for Omicron BA.1 strain). The RBD_WT_ stands for the RBD binding protein domain bearing amino acid sequence of the original SARS-CoV-2 wild-type (WT) Wuhan strain. The correlations were explored for (C) ACE2:RBD_WT_ inhibition vs. anti-WT VNT_50_, (D) ACE2:RBD_WT_ inhibition vs. anti-Delta VNT_50_, (E) ACE2:RBD_WT_ inhibition vs. anti-Omicron BA.1 pVNT_50_, (F) ACE2:RBD_WT_ inhibition vs. anti-Omicron BA.2 pVNT_50_, and (G) ACE2:RBD_WT_ inhibition vs. anti-Omicron BA.5 pVNT_50_. The correlation coefficients were evaluated by Spearman r with 95% CI. Statistical analysis was performed with the Student’s t-test (ns, *p*>0.05; ***, *p*<0.001; ****, *p*<0.0001). (H) Summary of geometric mean titer (GMT) and 95% CI are presented for plots as shown in panels (A) and (B).

We explored the correlation between viral-neutralizing activity (VNT_50_) and receptor binding inhibition (ACE2:RBD_WT_) using immune sera from 87 participants available on Day 1 (pre-dose), Day 57 (28 days post-2^nd^ dose), Day 220 (pre-boosting, 6 to 8 months post-2^nd^ dose), and Day 234 (14 days post-booster) which showed a high post-booster VNT_50_ titer of 738 (Fig 4A), a 17-fold increase over the pre-boosting (titer 44) and a 7-fold increase over the levels of both Day 57 (titer 104) and the human convalescent sera, HCS (titer 102).

In addition, a pronounced post-booster functional antibody-mediated inhibition of ACE2:RBD_WT_ binding inhibition was also observed at a high titer (expressed in standard-calibrated antibody concentration) of 198 μg/mL (Fig 4B), a ~57-fold increase over both the pre-boosting titer of 3.5 μg/mL and the Day 57 of 3.5 μg/mL, and a profound 140-fold over the HCS titer of 1.4 μg/mL. With increased monitoring time, the results again demonstrated that UB-612 induces a durable neutralizing antibody titer level, observed between Day 57 (post-2^nd^ dose) vs. Day 220 (pre-boosting), which represents a 42% retainment for VNT_50_ (titer, 104 vs. 44) against live WT virus (Fig 4A) and a 88% retainment for ACE2:RBD_WT_ (μg/mL, 4.0 vs. 3.5) binding inhibition (Fig 4B).

The inhibition of ACE2:RBD_WT_ binding on ELISA correlates well with anti-WT (Fig 4C) and anti-Delta VNT_50_ (Fig 4D) findings, both showed a similar high correlative Spearman’s rank correlation coefficients (r = 0.795 and 0.828, respectively). A lesser but significant correlation were also observed for ACE2:RBD_WT_ binding inhibition and anti-Omicron BA.1 and anti-BA.2 pVNT_50_, with Spearman’s correlation coefficients of r = 0.565 (Fig 4E) and r = 0.602 (Fig 4F). A lower yet substantial correlation was also noted with anti-BA.5 pVNT_50_ when only 12 sample points were available for regression analysis (Fig 4G).

### High neutralizing-titer correlation between pseudovirus and live virus assays

**Fig 5:**
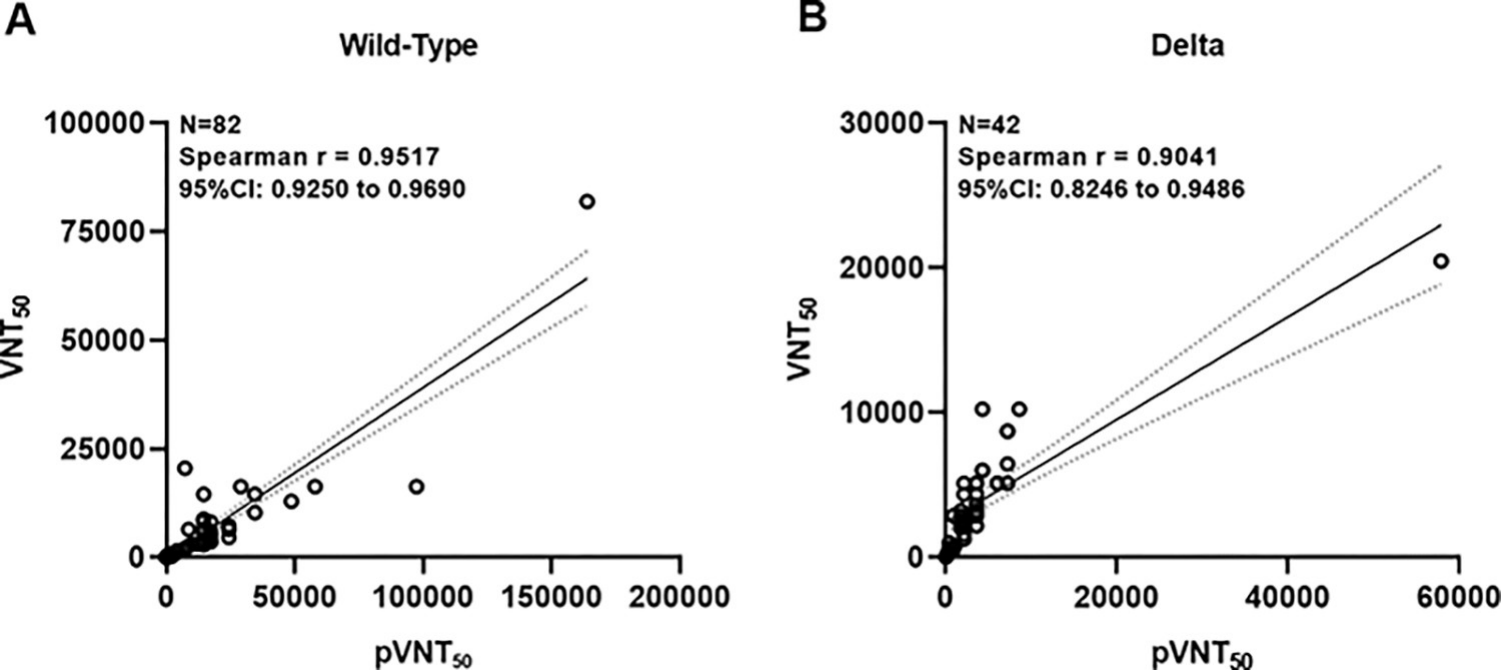
Functional correlations between pseudovirus and live virus neutralizing titers, pVNT_50_ and VNT_50_, against Wild-type and Delta strains. The correlations between two viral-neutralizing titer assays against pseudo-virus (pVNT_50_) and live-virus (VNT_50_) are explored. This case was only available from UB-612 vaccinees participated in the Phase-1 trial, whose serum samples were collected from primary series and booster vaccination and subjected to both neutralization assays against Wuhan wild-type virus (WT) and Delta strain. The correlation coefficients were evaluated by Spearman r with 95% CI.

The design of pseudovirus assay is based on contact with Spike protein only, while a live virus contains both Spike and non-Spike proteins that may behave differently in neutralizing strength. It is of high interest to address the issue as to whether an easier practice of pseudovirus assay could reflect the outcome from a live virus neutralization assay associated with UB-612 vaccination. It was found that the two viral-neutralizing titer assays against pseudo-virus (pVNT_50_) and live-virus (VNT_50_) are highly correlated, as exemplified by the assays against Wuhan wild-type virus (WT) with a Spearson r = 0.9517 (Fig 5A) and that against Delta strain presents a Spearson r = 0.9041 (Fig 5B).

## Discussion

The present Phase-2 UB-612 booster vaccination, proven safe and well tolerated without concerns of SAEs (S2 Fig), induces potent memory T cell immunity (Fig 1) that synergizes recalled B cell immunity with striking cross-neutralizing antibodies against WT, Delta and Omicrons (Figs 2 and 3). Of notable clinical interest, the booster uplifts a lower neutralizing antibody titer in the elderly [48] to a high level close to that in young adults regardless of viral mutant status (Figs 2 and 3). In addition, blockade of S1-RBD binding to ACE2 receptor correlates well with viral neutralization (Fig 4). Thus, the UB-612 vaccine platform, due to broadly recognizing conserved Th/CTL epitopes on Spike and non-Spike proteins, can maintain a target plasticity without much mutational distortion within the RBD domain of the target B immunogen. The present report reveals five salient findings.

Firstly, on the magnitude of pseudovirus-neutralizing titer (pVNT_50_/ID_50_) against Omicrons, UB-612 booster appears to implicate a competitive edge by contrast (Table 3) over other EUA approved vaccines using different platforms. While the rank order of pVNT_50_ against Omicrons BA.1/BA.2/BA.5 trends downward (Fig 3A) and UB-612 booster combats the most contagious BA.5 with a 13-fold reduction relative to anti-WT titer, its anti-BA.5 pVNT_50_ titer of 854 represents a substantially high neutralizing activity. In contrast, an anti-BA.5 pVNT_50_ titer of 582 was reported for NVX-CoV2373 [49], 378 for mRNA-1273 [7], 360 for BNT162b2 [2,3], 75 for CoronaVac [6] and 43 for AZD1222 [2] (Table 3). All available anti-BA.5 pVNT_50_ values account for approximately an 8.4- to 21-fold reduction. There is a stark vaccine platform-dependent difference in the anti-BA.5 potency.

**Table 3:** Viral-neutralizing antibody titers assessed by pseudotyped virus neutralization assay (pVNT_50_ and ID_50_) against SARS-CoV-2 wild-type (WT) and Omicron BA.1, BA.2, and BA.5 subvariants upon homologous boosting.

Vaccines^a^ (Homo-booster)^a^	Participants (No./Reference)	NeuAb assay (Method/Unit)	WT (GMT)^b^	Omicrons BA.1/BA.2/BA.5 (GMT)^b^	WT/Omicrons BA.1/BA.2/BA.5 (GMFR)
UB-612 (Ph-1)	(n = 45)/(ref. 48)	PNA/pVNT_50_	12,778	2,325/1993/ND	5.5/6.4/ND
UB-612 (Ph-2)^c^	(n = 41)/(present)	PNA/pVNT_50_	6,254	1,196/985/ND	5.2/6.3/ND
UB-612 (Ph-2)^c^	(n = 12)/(present)	PNA/pVNT_50_	11,167	2,314/1,890/854	4.8/5.9/13.0
BNT162b2	(n = 24)/(ref. 20)	PNA/pVNT_50_	6,539	1066/776/ND	6.1/8.4/ND
BNT162b2	(n = 19)/(ref. 2)	PNA/pVNT_50_	4,122	1,116/1,113/360	3.7/3.7/11.5
BNT162b2	(n = 27)/(ref. 3)	PNA/ID_50_	5783	900/829/275	6.4/7.0/21.0
NVX-CoV2373	(n = 48)/(ref. 49)	PNA/ID_50_	10,862	1,197/ND/582	9.1/ND/18.7
mRNA-1273	(n = 16)/(ref. 7)	PNA/ID_50_	4,679	945/780/378	5.0/5.9/12.4
MVC-COV1901	(n = 15)/(Ref. 50)	PNA/ID_50_	1,280–640	160-80/ND/ND	8.7/ND/ND
CoronaVac	(n = 40)/(Ref. 6)	PNA/pVNT_50_	632	122/122/75	5.2/5.2/8.4
AZD1222	(n = 41)/(Ref. 2)	PNA/pVNT_50_	516	89/76/43	5.8/6.8/12
BBIBIP-CorV	(n = 75)/(ref. 51)	PNA/pVNT_50_	295	15/ND/ND	20.1/ND/ND

Abbreviation: PNA = pseudotyped virus neutralization assay; GMT = geometric mean titer; GMFR = geometric mean fold reduction relative to WT; WT = wild type strain of SARS-CoV-2; Omicrons = Omicron subvariants BA.1/BA.2/BA.5; ND = not determined. pVNT_50_ & ID_50_ = 50% neutralization GMT by pseudoviruss assay

^a^ Vaccines reported of homologous booster (third dose) vaccination.

^b^ GMTs against WT measured at 14 or 28 days post-booster third dose.

^c^ UB-612—Pseudovirus assays conducted with sera of subset participants (Phase-2 booster extension study) when Omicron infection were dominated sequentially by BA.2 and BA.5 subvariant.

High anti-WT/anti-BA.1 pVNT_50_ titers of 12,778/2,352 for UB-612 were first observed in our Phase-1 booster vaccination study [48] (S4B Fig). As to “anti-WT vs. anti-BA.1 vs. anti-BA.2,” UB-612 booster exhibits a combined pVNT_50_ value profile of 6,254–12,778 vs. 1,196–2,325 vs. 985–1,993” (Table 3 and S4C Fig), which appear to be comparable to the respective counterparts reported for NVX-CoV2373 [49], mRNA-1273 [7], BNT162b2 [2], yet appear to be far greater than for MVC-COV1901 [50], CoronaVac [6], AZD1222 [2], and BBIP-CorV [51]. All listed anti-BA.1/BA.2 pVNT_50_ values account for approximately a ~3.7- to 20-fold reduction; and, relative to anti-BA.1/-BA.2, the overall anti-BA.5 pVNT_50_ is estimated to be within 2- to 3-fold reduction.

Overall, UB-612 booster appears to perform on a par with or to bear a competitive edge over other vaccine platforms based on pseudovirus-neutralizing pVNT_50_, against Omicron BA.1/BA.2/BA.5. From a comparative view of between-vaccine platforms, the magnitude of viral-neutralizing strength would matter much more than a GMFR factor.

It should be noted that both pseudovirus pVNT_50_ discussed above (Table 3) and live virus VNT_50_ data to be discussed below (Table 4) have drawbacks as all assay methods by various vaccine platforms are not uniformly comparable. No standardized neutralization methods have been set to follow with. These data points are laid out for contrast, not for comparison purpose (with statistics). Nonetheless, a solid trend of platform-dependent difference in viral-neutralization potency is discernable.

**Table 4:** Viral-neutralizing antibody titers assessed by live virus neutralization assay (VNT_50_, PRNT_50_, and FRNT_50_) against SARS-CoV-2 wild-type (WT) and Omicron BA.1/BA.2 subvariants upon homologous boosting.

Vaccines^a^ (Homo-booster)^a^	Participants (No./Reference)	NeuAb assay (Method/Unit)	WT (GMT)^b^	Omicrons BA.1/BA.2 (GMT)^b^	WT/Omicrons BA.1/BA.2 (GMFR)
UB-612	(n = 15)/(ref. 52)	MNA/VNT_50_	6,159	670/485	9.2/12.7
mRNA-1273	(n = 20)/(ref. 7)	FRNT/ID_50_	1659	81.0/ND	20.5/ND
BNT162b2	(n = 20)/(ref. 7)	FRNT/ID_50_	640	46.2/ND	13.3/ND
BNT162b2	(n = 30)/(ref. 35)	PRNT/VNT_50_	673	106/ND	6.3/ND
AZD1222	(n = 41)/(Ref. 53)	FRNT/FRNT_50_	723	57.0/ND	12.7/ND

Abbreviation: MNA = Microneutralization assay; PRNT = plaque reduction neutralization test; FRNT = focus reduction neutralization test; GMT = geometric mean titer; GMFR = geometric mean fold reduction relative to WT; WT = wild type strain of SARS-CoV-2; Omicrons = Omicron subvariants BA.1/BA.2; ND = not determined. pVNT_50_ & ID_50_ = 50% neutralization GMT by live virus assay; VNT_50_, ID_50_ & FRNT_50_ = 50% neutralization GMT by live virus assay.

^a^ Vaccines reported of homologous booster (third dose) vaccination.

^b^ GMTs against WT measured at 14 or 28 days post-booster third dose.

Secondly, at the level of live virus-neutralizing titer (VNT_50_/ID_50_/FRNT_50_) against Omicron BA.1 and BA.2 (Table 4), UB-612 booster excels over other vaccine platforms. UB-612 booster elicits an anti-WT/anti-BA.1 titer profile of 6,159/670 [52], in contrast with 1,699/81.0 for mRNA-1273 (7), 640-673/46.2–106 for BNT162b2 [7,35], and 723/57 for AZD1222 [53]. UB-612’s anti-BA.1 titer of 670 far exceeds other vaccines by ~6- to 12-fold higher. It is important to note that other vaccine platforms exhibit a low anti-BA.1 level at peak response (28 days post-booster), which requires a fourth-dose (the 2^nd^ booster) to compensate the dwindling neutralizing antibodies.

In addition, UB-612 booster presents a substantially high anti-BA.2 live-virus titer VNT_50_ at 485, which is even far greater than the anti-BA.1 titers observed with other vaccines. In light of the true measure for neutralizing activity, the live virus assay would reflect better than the pseudovirus assay, as the former stands for the combined anti-viral activity against both Spike and non-Spike proteins, while the latter assay measures the strength against the Spike only.

Similar high neutralizing titers against live WT/Delta virus (VNT_50_) have been noted earlier for UB-612 in the Phase-1 booster study [48]. By contrast, the post-booster VNT_50_ values against WT/Delta ranged from the low-end 122/54 (CoronaVac) to the high-end 3,992/2,358 (UB-612) (S1 Table). In the present Phase-2 booster study, UB-612 reproduces a high anti-Delta VNT_50_ at 1,282, only a 1.3-fold lower than the anti-WT live virus (Fig 2A).

Collectively, UB-612 booster performs on a par with or bears a competitive edge over other vaccine platforms in viral-neutralization potency, either pseudovirus or live virus, against Delta and Omicron BA.1, BA.2, and potentially the currently dominating BA.5.

Thirdly, UB-612 booster uplifts a lower viral-neutralizing titer generally associated with the elderly to a level approximately the same as that in the young adults. No significant age-dependent neutralization effect is evident between young adults and the elderly with respect to humoral immune responses against WT/Delta/BA.1/BA.2/BA.5 (Figs 2B, 2D, 2F and 3B–3D). This is of high clinical significance as elderly people, due to a decline in pathogen immunity, do not respond to immune challenge as robustly as young adults and so have a reduction in vaccine efficacy [54]. Thus, UB-612 as a primer or booster has a potential benefit not only for the elderly but also for immunocompromised people in general.

Fourthly, the strong blockade of ACE2:RBD_WT_ interaction (Fig 4B) correlates well with viral-neutralizing VNT_50_ (live virus WT and Delta) and pVNT_50_ (pseudovirus BA.1) (Fig 4C–4E). The positive functional correlation infers a substantial clinical efficacy against COVID-19. Indeed, using models of S protein binding activities [55] and neutralizing antibodies [56], the clinical efficacy of 2-dose primary immunization of UB-612 is predicted to be 70–80% and a booster vaccination may lead to 95% efficacy against symptomatic COVID-19 caused by the ancestral Wuhan strain [52]. The clinical efficacy protecting against infection of circulating subvariants including the dominant BA.5 would await outcome of an ongoing Phase-3 trial that compares UB-612 with authorized vaccines under homologous and heterologous boosting [ClinicalTrials.gov ID: NCT05293665].

The pronounced, broadly-neutralizing profiles illustrate one unique feature of UB-612, i.e., the serum neutralizing antibodies are directed solely at the critical receptor binding domain (RBD) that reacts with ACE2. In contrast with the currently authorized full-length S protein-based vaccine platforms, UB-612’s RBD-only design leaves little room in non-conserved sites of S protein for viral mutation to occur and so may result in less immune resistance.

Thus, booster vaccination can prompt recall of high levels of parallel anti-WT neutralizing VNT_50_ (Fig 4A) and RBD-ACE2 binding inhibition antibodies (Fig 4B**)**, and both functional events are durable over Day 57 and Day 220 with a substantial 42%/88% retainment at 6 months or longer after the second shot. This is consistent with the long-lasting anti-WT VNT_50_ effect with a half-life of 187 days observed in the Phase-2 primary series, in which a ~50% retainment was observed at 6 months relative to the peak response [48].

Further, the finding that the UB-612 induced a 140-fold higher increase in blocking the RBD:ACE2 interaction than by human convalescent sera (HCS) (Fig 4B) suggests that most of the antibodies in HCS may bind allosterically to the viral spike (N- or C- terminal domain of the S) rather than orthosterically to the RBD sites, which may include non-neutralizing anti-S antibodies to cause unintended side effects or Antibody-Dependent Effect (ADE) event. This warrants further investigation including sera from re-infections and breakthrough infections from all vaccine platforms.

Fifthly, UB-612 booster induces potent, durable Th1-oriented (IFN-γ^+^-) responses (peak/pre-boost/post-boost SFU/10^6^ PBMCs, 374/261/444) along with robust presence of cytotoxic CD8^+^ T cells (peak/pre-boost/post-boost CD107a^+^-Granzyme B^+^, 3.6%/1.8%/1.8%) (Fig 1). Vaccines designed to produce a strong systemic T cell response targeting conserved nonmutable epitopes may prevent immune escape and protect against current and future viral variants that causes COVID-19 [57,58]. Along the same vein of promoting T cell immunity, UB-612 armed with the pool of sequenced-conserved Th/CTL epitope peptides (S2x3, M, and N) (Table 2) has demonstrated to elicit a striking, durable Th1-predominant IFN-γ^+^ T cell response in Phase-1 primary vaccine series that peaked at 254 SFU/10^6^ cells and persisted with a ~50% retainment over 6 months post-2^nd^ dose (121 SFU/10^6^ cells) [48].

The Phase-1 booster-recalled T cell immunity is consistent with an even higher 70% sustaining T cell immunity (peak 374 vs. pre-boost 261 SFU/10^6^ cells) in the present Phase-2 primary series that surges to a 444 SFU/10^6^ cells two weeks post-booster (Fig 1A and 1D), which leads to a pronounced, durable cytotoxic (CD107a^+^-Granzyme B^+^) activity of CD8^+^ T lymphocytes (CTL) with high frequency levels (1.8%-3.6%) (Fig 1C and 1F).

UB-612 booster appears to trigger far greater T cell responses than those produced by the current Spike-only mRNA (BNT162b2) and adeno-vectored (ChAdOx1) vaccines [59]: e.g., the pre-boost/post-boost level of SFU/10^6^ cells (related to Delta strain) under homologous boosting for the 3-dose of ChAd/ChAd/ChAd were 38/45 and that of BNT/BNT/BNT were 28/82; and those under heterologous boosting were 42/123 for ChAd/ChAd/BNT and 36/108 for BNT/BNT/ChAd. For those currently licensed COVID vaccines, it is worthy to note that the fourth vaccine jab (the 2^nd^ booster) does not increase T cell response [60]: e.g., the 28 days post-3^rd^ dose/pre-4^th^ dose/14 days post-booster level of SFU/10^6^ cells (related to Delta strain) for the 4-dose of ChAd/ChAd/BNT/BNT were 133/19/108 and that of BNT/BNT/BNT/BNT were 62/14/80.

The lackluster booster-recalled T cell immunity seen with mRNA and adeno-vectored vaccines [59,60] may reflect the dwindling, weakened B cell humoral responses and clinical efficacy. A booster 3^rd^-dose of mRNA vaccines could compensate the waning immunity and reduce rates of hospitalization and severe disease, yet be less effective in protection against mild and asymptomatic infections [31–36]. At the time of Omicron BA.1 on the rise, vaccine effectiveness was seen reduced after booster (third dose) of mRNA vaccines in protection against COVID symptoms (45% at 10 weeks) [61] and hospital admission (55% at 12 weeks) [62].

In two retrospective large cohort studies, the elderly (aged ≧60) receiving the fourth dose of BNT162b2 (2^nd^ booster) while BA.2 infection was dominant also showed a modest and transient efficacy against severe disease (~60–75% protection, relative to the 1^st^ booster third dose) [63,64], and the effectiveness against infection completely wanes after 8 weeks [63].

Breakthrough infection could occur after the fourth dose [37], in particular amid the circulation of the dominant Omicron BA.5. The booster-compensated protection effectiveness offered by mRNA vaccines could be blunted soon upon boosting. Incessant, short-interval boosting with current mRNA vaccines could result in dwindling and weakened immune responses against Omicrons [65], for mechanisms remained to be elucidated.

While a substitute of the fourth dose with mRNA-1273 can elevate T cell response to a level of ~240 SFU/10^6^ cells [60], the increased response level appears to be lower than those by UB-612 at 261 SFU/10^6^ cells pre-3^rd^-dose boosting and at 444 SFU/10^6^ cells 14 days post-booster (third dose) (Fig 1). The UB-612 vaccine, designed to target multiple conserved epitopes on both Spike and non-Spike proteins, could have underpinned the base for a fuller T cell immunity.

The potential clinical significance of a striking T-cell immunity elicited by UB-612 vaccine platform is supported by the development of a plain T-cell vaccine (CoVac-1) containing a six-peptide backbone that, as a T-cell booster, triggered dramatic multifunctional CD4 and CD8 T-cell responses [66], which showed benefits to B-cell deficient, immunocompromised patients who could not mount B-cell antibody responses. The facts that potent memory CD4 and CD8 T cell memory can protect against SARS-CoV-2 infection in the absence of immune neutralizing antibodies [57,58,66] raises concerns over the fact that humoral antibody response has long been used as a sole bridging metric of protective immunity [67], which lacks full understanding of human post-vaccination immunity as antibody response is generally shorter-lived than virus-reactive T cells [68–70].

Further, the SARS-CoV-2’s non-Spike structure E, M and N proteins are the regions critically involved in the host cell interferon response and T-cell memory [41–43]. These structural proteins of virus’ main body when picked up by Antigen Presenting Cells (APC) and presented as viral peptides would fall beyond recognition by the currently authorized vaccines that are based on the outer spike protein only. Th1 cells help to stimulate B cells to make antibodies, and they can morph as well into memory helper CD4^+^ and cytotoxic CD8^+^ T cells to provide a long-lasting immune response [71]. UB-612’s booster-enhanced broader, durable B and T cell immunity may make Omicron evasion less likely as the booster vaccination could behave closer to the breadth of natural, infection-induced immunity.

While neutralizing antibodies can block ACE2:RBD interaction and protect against initial infection, the non-Spike protein cross-reactive memory T cell immunity is essential for protection from severe disease and for long-term prevention against infection; and, as such, T cell immunity should be recognized as a measure for long-term vaccine success [72–79]. The role of T cells, in particular the recognition against non-Spike targets and the associated T cell responses, has long been underestimated and overlooked from the outset of COVID vaccine development.

The cross-reactive T cell responses can limit disease severity, reduce viral replication, and limit the duration of illness, and these potential durable immune responses revealed by UB-612 (Fig 1) would be a key component of a pan-SARS-CoV-2 vaccine. To what extent that vaccine booster-induced memory T cell immunity would contribute to vaccine effectiveness in the clinic against COVID-19 infection of any degree, as a leading actor or a supportive cast, has become a research subject of major clinical interest [80].

Of additional clinical interest with strong T cell immunity is its function of viral clearance. Persistent SARS-CoV-2 infections can contribute to long COVID as residual viable SARS-CoV-2 particles, viral replication, viral RNA and viral spike protein antigens could sustain in tissues of the convalescents [81–83]. As long COVID is found to be associated with a decline in IFN-γ-producing CD8^+^ T cell [84], enhancing T cell immunity for clearance of residual systemic infection (sustained viral reservoirs) could be a sensible strategy for prevention of long COVID.

Facing the dwindling vaccine effectiveness and emergence of viral variants with higher infectivity and immune evasion, development of composition-updated vaccines [38,39] or universal coronavirus vaccines [40] has been strongly advocated. To meet an urgent need and for a long-term fight against new mutants, one would look beyond the practice of frequent short-interval booster jabs and resist clinging to use of variant-specific (e.g., omicron-updated) vaccines.

In fact, the recent bivalent vaccine mRNA-1273.214 (original wild-type Spike plus Omicron BA.1 Spike) as the fourth dose (second booster) was found to result in only 1.7-fold higher pseudovirus-neutralizing antibody titer (pVNT_50_) against BA.5 as compared to that by the original mRNA-1273 [85]. The extra modest anti-BA.5 gain of pVNT_50_ titer by the bivalent WT/BA.1 vaccine may not provide better protection efficacy against BA.5 infection.

Furthermore, bivalent WT/BA.5 mRNA vaccines (Pfizer and Moderna) at the 4^th^ dose in two studies have shown only 1.2 to 1.3-fold higher pVNT_50_ neutralizing titer against BA.5 than the original wild type [86,87]. And, the bivalent booster-induced T cell response remained unchanged at a low level [87], relative to the original vaccine. These observations are in line with the mechanism of Immune Imprinting [88] that tips the bulk of antibodies to react with the first encounter wild-type strain or the initial vaccine type an individual exposed to, implicating also that variant-specific vaccine would not perform better than thought.

Of interest to note, to enhance vaccine immunity, a T cell vaccine BNT162b4 targeting conserved epitopes on non-Spike proteins, in combination with BNT162b5 BA.5-bivalent or BNT162 BA.1-bivalent vaccine, is being developed [ClinicalTrials.gov ID: NCT05541861]. The goal of the two vaccines in one shot is to deliver durable antibody and T-cell immune protection against severe disease and hospitalization for at least one year.

By and large, a pragmatic approach to curbing ever-emergent new mutants would be “universal (pan-Sarbecovirus) vaccines” targeting conserved nonmutable epitopes on Spike and non-Spike proteins of coronavirus. In that sense, a shift of Spike-only vaccine design to a paradigm by targeting conserved epitopes on both Spike and non-Spike proteins would be a workable option. To be competent for next-generation vaccines, conserved regions on non-Spike proteins (membrane and nucleocapsid) to serve as immunogens may also contribute to the development of pan-betacoronavirus vaccines [89].

By incorporation of five sequence-conserved Th/CTL epitope peptides [90] and a sixth idealized universal Th peptide which serves as catalyst in T cell activation [48], the UB-612-induced T cell immunity may enhance the clearance of the virally infected cells, regardless of Omicrons or future mutants, as their mutation sites are not to overlap any of the amino acid residues on the precision-designed S2, N, and M epitope peptides that are highly conserved (or rarely mutate) across all VoCs (Table 2). By design, UB-612 could provide strong memory T cell immunity that associates with potent, broadly-recognizing and durable live virus-neutralizing effect without resorting to Omicron-specific immunogens. Whether a strong, fuller and broadly-recognizing T cell immunity could help with prevention/minimization of long COVID warrants additional clinical research.

In summary, we have simultaneously characterized the booster-enhanced B- and T-cell immunity in a large (N = 1,378) Phase-2 study, demonstrating UB-612 can elicit a fuller T cell immunity that comprehensively recognize Spike (S1-RBD and S2) and non-Spike structure N and M proteins, which seeds the potential for viral clearance upon infection; and the induced B cell responses would broadly neutralize all VoCs regardless of varying mutational epitope locations. Our UB-612 multitope vaccine may serve as a universal vaccine primer and booster to ward off all VoCs and future mutants, for which a US-FDA approved CEPI supported large-scale Phase-3 trial has also been underway to further evaluate the concept of protection efficacy.

## Supporting information

S1 Fig Flow of UB-612 Phase-2 primary 2-dose series with extension booster. The study design of the Phase-2 primary 2-dose series (100 μg dose; 28 days apart) of UB-612; and the extension study of booster vaccination [NCT04773067] conducted between Oct. 16, 2021 and Apr. 16, 2022. (A) Of the primary series (n = 3,875), a total of 1,478 participants (aged at 18–85 years) were enrolled to receive the booster third-dose of 100 μg UB-612; (B) the characteristics of the study participants in the primary and booster series.
(DOCX)

S2 Fig Incidence of adverse effects in the Phase-2 primary 2-dose and extended booster third-dose series. (A) Solicited local adverse reaction within 7 days after each vaccination. (B) Skin allergic reaction within 14 days after each vaccination. (C) Solicited systemic adverse reaction events 7 days after each vaccination (Doses 1 and 2 in the primary series; Dose 3 as a booster)
(DOCX)

S3 Fig Phase-2 immunogenicity overview (antigenic and functional) on homologous boosting. Immunogenicity overview are presented in (A) antigenic S1-RBD_WT_ binding, ACE2:RBD_WT_ binding inhibition, and anti-WT viral-neutralizing activity VNT_50_ and (B) the **s**ummary of geometric mean titer (GMT) with 95% CI. A total of 302 participants (n = 208 for aged 18–65 years; n = 94 for aged 65–85 years) received a booster 3^rd^-dose. The serum samples of 302 participants were collected at the indicted time points, Days 197 to 242 (the pre-booster day) and Days 211 to 256 (14 days post-booster), and tested for neutralizing antibody levels that inhibit 50% of live SARS-CoV-2 wild-type, expressed as VNT_50_ (WT, Wuhan strain) (functional), the inhibitory titers against S1-RBD binding to ACE2 by ELISA, expressed as μg/mL (functional), and anti-S1-RBD IgG antibody titers by ELISA (antigenic). Statistical analysis was performed by the Student’s t-test (ns, *p*>0.05; **** *p*<0.0001).
(TIF)

S4 Fig Viral-neutralizing titers against live SARS-CoV-2 wild type (Wuhan) and Delta variant (VNT_50_), and pseudo SARS-CoV-2 wild type (Wuhan) and Omicron BA.1 and BA.2 variants (pVNT_50_) after the booster third-dose in the Phase-1 trial*. Geometric mean titers (GMT) at 50% viral-neutralization observed 14 days after the booster third-dose of 100-μg administered at mean Day 286 (Days 255–316) after the primary 2-dose series (Days 0 and 28) of the 196-day Phase-1 trial. (A) In the participants of the 100-μg group (n = 18) with healthy adults aged at 20–55 years, the post-booster VNT_50_ titer reached at 3,992 against live SARS-CoV-2 Wuhan wild-type, and at 2,358 against live Delta variant. (B) Similarly, unusually high post-booster pVNT_50_ against Wuhan wild-type pseudovirus at 12,778, and at 2,325 against Omicron BA.1. (C) High post-booster pVNT_50_ against Omicron BA.2 as well. (D) Summary of geometric mean titer (GMT) with 95% CI are presented for plots shown in panels A—C. *Fig 4A and 4B adapted with permission from *J*. *Clin*. *Invest*. 2022;132(10):e157707. https://doi.org/10.1172/JCI157707.
(TIF)

S1 Table Comparison of post-booster viral-neutralizing antibody titers against SARS-CoV-2 wild-type (WT) and Delta variant by vaccines from different platforms. (DOCX)

S1 Methods Vaccine product and placebo. (DOCX)

S2 Methods Viral-neutralizing antibody titers against SARS-CoV-2 wild-type and variants by CPE based live virus neutralization assay. (DOCX)

S3 Methods Neutralizing titers against Omicron BA.1/BA.2/BA.5 by pesudovirus assay. (DOCX)

S4 Methods Inhibition of RBD_WT_ binding to ACE2 by ELISA. (DOCX)

S5 Methods Anti-S1-RBD_WT_ binding IgG antibody by ELISA. (DOCX)

S6 Methods T cell responses by ELISpot. (DOCX)

S7 Methods ICS. (DOCX)

S8 Methods Statistics. (DOCX)

S1 Appendix Phase-2 study V-205 protocol. (PDF)

S2 Appendix Phase-2 study V-205 IRB approval letters. (PDF)

S3 Appendix Phase-2 study V-205 ICF. (PDF)
