# Supplementary material for: Toward a pan-SARS-CoV-2 vaccine targeting conserved epitopes on spike and non-spike proteins for potent, broad and durable immune responses
Source: PLoS Pathog. 2023 Apr 20;19(4):e1010870. doi: 10.1371/journal.ppat.1010870 (PMC10153712; doi:10.1371/journal.ppat.1010870)
Supplement: S1 Fig — The study design of the Phase-2 primary 2-dose series (100 μg dose; 28 days apart) of UB-612; and the extension study of booster vaccination [NCT04773067] conducted between Oct. 16, 2021 and Apr. 16, 2022. (A) Of the primary series (n = 3,875), a total of 1,478 participants (aged at 18–85 years) were enrolled to receive the booster third-dose of 100 μg UB-612; (B) the characteristics of the study participants in the primary and booster series. (DOCX) [file ppat.1010870.s001.docx]

**Supporting Information - Supporting Figure 1**


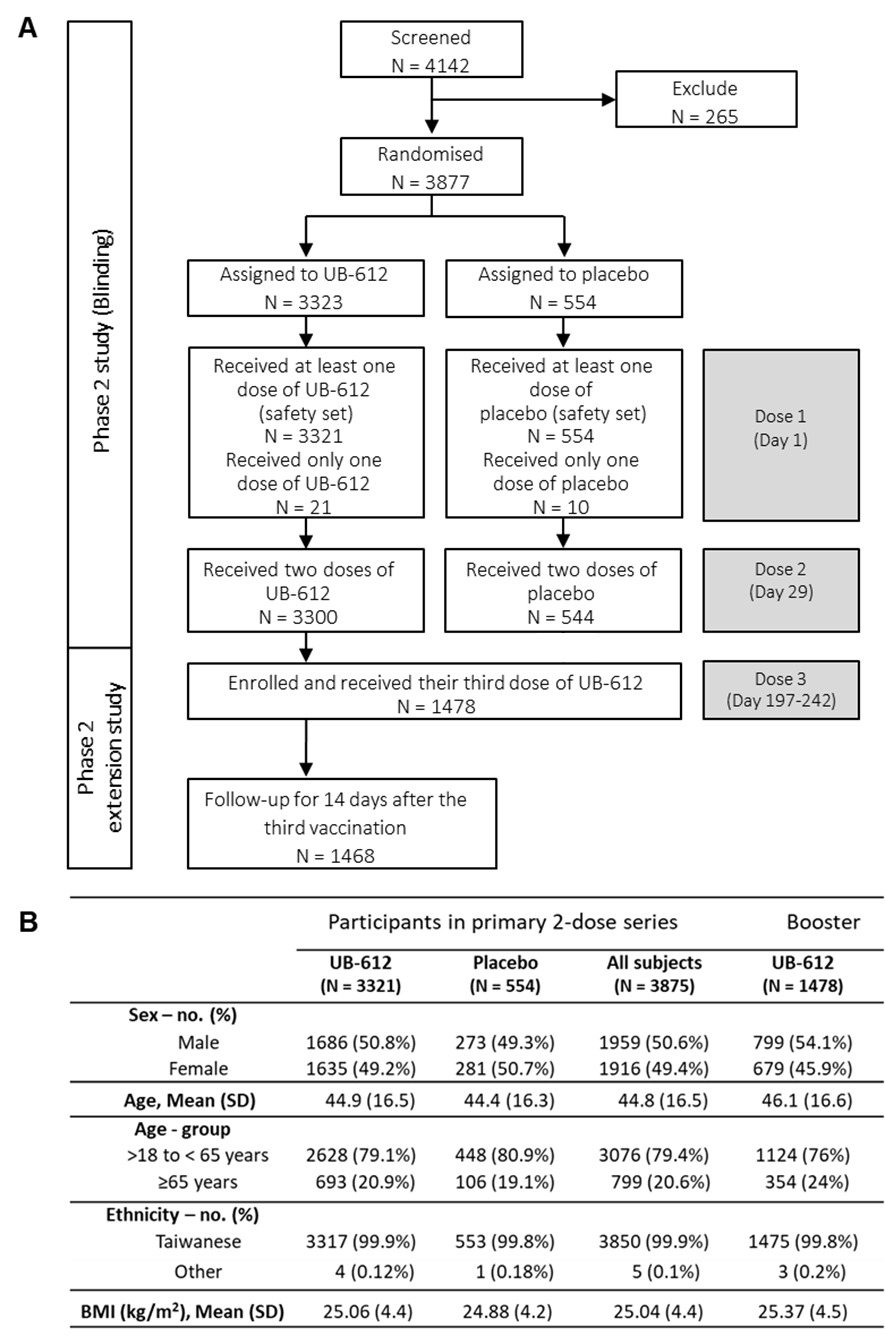


**S1 Fig. Flow** **of UB-612 Phase-2 primary 2-dose series with extension booster.**

The study design of the Phase-2 primary 2-dose series (100 μg dose; 28 days apart) of UB-612; and the extension study of booster vaccination [NCT04773067] conducted between Oct. 16, 2021 and Apr. 16, 2022. **(A)** Of the primary series (n = 3,875), a total of 1,478 participants (aged at 18-85 years) were enrolled to receive the booster third-dose of 100 μg UB-612; **(B)** the characteristics of the study participants in the primary and booster series.
