# Supplementary material for: Toward a pan-SARS-CoV-2 vaccine targeting conserved epitopes on spike and non-spike proteins for potent, broad and durable immune responses
Source: PLoS Pathog. 2023 Apr 20;19(4):e1010870. doi: 10.1371/journal.ppat.1010870 (PMC10153712; doi:10.1371/journal.ppat.1010870)
Supplement: S2 Fig — (A) Solicited local adverse reaction within 7 days after each vaccination. (B) Skin allergic reaction within 14 days after each vaccination. (C) Solicited systemic adverse reaction events 7 days after each vaccination (Doses 1 and 2 in the primary series; Dose 3 as a booster) (DOCX) [file ppat.1010870.s002.docx]

**Supporting Information - Supporting Figure 2**

**
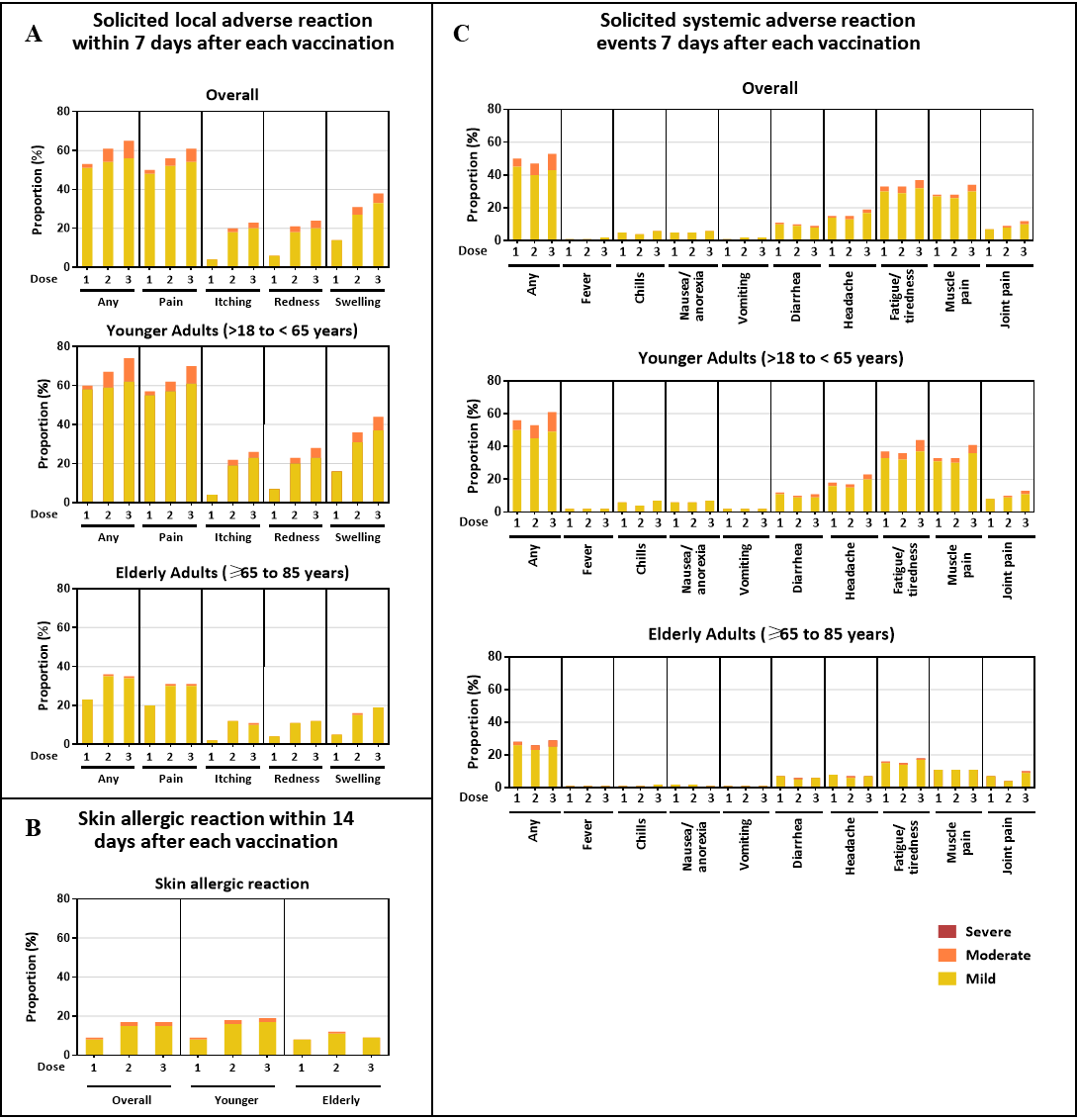
**

**S2 Fig. Incidence of adverse effects in the Phase-2 primary 2-dose and extended booster third-dose series.**

**(A)** Solicited local adverse reaction within 7 days after each vaccination. **(B)** Skin allergic reaction within 14 days after each vaccination. **(C)** Solicited systemic adverse reaction events 7 days after each vaccination (Doses 1 and 2 in the primary series; Dose 3 as a booster).
