# Supplementary figures and images for: Toward a pan-SARS-CoV-2 vaccine targeting conserved epitopes on spike and non-spike proteins for potent, broad and durable immune responses
Source: PLoS Pathog. 2023 Apr 20;19(4):e1010870. doi: 10.1371/journal.ppat.1010870 (PMC10153712; doi:10.1371/journal.ppat.1010870)

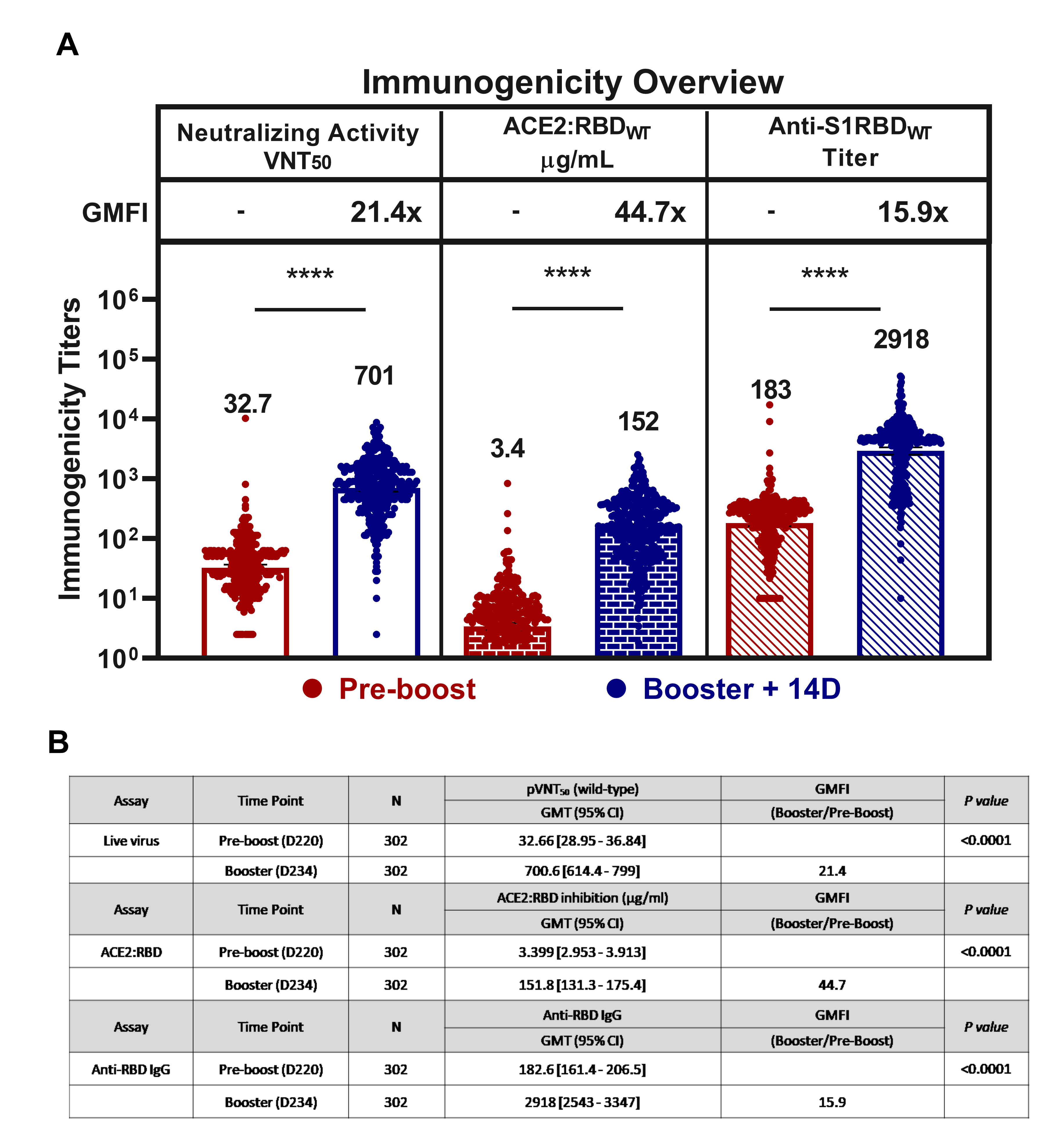

Supplement: S3 Fig — Immunogenicity overview are presented in (A) antigenic S1-RBDWT binding, ACE2:RBDWT binding inhibition, and anti-WT viral-neutralizing activity VNT50 and (B) the summary of geometric mean titer (GMT) with 95% CI. A total of 302 participants (n = 208 for aged 18–65 years; n = 94 for aged 65–85 years) received a booster 3rd-dose. The serum samples of 302 participants were collected at the indicted time points, Days 197 to 242 (the pre-booster day) and Days 211 to 256 (14 days post-booster), and tested for neutralizing antibody levels that inhibit 50% of live SARS-CoV-2 wild-type, expressed as VNT50 (WT, Wuhan strain) (functional), the inhibitory titers against S1-RBD binding to ACE2 by ELISA, expressed as μg/mL (functional), and anti-S1-RBD IgG antibody titers by ELISA (antigenic). Statistical analysis was performed by the Student’s t-test (ns, p>0.05; **** p<0.0001). (TIF) [file ppat.1010870.s003.tif]

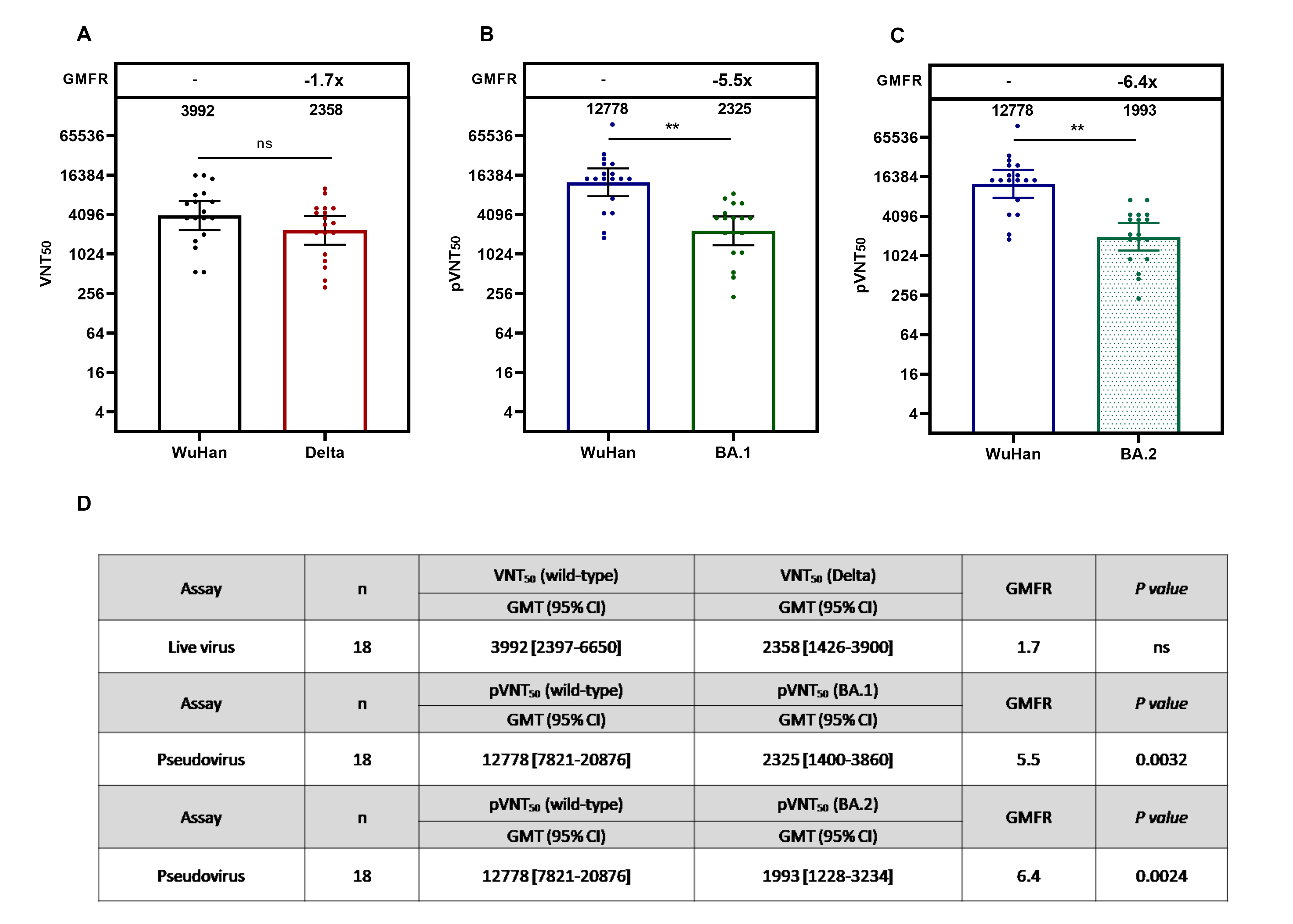

Supplement: S4 Fig — Geometric mean titers (GMT) at 50% viral-neutralization observed 14 days after the booster third-dose of 100-μg administered at mean Day 286 (Days 255–316) after the primary 2-dose series (Days 0 and 28) of the 196-day Phase-1 trial. (A) In the participants of the 100-μg group (n = 18) with healthy adults aged at 20–55 years, the post-booster VNT50 titer reached at 3,992 against live SARS-CoV-2 Wuhan wild-type, and at 2,358 against live Delta variant. (B) Similarly, unusually high post-booster pVNT50 against Wuhan wild-type pseudovirus at 12,778, and at 2,325 against Omicron BA.1. (C) High post-booster pVNT50 against Omicron BA.2 as well. (D) Summary of geometric mean titer (GMT) with 95% CI are presented for plots shown in panels A—C. *Fig 4A and 4B adapted with permission from J. Clin. Invest. 2022;132(10):e157707. https://doi.org/10.1172/JCI157707. (TIF) [file ppat.1010870.s004.tif]
