# Supplementary material for: Toward a pan-SARS-CoV-2 vaccine targeting conserved epitopes on spike and non-spike proteins for potent, broad and durable immune responses
Source: PLoS Pathog. 2023 Apr 20;19(4):e1010870. doi: 10.1371/journal.ppat.1010870 (PMC10153712; doi:10.1371/journal.ppat.1010870)
Supplement: S1 Table — (DOCX) [file ppat.1010870.s005.docx]

**Supporting Information**

**S1 Table. Comparison of post-booster viral-neutralizing antibody titers against SARS-CoV-2 wild-type (WT) and Delta variant by vaccines from different platforms*.**


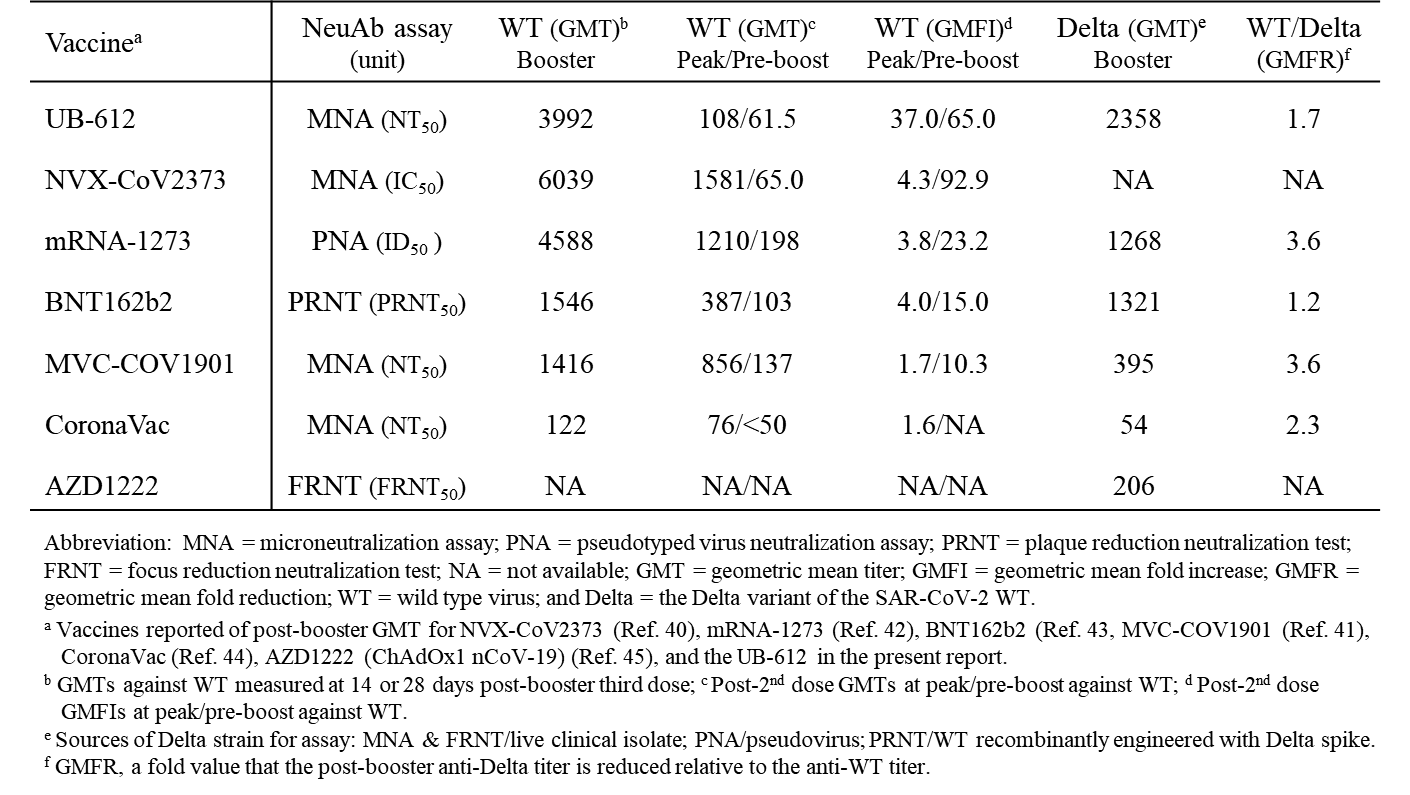


Abbreviation: MNA = Microneutralization assay; PNA = pseudotyped virus neutralization assay; PRNT = plaque reduction neutralization test; FRNT = focus reduction neutralization test; NA = not available; GMT = geometric mean titer; GMFI = geometric mean fold increase; GMFR = geometric mean fold reduction; WT = wild type virus; and Delta = the Delta variant of the SAR-CoV-2 WT.

^a^ Vaccine reported of post-booster GMT for NVX-CoV2373 (Ref. 40), mRNA-1273 (Ref. 42), BNT16b2 (Ref. 43), MVC-Cov1901 (Ref. 41), Corona Vac (Ref. 44), ADZ1222 (ChAdOx1 nCov-19) (Ref. 45), and UB-612 in the present report.

^b^ GMTs against WT measured at 14 or 28- days post-booster third dose.

^c^ Post-2^nd^ dose GMTs at peak/pre-booster against WT.

^d^ Post-2^nd^ dose GMFIs at peak/pre-booster against WT.

^e^ Sources of Delta strain for assay: MNA & FRNT/live clinical isolate: PNA/pseudovirus: PRNT/WT recombinantly engineered with Delta spike.

^f^ GMFR, a fold value that the post-booster anti-Delta titer is reduced relative to the anti-WT titer.

**^*^** Reprinted with permission from *J. Clin. Invest.* 2022;132(10):e157707. <https://doi.org/10.1172/JCI157707>.
