# Supplementary material for: Toward a pan-SARS-CoV-2 vaccine targeting conserved epitopes on spike and non-spike proteins for potent, broad and durable immune responses
Source: PLoS Pathog. 2023 Apr 20;19(4):e1010870. doi: 10.1371/journal.ppat.1010870 (PMC10153712; doi:10.1371/journal.ppat.1010870)
Supplement: S1 Methods — (DOCX) [file ppat.1010870.s006.docx]

**Supporting Methods**

**S1 Methods. Vaccine product and placebo.** UB-612 vaccine used in the present phase II extension booster vaccination is a multitope vaccine designed to activate both humoral and cellular responses. For SARS-CoV-2 immunogens, UB-612 combines a CHO-expressed S1-RBD-sFc fusion protein (Wuhan strain) and a mixture of synthetic T helper (Th) and cytotoxic T lymphocyte (CTL) epitope peptides, which are selected from immunodominant M, S2 and N regions known to bind to human major histocompatibility complexes (MHC) I and II. The preparation of UB-612 vaccine product consists of compounding, filtration, mixing, and filling operations. Before addition of the subunit protein S1-RBD-sFc, the individual components of the vaccine are filtered through a 0.22 micron membrane filter, including the peptide solution (2 µg/mL), CpG1, a proprietary oligonucleotide (ODN), solution (2 µg/mL), 10X protein buffer containing 40 mM Histidine, 500 mM Arginine and 0.6% Tween 80, 20% sodium chloride stock solution. After sequentially addition of each component, the S1-RBD-sFc fusion protein and peptides are formulated with components described as above to form a protein-peptide complex and then is adsorbed to aluminum phosphate (Adju-Phos) adjuvant. The last step would be addition of water for injection containing the 2-phenoxyethanol preservative solution to make final drug product at 200 µg/mL. The UB-612 vaccine product is stored at 2 to 8 ^o^C. Placebo used in the Phase-2 trial was sterile 0.9% normal saline.
