# Supplementary material for: Toward a pan-SARS-CoV-2 vaccine targeting conserved epitopes on spike and non-spike proteins for potent, broad and durable immune responses
Source: PLoS Pathog. 2023 Apr 20;19(4):e1010870. doi: 10.1371/journal.ppat.1010870 (PMC10153712; doi:10.1371/journal.ppat.1010870)
Supplement: S2 Methods — (DOCX) [file ppat.1010870.s007.docx]

**Supporting Methods**

**S2 Methods. Viral-neutralizing antibody titers against SARS-CoV-2 wild-type and variants by CPE based live virus neutralization assay.** Neutralizing antibody titers were measured by CPE-based live virus neutralization assay using Vero-E6 cells challenged with wild type (SARS-CoV-2-Taiwan-CDC#4, Wuhan) and Delta variant (SARS-CoV-2-Taiwan-CDC#1144, B.1.617.2), which was conducted in a BSL-3 lab at Academia Sinica, Taiwan. Vero-E6 (ATCC CRL-1586) cells were cultured in DMEM (Hyclone) supplemented with 10% fetal bovine serum (FBS, Gibco) and 1x Penicillin-Streptomycin solution (Thermo) in a humidified atmosphere with 5% CO_2_ at 37°C. The 96-well microtiter plates are seeded with 1.2 × 10^4^ cells/100 μL/well. Plates are incubated at 37°C in a CO_2_ incubator overnight. The next day tested sera were heated at 56°C for 30 min to inactivate complement, and then diluted in DMEM (supplemented with 2% FBS and 1x Penicillin/Streptomycin). Serial 2-fold dilutions of sera were carried out for the dilutions. 50 μL of diluted sera were mixed with an equal volume of virus (100 TCID_50_) and incubated at 37°C for 1 hr. After removing the overnight culture medium, 100 μL of the sera-virus mixtures were inoculated onto a confluent monolayer of Vero-E6 cells in 96-well plates in triplicate. After incubation for 4 days at 37°C with 5% CO_2_, the cells were fixed with 10% formaldehyde and stained with 0.5% crystal violet staining solution at room temperature for 20 min. Individual wells were scored for CPE as having a binary outcome of “infection” or “no infection”. Determination of SARS-CoV-2 virus specific neutralization titer was to measure the neutralizing antibody titer against SARS-CoV-2 virus based on the principle of VNT_50_ titer (≥50% reduction of virus-induced cytopathic effects). Virus neutralization titer of a serum was defined as the reciprocal of the highest serum dilution at which 50% reduction in cytopathic effects are observed and results are calculated by the method of Reed and Muench.
