# Supplementary material for: Toward a pan-SARS-CoV-2 vaccine targeting conserved epitopes on spike and non-spike proteins for potent, broad and durable immune responses
Source: PLoS Pathog. 2023 Apr 20;19(4):e1010870. doi: 10.1371/journal.ppat.1010870 (PMC10153712; doi:10.1371/journal.ppat.1010870)
Supplement: S3 Methods — (DOCX) [file ppat.1010870.s008.docx]

**Supporting Methods**

**S3 Methods. Neutralizing titers against Omicron BA.1/BA.2/BA.5 by pesudovirus assay.** Neutralizing antibody titers were measured by neutralization assay using HEK-293T-ACE2 cells challenged with SARS-CoV-2 pseudovirus variants. The study was conducted in a BSL2 lab at RNAi core, Biomedical Translation Research Center (BioTReC), Academia Sinica. Human embryonic kidney (HEK-293T/17; ATCC CRL-11268) cells were obtained from the American Type Culture Collection (ATCC). Cells were cultured in DMEM (Gibco) supplemented with 10% fetal bovine serum (Hyclone) and 100 U/mL of Penicillin-Streptomycin solution (Gibco), and then incubated in a humidified atmosphere with 5% CO2 at 37°C. HEK-293T-ACE2 cells were generated by transduction of VSV-G pseudotyped lentivirus carrying human ACE2 gene. To produce SARS-CoV-2 pseudoviruses, a plasmid expressing C-terminal truncated wild-type Wuhan-Hu-1 strain SARS-CoV-2 spike protein (pcDNA3.1-nCoV-SΔ18) was co-transfected into HEK-293T/17 cells with packaging and reporter plasmids (pCMVΔ8.91, and pLAS2w.FLuc.Ppuro, respectively) (BioTReC, Academia Sinica), using TransIT-LT1 transfection reagent (Mirus Bio). Site-directed mutagenesis was used to generate the Omicron BA.1, BA.2, and BA.4/BA.5 variants by changing nucleotides from Wuhan-Hu-1 reference strain. For BA.1 variant, the mutations of spike protein are A67V, Δ69-70, T95I, G142D/Δ143-145, Δ211/L212I, ins214EPE, G339D, S371L, S373P, S375F, K417N, N440K, G446S, S477N, T478K, E484A, Q493R, G496S, Q498R, N501Y, Y505H, T547K, D614G, H655Y, N679K, P681H, N764K, D796Y, N856K, Q954H, N969K, L981F. For BA.2 variant, the mutations of spike protein are T19I, L24S, Δ25-27, G142D, V213G, G339D, S371F, S373P, S375F, T376A, D405N, R408S, K417N, N440K, S477N, T478K, E484A, Q493R, Q498R, N501Y, Y505H, D614G, H655Y, N679K, P681H, N764K, D796Y, Q954H, N969K. For BA.4/5 variant, the mutations of spike protein are T19I, L24S, Δ25-27, Δ69-70, G142D, V213G, G339D, S371F, S373P, S375F, T,376A, D405N, R408S, K417N, N440K, L452R, S477N, T478K, E484A, L486V, Q493, Q498R, N501Y, Y505H, D614G, H655Y, N679K, N764K, D796Y, N856K, and Q954H, & L969K. Indicated plasmids were delivered into HEK-293T/17 cells by using TransITR-LT1 transfection reagent (Mirus Bio) to produce different SARS-CoV-2 pseudoviruses. At 72 hours post-transfection, cell debris were removed by centrifugation at 4,000 xg for 10 minutes, and supernatants were collected, filtered (0.45 μm, Pall Corporation) and frozen at −80 °C until use. HEK-293-hACE2 cells (1x10^4^ cells/well) were seeded in 96-well white isoplates and incubated for overnight. Tested sera were heated at 56°C for 30 min to inactivate complement, and diluted in medium (DMEM supplemented with 1% FBS and 100 U/ml Penicillin/Streptomycin), and then 2-fold serial dilutions were carried out for a total of 8 dilutions. The 25 μL diluted sera were mixed with an equal volume of pseudovirus (1,000 TU) and incubated at 37°C for 1 hr before adding to the plates with cells. After 1-hr incubation, the 50 μL mixture added to the plate with cells containing with 50 μL of DMEM culture medium per well at the indicated dilution factors. On the following 16 hours incubation, the culture medium was replaced with 50 μL of fresh medium (DMEM supplemented with 10% FBS and 100 U/ml Penicillin/Streptomycin). Cells were lysed at 72 hours post-infection and relative light units (RLU) was measured by using Bright-GloTM Luciferase Assay System (Promega). The luciferase activity was detected by Tecan i-control (Infinite 500). The percentage of inhibition was calculated as the ratio of RLU reduction in the presence of diluted serum to the RLU value of virus only control and the calculation formula was shown below: (RLU ^Control^ - RLU ^Serum^) / RLU ^Control^. The 50% protective titer (NT_50_ titer) was determined by Reed and Muench method.
