# Supplementary material for: Toward a pan-SARS-CoV-2 vaccine targeting conserved epitopes on spike and non-spike proteins for potent, broad and durable immune responses
Source: PLoS Pathog. 2023 Apr 20;19(4):e1010870. doi: 10.1371/journal.ppat.1010870 (PMC10153712; doi:10.1371/journal.ppat.1010870)
Supplement: S4 Methods — (DOCX) [file ppat.1010870.s009.docx]

**Supporting Methods**

**S4 Methods. Inhibition of RBDWT binding to ACE2 by ELISA.** The 96-well ELISA plates were coated with 2 µg/mL ACE2-ECD-Fc antigen (100 μL/well in coating buffer, 0.1M sodium carbonate, pH 9.6) and incubated overnight (16 to 18 hr) at 4°C. Plates were washed 6 times with Wash Buffer (25-fold solution of phosphate buffered saline, pH 7.0-7.4 with 0.05% Tween 20, 250 μL/well/wash) using an Automatic Microplate Washer. Extra binding sites were blocked by 200 μL/well of blocking solution (5 N HCl, Sucrose, Triton X-100, Casein, and Trizma Base). Five-fold dilutions of immune serum or a positive control (diluted in a buffered salt solution containing carrier proteins and preservatives) were mixed with a 1:100 dilution of RBDWT-HRP conjugate (horseradish peroxidase-conjugated recombinant protein S1-RBD-His), incubated for 30 ± 2 min at 25 ± 2°C, washed and TMB substrate (3,3’,5,5’-tetramethylbenzidine diluted in citrate buffer containing hydrogen peroxide) is added. Reaction is stopped by stop solution (diluted sulfuric acid, H_2_SO_4,_ solution, 1.0 M) and the absorbance of each well is read at 450 nm within 10 min using the Microplate reader (VersaMax). Calibration standards for quantitation ranged from 0.16 to 2.5 μg/mL. Samples with titer value below 0.16 μg/mL were defined as being half of the detection limit. Samples with titer exceed 2.5 μg/mL were further diluted for reanalysis.
