# Supplementary material for: Toward a pan-SARS-CoV-2 vaccine targeting conserved epitopes on spike and non-spike proteins for potent, broad and durable immune responses
Source: PLoS Pathog. 2023 Apr 20;19(4):e1010870. doi: 10.1371/journal.ppat.1010870 (PMC10153712; doi:10.1371/journal.ppat.1010870)
Supplement: S5 Methods — (DOCX) [file ppat.1010870.s010.docx]

**Supporting Methods**

**S5 Methods. Anti-S1-RBD_WT_ binding IgG antibody by ELISA.** The 96-well ELISA plates were coated with 2 µg/mL recombinant S1-RBD_WT_-His protein antigen (100 µL/well in coating buffer, 0.1 M sodium carbonate, pH 9.6) and incubated overnight (16 to 18 hr) at room temperature. One hundred μL/well of serially diluted serum samples (diluted from 1:20, 1:1,000, 1:10,000 and 1:100,000, total 4 dilutions) in 2 replicates were added and plates are incubated at 37°C for 1 hr. The plates were washed six times with 250 μL Wash Buffer (PBS-0.05% Tween 20, pH 7.4). Bound antibodies were detected with HRP-rProtein A/G at 37^o^C for 30 min, followed by six washes. Finally, 100 μL/well of TMB (3,3’,5,5’-tetramethylbenzidine) prepared in Substrate Working Solution (citrate buffer containing hydrogen peroxide) was added and incubated at 37^o^C for 15 min in the dark, and the reaction stopped by adding 100 μL/well of H_2_SO_4,_ 1.0 M. Sample color developed was measured on ELISA plate reader (Molecular Device, VersaMax). UBI EIA Titer Calculation Program was used to calculate the relative titer. The anti-S1-RBD antibody level is expressed as Log_10_ of an end point dilution for a test sample (SoftMax Pro 6.5, Quadratic fitting curve, Cut-off value 0.248).
