# Supplementary material for: Toward a pan-SARS-CoV-2 vaccine targeting conserved epitopes on spike and non-spike proteins for potent, broad and durable immune responses
Source: PLoS Pathog. 2023 Apr 20;19(4):e1010870. doi: 10.1371/journal.ppat.1010870 (PMC10153712; doi:10.1371/journal.ppat.1010870)
Supplement: S6 Methods — (DOCX) [file ppat.1010870.s011.docx]

**Supporting Methods**

**S6 Methods. T cell responses by ELISPOT.** Human peripheral blood mononuclear cells (PBMCs) were used in the detection of the T cell response. For the booster-series third-dose series extension study, ELISpot assays were performed using the human IFN-γ/IL-4 FluoroSpot^PLUS^ kit (MABTECH). Aliquots of 250,000 PBMCs were plated into each well and stimulated, respectively, with 10 μg/mL (each stimulator) of RBD-WT + Th/CTL, Th/CTL, or Th/CTL pool without UBITh1a (CoV2 peptides), and cultured in culture medium alone as negative controls for each plate for 24 hours at 37°C with 5% CO_2_. The analysis was conducted according to the manufacturer’s instructions. Spot-forming units (SFU) per million cells was calculated by subtracting the negative control wells.
