# Supplementary material for: Toward a pan-SARS-CoV-2 vaccine targeting conserved epitopes on spike and non-spike proteins for potent, broad and durable immune responses
Source: PLoS Pathog. 2023 Apr 20;19(4):e1010870. doi: 10.1371/journal.ppat.1010870 (PMC10153712; doi:10.1371/journal.ppat.1010870)
Supplement: S7 Methods — (DOCX) [file ppat.1010870.s012.docx]

**Supporting Methods**

**S7 Methods. Intracellular Cytokine Staining (ICS).** Intracellular cytokine staining and flow cytometry was used to evaluate CD4^+^ and CD8^+^ T cell responses. PBMCs were stimulated, respectively, with S1-RBD-His recombinant protein plus with Th/CTL peptide pool, Th/CTL peptide pool only, CoV2 peptides, PMA + Inonmycin (as positive controls), or cultured in culture medium alone as negative controls for 6 hours at 37°C with 5% CO_2_. Following stimulation, cells were washed and followed by surface stain for 30 minutes on ice, cell fixation and permeabilization with the BD cytofix/cytoperm kit (Catalog #554714) for 20 minutes on ice, and then intracellular stain for 30 minutes on ice. Intracellular cytokine staining of IFN-γ, IL-2 and IL-4 was used to evaluate CD4^+^ T cell response. Intracellular cytokine staining of IFN-γ, IL-2, CD107a and Granzyme B was used to evaluate CD8^+^ T cell responses. Note, CD107a was added prior to the stimulation step. Upon completion of staining, cells were analyzed in a FACSCanto II flow cytometry (BD Biosciences) using BD FACSDiva software.
