# Supplementary material for: Toward a pan-SARS-CoV-2 vaccine targeting conserved epitopes on spike and non-spike proteins for potent, broad and durable immune responses
Source: PLoS Pathog. 2023 Apr 20;19(4):e1010870. doi: 10.1371/journal.ppat.1010870 (PMC10153712; doi:10.1371/journal.ppat.1010870)
Supplement: S8 Methods — (DOCX) [file ppat.1010870.s013.docx]

**Supporting Methods**

**S8 Methods. Statistics.** For the Phase-2 extension booster vaccination study, the immunogenicity results for Geometric Mean Titer (GMT) are presented with the 95% confidence intervals and charts were prepared using GraphPad Prism software. Statistical analyses were performed using SAS Version 9.4 (SAS Institute, Cary, NC, USA) or Wilcoxon sign rank test. Spearman correlation was used to evaluate the monotonic relationship between non-normally distributed data sets. For the Phase-2 primary 2-dose series, the sample size of the trial design meets the minimum safety requirement of 3000 study participants in the vaccine group with healthy adults, as recommended by the US FDA and WHO. US Food and Drug Administration, Emergency use authorization for vaccines to prevent COVID-19: Guidance for industry, <https://downloads.regulations.gov/FDA-2020-D-1137-0019/attachment_1.pdf>; WHO Guidelines on clinical evaluation of vaccines: regulatory expectations, <https://cdn.who.int/media/docs/default-source/prequal/vaccines/who-trs-1004-web-annex-9.pdf?sfvrsn=9c8f4704_2&download=true>.
