# Supplementary material for: Toward a pan-SARS-CoV-2 vaccine targeting conserved epitopes on spike and non-spike proteins for potent, broad and durable immune responses
Source: PLoS Pathog. 2023 Apr 20;19(4):e1010870. doi: 10.1371/journal.ppat.1010870 (PMC10153712; doi:10.1371/journal.ppat.1010870)
Supplement: S1 Appendix — (PDF) [file ppat.1010870.s014.pdf]

S1 Supplemental Appendix

Phase-2 study V-205 protocol

[illegible]
06 Sep. 2021.

CLINICAL STUDY PROTOCOL

A Phase II, Placebo-controlled, Randomized, Observer-blind Study to Evaluate the Immunogenicity, Safety, and Tolerability of UB-612 Vaccine against COVID-19 in Adolescent, Younger and Elderly Adult Volunteers

**Protocol Number:**	V-205
**EudraCT Number:**	Not Applicable
**Investigational Product:**	UB-612
**Phase:**	II
**Sponsor:**	聯亞生技開發股份有限公司<br>United Biomedical, Inc., Asia (UBI Asia),<br>Hsinchu County, Taiwan
**Protocol Date:**	06 September, 2021
**Protocol Version:**	3.0

CONFIDENTIAL
This protocol may not be reproduced or communicated to a third party without the written permission of United Biomedical, Inc., Asia

1 PROTOCOL APPROVAL SIGNATURES

Protocol Title: A Phase II, Placebo-controlled, Randomized, Observer-blind Study to Evaluate the Immunogenicity, Safety, and Tolerability of UB-612 Vaccine against COVID-19 in Adolescent, Younger and Elderly Adult Volunteers

Protocol Number: V-205

This study will be conducted in compliance with the clinical study protocol (and amendments), International Council for Harmonisation (ICH) guidelines for current Good Clinical Practice (cGCP) and applicable regulatory requirements.

Sponsor Signatory

United Biomedical, Inc., Asia (UBI Asia),
No. 45, Guangfu N. Rd., Hukou Township,
Hsinchu County 303, Taiwan (R.O.C.)

Signature

Date

Reviewed and Approved by:

Sponsor's Representative

Thomas P. Monath, MD, FACP, FASTMH
Vaxxinity Inc.
1717 Main Street, Suite 3388
Dallas, TX 75201
Mobile: (978) 549-0708
Email: tom@vaxxinity.com

聯亞生技開發股份有限公司

06. Sep. 2021

2 SYNOPSIS

Protocol Number:

V-205

Title:

A Phase II, Placebo-controlled, Randomized, Observer-blind Study to Evaluate the Immunogenicity, Safety, and Tolerability of UB-612 Vaccine against COVID-19 in Adolescent, Younger and Elderly Adult Volunteers

Investigational Product:

UB-612 vaccine

Phase:

Phase II

Objectives:

Primary Objectives

- To evaluate the SARS-CoV-2 neutralizing antibody titer induced by UB-612 vaccine
- To evaluate the safety and tolerability of the UB-612 vaccine after vaccination

Secondary Objectives

- To evaluate the immune response to SARS-CoV-2 during the study
- To evaluate the lot consistency of immune responses induced by 3 independent batches of vaccine

Exploratory Objectives

- To evaluate the T cell function induced by UB-612
- To evaluate the safety and immunogenicity of the UB-612 vaccine in adolescents
- To evaluate the efficacy of UB-612 vaccine
- To describe the serological responses to the UB-612 vaccine in confirmed and/or severe COVID-19 cases
- **To evaluate antibody against SARS-CoV-2 antigens**

Study Design:

This is a phase II, observer-blind, multiple-centre, randomized, placebo-controlled study to evaluate the immunogenicity, safety, tolerability and lot consistency of 2 doses of UB-612 vaccine in adolescent, younger and elderly adults. Around 3850 adult subjects will be randomized to be composed of the core group for EUA application, while around 385 adolescents will be randomized to be the supplementary group for broader indication. All subjects will be randomly allocated to receive 2 doses of 100 µg vaccine or placebo in a 6:1 ratio, including 462 aged from >18 to < 65 years old, evaluable subjects in the lot-to-lot consistency group. As for immunogenicity, at least 350 evaluable young adults (aged >18 to < 65 years old) and 154 evaluable elderly (aged ≥ 65 years old) will be enrolled for descriptive analysis. Subjects in immunogenicity group should be enrolled first. All subjects will be included in the safety group, and it is intended that a minimum of 770 subjects will be randomized to be in the ≥ 65-year stratum. Adolescents will start to enrol after recruitment of the core group has been completed. 385 adolescents will be randomized to be allocated in 6:1 ratio, in which 154 evaluable adolescents will have immunogenicity data to compare with adults.

It will be consisted of 7 clinical visits and one long-term follow-up visit. All subjects will have blood test for safety before and after full vaccination. Subjects in lot-to-lot consistency and immunogenicity group will also have blood drawn for immune response, in which tests for T cell function will be optional. Subjects will come to the clinics at Visit 1 for screening, Visit 2 (Day 1, baseline) for randomization and 1st vaccination, Visit 3 (Day 29) for 2nd vaccination, Visit 4 (Day 57) for safety check and immunogenicity assessment, and Visit 5 (Day 197) for safety check and assessment of the persistence of immune response. **Subjects will also be unblinded at Visit 5, subjects in placebo group will withdraw from the study and subjects in vaccine group will be encouraged to have 3rd dose**

of vaccination (Day 197~Day 242) at Visit 6. Those who received 3rd dose will have Visit 7 (14 days after Visit 6) to check the booster effect. After Day 197, subjects will enter the long-term follow-up with a safety call bi-monthly. Subjects in lot-to-lot and immunogenicity group will be encouraged to visit site at Day 365 to check immune persistence. Subjects will be expected to participate for up to a maximum of approximately 13 months. Unblinding will be carried out after **subjects** in core group or supplementary group have completed Visit 5. For females of child-bearing potential, a urine pregnancy test will be performed before **each** vaccination and will be found negative. Female subjects or the female partners of male subjects who are pregnant during the study period will be followed the pregnancy outcomes.

Before any study-related procedures are performed, voluntary, written, study-specific informed consent will be obtained from the subjects. Subject will be screened at Visit 1. All subjects will have blood sampling for hematology, and biochemistry. After checking the eligibility of the subject at Visit 2, the randomization number and adjoining study intervention allocation will be assigned. **Subjects in lot-to-lot consistency and immunogenicity group will have blood drawn for immunogenicity before vaccination.** Unblinded site staff member(s) will dispense/administer 0.5 mL UB-612/placebo into the deltoid muscle of the preferably nondominant arm. Blinded site staff must observe the subject for at least 30 minutes after study vaccine administration for changes in vital signs or any acute anaphylactic reactions. The investigator will provide instructions for reactogenicity e-diary completion and ask the subject to complete the reactogenicity e-diary from Day 1 to Day 7, skin allergy reaction from Day 1 to Day 14, with Day 1 being the day of vaccination. Participants will be instructed to contact the site staff or investigator immediately if he or she experiences any of the Grade 3/4 AEs from Day 1 to Day 7 to determine if an unscheduled reactogenicity visit is required. Apart from reactogenicity, allergic skin rash should be monitored for 14 days post vaccination. **Once subject encountered the Grade 3/4 allergic skin rash, , site staff should call for detail to evaluate the necessarily of unscheduled visit. Investigators could ask for HLA typing survey if he/she concerned about the possibility of hypersensitivity. Of course, IDMC could also request HLA typing once they reviewed the safety data.**

At Visit 3 (Day 29), the 2nd study vaccine administration will be done and subjects will be closely monitored for 30 minutes after vaccination for changes in vital signs or acute anaphylactic reaction. The **7/14**-day post-vaccination e-diary will be given to subjects with a suitable instruction. For females of child-bearing potential, a urine pregnancy test will be performed before vaccination and found negative. At Visit 4 (Day 57, 1-month follow-up visit after 2nd vaccination), collect sample for safety and immunogenicity, review the participants' reactogenicity e-diary data and record any unsolicited AE.

All subjects will come back on Day 197, Visit 5, for immunogenicity persistence check in immunogenicity group and safety check for safety group. **Once subject completes Visit 5, he or she will be unblinded. Subjects in placebo group will withdraw from the study. Subjects who received UB-612 vaccine will be invited to join the extension study to determine the durability of the immune response and long-term safety after unblinding. Meanwhile, subjects in vaccine group will be encouraged to have a third dose of UB-612. All subjects who received UB-612 vaccine will be followed up for 12 months.**

All subjects will receive safety calls on Day 8, Day 15, Day 22, Day 36, Day 43, Day 64, Day 71, Day 78, Day 85, Day 253, and Day 309 that will serve both to monitor for unsolicited AEs or/and to monitor for symptoms of COVID-19. **After Day 57, each week (i.e., every 7 days) the subjects will receive a prompt on their smartphone device to regularly surveillance for signs and symptoms of COVID-19 and SAE. Subjects who received 3rd dose of UB-612 will receive an extra safety call on 7 days post 3rd vaccination.**

If a Grade 3 local reaction, systemic event, or fever are reported in the reactogenicity e-diary, a telephone contact should occur to ascertain further details and determine whether a site visit is clinically indicated. If suspected Grade 4 local reaction systemic event and /or fever is reported in the reactogenicity e-diary, a site visit should occur to confirm whether the event meets the criteria for Grade 4. All subjects were encouraged to contact site staff once they encountered Grade 3/4 AE. Allergic skin reaction should be monitored for 14 days post vaccination and should be contacted regardless of severity.

If a subject experiences a febrile illness associated with respiratory symptoms, he or she is instructed to contact the site immediately. Subjects may utilize a COVID-19 illness e-diary to prompt him/her to report any symptoms.

Number of Subjects:

At least 3850 adult subjects will be enrolled into core group, and 385 adolescents will be recruited for supplementary group. All subjects will randomly receive the 100 µg of UB-612 vaccine or placebo with allocation rate 6:1.

Treatment:

The UB-612 vaccines (200 μg/mL), 6.5 mL/vial, containing Adju-Phos® and CpG1 adjuvant, and the placebo, normal saline 0.9%, will be administered 0.5 ml by an intramuscular (IM) injection, 28 days apart (on Day 1 and 29)**, and a 3rd dose of UB-612 will be given after unblinding if subjects qualified.**

Duration of Study: 13 months

Study Population:

To be eligible for study entry, subjects must satisfy all of the following inclusion criteria:

1. Healthy male or non-pregnant female between the age of 12 to 85 years at time of enrolment.
2. Women of childbearing potential and men must agree to practice medically effective contraception from first vaccination until 3 months after the last vaccination. The acceptable effective contraception methods include:
    a. Male or female sterilization, implant, or intrauterine device;
    b. Injectable, pill, patch, ring plus one barrier method*;
    c. Two combined barrier methods*.

    *Effective barrier methods are diaphragm, male or female condoms, sponge, or spermicides (creams or gels that contain a chemical to kill sperm).
3. Able to understand the content and possible risks of informed consent and willing to sign the Informed Consent Form (ICF).
4. Able to understand and agrees to comply with all study procedures and be available for all study visits.
5. Ear temperature ≤ 38.0°C.
6. Healthy participants** who are determined by medical history, physical examination, and clinical judgment of the investigator to be eligible for inclusion in the study. In the investigator's clinical judgement, participant may have a stable and well-controlled comorbidity associated with an increased risk of progression to severe COVID-19.

    ** Healthy participants with preexisting stable disease, defined as disease not requiring significant change in therapy or hospitalization for worsening disease during the 12 weeks before enrolment and unlikely to require a significant change in therapy or hospitalization in the six months following enrollment, can be included.

Subjects will be excluded from the study if one or more of the following exclusion criteria is applicable:

1. History of anaphylaxis, urticarial, or other significant adverse reaction requiring medical intervention after receipt of a vaccine.
2. Female who is pregnant or positive in pregnancy test at screening or just prior to each vaccination administration.
3. Female who is breast-feeding or plans to breastfeed from the time of the first vaccination through 60 days after the last vaccination.
4. Any acute illness, as determined by the study investigator 3 days before first vaccination (these subjects can be re-scheduled).
5. Any major surgery one month before first vaccination (these subjects can be -rescheduled).
6. Known HIV antibody positive
7. Known active hepatitis B and hepatitis C disease. Active hepatitis means liver aminotransferase (AST and/or ALT) greater than 3xULN, and/or total bilirubin greater then 3xULN at screening.
8. Previous exposure to SARS-CoV-2 or receipt of an investigational or licensed product for the prevention of COVID-19, MERS or SARS.
9. Have history of Guillain-Barre syndrome.

10. Subjects who take part in another clinical study within 12 weeks prior to the day of informed consent.
11. Immune deficiency/disorder, whether due to genetic defect, immunodeficiency disease or immunosuppressive therapy
12. Subjects who plan to or are undergoing anti-cancer therapy
13. Platelet disorder or other bleeding disorder may cause injection contraindication.
14. Prior chronic administration (defined as ≥ 14 day of continuous use) of immunosuppressant or corticosteroids (equivalent to ≥ 20 mg daily of prednisone), cytotoxic treatment in last 6 months before first vaccination.
15. Prior administration of immunoglobulins and/or any blood products in last 4 months before first vaccination.
16. Receipt of any seasonal or pandemic influenza vaccine within 14 days, or any other nonstudy vaccine within 28 days, before study intervention administration.
17. Anticipated receipt of any seasonal or pandemic influenza vaccine within 14 days, or any other nonstudy vaccine within 28 days, after study intervention administration.
18. Receipt of short-term (<14 days) systemic corticosteroids. Study intervention administration should be delayed until systemic corticosteroid use has been discontinued for at least 28 days. Inhaled/nebulized, intra-articular, intrabursal, or topical (skin or eyes) corticosteroids are permitted.
19. Loss or donation of blood over 500 mL within 3 months prior to Screening Visit or intention to donate blood or blood products for transfusion during the study.
20. Any medical disease or condition that, in the opinion of the study investigator, may confound the results of the study or pose an additional risk to the subjects by their participation in the study.
21. Employees at the investigator's site, of the Sponsor or the contract research organization (CRO) **who** directly involved in the conduct of the study.

Primary Endpoint(s):

Immunogenicity Endpoint(s)

- Geometric mean titer (GMT) of SARS-CoV-2 neutralizing antibody on Day 57
- Seroconversion rate (SCR) of SARS-CoV-2 neutralizing antibody on Day 57

Safety Endpoint(s)

- Local reactions for up to 7 days following each dose
- Systemic events for up to 7 days following each dose
- Unsolicited AEs from Day 1 to Day 57
- MAAEs and SAEs from Day 1 to Day **365**
- AESIs and ADEs from Day 1 to Day **365**

Secondary Endpoint(s):

Immunogenicity Endpoint(s)

- Seroconversion rate (SCR) of antigen-specific antibody (Anti-S1-RBD) on Day 57
- Geometric mean titer (GMT) of SARS-CoV-2 neutralizing antibody on Day 197 and 365
- Geometric mean titer (GMT) of antigen-specific antibody (Anti-S1-RBD) on Day 57, 197 and 365
- Geometric mean fold increase in **SARS-CoV-2 neutralizing antibody and** antigen-specific antibody (Anti-S1-RBD) on Day 57, 197 and 365
- Lot consistency as assessed by the comparisons of the GMT of SARS-CoV-2 neutralizing antibody on Day 57 induced by 3 independent UB-612 vaccine clinical materials. The 95% confidence intervals between groups will be within the margin of 0.5 to 2.

Safety Endpoint(s)

- Changes of safety laboratory measures

Exploratory endpoint(s):

- T cell responses to UB-612 measured by ELISpot and flow cytometric assays on Day 57
- **T cell responses to UB-612 measured by ELISpot and flow cytometric assays on 14 days post 3rd dose of UB-612**
- **Geometric mean titer (GMT) of SARS-CoV-2 neutralizing antibody on 14 days post 3rd dose of UB-612**
- **Geometric mean titer (GMT) of antigen-specific antibody (Anti-S1-RBD) on 14 days post 3rd dose of UB-612**
- **Geometric mean fold increase in SARS-CoV-2 neutralizing antibody and antigen-specific antibody (Anti-S1-RBD) on 14 days post 3rd dose against pre-3rd dose baseline**
- **Geometric mean titer (GMT) of SARS-CoV-2 neutralizing antibody and antigen-specific antibody (Anti-S1-RBD) on Day 57, Day 197 and Day 365 in adolescents**
- **Seroconversion rate (SCR) of SARS-CoV-2 neutralizing antibody and antigen-specific antibody (Anti-S1-RBD) on Day 57 in adolescents**
- **Geometric mean fold increase in SARS-CoV-2 neutralizing antibody and antigen-specific antibody (Anti-S1-RBD) on Day 57, Day 197, and Day 365 in adolescents**
- Local reactions for up to 7 days following each dose in adolescents
- Systemic events for up to 7 days following each dose in adolescents
- Unsolicited AEs from Day 1 to Day 57 in adolescents
- MAAEs and SAEs from Day 1 to Day 365 in adolescents
- AESIs and ADEs from Day 1 to Day 365 in adolescents
- Changes of safety laboratory measures in adolescents
- COVID-19 incidence per 1000 person-years of follow-up based on PCR test
- To describe the anti-S1-RBD IgG levels and SARS-CoV-2 neutralizing titers to UB-612 in confirmed and/or severe COVID-19 cases
- **To detect antibody against SARS-CoV-2 antigens derived from S2, N, and M protein**

Sample Size Determination:

Consider a 10% drop-out rate, around 3850 adult subjects in core group will be recruited and randomized with 6:1 allocation rate (100 µg and placebo) and around 385 adolescents will be recruited for supplementary group and randomized with same allocation ratio.

Sample Size for Safety Evaluation

For safety outcomes, the following table shows the probability of observing at least 1 SAE for a given true event rate of a particular SAE. For example, if the true SAE rate is 0.01%, with 2400 young adult subjects, 600 elderly adult subjects, and 300 adolescent subjects received UB-612 vaccine, there are 21.3%, 5.8%, and 3.0% probabilities of observing at least 1 SAE. Overall, probability of observing at least 1 SAE is 25.9% with true SAE rate of 0.01% for all 3000 adult subjects received study vaccine in core group.

True SAE Rate	N=300	N=600	N=2400	N=3000
0.001%	0.003	0.006	0.024	0.030
0.010%	0.030	0.058	0.213	0.259

0.100%	0.259	0.451	0.909	0.950

Sample Size for Lot-to-Lot Consistency

Younger adults are selected for this analysis since they should have immune responses less affected by immune senescence, which tends to result in more variable responses. The sample size is driven by the objective to demonstrate the consistency of GMT ratio (GMTR) with the 3 consecutive manufacturing lots of the UB-612 vaccine. The clinical lot-to-lot consistency will be tested for the three pair-wise comparisons by computing the two-sided 95% CI on the GMTR. If all confidence intervals are within the pre-defined clinical limits of [0.5, 2.0], one can conclude that the lots are consistent.

Assume the expected GMTR of 1 and a value of 0.6 for the standard deviation (SD) of the decimal logarithmic transformation (log base 10) of antibody titers (log10(GSD) is about 0.2 to 0.6; refer to following table of the V-122 interim result). In order to have at least 90% power to achieve the lot-to-lot consistency, the estimated sample size should ensure beta less than 3.3% (Bonferroni adjustment of beta for 3 comparisons between 3 lots). With the parameters above it estimates a minimum evaluable sample size per lot of 115 subjects. Consider a 10% drop-out rate, a total of 396 subjects (132 subjects per lot) will be needed for ensuring overall lot-to-lot consistency with at least 90% overall power.

**Antibody Titers**	**UB-612 10 µg**		**UB-612 30 µg**		**UB-612 100 µg**	
	GSD	log10(GSD)	GSD	log10(GSD)	GSD	log10(GSD)
Neutralizing	3.286	0.52	2.438	0.39	1.670	0.223
Anti-S1-RBD	4.185	0.62	3.112	0.49	1.436	0.16

Statistical Analysis:

Enrolled Population: Enrolled Population includes subjects who have a signed ICF.

Randomized Population: Subjects who are assigned a random number be regarded as Randomized Population.

Evaluable Immunogenicity Population: Evaluable Immunogenicity Population will consist of all eligible randomized subjects who are assigned to **lot-to-lot and** immunogenicity group, receive two vaccinations within the predefined window, have a valid immunogenicity result at visit 4 (Day 57), have no major protocol deviations or protocol deviations having impact on immunogenicity data. Evaluable Immunogenicity Population will be regarded as primary population for immunogenicity evaluation on primary and secondary immunogenicity endpoints except lot-to-lot consistency.

Evaluable Lot-to-Lot Population: A subset of Evaluable Immunogenicity but only includes subjects who are assigned to lot-to-lot consistency group and have immunogenicity determination at Day 57. Evaluable Lot-to-Lot Population will be used for evaluating lot-to-lot consistency only.

Evaluable Efficacy Population: **All eligible randomized subjects who receive two vaccinations within the predefined window and have no major protocol deviations or protocol deviations having impact on immunogenicity data will be the Evaluable Efficacy Population.** Evaluable Efficacy Population will be used to evaluate vaccine efficacy in exploratory analysis.

Available Immunogenicity Population: Available Immunogenicity Population will consist of all eligible randomized subjects who receive at least one vaccination, and have at least one post immunogenicity data determination. Available Immunogenicity Population will also be used to the immunogenicity evaluation except lot-to-lot consistency.

Evaluable Booster Population: All eligible randomized subjects who receive a third dose of UB-612 within the predefined window and have no major protocol deviations or protocol deviations having impact on immunogenicity data will be the Evaluable Booster Population. Evaluable Booster Population will be used to explore the immunogenicity evaluation after the booster vaccination.

Safety Population: The Safety Population (SAF) will consist of all subjects who received at least one vaccination. The Safety Set is for safety evaluation in analysis.

All safety assessments, including AEs, physical examinations (PEs), vital signs (VS), and clinical laboratory evaluations, where indicated, will be presented using descriptive statistics for each study group.

Local reactions for up to 7 days following each dose and systemic events for up to 7 days following each dose will be summarized with counts and percentages by study groups. Unsolicited AEs will be presented in counts and percentage with system organ class and preferred term by study groups. The number and percentage of MAAEs, SAEs, AESIs and ADEs will be displayed in summary table by study groups. Changes of safety laboratory measures will be summarized with descriptive statistics by study group and each time point.

The immunogenicity will be evaluated descriptively by seroconversion rate (SCR), GMT, GMFI and the associated 95% confidence intervals (CIs). **Immunogenicity evaluation for subjects received two vaccinations and three vaccinations will be summarized separately. Analysis of immunogenicity for subjects received a third dose of UB-612 will be performed with the Evaluable Booster Population only.**

For lot consistency, all pairs of lots, the two sided 95% CIs for the GMT ratios of SARS-COV-2 neutralizing titers will be calculated. If the two-sided 95% CIs for the GMTR of SARS-CoV-2 neutralizing titers are within the [0.5, 2.0] clinical limit interval, lot consistency will be concluded.

Interim Analysis:

First interim analysis and report will be performed when all adult immunogenicity data (350 evaluable young adults and 154 evaluable elderly subjects) for Day 57 are available

Second interim analysis and report, as EUA application dossier, will be performed when at least half of core group subjects (at least 3500 evaluable subjects) will be completed Day 85 safety follow up.

Third interim analysis and report, as EUA application dossier, will be performed when all immunogenicity data for lot-to lot consistency of Day 57 are available

Fourth interim analysis and report, as supplementary dossier for EUA, will be performed when 350 evaluable adolescents completed Day 57 safety follow up.

Fifth interim analysis will be performed for immunogenicity and safety data for young and elderly adult subjects receiving 3rd vaccination completed Visit 7, which is 14 days after 3rd vaccination.

Sixth interim analysis will be performed for immunogenicity and safety data for adolescent subjects receiving 3rd vaccination completed Visit 7, which is 14 days after 3rd vaccination.

Planned IDMC Meeting:

1st time: when all adult immunogenicity data (350 evaluable young adults and 154 evaluable elderly subjects) for Day 57 are available

2nd time: when at least half of core group subjects (at least 3500 evaluable subjects) will be completed Day 85 safety follow up. Adolescence could be enrolled after 2nd IDMC meeting.

3rd time: when all immunogenicity data for lot-to lot consistency of Day 57 are available.

4th time: when 350 evaluable adolescents will be completed Day 57 safety follow up.

The meeting schedule might be adjusted based on the progress of recruitment. The independent data monitoring committee (IDMC), consisting of at least two physicians and one statistician, will review the interim analysis results or reports.

Disease-Related Events and/or Disease-Related Outcomes Not Qualifying as AEs or SAEs:

Potential COVID-19 illnesses and their sequelae that are consistent with the disease definition should not be recorded as AEs. These data will be captured as efficacy assessment data only on the relevant pages of the CRF, as these will be expected exploratory endpoints.

Schedule of Assessments

Lot-to-lot consistency & Immunogenicity group

Scheduled visit	***1[m]***	***2[m]***		***3***		***4***		***5/ET***	***6[t]***		***7***		***Long-term follow-up***
Test & observations	***Screening***	***1st vaccination***		***2nd vaccination***		***Follow-up***		***Unblinding***	***3rd vaccination[h]***		***Follow-up[h]***		***Month 12 follow-up[i]***
Day	***-28~-1***	*1*	*8, 15, 22*	*29 ±3 days*	*36, 43*	*57 ±3 days*	*64, 71, 78, 85*	*197 ±15 days*	***197~242***	***7 days after Visit 6***	***14 days after Visit 6 ±3 days***	*253, 309*	*365 ±45 days*
Informed consent	X								**X[h]**				
Inclusion/Exclusion Criteria	X	X											
Randomization		X											
**Contraindication to vaccination**				X					**X[h]**				
Demographics	X												
Medical history	X	X											
Physical Exam [a]	X	X		X		X		X	**X[h]**		**X[h]**		
Vital sign	X	X		X		X		X	**X[h]**		**X[h]**		X
ECG	X												
**Lab (Safety)**													
Blood routine [j]	X					X			**X[h]**		**X[h]**		
Biochemistry [j]	X					X			**X[h]**		**X[h]**		
Immunology [j]	X					X			**X[h]**		**X[h]**		
Pregnancy [b]		X		X					**X[h]**				
Urinalysis [b]	X	X[q]		X[q]		X[q]		X[q]	**X[q, h]**		**X[q, h]**		
**Lab (Immunogenicity)**													
Immune response assessment [c]		X		X[n]		X		X					X

Scheduled visit	***1[m]***	***2[m]***		***3***		***4***		***5/ET***	***6[t]***		***7***		***Long-term follow-up***
Test & observations	***Screening***	***1st vaccination***		***2nd vaccination***		***Follow-up***		***Unblinding***	***3rd vaccination[h]***		***Follow-up[h]***		***Month 12 follow-up[i]***
Day	***-28~-1***	*1*	*8, 15, 22*	*29 ±3 days*	*36, 43*	*57 ±3 days*	*64, 71, 78, 85*	*197 ±15 days*	***197~242***	***7 days after Visit 6***	***14 days after Visit 6 ±3 days***	*253, 309*	*365 ±45 days*
T cell function (optional [o])		X				X							
**Lab (Exploratory)**													
**T cell function (optional [u])**									**X [h]**		**X [h]**		
**Spared sample [r]**	**X**					**X**							
**Immune response[s]**									**X [h]**		**X[h]**		
Vaccination		X		X					**X [h]**				
e-Diary instruction		X		X					**X [h]**				
Safety calls [d]			X		X		X			**X[s ,h]**		X	
AEs/AESIs [p] /MAAEs/SAEs		X [k]	X [k]	X [k]	X [k]	X [k]	X [k]	X [l]	**X [l, h]**	**X [l, h]**	**X [l, h]**	X [l]	X [l]
COVID-19 infection surveillance		X	X	X	X	X [e]	X [e]	X [e]	**X [e, h]**	**X [e, h]**	**X [e, h]**	X [e]	X [e]
Concomitant Medication		X		X		X		X [f]	**X [f, h]**		**X [f, h]**		

a: Body weight and height will be assessed at Visit 1 only.
b: Screening for pregnancy will be performed by urine sample test on Day 1, Day 29. When positive urine pregnancy test is presented, it should be confirmed by a serum test. Serum testing in lieu of urine tests will not be considered a protocol deviation. Proteinuria will be checked via urine routine as baseline.
c: For anti-S1-RBD IgG levels, SARS-CoV-2 neutralizing titers, and antibody titers which inhibit S1-RBD: ACE2 binding.
d: All subjects will receive safety calls that will serve both to monitor for unsolicited AEs, including AESIs, and to monitor for symptoms of COVID-19.
e: Each week (i.e., every 7 days) the subject will receive a prompt on their smartphone device to regularly surveillance for signs and symptoms of COVID-19. Record medication for potential COVID-19 on COVID-19 page.
f: Only record medications for MAAE and SAE.
g: 28 days after 2nd vaccination
h: Only for subjects in vaccine group and have agreed to receive 3rd dose of UB-612.
i: 12 months (336 days) after 2nd vaccination

j: Safety laboratory includes complete blood count (Hgb, Hct, RBC), WBC with differential, platelet count, creatinine, ALT, AST, total and direct bilirubin, hs-CRP, and ANA.
k: Active collection period
l: Passive surveillance period
m: Visit 1 and 2 could be emerged as one visit.
n: Anti-S1-RBD IgG level only
o: At least 100 aged >18-< 65 subjects in selected sites will be invited
p: To include PIMMC (listed in **Table 9-6**), or any newly identified potential AESI followed through 12 months after participants' final vaccination. Complications of COVID-19, also termed as ADE, (listed in **Table 9-5**) should be considered and reported as AESIs.
q: If subject encountered ≥ Grade 3 hypertension, the existence or deterioration of proteinuria will be checked.
r: An aliquot of spared serum is to be frozen and stored for UBI SARS-CoV-2 ELISA, Confirmatory SARS-CoV-2 ELISA, and future immunologic tests.
s: Subjects who qualified to receive 3rd dose will enter 2 weeks e-diary for follow-up and a safety call post 7 days post vaccination.
t: Visit 5 and Visit 6 could be the same day.
u: Approximately thirty aged >18-< 65 subjects and approximately thirty aged ≥ 65 years subjects in selected sites will be invited. When applicable, subjects who were assessed for T cell functions on Day 57 are preferred to be invited.
ADE (Antibody Dependent Enhancement), AESIs (Adverse Event of Special Interests), ANA (Anti-Nuclear Antibody), ET (Early Termination), MAAEs (Medically Attend Adverse Events)

Safety check group

Scheduled visit	***1*** [l]	***2*** [l]		***3***		***4***		***5/ET***	***6*** [q]		***7***		***Long-term follow-up***
Test & observations	***Screening***	***1st vaccination***		***2nd vaccination***		***Follow-up*** [f]		***Unblinding***	***3rd vaccination*** [g]		***Follow-up*** [g]		***Month 12 follow-up*** [h]
Day	***-28~-1***	*1*	*8, 15, 22*	*29 ±3 days*	*36, 43*	*57 ±3 days*	*64, 71, 78, 85*	*197 ±15 days*	***197~242***	***7 days after Visit 6***	***14 days after Visit 6 ±3 days***	*253, 309*	*365 ±45 days*
Informed consent	X								**X** [g]				
Inclusion/Exclusion Criteria	X	X											
Randomization		X											
**Contraindication to vaccination**				X					**X** [g]				
Demographics	X												
Medical history	X	X											
Physical Exam [a]	X	X		X		X		X	**X** [g]		**X** [g]		
Vital sign	X	X		X		X		X	**X** [g]		**X** [g]		X
ECG	X												
**Lab (Safety)**													
Blood routine [i]	X					X			**X** [g]		**X** [g]		
Biochemistry [i]	X					X			**X** [g]		**X** [g]		
Immunology [i]	X					X			**X** [g]		**X** [g]		
Pregnancy [b]		X		X					**X** [g]				
Urinalysis [b]	X	X [n]		X [n]		X [n]		X [n]	**X** [g, n]		**X** [g, n]		
**Lab (Exploratory)**													

Scheduled visit	***1*** [*l*]	***2*** [*l*]		***3***		***4***		***5/ET***	***6***[*q*]		***7***		***Long-term follow-up***
Test & observations	***Screening***	***1st vaccination***		***2nd vaccination***		***Follow-up***[*f*]		***Unblinding***	***3rd vaccination*** [*g*]		***Follow-up***[*g*]		***Month 12 follow-up*** [*h*]
Day	***-28~-1***	*1*	*8, 15, 22*	*29 ±3 days*	*36, 43*	*57 ±3 days*	*64, 71, 78, 85*	*197 ±15 days*	***197~242***	***7 days after Visit 6***	***14 days after Visit 6 ±3 days***	*253, 309*	*365 ±45 days*
**Spared sample** [o]	**X**					**X**							
**Immune response** [g]									**X** [g]		**X** [g]		**X** [g]
Vaccination		X		X					**X** [g]				
e-Diary instruction		X		X					**X** [g]				
Safety calls [c]			X		X		X			**X**[p, g]		X	
AEs/AESIs[m]/MAAEs/SAEs		X [j]	X [j]	X [j]	X [j]	X [j]	X [j]	X [k]	**X** [k, g]	**X** [k, g]	**X** [k, g]	**X** [k]	X [k]
COVID-19 infection surveillance		X	X	X	X	X [d]	X [d]	X [d]	**X** [d, g]	**X** [d, g]	**X** [d, g]	**X** [d]	X[d]
Concomitant Medication		X		X		X		X [e]	**X** [e, g]		**X** [e, g]		

a: Body weight and height will be assessed at Visit 1 only.
b: Screening for pregnancy and proteinuria will be performed by urine sample on Day 1, Day 29 . When positive urine pregnancy test is presented, it should be confirmed by a serum test. Serum testing in lieu of urine tests will not be considered a protocol deviation. Proteinuria will be checked via urine routine as baseline.
c: All subjects will receive safety calls that will serve both to monitor for unsolicited AEs, including AESIs, and to monitor for symptoms of COVID-19.
d: Each week (i.e., every 7 days) the subject will receive a prompt on their smartphone device to regularly surveillance for signs and symptoms of COVID-19. Record medication for potential COVID-19 on COVID-19 page.
e: Only record medications for MAAE and SAE.
f: 28 days after 2nd vaccination
g: Only for subjects in vaccine group and have agreed to receive 3rd dose of UB-612.
h: 12 months (336 days) after 2nd vaccination
i: Safety laboratory includes complete blood count (Hgb, Hct, RBC), WBC with differential, platelet count, creatinine, ALT, AST, total and direct bilirubin, hs-CRP and ANA.
j: Active collection period
k: Passive surveillance period
l: Visit 1 and 2 could be emerged as one visit.

m: To include PIMMC (listed in **Table 9-6**), or any newly identified potential AESI followed through 12 months after participants' final vaccination. Complications of COVID-19, also termed as ADE, (listed in **Table 9-5**) should be considered and reported as AESIs.

n: If subject encountered ≥ Grade 3 hypertension, the existence or deterioration of proteinuria will be checked .

o: An aliquot of spared serum is to be frozen and stored for UBI SARS-CoV-2 ELISA, Confirmatory SARS-CoV-2 ELISA, anti-S1-RBD IgG ELISA, S1-RBD:ACE2 binding inhibition ELISA, and future immunologic tests.

p: Subjects who qualified to receive 3rd dose will enter 2 weeks e dairy for follow-up and a safety call post 7 days post vaccination.

q: Visit 5 and Visit 6 could be the same day.

ADE (Antibody Dependent Enhancement), AESIs (Adverse Event of Special Interests), ANA (Anti-Nuclear Antibody), ET (Early Termination), MAAEs (Medically Attend Adverse Events)

Blood collection at scheduled visits
Lot-to-lot consistency & Immunogenicity group

Scheduled visit	***1***	***2***		***3***		***4***			***5/ET***	***6***		***7***		***Long-term follow-up***
Test & observations	***Screening***	***1st vaccination***		***2nd vaccination***		***Follow-up***		***Unblinding***	***3rd vaccination [a]***		***Follow-up [a]***		***Month 12 follow-up***
Day	***-28~-1***	*1*	*8, 15, 22*	*29 ±3 days*	*36, 43*	*57 ±3 days*	*64, 71, 78, 85*	*197 ±15 days*	***197~242***	***7 days after Visit 6***	***14 days after Visit 6 ±3 days***	*253, 309*	*365 ±45 days*
**Lab (Safety)**													
Blood routine	4.5					4.5			**4.5**		**4.5**		
Biochemistry	**3**					**3**			**3**		**3**		
Immunology	3					3			**3**		**3**		
**Lab (Immunogenicity)**													
Immune response assessment		20		5		20		20					20
**Lab (Exploratory)**													
**Spared sample**	**5**					**5**							
**Immune response***									**10[b]**		**10**		
Total blood collection	**15.5**	20		5		**35.5**		20	**20.5**		**20.5**		20

Total amount of blood collection: **157 mL.**

* An additional 56 mL of blood sample will be collected for the optional T cell assessments at **Visit 2, Visit 4, Visit 6 and Visit 7.**

a: Only for subjects in vaccine group and have agreed to receive 3rd dose of UB-612. An additional 10 mL of blood sample will be collected at Visit 6 and 14 days after Visit 6.

b: If Visit 5 and Visit 6 are the same visit, the 10mL blood sample for exploratory immune response will be not collected.

Safety check group

Scheduled visit	***1***	***2***		***3***		***4***		***5/ET***	***6***		***7***		***Long-term follow-up***
Test & observations	***Screening***	***1st vaccination***		***2nd vaccination***		***Follow-up***		***Unblinding***	***3rd vaccination[a]***		***Follow-up[a]***		***Month 12 follow-up[a]***
Day	***-28~-1***	*1*	*8, 15, 22*	*29 ±3 days*	*36, 43*	*57 ±3 days*	*64, 71, 78, 85*	*197 ±15 days*	***197~242***	***7 days after Visit 6***	***14 days after Visit 6 ±3 days***	*253, 309*	*365 ±45 days*
**Lab (Safety)**													
Blood routine	4.5					4.5			**4.5**		**4.5**		
Biochemistry	**3**					**3**			**3**		**3**		
Immunology	3					3			**3**		**3**		
**Lab (Exploratory)**													
**Spared sample**	**5**					**5**							
**Immune response**									**10**		**10**		**10**
Total blood collection	**15.5**					**15.5**			**20.5**		**20.5**		**10**

Total amount of blood collection: **82 mL.**

a: Only for subjects in vaccine group and have agreed to receive 3rd dose of UB-612. An additional 10 mL of blood sample will be collected at Visit 6, 14 days after Visit 6, and Day 365.

3 TABLE OF CONTENTS

1 Protocol Approval Signatures ... 2

2 Synopsis ... 3

3 Table of Contents ... 18

4 List of Abbreviations ... 22

5 Introduction ... 24

5.1 Study Rationale ... 24

5.2 Background ... 24

5.3 UB-612 COVID-19 Vaccine ... 25

5.4 Phase I study summary (V-122 and V-123) ... 25

5.5 Risk/Benefit Assessment ... 27

6 Study Objectives & Endpoints ... 29

6.1 Primary Objective(s) ... 29

6.2 Primary Endpoint(s) ... 29

6.3 Secondary Objective(s) ... 29

6.4 Secondary Endpoint(s) ... 29

6.5 Exploratory Objective(s) ... 29

6.6 Exploratory endpoint(s) ... 30

7 Investigational Plan ... 31

7.1 Overall Study Design and Plan: Description ... 31

7.1.1 End of Study Definition ... 32

7.2 Schedule of Assessments ... 33

7.3 Discussion of Study Design ... 42

7.4 Selection of Study Population ... 42

7.4.1 Number of Planned Subjects ... 42
7.4.2 Inclusion Criteria ... 43
7.4.3 Exclusion Criteria ... 43
7.4.4 Contraindications to Vaccination ... 45
7.4.4.1 Criteria for Delay of Study Treatment ... 45
7.4.4.2 Criteria for Discontinuation of Study Treatment ... 45
7.4.5 Screen Failures ... 45
7.4.6 Method of Assigning Subjects to Treatment Groups ... 46

7.4.7 Blinding 46
7.4.8 Removal of Subjects 47
7.4.8.1 Removal of Subjects from Immunogenicity Analysis 47
7.4.8.2 Removal of Subjects from the Study 47
7.4.8.3 Study Termination 47
7.4.8.4 Reporting and Follow-up of Pregnancies 48
7.4.8.5 SARS-CoV-2 infection 48

7.5 Investigational Products 48

7.5.1 Identity of Investigational Products 48
7.5.2 Investigational Products Administered 49
7.5.2.1 Injection Volume(s) 49
7.5.2.2 Administration 49
7.5.2.3 Emergency Event Management 49
7.5.3 Packaging and Labeling 49
7.5.4 Prior and Concomitant Therapy 49
7.5.4.1 Prohibited Medication/Therapy 50

8 Timing of Study Procedures 51

8.1 Study Procedures 51

8.1.1 Visit 1 (Day -28 ± 3 days) –Screening 51
8.1.2 Visit 2 (Day 1) –Baseline: 1st Vaccination 51
8.1.3 Day 8, 15, and 22 –Safety calls 52
8.1.4 Visit 3 (Day 29 ± 3 day) –2nd Vaccination 52
8.1.5 Day 36 and 43 –Safety calls 53
8.1.6 Visit 4 (Day 57 ± 3 day) –Follow-up 53
8.1.7 Day 64, Day 71, Day 78, and Day 85 –Safety calls 53
8.1.8 Visit 5 (Day 197 ± 15 days) –Unblinding / Early Termination 53
8.1.9 Visit 6 (Day 197~242) –3rd Vaccination 54
8.1.10 Safety call on 7 days after Visit 6 (Only for subjects who have 3rd vaccination) 55
8.1.11 Follow up on 14 days after Visit 6 (± 3 day) (Only for subjects who have 3rd vaccination) 55
8.1.12 Day 253 and Day 309- Bi-monthly Safety calls 55
8.1.13 Long-term follow-up (Day 365 ± 45 days) 55

8.2 Early Termination (ET) 56

8.3 Unscheduled Visit 56

8.4 COVID-19 Surveillance (All Subjects) 56

8.4.1 Taiwan CDC Criteria for Case Definition of COVID-19 56
8.4.2 Intercurrent COVID-19 57
8.4.3 Potential COVID-19 Infection 59
8.4.4 COVID-19 Convalescent Visit 59

8.5 Duration of Treatment 59

9 Immunogenicity and Safety Variables 60

9.1 Informed Consent Form 60

9.2 Demographics / Other Baseline Characteristics 60

9.3 Eligibility 60

9.4 Administration 60

9.5 Safety and Immunogenicity Measurements Assessed 60

9.5.1 Safety Variables 60
9.5.1.1 Physical Examination 60
9.5.1.2 Vital Sign 61
9.5.2 Electrocardiogram(ECG) 61
9.5.3 Clinical Laboratory Evaluation 61
9.5.4 Pregnancy Test 62
9.5.5 Self-Evaluation/Reporting (Solicited/Unsolicited Symptoms) 62
9.5.5.1 Solicited Symptoms 62
9.5.5.2 Unsolicited Adverse Event 64
9.5.5.3 Time Period and Frequency for Collecting AE and SAE Information 64
9.5.6 Adverse events (AEs) 65
9.5.6.1 Definition of Adverse Events (AE) 65
9.5.6.2 Definition of Serious Adverse Events (SAE) 65
9.5.6.3 Definition of Unexpected Adverse Reaction Definition 66
9.5.6.4 Definition of Medically Attended Adverse Event 66
9.5.6.5 Definition of Adverse Events of Special Interest (AESI) 67
9.5.6.6 Assessment of Severity 69
9.5.6.7 The Relationship to Study Vaccine 69
9.5.6.8 Adverse Events Reporting 71
9.5.7 Immunogenicity Assessments 72
9.5.7.1 Surveillance and Laboratory Diagnosis of SARS-CoV-2 Infection during Clinical Trial 72
9.5.7.2 Detection of ELISA Antibodies against S1-RBD of SARS-CoV-2 73
9.5.7.3 Detection of Antibody Titers Which Inhibit S1-RBD:ACE2 binding 73
9.5.7.4 Neutralizing Antibody Titers against SARS-CoV-2 73
9.5.7.5 Detection of antibody against SARS-CoV-2 antigens 73
9.5.7.6 Detection of T Cell Response 73
9.5.8 Communication and Use of Technology 74
9.5.9 Appropriateness of Measurements 74

9.6 Independent Data Monitoring Committee (IDMC) 74

9.6.1 Overall Study Stopping Rules 75

10 Statistical Methods 77

10.1 Statistical and Analytical Plans 77

10.2 Sample Size Determination 77

10.2.1 Sample Size for Safety Evaluation 77
10.2.2 Sample Size for Lot-to-Lot Consistency 77

10.3 Analysis Populations 78

10.4 Demographic and Other Baseline Characteristics 79

10.5 Safety Evaluation 79

10.5.1 Analysis of Primary Safety Endpoint(s) 80
10.5.2 Analysis of Secondary Safety Endpoint 80

10.6 Immunogenicity Evaluation 81

10.6.1 Analysis of Primary Immunogenicity Endpoint(s) 81
10.6.2 Analysis of Secondary Immunogenicity Endpoint(s) 81

10.7 Analysis of Exploratory Endpoint(s) 82

10.8 Subgroup Analysis 85

10.9 Interim Analysis 85

10.10 Handling of Missing Data 86

10.11 Protocol Deviations 86

11 Quality Assurance and Quality Control 87

11.1 Audit and Inspection 87

11.2 Monitoring 87

11.3 Data Management and Coding 87

12 Records and Supplies 88

12.1 Drug Accountability 88

12.2 Financing and Insurance 88

13 Ethics 89

13.1 Independent Ethics Committee or Institutional Review Board 89

13.2 Regulatory Authorities 89

13.3 Ethical Conduct of the Study 89

13.4 Informed Consent 89

13.5 Subject Confidentiality 90

14 Reporting and Publication, Including Archiving 91

15 References 92

4 LIST OF ABBREVIATIONS

ACE2	Angiotensin-converting enzyme 2
ADE	Antibody dependent enhancement (of viral replication)
AE	adverse event
AESI	adverse event of special interest
AEFI	adverse events following immunization
ALP	alkaline phosphatase
ALT	alanine aminotransferase
ANA	Anti-nuclear antibody
ANCA	anti-neutrophil cytoplasmic antibody
AST	aspartate aminotransferase
β-HCG	beta-human chorionic gonadotropin
BP	blood pressure
BUN	blood urea nitrogen
CBC	complete blood count
cGCP	current good clinical practice
CHO	Chinese hamster ovary
CRF	case report form
CT	computed tomography
ECG	Electrocardiogram
EUA	Emergency Use Authorization
FDA	Food and Drug Administration
**GMFI**	**geometric mean fold increase**
**GMT**	**geometric mean titer**
HBV	hepatitis B virus
HCT	hematocrit
HCV	hepatitis C virus
HIV	human immunodeficiency virus
HR	heart rate
hs-CRP	high sensitivity (hs) CRP
ICF	informed consent form
ICH	International Council for Harmonisation
ICTV	International Committee on Taxonomy of Viruses
IEC	independent ethics committee
IDMC	Independent Data Monitoring Committee
IgA	immunoglobulin A
IgG	immunoglobulin G
IM	intramuscular
IRB	institutional review board
N	number of subjects in the dataset or population
N/A	not applicable
ORF	open reading frame
PE	physical examination
**PIMMC**	**potential immune-mediated medical conditions**
RBD	receptor binding domain
RR	respiratory rate
RT-PCR	reverse transcriptase- polymerase chain reaction
SCR	seroconversion rate

SAE	serious adverse event
SUSAR	suspected unexpected serious adverse reaction
TB	tuberculosis
UB-612	United Biomedical, Inc.’s COVID-19 vaccine
VAERD	vaccine-associated enhanced respiratory disease
VS	vital signs
WBC	white blood cell
WoCBP	woman/women of childbearing potential

5 INTRODUCTION

5.1 Study Rationale

The global COVID-19 pandemic caused by the SARS-CoV-2 virus has made the development of an effective vaccine a top biomedical priority. Antibodies are essential elements of most vaccines and will likely be crucial component of an effective vaccine against SARS-CoV-2. Though plasma neutralizing activity is low in most convalescent individuals, the recurrent anti-SARS-CoV-2 RBD antibodies (the most immunogenic fragment within the SARS-CoV-2 Spike protein) with potent neutralizing activity can be found in individuals with unexceptional plasma neutralizing activity, suggesting that humans are intrinsically capable of generating anti-RBD antibodies that potently neutralize SARS-CoV-2. In addition, substantial activation of $CD4^+$ and $CD8^+$ T cells are required to prevent further infection and to help the clearance of virus after exposure. Thus, vaccines that efficiently induce neutralizing antibodies targeting the SARS-CoV-2 RBD and trigger SARS-CoV-2-specific cellular responses are anticipated to induce an optimal immunogenicity profile and achieve the prevention purpose.

5.2 Background

In December 2019, a cluster of patients with pneumonia surfaced in Wuhan, China. The culprit was quickly identified as a beta-coronavirus that has never been reported before, and the disease was named by WHO as Corona Virus Disease 2019 (COVID-19) and the virus that causes it by the International Committee on Taxonomy of Viruses (ICTV) as SARS-CoV-2 [1, 2]. As of January 7, 2020, a global outbreak has caused 87,200,000 confirmed cases in more than 220 countries or territories, with 1,880,000 deaths, making the SARS-CoV-2 pandemic a general public health event that has stirred up worldwide attention. Currently, the epidemic is still spreading and there is no effective means to prevent the infection.

SARS-CoV-2 is a positive-strand RNA virus that belongs to the group of *Betacoronaviruses*. The genome of SARS-CoV-2 is approximately 29,700 nucleotides long and shares 79.5% sequence identity with SARS-CoV [3]. The long ORF1ab polyprotein at 5’ end of the genome encodes 15 or 16 non-structural proteins, and the 3’ end encodes 4 major structural proteins, including the spike (S) protein, nucleocapsid (N) protein, membrane (M) protein, and the envelope (E) protein [4]. SARS-CoV-2 interacts with the receptor angiotensin converting enzyme 2 (ACE2) on host cells via receptor binding domain (RBD) of its S protein for viral entry and subsequent pathogenesis [5], resulting in severe respiratory illness with symptoms of fever, cough, and shortness of breath, and even death in severe cases [6].

Vaccines are the most effective and economical means to prevent and control infectious diseases [7]. The development of an effective vaccine against SARS-Co-2 infection is urgently required. Currently, more than 200 pharmaceutical companies and academic institutions worldwide have launched their programs on vaccine development against SARS-CoV-2. There are several different types of vaccines under development; one of them is subunit vaccine. Subunit vaccines include one or more antigens with strong immunogenicity capable of efficiently stimulating the host immune system. In general, this type of vaccine is safer and easier to produce, but often requires the addition of adjuvants to elicit a strong protective immune response. So far, several institutions have initiated programs on the SARS-CoV-2 subunit vaccine, and almost all of them use the S protein as antigens. For example, the University of Queensland is developing a subunit vaccine based on the “molecular clamp” technology [8]. Clover Biopharmaceuticals Inc. revealed that they

are developing a vaccine candidate against SARS-CoV-2 using the “Trimer-Tag” technology [9], and the trimeric S protein subunit vaccine candidate was produced via a mammalian cell expression system. Novavax, Inc. announced that they had produced multiple nanoparticle vaccine candidates based on S protein, and after assessing efficacy in animal models to identify an optimal vaccine candidate, began Phase I clinical testing in May, 2020. Besides, Johnson & Johnson, Pasteur Institute, Sanofi Pasteru, GSK, and Chongqing Zhifei Biological Products Co., Ltd. also started subunit vaccine development against SARS-CoV-2.

Safety is the most important issue that should be taken into consideration during drug and vaccine development, and some scientists urge that we should not rush to deploy COVID-19 vaccines and drugs without sufficient safety guarantees [10]. There have been concerns regarding vaccine-associated enhanced respiratory disease (VAERD) by certain candidate COVID-19 vaccine approaches, via antibody-dependent enhancement (ADE) or development of Th2 immunopathology [11]. Grifoni *et al.* [12] revealed predominant Th1 responses in convalescing COVID-19 cases, with little to no Th2 cytokines. Clearly more studies are required, but the data Grifoni *et al.* shown appear to predominantly represent a classic Th1 response to SARS-CoV-2.

5.3 UB-612 COVID-19 Vaccine

United Biomedical, Inc. (UBI) has developed a vaccine candidate against SARS-CoV-2 that is designed to activate both humoral and cellular responses. For SARS-CoV-2 immunogens, UB-612 includes a designer S1-RBD-sFc (SRsFc) fusion protein formulated with designer Th and CTL epitope peptides selected from immunodominant M, S2 and N regions known to bind to human MHC I and II. This mixture of designer Th/CTL peptides is designed to elicit T cell activation, memory recall and effector functions similar to that of natural COVID-19. The S1-RBD-sFc fusion protein incorporates both linear and conformation epitopes and induces high affinity antibodies to the RBD of SARS-CoV-2. The immunogen components are formulated with an oligonucleotide containing unmethylated CpG motifs and Adju-Phos® adjuvants, which promotes the activation of antigen-presenting cells pathways to induce an optimal immunogenicity profile and achieve the prevention purpose.

In summary, UB-612 vaccine design composition (S1-RBD-sFc+Th and CTL peptides + CpG, formulated with Adju-Phos®) is expected not only to be safe and induce high titers of neutralizing antibodies, but also to provide T cell memory for a long lasting protection against COVID-19 across all human subjects irrespective of age, sex and ethnicities.

5.4 Phase I study summary (V-122 and V-123)

The First-in-Human (FIH) phase I study began dosing on 25 September 2020 (clinicaltrials.gov**: NCT04545749, protocol number: V-122)** and is ongoing. This is an open-label, dose-escalation clinical study to evaluate the safety, tolerability and immunogenicity of 3 ascending doses of UB-612 COVID-19 vaccine in healthy adults between 20 and 55 years of age. This study **was** carried out in three groups, including A group (receiving 2 doses of UB-612 vaccine 10 μg), B group (receiving 2 doses of UB-612 vaccine 30 μg), and C group (receiving 2 doses of UB-612 vaccine 100 μg). A total of 60 subjects **were** enrolled into the study (20 subjects per groups). Safety monitoring reviews **were** held to decide if the study can go on to second immunization for each cohort or to the higher dose regimens. In the co-primary endpoints, occurrence of adverse reaction within 7 days after vaccination, and the percentage of subjects with ≥ Grade 3 adverse events within 7 days after vaccination **were** calculated. In the secondary endpoints, the immunogenicity

of the UB-612 vaccine was assessed, including GMT, SCR, and geometric mean fold increase of antigen-specific antibody (Anti-S1-RBD). In the exploratory endpoints, GMT, SCR, and geometric mean fold increase of neutralizing antibody, the correlation between immune response detected by ELISA and live virus neutralization test, and T cell responses were evaluated.

A total number of 63 subjects were screened, in which 60 subjects were enrolled equally disturbed in three dose groups, that is 20 subjects receiving UB-612 vaccine 10 μg (A group), 20 subjects receiving UB-612 vaccine 30 μg (B Group), and 20 subjects receiving UB-612 vaccine 100 μg (C Group). This study is still ongoing, and no one withdrew from the study.

In Group A, 13 subjects (65.0%) in the subjects receiving UB-612 vaccine 10 μg had any adverse reactions within 7 days after first vaccination. No any ≥ Grade 3 adverse reaction in UB-612 vaccine 10 μg group within 7 days after first vaccination was reported. For any adverse reactions within 7 days after second vaccination were reported in 11 subjects (55.0%) in UB-612 vaccine 10 μg group. There was no any ≥ grade 3 adverse reactions within 7 days after second vaccination in UB-612 vaccine 10 μg group.

In Group A, all subjects in UB-612 vaccine 10 μg group revealed no detection in antigen-specific antibody before vaccination. The GMT of antigen-specific antibody increased to 73.56(95% CI: 33.458~161.720) at Visit 6 (Day 28), 350.83 (95% CI: 161.630~761.501) at Visit 8 (Day 42) and continued to increase to 526.98 (95% CI: 269.663~1029.839) at Visit 9 (Day 56). At Day 56 after second vaccination, nineteen participants showed a 4-fold change in antigen-specific antibody titer from baseline. The GMT of neutralizing antibody increased to 7.73 (95% CI: 4.704~12.694) at Visit 6 (Day 28), 44.45 (95% CI: 23.017~85.821) at Visit 8 (Day 42), and 41.92 (95% CI: 24.020~73.146) at Visit 9 (Day 56). SCR of neutralizing antibody was observed in 18 (90.0%; 95% CI: 68.30%~98.77%) participants at Visit 9 (Day 56).

In Group B receiving UB-612 vaccine 30 μg, 14 (70.0%) had any adverse reactions within 7 days after first vaccination. No any ≥ Grade 3 adverse reaction within 7 days after first vaccination was found. For any adverse reactions within 7 days after second vaccination were reported in 12 (60.0%). There was no any ≥ grade 3 adverse reactions within 7 days after second vaccination.

All subjects in UB-612 vaccine 30 μg group reported no detection in antigen-specific antibody before vaccination. The GMT of antigen-specific antibody were to 42.75 (95% CI: 21.412 ~ 85.358) at Visit 6 (Day 28), increased to 366.80 (95% CI: 187.085 ~ 719.139) at Visit 8 (Day 42) but decrease to 269.06 (95% CI: 157.931 ~ 458.381) at Visit 9 (Day 56). At Day 56 after second vaccination, 19 (95.0%) participants showed a 4-fold change in antigen-specific antibody titer from baseline. The GMT of neutralizing antibody increased to 4.97 (95% CI: 3.259~7.594) at Visit 6 (Day 28), 31.29 (95% CI: 17.159, 57.060) at Visit 8 (Day 42), and 28.99 (95% CI: 19.101, 43.991) at Visit 9 (Day 56). SCR of neutralizing antibody was observed in 16 (80.0%; 95% CI: 56.34 %~ 94.27 %) participants at Visit 9 (Day 56).

In Group C receiving UB-612 vaccine 100 μg, 16 (80.0%) had any adverse reactions within 7 days after first vaccination. No any ≥ Grade 3 adverse reaction within 7 days after first vaccination was found. For any adverse reactions within 7 days after second vaccination were reported in 7 (35.0%). There was no any ≥ grade 3 adverse reactions within 7 days after second vaccination. All subjects reported no detection in antigen-specific antibody before vaccination. The GMT of antigen-specific antibody were to 103.20 (95% CI: 48.132 ~ 221.265) at Visit 6 (Day 28), increased to 2240.15 (95% CI: 1233.797 ~ 4067.353) at Visit 8 (Day 42). In additional, The GMT of antigen-specific antibody at Visit 9 (Day 56) in first 6 subjects was 897.44 (95% CI: 613.808 ~ 1312.121).

At Day 42 after second vaccination, 20 (100%) participants showed a 4-fold change in antigen-specific antibody titer from baseline. At Day 56 after second vaccination, first 6 participants all showed a 4-fold change in antigen-specific antibody titer from baseline. The GMT of neutralizing antibody increased to 10.84 (95% CI: 5.859~20.048) at Visit 6 (Day 28) and 107.66 (95% CI:72.402 ~160.087) at Visit 8 (Day 42). At Day 56, GMT of neutralizing antibody first 6 participants was 88.79 (95% CI: 51.828, 152.108). SCR of neutralizing antibody were 100% (95% CI: 83.16% ~ 100.00%) observed at Visit 8 (Day 42). At Day 56, SCR of neutralizing antibody was 100% (95% CI: 54.07% ~ 100%) in first 6 participants at Visit 9 (Day 56).

V-123 is an extension study (clinicaltrials.gov: NCT04967742) to evaluate the safety, tolerability and immunogenicity of one booster dose of UB-612 COVID-19 vaccine in adults who completed two vaccinations of UB-612 vaccine at 10, 30, and 100 µg in V-122 study.

The subjects who completed two vaccinations in Phase I study will be recruited in the extension study. After the inform consent is obtained from the subject, eligible subjects will receive one booster dose of UB-612 vaccines 100 µg with the same dosing which was offered in Phase II study, at least 6 months after the second vaccination.

Preliminary data showed extremely high immune response (Figure 1) after 14 days post 3rd vaccination. No SAE had been reported since then. The incidence of local reactions increased, but no grade 3/4 AE recorded.

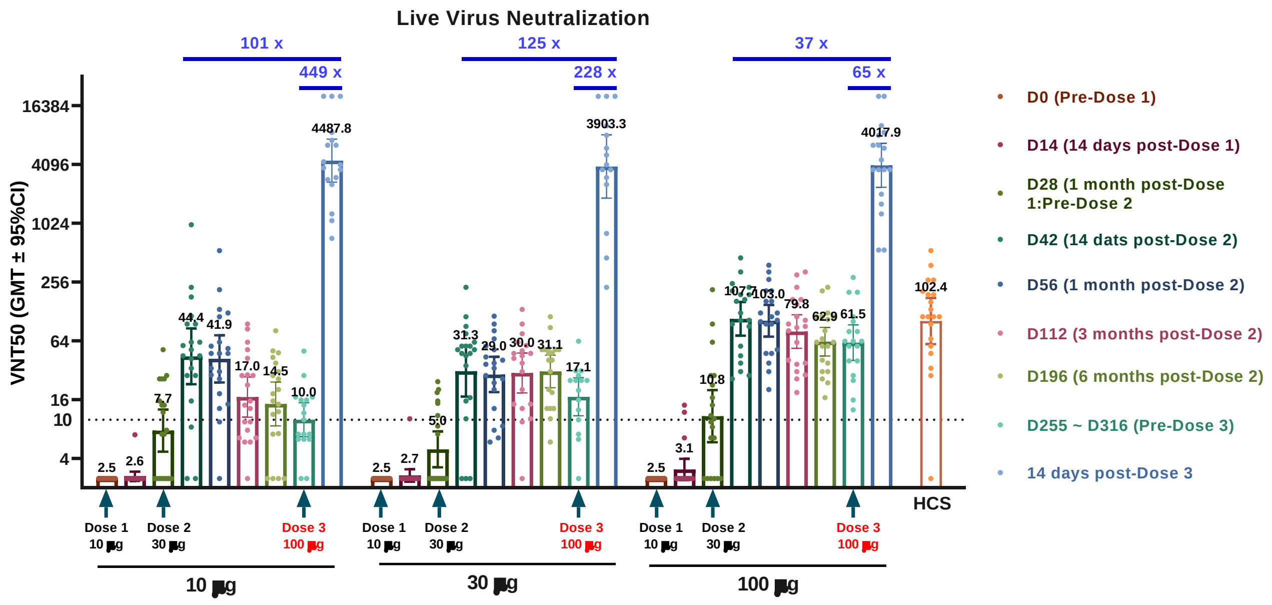

Figure 1. Neutralizing antibody titers after 3rd vaccination measured by wild-type live virus assay

5.5 Risk/Benefit Assessment

Nonclinical studies of UB-612 antigens have shown that IM delivery of the vaccine is (1) safe and well-tolerated in tested animals and (2) effective in inducing potent anti-S1-RBD antibody responses. Antibodies elicited by UB-612 antigens exhibit high neutralizing activity, which blocks

the protein-protein interaction between ACE2 and S1-RBD and prevent SARS-CoV-2-induced *in vitro* cytopathology. Regarding to the ongoing phase 1 study, based on DSMB judgement, there was no safety concern in UB-612 at 10 μg, 30 μg and 100 μg.

Based on the current report, administration 100 μg of UB-612 vaccine was safe and tolerable. The first 6 subjects' immune responses in neutralizing antibody titer at Day 42 could achieve 107, close to the human convalescent serum. We aim to further evaluate the immunogenicity, tolerability and safety of 100 μg UB-612 in adults and adolescents in phase II study.

Since no Grade 3/4 solicited AE and SAE reported from V-123, administration of 3rd dose of 100 μg of UB-612 vaccine is expected to be safe and tolerable. And based on immune response from V-123, the booster effect of 3rd vaccination on Visit 6 (Day 197-242) could also be expected.

6 STUDY OBJECTIVES & ENDPOINTS

6.1 Primary Objective(s)

- To evaluate the SARS-CoV-2 neutralizing antibody titer induced by UB-612 vaccine
- To evaluate the safety and tolerability of the UB-612 vaccine after vaccination

6.2 Primary Endpoint(s)

Immunogenicity Endpoint(s)

- Geometric mean titer (GMT) of SARS-CoV-2 neutralizing antibody on Day 57
- Seroconversion rate (SCR) of SARS-CoV-2 neutralizing antibody on Day 57

Safety Endpoint(s)

- Local reactions for up to 7 days following each dose
- Systemic events for up to 7 days following each dose
- Unsolicited AEs from Day 1 to Day 57
- MAAEs and SAEs from Day 1 to Day **365**
- AESIs and ADEs from Day 1 to Day **365**

6.3 Secondary Objective(s)

- To evaluate the immune response to SARS-CoV-2 during the study
- To evaluate the lot consistency of immune responses induced by 3 independent batches of vaccine

6.4 Secondary Endpoint(s)

Immunogenicity Endpoint(s)

- Seroconversion rate (SCR) of antigen-specific antibody (Anti-S1-RBD) on Day 57
- Geometric mean titer (GMT) of SARS-CoV-2 neutralizing antibody on Day 197 and 365
- Geometric mean titer (GMT) of antigen-specific antibody (Anti-S1-RBD) on Day 57, 197 and 365
- Geometric mean fold increase in **SARS-CoV-2 neutralizing antibody and** antigen-specific antibody (Anti-S1-RBD) on Day57, 197 and 365
- Lot consistency as assessed by the comparisons of the GMT of SARS-CoV-2 neutralizing antibody on Day 57 induced by 3 independent UB-612 vaccine clinical materials. The 95% confidence intervals between groups will be within the margin of 0.5 to 2.

Safety Endpoint(s)

- Changes of safety laboratory measures

6.5 Exploratory Objective(s)

- To evaluate the T cell function induced by **UB-612** vaccine
- To evaluate the safety and immunogenicity of the UB-612 vaccine in adolescents

- To evaluate the efficacy of UB-612 vaccine
- To describe the serological responses to the UB-612 vaccine in confirmed and/or severe COVID-19 cases
- **To evaluate antibody against SARS-CoV-2 antigens**
-

6.6 Exploratory endpoint(s)

- T cell responses to UB-612 measured by ELISpot and flow cytometric assays on Day 57
- **T cell responses to UB-612 measured by ELISpot and flow cytometric assays on 14 days post 3rd dose of UB-612**
- **Geometric mean titer (GMT) of SARS-CoV-2 neutralizing antibody on 14 days post 3rd dose of UB-612**
- **Geometric mean titer (GMT) of antigen-specific antibody (Anti-S1-RBD) 14 days post 3rd dose of UB-612**
- **Geometric mean fold increase in SARS-CoV-2 neutralizing antibody and antigen-specific antibody (Anti-S1-RBD) on14 days post 3rd dose against pre-3rd dose baseline**
- **Geometric mean titer (GMT) of SARS-CoV-2 neutralizing antibody and antigen-specific antibody (Anti-S1-RBD) on Day 57, Day 197 and Day 365 in adolescents**
- **Seroconversion rate (SCR) of SARS-CoV-2 neutralizing antibody and antigen-specific antibody (Anti-S1-RBD) on Day 57 in adolescents**
- **Geometric mean fold increase in SARS-CoV-2 neutralizing antibody and antigen-specific antibody (Anti-S1-RBD) on Day 57, Day 197, and Day 365 in adolescents**
- Local reactions for up to 7 days following each dose in adolescents
- Systemic events for up to 7 days following each dose in adolescents
- Unsolicited AEs from Day 1 to Day 57 in adolescents
- MAAEs and SAEs from Day 1 to Day **365** in adolescents
- AESIs and ADEs from Day 1 to Day **365** in adolescents
- Changes of safety laboratory measures in adolescents
- COVID-19 incidence per 1000 person-years of follow-up based on PCR test
- To describe the anti-S1-RBD IgG levels and SARS-CoV-2 neutralizing titers to UB-612 in confirmed and/or severe COVID-19 cases
- **To detect antibody against SARS-CoV-2 antigens derived from S2, N, and M protein**

7 INVESTIGATIONAL PLAN

7.1 Overall Study Design and Plan: Description

This is a phase II, observer-blind, multiple-centre, randomized, placebo-controlled study to evaluate the immunogenicity, safety, tolerability and lot consistency of 2 doses of UB-612 vaccine in adolescent, younger and elderly adults. Around 3850 adult subjects will be randomized to be composed of the core group for EUA application, while around 385 adolescents will be randomized to be the supplementary group for broader indication. All subjects will be randomly allocated to receive 2 doses of 100 µg vaccine or placebo in a 6:1 ratio, including 462 aged from >18 to < 65 years old, evaluable subjects in the lot-to-lot consistency group. As for immunogenicity, at least 350 evaluable young **adults** (aged >18 to < 65 years old) and 154 evaluable elderly (aged ≥ 65 years old) will be enrolled for descriptive analysis. Subjects in immunogenicity group should be enrolled first. All subjects will be included in the safety group, and it is intended that a minimum of 770 subjects will be randomized to be in the ≥ 65-year stratum. Adolescents will start to enrol after recruitment of the core group has been completed. Around 385 adolescents will be randomized to be allocated in 6:1 ratio, in which 154 evaluable adolescents will have immunogenicity data to compare with adults.

It will be consisted of 7 clinical visits and one long-term follow-up visit. All subjects will have blood test for safety before and after full vaccination. Subjects in lot-to-lot consistency and immunogenicity group will also have blood drawn for immune response, in which tests for T cell function will be optional. Subjects will come to the clinics at Visit 1 for screening, Visit 2 (Day 1, baseline) for randomization and 1st vaccination, Visit 3 (Day 29) for 2nd vaccination, Visit 4 (Day 57) for safety check and immunogenicity assessment, and Visit 5 (Day 197) for safety check and assessment of the persistence of immune response. **Subjects will also be unblinded at Visit 5. Subjects in placebo group will withdraw from the study and subjects in vaccine group will be encouraged to have 3rd dose of vaccination at Visit 6 (Day 197~Day 242). Those who received 3rd dose will have Visit 7 (14 days after Visit 6) to check the booster effect. After Day 197, subjects will enter the long-term follow-up with a safety call bi-monthly.** Subjects in lot-to-lot and immunogenicity group will be encouraged to visit site at Day 365 to check the immune persistence. Thus, subjects will be expected to participate for up to a maximum of approximately 13 months. Unblinding will be carried out when **subjects** in core group or supplementary group have completed Visit 5. For females of child-bearing potential, a urine pregnancy test will be performed before **each** vaccination and will be found negative. Female subjects or the female partners of male subjects who are pregnant during the study period will be followed the pregnancy outcomes.

Before any study-related procedures are performed, voluntary, written, study-specific informed consent will be obtained from the subjects. **Subjects will be screened at Visit 1.** All subjects will have blood sampling for hematology and biochemistry. After checking the eligibility of the subject at Visit 2, the randomization number and adjoining study intervention allocation will be assigned. **Subjects in lot-to-lot consistency and immunogenicity group will have blood drawn for immunogenicity before vaccination.** Unblinded site staff member(s) will dispense/administer 0.5 mL UB-612/placebo into the deltoid muscle of the preferably nondominant arm. Blinded site staff must observe the subject for at least 30 minutes after study vaccine administration for changes in vital signs or any acute anaphylactic reactions. The investigator will provide instructions for reactogenicity e-diary completion and ask the subject to complete the reactogenicity e-diary from

Day 1 to Day 7, skin allergic reaction e-diary from Day 1 to Day 14, with Day 1 being the day of vaccination. Participants will be instructed to contact the site staff or investigator immediately if he or she experiences any of the Grade 3/4 AEs from Day 1 to Day 7 to determine if an unscheduled reactogenicity visit is required. Apart from reactogenicity, allergic skin rash should be monitored for 14 days post vaccination. **Once subject encountered the Grade 3/4 allergic skin rash, site staff should call for detail to evaluate the necessarily of unscheduled visit. Investigators could ask for HLA typing survey if he/she concerned about the possibility of hypersensitivity. Of course, IDMC could also request HLA typing once they reviewed the safety data.**

At Visit 3 (Day 29), the 2nd study vaccine administration will be done and subjects will be closely monitored for 30 minutes after vaccination for changes in vital signs or acute anaphylactic reaction. The 7/14-day post-vaccination e-diary will be given to subjects with a suitable instruction. For females of child-bearing potential, a urine pregnancy test will be performed before vaccination and found negative. At Visit 4 (Day 57, 1-month follow-up visit after 2nd vaccination), collect sample for safety and immunogenicity, review the participants' reactogenicity e-diary data and record any unsolicited AE.

All subjects will come back on Day 197, Visit 5, for immunogenicity persistence check in immunogenicity group and safety check for safety group. **Once subject completes Visit 5, he or she will be unblinded. Subjects received placebo will withdraw from the study and subjects who received UB-612 vaccine will be invited to join the extension study to determine the durability of the immune response and long-term safety after unblinding. Meanwhile, subjects in vaccine group will be encouraged to have a third dose of UB-612.All subjects who received UB-612 vaccine will be followed up for 12 months.**

All subjects will receive safety calls on Day 8, Day 15, Day 22, Day 36, Day 43, Day 64, Day 71, Day 78, Day 85, Day 253, and Day 309 that will serve both to monitor for unsolicited AEs or/and to monitor for symptoms of COVID-19. After Day 57, each week (i.e., every 7 days) the subject will receive a prompt on their smartphone device to regularly surveillance for signs and symptoms of COVID-19 and SAE. **Subjects who received 3rd dose of UB-612 will receive an extra safety call on 7 days post 3rd vaccination.**

If a Grade 3 local reaction, systemic event, or fever are reported in the reactogenicity e-diary, a telephone contact should occur to ascertain further details and determine whether a site visit is clinically indicated. If suspected Grade 4 local reaction systemic event and /or fever is reported in the reactogenicity e-diary, a site visit should occur to confirm whether the event meets the criteria for Grade 4. All subjects were encouraged to contact site staff once they encountered Grade 3/4 AE. Allergic skin reaction should be monitored for 14 days post vaccination and should be contacted regardless of severity.

If a subject experiences a febrile illness associated with respiratory symptoms, he or she is instructed to contact the site immediately. Subjects may utilize a COVID-19 illness e-diary to prompt him/her to report any symptoms.

7.1.1 End of Study Definition

The end of study is defined as the date of last visit of the last participant in the study. A subject is supposed to have completed the study if he/she has completed all data required for this study.

7.2 Schedule of Assessments

Below is a list of all study procedures through the study period and the signs “X” indicate when the procedures are performed.

Lot-to-lot consistency & Immunogenicity group

Scheduled visit	***1[m]***	***2[m]***		***3***		***4***		***5/ET***	***6[t]***		***7***		***Long-term follow-up***
Test & observations	***Screening***	***1st vaccination***		***2nd vaccination***		***Follow-up***		***Unblinding***	***3rd vaccination[h]***		***Follow-up[h]***		***Month 12 follow-up[i]***
Day	***-28~-1***	*1*	*8, 15, 22*	*29 ±3 days*	*36, 43*	*57 ±3 days*	*64, 71, 78, 85*	*197 ±15 days*	***197~242***	***7 days after Visit 6***	***14 days after Visit 6 ±3 days***	*253, 309*	*365 ±45 days*
Informed consent	X								**X[h]**				
Inclusion/Exclusion Criteria	X	X											
Randomization		X											
**Contraindication to vaccination**				X					**X[h]**				
Demographics	X												
Medical history	X	X											
Physical Exam[a]	X	X		X		X		X	**X[h]**		**X[h]**		
Vital sign	X	X		X		X		X	**X[h]**		**X[h]**		X
ECG	X												
**Lab (Safety)**													
Blood routine[j]	X					X			**X[h]**		**X[h]**		
Biochemistry[j]	X					X			**X[h]**		**X[h]**		
Immunology[j]	X					X			**X[h]**		**X[h]**		
Pregnancy[b]		X		X					**X[h]**				
Urinalysis[b]	X	X[q]		X[q]		X[q]		X[q]	**X[h, q]**		**X[h, q]**		
**Lab (Immunogenicity)**													

Scheduled visit	***1***[m]	***2***[m]		***3***		***4***		***5/ET***	***6***[t]		***7***		***Long-term follow-up***
Test & observations	***Screening***	***1***[st] ***vaccination***		***2***[nd] ***vaccination***		***Follow-up***		***Unblinding***	***3rd vaccination***[h]		***Follow-up***[h]		***Month 12 follow-up*** [i]
Day	***-28~-1***	*1*	*8, 15, 22*	*29 ±3 days*	*36, 43*	*57 ±3 days*	*64, 71, 78, 85*	*197 ±15 days*	***197~242***	***7 days after Visit 6***	***14 days after Visit 6 ±3 days***	*253, 309*	*365 ±45 days*
Immune response assessment [c]		X		X [n]		X		X					X
T cell function (optional [o])		X				X							
**Lab (Exploratory)**													
**T cell function (optional [u])**									**X [h]**		**X [h]**		
**Spared sample [r]**	**X**					**X**							
**Immune response**[s]									**X [h]**		**X**[h]		
Vaccination		X		X					**X [h]**				
e-Diary instruction		X		X					**X [h]**				
Safety calls [d]			X		X		X			**X**[s ,h]		X	
AEs/AESIs [p] /MAAEs/SAEs		X [k]	X [k]	X [k]	X [k]	X [k]	X [k]	X [l]	**X [l, h]**	**X [l, h]**	**X [l, h]**	X [l]	X [l]
COVID-19 infection surveillance		X	X	X	X	X [e]	X [e]	X [e]	**X [e, h]**	**X [e, h]**	**X [e, h]**	X [e]	X [e]
Concomitant Medication		X		X		X		X [f]	**X [f, h]**		**X [f, h]**		

a: Body weight and height will be assessed at Visit 1 only.
b: Screening for pregnancy and proteinuria will be performed by urine sample on Day 1, Day 29. When positive urine pregnancy test is presented, it should be confirmed by a serum test. Serum testing in lieu of urine tests will not be considered a protocol deviation. Proteinuria is checked via urine routine as baseline.
c: For anti-S1-RBD IgG levels, SARS-CoV-2 neutralizing titers, and antibody titers which inhibit S1-RBD: ACE2 binding.
d: All subjects will receive safety calls that will serve both to monitor for unsolicited AEs, including AESIs, and to monitor for symptoms of COVID-19.
e: Each week (i.e., every 7 days) the subject will receive a prompt on their smartphone device to regularly surveillance for signs and symptoms of COVID-19. Record medication for potential COVID-19 on COVID-19 page.
f: Only record medications for MAAE and SAE.

g: 28 days after 2nd vaccination
h: **Only for subjects in vaccine group and have agreed to receive 3rd dose of UB-612.**
i: 12 months (336 days) after 2nd vaccination
j: Safety laboratory includes complete blood count (Hgb, Hct, RBC), WBC with differential, platelet count, creatinine, ALT, AST, total and direct bilirubin, hs-CRP, and ANA.
k: Active collection period
l: Passive surveillance period.
m: Visit 1 and 2 could be emerged as one visit.
n: Anti-S1-RBD IgG level only
o: Up to 100 aged >18-< 65 subjects in selected sites will be invited
p: To include PIMMC (listed in **Table 9-6**), or any newly identified potential AESI followed through 12 months after participants' final vaccination. Complications of COVID-19, also termed as ADE, (listed in **Table 9-5**) should be considered and reported as AESIs.
q: If subject encountered ≥ Grade 3 hypertension, the existence or deterioration of proteinuria will be checked .
r: An aliquot of spared serum is to be frozen and stored for UBI SARS-CoV-2 ELISA, Confirmatory SARS-CoV-2 ELISA, and future immunologic tests.
s: Subjects who qualified to receive 3rd dose will enter 2 weeks e-diary for follow-up and a safety call post 7 days post vaccination.
t: Visit 5 and Visit 6 could be the same day.
u: Approximately thirty aged >18-< 65 subjects and approximately thirty aged ≥ 65 years subjects in selected sites will be invited. When applicable, subjects who were assessed for T cell functions on Day 57 are preferred to be invited.
ADE (Antibody Dependent Enhancement), AESIs (Adverse Event of Special Interests), ANA (Anti-Nuclear Antibody), ET (Early Termination), MAAEs (Medically Attend Adverse Events)

Safety check group

Scheduled visit	***1*** [l]	***2*** [l]		***3***		***4***		***5/ET***	***6***[q]		***7***		***Long-term follow-up***
Test & observations	***Screening***	***1st vaccination***		***2nd vaccination***		***Follow-up***[f]		***Unblinding***	***3rd vaccination***[g]		***Follow-up***[g]		***Month 12 follow-up*** [h]
Day	***-28~-1***	*1*	*8, 15, 22*	*29 ±3 days*	*36, 43*	*57 ±3 days*	*64, 71, 78, 85*	*197 ±15 days*	***197~242***	***7 days after Visit 6***	***14 days after Visit 6 ±3 days***	*253, 309*	*365 ±45 days*
Informed consent	X								**X** [g]				
Inclusion/Exclusion Criteria	X	X											
Randomization		X											
**Contraindication to vaccination**				X					**X** [g]				
Demographics	X												
Medical history	X	X											
Physical Exam [a]	X	X		X		X		X	**X** [g]		**X** [g]		
Vital sign	X	X		X		X		X	**X** [g]		**X** [g]		X
ECG	X												
**Lab (Safety)**													
Blood routine [i]	X					X			**X**[g]		**X**[g]		
Biochemistry [i]	X					X			**X**[g]		**X**[g]		
Immunology [i]	X					X			**X**[g]		**X**[g]		
Pregnancy [b]		X		X					**X**[g]				
Urinalysis [b]	X	X[n]		X[n]		X[n]		X[n]	**X**[g, n]		**X**[g, n]		
**Lab (Exploratory)**													
**Spared sample** [o]	**X**					**X**							

Scheduled visit	1[l]	2[l]		3		4		5/ET	6[q]		7		Long-term follow-up
Test & observations	Screening	1st vaccination		2nd vaccination		Follow-up[f]		Unblinding	3rd vaccination[g]		Follow-up[g]		Month 12 follow-up [h]
Day	-28~-1	1	8, 15, 22	29 ±3 days	36, 43	57 ±3 days	64, 71, 78, 85	197 ±15 days	197~242	7 days after Visit 6	14 days after Visit 6 ±3 days	253, 309	365 ±45 days
**Immune response [g]**									X [g]		X [g]		X [g]
Vaccination		X		X					X [g]				
e-Diary instruction		X		X					X [g]				
Safety calls [c]			X		X		X			X[p, g]		X	
AEs/AESIs[m]/MAAEs/SAEs		X [j]	X [j]	X [j]	X [j]	X [j]	X [j]	X [k]	X [k, g]	X [k, g]	X [k, g]	X [k]	X [k]
COVID-19 infection surveillance		X	X	X	X	X [d]	X [d]	X [d]	X [d, g]	X [d, g]	X [d, g]	X [d]	X[d]
Concomitant Medication		X		X		X		X [e]	X [e, g]		X [e, g]		

a: Body weight and height will be assessed at Visit 1 only.
b: Screening for pregnancy and proteinuria will be performed by urine sample on Day 1 and Day 29. When positive urine pregnancy test is presented, it should be confirmed by a serum test. Serum testing in lieu of urine tests will not be considered a protocol deviation. Proteinuria will be checked via urine routine as baseline.
c: All subjects will receive safety calls that will serve both to monitor for unsolicited AEs, including AESIs, and to monitor for symptoms of COVID-19.
d: Each week (i.e., every 7 days) the subject will receive a prompt on their smartphone device to regularly surveillance for signs and symptoms of COVID-19. Record medication for potential COVID-19 on COVID-19 page.
e: Only record medications for MAAE and SAE.
f: 28 days after 2nd vaccination
g: Only for subjects in vaccine group and have agreed to receive 3rd dose of UB-612.
h: 12 months (336 days) after 2nd vaccination
i: Safety laboratory includes complete blood count (Hgb, Hct, RBC), WBC with differential, platelet count, creatinine, ALT, AST, total and direct bilirubin, hs-CRP, and ANA.
j: Active collection period
k: Passive surveillance period,
l: Visit 1 and 2 could be emerged as one visit.
m: To include PIMMC (listed in **Table 9-6**), or any newly identified potential AESI followed through 12 months after participants' final vaccination. Complications of COVID-19, also termed as ADE, (listed in **Table 9-5**) should be considered and reported as AESIs.
n: If subject encountered ≥ Grade 3 hypertension, the existence or deterioration of proteinuria will be checked.

o: An aliquot of spared serum is to be frozen and stored for UBI SARS-CoV-2 ELISA, Confirmatory SARS-CoV-2 ELISA, anti-S1-RBD IgG ELISA, S1-RBD:ACE2 binding inhibition ELISA, and future immunologic tests.
p: Subjects who qualified to receive 3rd dose will enter 2 weeks e dairy for follow-up and a safety call post 7 days post vaccination.
q: Visit 5 and Visit 6 could be the same day.
ADE (Antibody Dependent Enhancement), AESIs (Adverse Event of Special Interests), ET (Early Termination), MAAEs (Medically Attend Adverse Events)

Blood collection at scheduled visits
Lot-to-lot consistency & Immunogenicity group

Scheduled visit	***1***	***2***		***3***		***4***		***5/ET***	***6***		***7***		***Long-term follow-up***
Test & observations	***Screening***	***1st vaccination***		***2nd vaccination***		***Follow-up***		***Unblinding***	***3rd vaccination a***		***Follow-up a***		***Month 12 follow-up***
Day	***-28~-1***	*1*	*8, 15, 22*	*29 ±3 days*	*36, 43*	*57 ±3 days*	*64, 71, 78, 85*	*197 ±15 days*	***197~242***	***7 days after Visit 6***	***14 days after Visit 6 ±3 days***	*253, 309*	*365 ±45 days*
**Lab (Safety)**													
Blood routine	4.5					4.5			**4.5**		**4.5**		
Biochemistry	**3**					**3**			**3**		**3**		
Immunology	3					3			**3**		**3**		
**Lab (Immunogenicity)**													
Immune response assessment		20		5		20		20					20
**Lab (Exploratory)**													
**Spared sample**	**5**					**5**							
**Immune response***									**10b**		**10**		
Total blood collection	**15.5**	20		5		**35.5**		20	**20.5**		**20.5**		20

Total amount of blood collection: **157 mL.**
* An additional 56 mL of blood sample will be collected for the optional T cell assessments at **Visit 2, Visit 4, Visit 6 and Visit 7.**
a: Only for subjects in vaccine group and have agreed to receive 3rd dose of UB-612. An additional 10 mL of blood sample will be collected at Visit 6 and 14 days after Visit 6.
b: If Visit 5 and Visit 6 are the same visit, the 10mL blood sample for exploratory immune response will be not collected.

Safety check group

Scheduled visit	***1***	***2***		***3***		***4***		***5/ET***	***6***		***7***		***Long-term follow-up***
Test & observations	***Screening***	***1st vaccination***		***2nd vaccination***		***Follow-up***		***Unblinding***	***3rd vaccination[a]***		***Follow-up[a]***		***Month 12 follow-up***
Day	***-28~-1***	*1*	*8, 15, 22*	*29 ±3 days*	*36, 43*	*57 ±3 days*	*64, 71, 78, 85*	*197 ±15 days*	***197~242***	***7 days after Visit 6***	***14 days after Visit 6 ±3 days***	*253, 309*	*365 ±45 days*
**Lab (Safety)**													
Blood routine	4.5					4.5			**4.5**		**4.5**		
Biochemistry	**3**					**3**			**3**		**3**		
Immunology	3					3			**3**		**3**		
**Lab (Exploratory)**													
**Spared sample**	**5**					**5**							
**Immune response**									**10**		**10**		**10**
Total blood collection	**15.5**					**15.5**			**20.5**		**20.5**		**10**

Total amount of blood collection: **82** mL.

a: Only for subjects in vaccine group and have agreed to receive 3rd dose of UB-612. An additional 10 mL of blood sample will be collected at Visit 6, 14 days after Visit 6, and Day 365.

Diagram of the study design (N represented evaluable numbers)

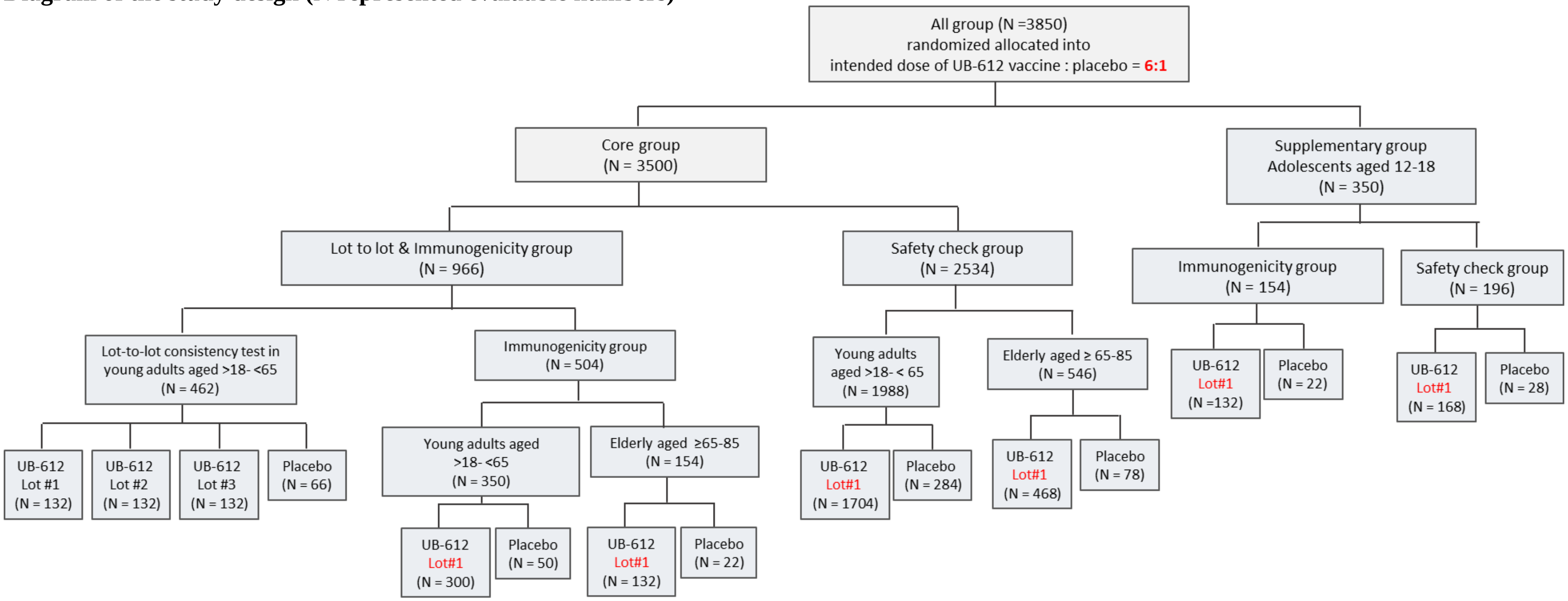

7.3 Discussion of Study Design

The primary objectives of this study are to evaluate the immune response after full immunization. Secondary objectives are to evaluate persistence of immune response, and safety and tolerability of UB-612 vaccine to prevent SARS-CoV-2 infection up to 12 months. COVID-19 can affect anyone, and the disease can cause symptoms ranging from mild to very severe. For some other illnesses caused by respiratory viruses (such as influenza), some people may be more likely to have severe illness than others because they have characteristics or medical conditions that increase their risk. These are commonly called "risk factors." Examples include older age or having certain underlying medical conditions. Older adults are at greatest risk of severe disease and death due to coronavirus disease 2019 (COVID-19). Globally, persons older than 65 years comprise 9% of the population, yet account for 30% to 40% of cases and more than 80% of deaths [13]. COVID-19 is a new disease. Currently there are limited data and information about the impact of many underlying medical conditions on the risk for severe illness from COVID-19. In this study design, adults of any age might be at an increased risk for severe illness from the virus that causes COVID-19 as defined who had at least one of the Charlson comorbidity index [14] category or obesity only (BMI $\geq$30 kg/m^2).

Per EUA requirement issued by TFDA, the sponsor should submit the lot-to-lot consistency report to demonstrate the quality of vaccine product. Core group is designed for EUA application. Among 3850 subjects, 462 evaluable subjects aged from >18 to < 65 will be enrolled for lot-to-lot consistency. Additional 350 evaluable adult subjects will be assigned into immunogenicity study. In order to demonstrate vaccine efficacy in elderly, authority requests EUA package should include at least 600 elder subjects who received the vaccine. Thus we plan to enrol around 770 randomized subjects aged from ≥ 65 to 85 years old, in which at least 154 evaluable subjects will have immunogenicity data to compare with young adults. After completion recruitment of the core group, 385 adolescents will be randomly recruited as supplementary group to extend vaccine indication. At least 154 evaluable adolescents (allocation rate 6:1) will have immune data to compare with adults.

There are two interim reports for EUA application. The first is for safety follow up in core group, and the second is for lot-to-lot consistency. Also, an additional interim report of immunogenicity and safety in adolescents, will be submitted for indication extension.

The surveillance for COVID-19 will be conducted as part of the study. The serological evidences in case of confirmed and/or severe COVID-19 will be described for the change of antibody level in vaccinated subjects. All subjects would be included in the exploratory objectives of protective efficacy against COVID-19 if case detected.

Unblinding will be carried out when the subject in core group or supplementary group has completed Visit 5. Subjects who receive the placebo vaccine will allow to withdraw from the study.

7.4 Selection of Study Population

7.4.1 Number of Planned Subjects

In core group, around 3850 adult subjects will be randomized to receive 100 µg UB-612 vaccine or placebo with 6:1 allocation rate. In supplementary group, around 385 adolescent subjects aged

from 12 to 18 years old will randomly receive UB-612 vaccine 100 µg or placebo with allocation rate 6:1, too.

7.4.2 Inclusion Criteria

To be eligible for study entry subjects must satisfy all of the following inclusion criteria:

1. Healthy male or non-pregnant female between the age of 12 to 85 years at time of enrolment.
2. Women of childbearing potential and men must agree to practice medically effective contraception from first vaccination until 3 months after the last vaccination. The acceptable effective contraception methods include:
    a. Male or female sterilization, implant, or intrauterine device;
    b. Injectable, pill, patch, ring plus one barrier method*;
    c. Two combined barrier methods*.

    *Effective barrier methods are diaphragm, male or female condoms, sponge, or spermicides (creams or gels that contain a chemical to kill sperm).
3. Able to understand the content and possible risks of informed consent and willing to sign the Informed Consent Form (ICF).
4. Able to understand and agrees to comply with all study procedures and be available for all study visits.
5. Ear temperature ≤ 38.0°C.
6. Healthy participants** who are determined by medical history, physical examination, and clinical judgment of the investigator to be eligible for inclusion in the study. In the investigator's clinical judgement, participant may have a stable and well-controlled comorbidity associated with an increased risk of progression to severe COVID-19.

    ** Healthy participants with preexisting stable disease, defined as disease not requiring significant change in therapy or hospitalization for worsening disease during the 12 weeks before enrolment and unlikely to require a significant change in therapy or hospitalization in the six months following enrollment, can be included.

7.4.3 Exclusion Criteria

Subjects will be excluded from the study if one or more of the following exclusion criteria is applicable:

1. History of anaphylaxis, urticarial, or other significant adverse reaction requiring medical intervention after receipt of a vaccine.
2. Female who is pregnant or positive in pregnancy test at screening or just prior to each vaccination administration.
3. Female who is breast-feeding or plans to breastfeed from the time of the first vaccination through 60 days after the last vaccination.
4. Any acute illness, as determined by the study investigator 3 days before first vaccination (these subjects can be re-scheduled).

5. Any major surgery one month before first vaccination (these subjects can be -rescheduled)
6. Known HIV antibody positive
7. Known active hepatitis B and hepatitis C disease. Active hepatitis means liver aminotransferase (AST and/or ALT) greater than 3xULN, and/or total bilirubin greater then 3xULN at screening.
8. Previous exposure to SARS-CoV-2 or receipt of an investigational or licensed product for the prevention of COVID-19, MERS or SARS.
9. Have history of Guillain-Barre syndrome.
10. Subjects who take part in another clinical study within 12 weeks prior to the day of informed consent.
11. Immune deficiency/disorder, whether due to genetic defect, immunodeficiency disease or immunosuppressive therapy
12. Subjects who plan to or are undergoing anti-cancer therapy
13. Platelet disorder or other bleeding disorder may cause injection contraindication.
14. Prior chronic administration (defined as ≥ 14 day of continuous use) of immunosuppressant or corticosteroids (equivalent to ≥ 20 mg daily of prednisone), cytotoxic treatment in last 6 months before first vaccination.
15. Prior administration of immunoglobulins and/or any blood products in last 4 months before first vaccination.
16. Receipt of any seasonal or pandemic influenza vaccine within 14 days, or any other nonstudy vaccine within 28 days, before study intervention administration.
17. Anticipated receipt of any seasonal or pandemic influenza vaccine within 14 days, or any other nonstudy vaccine within 28 days, after study intervention administration.
18. Receipt of short-term (<14 days) systemic corticosteroids. Study intervention administration should be delayed until systemic corticosteroid use has been discontinued for at least 28 days. Inhaled/nebulized, intra-articular, intrabursal, or topical (skin or eyes) corticosteroids are permitted.
19. Loss or donation of blood over 500 mL within 3 months prior to Screening Visit or intention to donate blood or blood products for transfusion during the study.
20. Any medical disease or condition that, in the opinion of the study investigator, may confound the results of the study or pose an additional risk to the subjects by their participation in the study.
21. Employees at the investigator's site, of the Sponsor or the contract research organization (CRO) **who** directly involved in the conduct of the study.

7.4.4 Contraindications to Vaccination

7.4.4.1 Criteria for Delay of Study Treatment

The following conditions constitute a contraindication to vaccination and should be checked prior to second **and third** vaccinations:

1. Subject has a fever or other acute illness within 24 hours before vaccination.
2. Clinically significant acute illness at the time of vaccination, including concurrent symptoms which may be considered as a potential COVID-19 illness. This does not include minor illnesses, such as diarrhea or mild upper respiratory tract infection.
3. An illness which in the judgement of the investigator may interfere with reactogenicity and 7-day safety assessments after vaccination.

If any AEs other than listed above or fever occurred at the time scheduled for vaccination, the subject may be vaccinated at a later date no later than 7 days when symptoms or fever are resolved.

7.4.4.2 Criteria for Discontinuation of Study Treatment

The following conditions constitute a contraindication to vaccination and should be checked prior to second **and third vaccinations**:

1. Has any Grade 4 adverse reaction related to IP within 7 days after **previous vaccination**.
2. Has any SAE related to **previous** dose related to IP during the follow-up of **previous vaccination**.
3. Subject has or develops the symptom or condition listing in 1 or more exclusion criteria.
4. Subject diagnoses with SARS-CoV-2 infection or has suspected SARS-CoV-2 infection based on symptoms according to investigator's judgment (lab test conformation is not necessary).
5. Subject becomes pregnant.
6. Any condition that in the opinion of the investigator would be a contraindication to **vaccinations.**

Any subjects who receive the vaccination with dosage deviation or who does not receive the second **and third** vaccination on schedule may not necessarily be withdrawn from the study as further study procedures and the follow-up visits may be performed which will be decided by sponsor.

7.4.5 Screen Failures

Screen failures are defined as participants who consent to participate in the clinical study but are not eligible randomly assigned to study intervention. **A minimal set of screen failure information is required demography, screen failure details, and eligibility criteria.**

Participants who do not meet the selection criteria in this study may be allowed to rescreened under different screen number.

7.4.6 Method of Assigning Subjects to Treatment Groups

The study vaccine and placebo will be randomly assigned to subjects. The randomized sequence of study treatments will be generated by an independent qualified biostatistician. The randomization schedule will be kept and maintained by the designated personnel before the study begins and until the database lock. The independent biostatistician prepares the randomization schedules via the SAS software (9.4 or higher version).

For core group, around 3850 participants will be randomly assigned to UB-612 vaccine 100 μg or placebo with a 6:1 randomization ratio (vaccine: placebo) by block randomization. In additional, a total of 966 evaluable subjects including 812 evaluable young adult and 154 evaluable elderly subjects from core group will be apportioned to immunogenicity and **lot-to-lot group** with a randomization ratio of 6:1 to UB-612 vaccine (696 evaluable young adult subjects and 132 evaluable elderly subjects) and placebo (116 evaluable young adult subjects and 22 evaluable elderly subjects). Furthermore, the 396 evaluable young adult subjects in immunogenicity group assigned to UB-612 vaccine will be allocated to 3 specified vaccine lots with a 1:1:1 ratio for evaluating the lot-to-lot consistency. Therefore, a total of 462 evaluable young adult subjects of immunogenicity group will be randomized to 3 vaccine lots and placebo with a ratio of 2:2:2:1 (vaccine lot 1: vaccine lot 2: vaccine lot 3: placebo).

In supplementary group, around 385 adolescent subjects aged from 12 to 18 years old will randomly receive UB-612 vaccine 100 μg or placebo with allocation rate 6:1, too.

Eligible subjects will receive a random number at randomization in form of a sealed envelope. The authorized unblinded dispenser(s)/administrator(s) who involve in the treatment of vaccine/ placebo will be required to open the envelope and administer the corresponding treatment assigned by the envelope. For any reason that a subject withdraws from the study prematurely after the randomization, his/her randomization number will not be reused. The next eligible subject will receive the lowest available randomization number.

7.4.7 Blinding

This is an observer-blind study, as the physical appearance of the investigational vaccine candidates and the placebo may differ. Only the dispenser(s)/administrator(s) will be unblinded. These study staffs are responsible for receiving, dispensing, preparing and administrating the study intervention. The investigational products should be administered in a manner that prevents the subjects to identifying their treatment based on vaccine physical appearance. The dispenser(s)/ administrator(s) should not participate in any of the study clinical evaluations or assay, and keep minimum contacts with other study personnel.

Neither the subjects nor the investigators, sponsor staffs, clinical monitors, study coordinators other than dispenser(s)/administrator(s), or other site staff who involve in clinical evaluation of the subjects will be aware of the treatment received either at the time of randomization or later, throughout the conduct of the trial. **These study personnel will be blinded until Visit 5. Unblinding will be carried out via disclosed Randomization Envelop during Visit 5.**

The treatment codes will be not prematurely broken unless an emergency situation, when the appropriate management of the subject necessitated knowledge of the treatment allocation, occurred. In the event of a medical emergency, if possible, the clinical monitor should be contacted before the treatment code blind is broken to discuss the need for unblinding. For unblinding a

subject, the treatment code blind can be obtained by the investigator, by opening the emergency envelope.

The sponsor must be notified immediately if the treatment code blind is broken. The date, time, and reason that the blind is broken must be recorded in the source document and the same information (except the time) must be recorded on the eCRF.

7.4.8 Removal of Subjects

7.4.8.1 Removal of Subjects from Immunogenicity Analysis

Subjects may stop study vaccine and withdraw from the immunogenicity analysis for any of the following reasons:

- Subjects do not receive **the first** 2 doses of UB-612 vaccine (refer to Section 7.4.4).
- Administration of prohibited medication/treatment/vaccine during pre-specified period which is enough to interfere with immunogenicity.

Subjects who do not comply with the protocol will be replaced. Subjects who stop study vaccine for any other reason (i.e., AE) will not be replaced.

7.4.8.2 Removal of Subjects from the Study

Subjects may withdraw from the study for any of the following reasons:

- Lost to follow-up
- Consent withdrawal
- Death
- Any pathological event, clinical adverse event, or any change in the subject’s status giving indication to the doctor that further participation in the study may not be the best interests of the subject, according to investigator’s discretion.
- Study terminated by sponsor

Subjects who withdraw consent will be replaced.

Subjects are free to withdraw from the study at any time without providing reason(s) for withdrawal and without prejudice to further research treatment. The reason(s) for withdrawal will be documented in the case report form (CRF).

Subjects withdrawing from the study, except subject death, will be encouraged to complete the same final evaluations (as Visit 5 procedure) within 7 days after withdrawal, as subjects completing the study according to this protocol, particularly safety evaluations. The aim is to record data in the same way as for subjects who completed the study.

Reasonable efforts will be made to contact subjects who are lost to follow-up (where possible, at least 3 telephone calls). These efforts must be documented in the subject’s file.

7.4.8.3 Study Termination

The sponsor has the right to terminate the study at any time in case of SAEs or if special

circumstances concerning the study agent or the company itself occur, making further research treatment of subjects impossible. In this event, the investigator(s) will be informed of the reason for study termination.

7.4.8.4 Reporting and Follow-up of Pregnancies

A positive urine pregnancy test should be confirmed by a serum pregnancy test. A negative pregnancy result is required before the subject may receive the study treatment. Subjects who become pregnant while on study must immediately discontinue study treatment. The pregnancy must be reported to sponsor within 24 hours of the investigator's or study site staff's acknowledgement of the pregnancy. Pregnancies for female subjects, or for the female partners of male subjects occurring during the study period, must be reported to the sponsor. Pregnancies should be handled and reported as AEs.

The investigator should inform the subjects of the risks of continuing with the pregnancy and the possible side effects to the fetus. The outcome of all pregnancies (spontaneous miscarriage, elective termination, normal birth, or congenital abnormality) must be followed up and documented even if the subject is discontinued from the study. If subject is pregnant **from first vaccination until 3 months after the last vaccination**, she will be encouraged to contact site and will be follow-up till delivery.

All reports of congenital abnormalities/birth defects and spontaneous miscarriages should be handled and reported as SAEs, to be reported within 24 hours of site awareness (regardless of interval since study treatment). Elective abortions should be handled and reported as AEs.

7.4.8.5 SARS-CoV-2 infection

Subjects encountered SARS-CoV-2 infection will not withdraw from the study, unless other withdrawal reason judged by investigators.

7.5 Investigational Products

7.5.1 Identity of Investigational Products

Name	UB-612	Saline Placebo
Characteristics & Physical State	**White to off-white suspension without foreign objects**	Transparent liquid
Formulated & Supplied by	UBI Pharma Inc.	Taiwan Biotech Co., LTD
Storage Conditions	Cooled (2℃ - 8℃) following receipt at site until the time of use	< 25℃
Shipments	Cooled (2℃ - 8℃)	< 25℃
Package	A disposable multi-dose vial containing 200 µg/mL UB-612 protein/peptide as the following:<br>- 200 µg/mL: 176 µg S1-RBD-sFc protein and 24 µg of six peptides/per 1 mL included	A plastic vial with 0.9% sodium chloride solution for injection as open-label supply will be provided.
Batch No.	**Lot #1: 303981**	**1MN2B022**

	**Lot #2: 304007**<br>**Lot #3: 304008**	

Each vial of 6.5 mL of UB-612 vaccine will be supplied in 10 mL glass vial. The vaccine lots used in this study have been tested and released by the quality control department of the UBI Pharma Inc.

7.5.2 Investigational Products Administered

7.5.2.1 Injection Volume(s)

	1st vaccination	2nd vaccination	**3rd vaccination**
Active vaccine group	UB-612 vaccine 100 µg, 0.5 mL	UB-612 vaccine 100 µg, 0.5 mL	**UB-612 vaccine 100 µg, 0.5 mL**
Placebo group	Normal saline 0.9%, 0.5 mL	Normal saline 0.9%, 0.5 mL	**N/A**

7.5.2.2 Administration

For this trial, the **first two doses** of UB-612 vaccine or saline placebo must be injected by an intramuscular (IM) route spaced 28 days apart. Injections on Day 1 and Day 29 will be given into non-dominant deltoid muscles, as detailed in Pharmacy Manual. **The 3rd dose of UB-612 will be given after unblinding once subjects qualified.**

7.5.2.3 Emergency Event Management

The subjects in each group will be closely monitored by the study staff at least 30 minutes after vaccination (for the change of vital signs or acute anaphylactic event). Subjects will be encouraged to quickly report any symptoms at the time during this period. The necessary rescue material, equipment, and appropriate medications will be available in the clinic to allow rapid intervention in case of anaphylaxis or other emergency.

7.5.3 Packaging and Labeling

The study packaging of UB-612 vaccine will be performed by UBI Pharma Inc.

All packaging and labeling operations will be performed according to Good Manufacturing Practice for Medicinal Products and the relevant regulatory requirements. The labels for the outer box/vial of the study vaccine contain the following information: the name/address/telephone number of UBI Pharma Inc., Product name, Study code, Indication, Package size, Dosage unit, Manufacture company, Batch No., Manufacture date, Expiration date, Storage conditions, Active ingredient concentration (only for UB-612 protein/peptide), Injection method, and words of caution stating the product is for investigational and clinical trial use only.

7.5.4 Prior and Concomitant Therapy

There is no specific known evidence of contraindications between the ingredients of UB-612 vaccine and other prior and concomitant therapy. Concomitant medications and therapies will be recorded beginning 1 month prior to 1st vaccination, as well as during study period.

7.5.4.1 Prohibited Medication/Therapy

The following medications or treatments which may affect the immunogenicity and clinical efficacy assessments will be prohibited in subjects who assess immunogenicity:

- Immunosuppressant, cytotoxic treatment until Day 197
- Immunoglobulins and/or any blood products until Day 197
- Systemic corticosteroids (≥ 20 mg/day of prednisone or equivalent) until Day 197
- Investigational product (including drug, vaccine) during study period
- Any seasonal or pandemic influenza vaccine within 14 days, or any other nonstudy vaccine within 28 days, after study intervention administration. Licensed COVID-19 vaccine is prohibited through the whole study period.

The following medications or treatments which may affect the immunogenicity and clinical efficacy assessments will be prohibited in subjects who assess safety only:

- Immunosuppressant, cytotoxic treatment until Day 57
- Immunoglobulins and/or any blood products until Day 57
- Systemic corticosteroids (≥ 20 mg/day of prednisone or equivalent) until Day 57
- Investigational product (including drug, vaccine) during study period
- Any seasonal or pandemic influenza vaccine within 14 days, or any other nonstudy vaccine within 28 days, after study intervention administration. Licensed COVID-19 vaccine is prohibited through the whole study period.

8 TIMING OF STUDY PROCEDURES

8.1 Study Procedures

8.1.1 Visit 1 (Day -28~-1 ± 3 days) –Screening

(1) Record date of informed consent will be signed. The following should be documented in the subject's medical chart: that they are participating in this study that informed consent has been obtained and that a copy of the consent has been given to the subject.

(2) Assign screen number

(3) Eligibility: Assess against the inclusion and exclusion criteria

(4) Demographics

(5) Medical history and concurrent diseases.

(6) Conduct physical exam including the measurement of weight and height

(7) Measure vital signs

(8) Perform 12 lead ECG

(9) Collect urine sample for urinalysis

(10) Collect blood sample for following tests:

- ➢ Blood routine tests
- ➢ Biochemistry tests
- ➢ Immunology test
- ➢ **Spared sample (tests for detecting serum antibody against SARS-CoV-2 or further researches)**

8.1.2 Visit 2 (Day 1) –Baseline: 1st Vaccination

(1) Recheck eligibility: Assess against the inclusion and exclusion criteria

(2) Randomization; assign randomization number

(3) Medical history and concurrent diseases. Any pre-vaccination medical event will be recorded as medical history since last visit.

(4) Conduct physical exam

(5) Measure vital signs

(6) Perform a urine pregnancy test.

(7) If subject encountered ≥ Grade 3 hypertension, subject should be checked the existence or deterioration of proteinuria or other renal damage sign.

(8) Collect blood sample before vaccination for following tests only for subjects in lot-to-lot consistency /immunogenicity group:

- ➢ Immune response assessments

  - T cell function (optional)

(9) Perform 1st vaccination by unblinded administrator.

(10) Observe closely during vaccination and at least 30 minutes after vaccination in each group (for the change of vital signs or acute anaphylactic event) by blinded site staff.

(11) Instruct the subject to monitor body temperature and complete self-evaluation e-diary correctly.

Subject will be instructed to record any solicited adverse events occurring during a 7-day post-vaccination period on the e-diary system. Meanwhile, skin allergic reaction should be monitored for 14 days via e-diary. If the subject perceives any signs or symptoms are progressing or serious, contact the investigator or their delegates immediately. Additional return visits can be scheduled by the investigators when necessary.

(12) Review concomitant medications.

8.1.3 Day 8, 15, and 22 –Safety calls

Safety calls on Day 8, Day 15 and Day 22 to monitor subjects' unsolicited AEs and symptoms of COVID-19. Calls on Day 8 and Day 15 should be inquire the phenomenon of allergic skin reaction, especially itching and redness, or other unsolicited allergy reaction. No matter injection site or extending to other body site, site staff should arrange unscheduled visit to verify the allergic reaction and check HLA typing if indicated.

8.1.4 Visit 3 (Day 29 ± 3 day) –2nd Vaccination

(1) Check contraindication for second vaccination and the possibility of delay vaccination

(2) Conduct physical examination.

(3) Measure vital signs.

(4) Perform a urine pregnancy test.

(5) If subject encountered ≥ Grade 3 hypertension, subject should be checked the existence or deterioration of proteinuria or other renal damage sign.

(6) Collect blood sample before vaccination for following tests only for subjects in lot-to-lot consistency/immunogenicity only:

  - Anti-RBD-S1 antibody

(7) Perform 2nd vaccination by unblinded administrator.

(8) Observe closely during vaccination and at least 30 minutes after vaccine administration for the change of vital sign or acute anaphylactic event at site by blinded site staff.

(9) Instruct the subject to monitor body temperature and complete self-evaluation e-diary correctly.

Subject will be instructed to record any solicited adverse events occurring during a 7-day post-vaccination period on the e-diary system. Meanwhile, skin allergic reaction should be monitored for 14 days via e-diary. If the subject perceives any signs or

symptoms are progressing or serious, contact the investigator or their delegates immediately. Additional return visits can be scheduled by the investigators when necessary.

(10) Review concomitant medications.

(11) Record and report adverse event, serious adverse event, or any symptoms of COVID-19 if any has occurred since the previous visit.

8.1.5 Day 36 and 43 –Safety calls

Safety calls on Day 36 and 43 to monitor subjects' unsolicited AEs and symptoms of COVID-19.

8.1.6 Visit 4 (Day 57 ± 3 day) –Follow-up

(1) Conduct physical examination.

(2) Measure vital signs.

(3) If subject encountered ≥ Grade 3 hypertension, subject should be checked the existence or deterioration of proteinuria or other renal damage sign.

(4) Collect blood sample for following tests for subjects in lot-to-lot consistency /immunogenicity group:

- Immune response assessments
- T cell function (optional)

(5) Collect blood sample for following tests for all subjects:

- Blood routine tests
- Biochemistry test
- Immunology test
- **Spared sample (tests for detecting serum antibody against SARS-CoV-2 or further researches)**

(6) Review concomitant medications.

(7) Record and report adverse event, serious adverse event, or any symptoms of COVID-19 if any has occurred since the previous visit.

After Day 57, each week (i.e., every 7 days) the subject will receive a prompt on their smartphone device to regularly surveillance for signs and symptoms of COVID-19.

8.1.7 Day 64, Day 71, Day 78, and Day 85 –Safety calls

Safety calls on Day 64, Day 71, Day 78, and Day 85 to monitor subjects' unsolicited AEs and symptoms of COVID-19.

8.1.8 Visit 5 (Day 197 ± 15 days) –Unblinding / Early Termination

(1) Conduct physical examination.

(2) Measure vital signs.

(3) If subject encountered ≥ Grade 3 hypertension, subject should be checked the existence or deterioration of proteinuria or other renal damage sign.

(4) **Unblinding**

(5) **Withdraw subjects who received placebo**

(6) Collect blood sample for following tests only for subjects in lot-to-lot consistency /immunogenicity group:

- ➢ Immune response assessments

(7) Review concomitant medications.

(8) Record and report adverse event, serious adverse event, or any symptoms of COVID-19 if any has occurred since the previous visit.

The subject will receive a prompt on their smartphone device to regularly surveillance for signs and symptoms of COVID-19 each week.

8.1.9 Visit 6 (Day 197~242) –3rd Vaccination

The following procedures will only be performed on subjects who agreed to have 3rd vaccination. The Visit 5 and Visit 6 could be the same day.

(1) Collect inform consent form for vaccine group who agreed to have 3rd vaccination.

(2) Check contraindication for the third vaccination and the possibility of delay vaccination

(3) Conduct physical examination.

(4) Measure vital signs.

(5) Perform a urine pregnancy test.

(6) If subject encountered ≥ Grade 3 hypertension, subject should be checked the existence or deterioration of proteinuria or other renal damage sign.

(7) Collect blood sample for following tests:

- ➢ **Immune response assessments**
- ➢ **Blood routine tests**
- ➢ **Biochemistry test**
- ➢ **Immunology test**
- ➢ **T cell function (optional)**

(8) Perform 3rd vaccination

(9) Observe closely during vaccination and at least 30 minutes after vaccine administration for the change of vital sign or acute anaphylactic event at site by site staff.

(10) Instruct the subject to monitor body temperature and complete self-evaluation e-diary correctly.

(11) Review concomitant medications.

(12) Conduct COVID-19 surveillance

(13) Record and report adverse event, serious adverse event, or any symptoms of COVID-19 if any has occurred since the previous visit.

8.1.10 Safety call on 7 days after Visit 6 (Only for subjects who have 3rd vaccination)

Safety calls on 7 days after Visit 6 to monitor subjects' unsolicited AEs and symptoms of COVID-19. Call should be inquired the phenomenon of allergic skin reaction, especially itching and redness, or other unsolicited allergy reaction. No matter injection site or extending to other body site, site staff should arrange unscheduled visit to verify the allergic reaction and check HLA typing if indicated.

8.1.11 Follow up on 14 days after Visit 6 (± 3 day) (Only for subjects who have 3rd vaccination)

(1) Conduct physical examination.

(2) Measure vital signs.

(3) If subject encountered ≥ Grade 3 hypertension, subject should be checked the existence or deterioration of proteinuria or other renal damage sign.

(4) Collect blood sample for following tests:

- **Immune response assessments**
- **Blood routine tests**
- **Biochemistry test**
- **Immunology test**
- **T cell function (optional)**

(5) Review concomitant medications.

(6) Conduct COVID-19 surveillance

(7) Record and report adverse or serious adverse event.

8.1.12 Day 253 and Day 309- Bi-monthly Safety calls

After Day 197, subjects will enter the **long-term follow-up** with a safety call bi-monthly on Day 253 and Day 309 to monitor subjects' unsolicited AEs and symptoms of COVID-19.

8.1.13 Long-term follow-up (Day 365 ± 45 days)

(1) Measure vital signs.

(2) If subject encountered ≥ Grade 3 hypertension, subject should be checked the existence or deterioration of proteinuria or other renal damage sign.

(3) Collect blood sample for following tests only for subjects **have 2 vaccinations** in lot-to-lot consistency /immunogenicity group:

- ➢ Immune response assessments

(4) **Collect blood sample for following tests only for subjects have 3rd vaccination:**

- ➢ **Immune response assessments**

(5) Record adverse events, including AESI, MAAE, SAE, or any symptoms of COVID-19 if any has occurred since the previous visit.

8.2 Early Termination (ET)

For subjects in lot-to-lot consistency/immunogenicity group who discontinue this study earlier, a final follow-up may be arranged not **later** than 7 days and all study procedures listed for Visit 5 should be completed.

8.3 Unscheduled Visit

Subjects who suffer from Grade 3/4 solicited AE should return to site ASAP for further survey and treatment. All AEs recognised during the unscheduled visits are medically attended adverse events (MAAE), except for confirmed COVID-19 disease.

- If a Grade 3 local reaction, systemic event, or fever is reported in the reactogenicity e-diary, a telephone contact should occur to ascertain further details and determine whether a site visit is clinically indicated.
- If suspected Grade 4 local reaction systemic event or fever is reported in the reactogenicity e-diary, a site visit should occur to confirm whether the event meets the criteria for Grade 4.

8.4 COVID-19 Surveillance (All Subjects)

If a subject experiences any of the following (irrespective of perceived etiology or clinical significance), he or she is instructed to contact the site immediately. During the 7 days following each vaccination, potential COVID-19 symptoms that overlap with solicited systemic events (i.e. fever, chills, new or increased muscle pain, diarrhea, vomiting) should not trigger a potential COVID-19 illness visit unless, in the investigator's opinion, the event is more indicative of a possible COVID-19 illness than vaccine reactogenicity.

8.4.1 Taiwan CDC Criteria for Case Definition of COVID-19

**Criteria**	**Definition**
**Clinical Presentation Criteria**	One or more of the following:<br>(1) Fever (≥ 38 °C) or symptoms of acute respiratory tract infection.<br>(2) Abnormal sense of smell (anosmia), abnormal sense of taste (dysgeusia), or diarrhea of unknown etiology.<br>(3) .Community-acquired pneumonia (CAP) highly suspected to be COVID-19 by doctors
**Laboratory Diagnosis Criteria**	One or more of the following:<br>(1) Pathogen (SARS-CoV-2) isolated and identified from a clinical specimen (nasopharyngeal swab, throat swab,

	expectorated sputum, or lower respiratory tract aspirates).<br>(2) Positive molecular biological testing for viral (SARS-CoV-2) RNA from a clinical specimen (nasopharyngeal swab, throat swab, expectorated sputum, or lower respiratory tract aspirates).
**Epidemiological Criteria**	One or more of the following within 14 days prior to symptom onset:<br>(1) History of traveling or living abroad, or contact with symptomatic (fever or other respiratory tract infection symptoms) individuals returning from abroad.<br>(2) History of close contact with symptomatic suspected or confirmed case(s), including caring for or interacting with these individuals, or direct contact with body fluid or respiratory secretions without adequate personal protective equipment (PPE).<br>(3) History of cluster related to confirmed cases.
**Reporting Requirements for COVID—19**	Any cases with one or more of the following conditions should be reported to the Taiwan Centers for Disease Control (Taiwan CDC):<br>(1) Meet clinical presentation criteria (1) **AND** one or more epidemiological criteria.<br>(2) Meet clinical presentation criteria (2) **AND** any of epidemiological criteria (1)) or (2).<br>(3) Meet clinical presentation criteria (3).<br>(4) Meet laboratory diagnosis criteria.
**Case Definition**	(1) Suspected case: meet clinical presentation criteria but not laboratory proven, plus history of close contact with symptomatic confirmed case(s) within 14 days prior to symptom onset.<br>(2) Confirmed case: meet laboratory diagnosis criteria, regardless. of clinical signs and symptoms.

8.4.2 Intercurrent COVID-19

If, at any time, a subject develops acute respiratory illness, for the purposes of the study she will be considered to potentially have COVID-19 illness. **In this circumstance, the subject should contact the site, an in-person or telemedicine contact should occur, and assessments should be conducted, as appropriate.** The assessments will include a clinical specimen defined above, which will be tested at a laboratory using an TFDA-approved polymerase chain reaction (PCR) test to detect SARS-CoV-2.

Taiwan CDC Criteria for case definition of COVID-19 is described in Section 8.4.1, and subjects who fulfill the reporting criteria should report to CDC. The diagram for COVID-19 surveillance is as below:

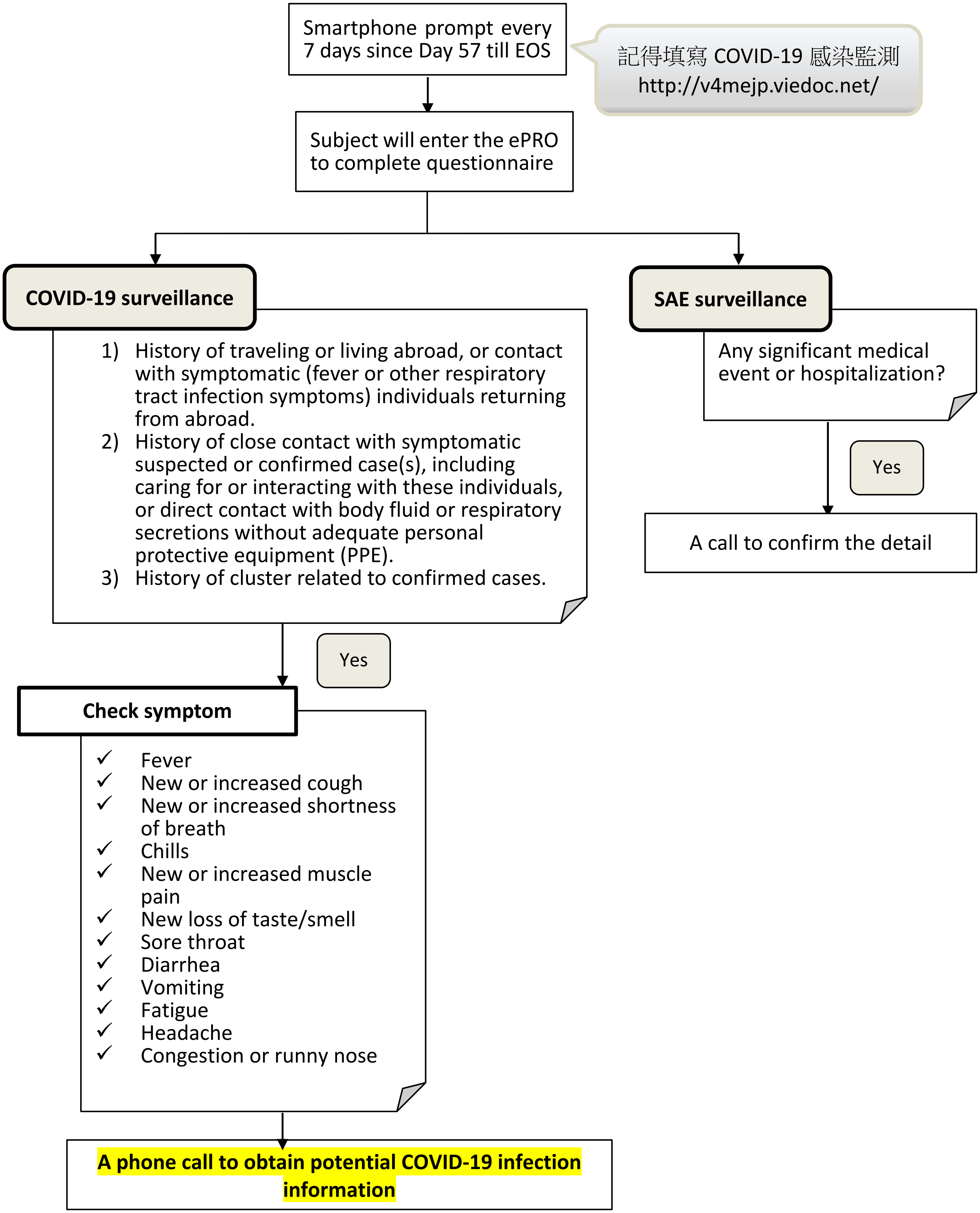
Smartphone prompt every 7 days since Day 57 till EOS
記得填寫 COVID-19 感染監測
http://v4mejp.viedoc.net/
Subject will enter the ePRO to complete questionnaire
COVID-19 surveillance
1) History of traveling or living abroad, or contact with symptomatic (fever or other respiratory tract infection symptoms) individuals returning from abroad.
2) History of close contact with symptomatic suspected or confirmed case(s), including caring for or interacting with these individuals, or direct contact with body fluid or respiratory secretions without adequate personal protective equipment (PPE).
3) History of cluster related to confirmed cases.
Yes
Check symptom
✓ Fever
✓ New or increased cough
✓ New or increased shortness of breath
✓ Chills
✓ New or increased muscle pain
✓ New loss of taste/smell
✓ Sore throat
✓ Diarrhea
✓ Vomiting
✓ Fatigue
✓ Headache
✓ Congestion or runny nose
A phone call to obtain potential COVID-19 infection information
SAE surveillance
Any significant medical event or hospitalization?
Yes
A call to confirm the detail

8.4.3 Potential COVID-19 Infection

Through ePRO system, the potential COVID-19 infection will be monitored. Once the events occur, the system alert will be sent to notice the study team members. The in-person or telemedicine contact will be conducted, optimally within 3 days after onset of illness. Several contacts may be required to obtain the following information:

- **Record potential COVID-19 illnesses, as appropriate. Note: Potential COVID-19 illnesses that are consistent with the clinical endpoint definition will be recorded on a COVID-19 CRF and will not be listed as AEs.**
- **Record the results of COVID-19 testing by an authorities approved test, if available**
- **Schedule an appointment for the subject to return for the potential COVID-19 convalescent visit once he or she has recovered.**
- **The investigator or designee completes the CRFs on COVID-19 page.**

8.4.4 COVID-19 Convalescent Visit

This visit may be conducted 28 to 35 days after the Potential COVID-19 Illness Visit.

- Record AEs, as appropriate.

  Note: Potential COVID-19 illnesses that are consistent with the clinical endpoint definition should not be recorded as AEs. These data will be captured as efficacy assessment data only on the relevant pages of the CRF, as these are expected endpoints.
- Record details of any of the prohibited medications specified in Section 7.5.4.1 received by the subject if required for his or her clinical care.
- **Collect blood samples for immunogenicity testing:**
  - **UBI SARS-CoV-2 ELISA, IgG S1-RBD ELISA, ACE2 binding inhibition ELISA and neutralization tests (~20 mL)**
- Collect/update COVID-19–related clinical and laboratory information
- Complete the source documents
- The investigator or an authorized designee completes the CRFs on COVID-19 page.

8.5 Duration of Treatment

The duration of each visit is expected to last between 2 to 3 hours, barring any unexpected adverse reactions.

It takes about 13 months for each subject to participate in the study, from recruiting to the last visit. Some subjects may withdraw the study during the course of the study.

9 IMMUNOGENICITY AND SAFETY VARIABLES

The planned schedule of assessments is in Section 7.2.

9.1 Informed Consent Form

The investigator or designee must explain to each subject the nature of the study, its purpose, the procedures involved, the expected duration, the potential risks and benefits involved and any potential adverse events. Each subject will be informed that participation in the study is voluntary and that they can be withdrawn from participation at any time.

All subjects must provide a signed and dated informed consent at Visit 1. An informed consent form must be approved by the Institutional Review Board (IRB), Ethics Committee (EC), and/or the applicable health authorities.

9.2 Demographics / Other Baseline Characteristics

The demographic and baseline characteristic data for subjects will be collected at Visit 1. The demographics include date of birth, age, sex and ethnicity. Relevant history/conditions include all those present prior to the administration of study vaccine that are listed below:

- Relevant medical history,
- All current medical conditions,
- Allergy history,

Whenever possible, diagnoses but not symptoms should be recorded.

9.3 Eligibility

Eligibility should be checked by the investigator at Visit 1 and rechecked at Visit 2 before vaccination, these 2 visits could be emerged as one. **For subjects agreed to receive 3rd vaccination, eligibility should be rechecked at Visit 6.**

9.4 Administration

The unblinded staff takes a vial of investigational vaccine/placebo, use a disposable syringe to extract vaccine/placebo and intramuscularly inject it into the middle of the lateral deltoid muscle of the subject's upper arm.

Vaccine administration will be recorded. The containers from which the vaccine is administered to the subjects will be retained for confirmation.

9.5 Safety and Immunogenicity Measurements Assessed

9.5.1 Safety Variables

9.5.1.1 Physical Examination

Complete physical examinations should be conducted by investigator/site staff at all visits. A complete physical exam will include the examination of general appearance, HEENT (head, ears, eyes, nose, throat), neck (including thyroid), lymph nodes, skin, cardiovascular, pulmonary, abdomen, neurological system and musculoskeletal/joints.

Body weight and height will be collected at Visit 1. Body weight will be measured in indoor clothing, but without shoes and blanket to the nearest 0.1 kilogram (kg). Body height will be measured in centimeters (cm).

It must be recorded when any abnormality has been found out.

9.5.1.2 Vital Sign

Systolic/diastolic blood pressure, pulse, respiratory rate, and ear temperature will be collected at all visits.

For two vaccination visits, vital sign should be measured prior to and after vaccine administration. All subjects in each group will be closely monitored by the study staff at pre-vaccination and 30 minutes after vaccination (at least) and the results will be recorded in CRF.

9.5.2 Electrocardiogram(ECG)

A 12-lead electrocardiogram (ECG) will be performed by the investigator or a suitable qualified designee after measurement of vital signs. The ECG measurement should be obtained after the subject has been seated and at rest for at least 5 minutes. The ECGs will be performed at screening visit.

Qualified physicians will interpret the ECG. Any abnormal ECG reading should be noted and recorded in the CRF.

9.5.3 Clinical Laboratory Evaluation

The clinical laboratory analyses will be performed at central laboratory designated by sponsor. Reference ranges will be supplied by the central laboratory and used by the investigator to assess the laboratory data for clinical significance and pathological changes.

Methods and timing for assessing, recording and analyzing each laboratory variable should follow local guidelines. The following laboratory safety tests will be performed:

Blood Routine

CBC, including Hgb, Hct, RBC, WBC with differential, platelet count.

Biochemistry

Creatinine, ALT, AST, total and direct bilirubin; high sensitivity (hs) CRP,

Immunology

Anti-nuclear antibody (ANA)

Urinalysis

Color, **pH,** specific gravity, appearance, nitrate, blood, glucose, ketones, protein, leukocyte esterase, WBC, RBC, casts, epithelial cells, yeast, bacteria

Proteinuria will be checked via urine routine as baseline on Visit 1. **If subject encountered ≥ Grade 3 hypertension, the existence or deterioration of proteinuria or other renal damage sign will be checked.**

9.5.4 Pregnancy Test

Screening for urine pregnancy, using pregnancy strip, will be performed on Day 1 and on Day 29 before vaccination for WoCBP only. It is not required for postmenopausal or surgically sterilized women.

A positive urine pregnancy test should be confirmed by a serum test. Pregnancy tests may be performed more frequently per request of Institutional Review Board (IRB)/Independent Ethics Committee (IEC) or if required by local regulations.

9.5.5 Self-Evaluation/Reporting (Solicited/Unsolicited Symptoms)

9.5.5.1 Solicited Symptoms

Information of solicited symptoms and body temperature will be collected by the subjects in the provided e-diary cards during a 7-day follow-up period after each vaccination (i.e. day of vaccination and 6 subsequent days), and reported by the investigator team. Meanwhile, skin allergic reaction should be monitored for 14 days via e diary. The subject should complete the assessments in the e-diary every evening.

Local reaction, skin allergic reaction, and systemic event are the one whose nature or intensity is consistent with the expected AEs described and listed below.

Table 9-1 Grading for local reaction

**Local Reaction**	**Mild (Grade 1)**	**Moderate (Grade 2)**	**Severe (Grade 3)**	**Potentially Life Threatening (Grade 4)**
Pain at injection site	Does not interfere with activity	Interferes with activity	Prevents daily activity	Emergency room visit or hospitalization for severe pain
Itching at injection site	Does not interfere with activity	Interferes with activity	Prevents daily activity	**Emergency room visit or hospitalization for severe symptoms**
Redness	>2.0 to 5.0 cm (5 to 10 measuring device units)	>5.0 to 10.0 cm (11 to 20 measuring device units)	>10 cm (≥21 measuring device units)	Necrosis or exfoliative dermatitis
Swelling	>2.0 to 5.0 cm (5 to 10 measuring device units)	>5.0 to 10.0 cm (11 to 20 measuring device units)	>10 cm (≥21 measuring device units)	Necrosis or exfoliative dermatitis

Table 9-2 Grading for allergic skin reaction

Local Reaction	Mild (Grade 1)	Moderate (Grade 2)	Severe (Grade 3)	Potentially Life Threatening (Grade 4)
Skin allergic reaction	Localized urticaria (wheals) with no medical intervention indicated	Localized urticaria with intervention indicated OR Mild angioedema with no medical intervention indicated	Generalized urticarial OR Angioedema with intervention indicated	Anaphylaxis

Table 9-3 Grading for systemic events

Systemic Event	Mild (Grade 1)	Moderate (Grade 2)	Severe (Grade 3)	Potentially Life Threatening (Grade 4)
Subjective fever	Does not interfere with activity	Some interference with activity	Prevents daily routine activity	**Emergency room visit or hospitalization for severe symptoms**
Chills	Does not interfere with activity	Some interference with activity	Prevents daily routine activity	Emergency room visit or hospitalization for severe chills
Nausea/anorexia	Does not interfere with activity	Some interference with activity	Prevents daily routine activity	**Emergency room visit or hospitalization for severe symptoms**
Vomiting	1 to 2 times in 24 hours	>2 times in 24 hours	Requires intravenous hydration	Emergency room visit or hospitalization for hypotensive shock
Diarrhea	2 to 3 loose stools in 24 hours	4 to 5 loose stools in 24 hours	6 or more loose stools in 24 hours	Emergency room visit or hospitalization for severe diarrhea
Headache	Does not interfere with activity	Some interference with activity	Prevents daily routine activity	Emergency room visit or hospitalization for severe headache
Fatigue/tiredness	Does not interfere with activity	Some interference with activity	Prevents daily routine activity	Emergency room visit or hospitalization for severe fatigue
Muscle pain	Does not interfere with activity	Some interference with activity	Prevents daily routine activity	Emergency room visit or hospitalization for severe new or

Systemic Event	Mild (Grade 1)	Moderate (Grade 2)	Severe (Grade 3)	Potentially Life Threatening (Grade 4)
				worsened muscle pain
Joint pain	Does not interfere with activity	Some interference with activity	Prevents daily routine activity	Emergency room visit or hospitalization for severe new or worsened joint pain

The axillary temperature will be monitored every evening on day of vaccination and 6 subsequent days. If axillary temperature has been measured for more than one time, only the highest degree level should be recorded in the e-diary card.

Table 9-4 Grading for axillary temperature

	Mild (Grade 1)	Moderate (Grade 2)	Severe (Grade 3)	Potentially Life Threatening (Grade 4)
Fever (℃)	37.3～＜38.0	38.0～＜38.5	38.5～＜39.5	≥39.5, last more than 3 days

9.5.5.2 Unsolicited Adverse Event

An unsolicited local and general AE or symptoms are the one whose nature or intensity is NOT consistent with the expected AEs described and listed above in this protocol. Information of unsolicited symptoms/AEs will be collected during study period and reported at each visit or safety calls.

9.5.5.3 Time Period and Frequency for Collecting AE and SAE Information

The time period for actively eliciting and collecting AEs and SAEs ("active collection period") for each participant begins from the time the participant receiving vaccination. All AEs, including AESI, MAAE and SAE, will be collected during the study period.

Follow-up by the investigator continues throughout and after the active collection period and until the AE or SAE or its sequelae resolves or stabilizes at a level acceptable to the investigator.

If the participant withdraws from the study and also withdraws consent for the collection of future information, the active collection period ends when consent is withdrawn.

If a participant definitively discontinues or temporarily discontinues study intervention because of an AE or SAE, the AE or SAE must be recorded on the CRF and the SAE reported using the SAE reporting form.

9.5.6 Adverse events (AEs)

9.5.6.1 Definition of Adverse Events (AE)

An AE is any untoward medical occurrence in a patient or clinical investigation subject temporally associated with the use of a medicinal product, whether or not considered related to the medicinal product. The occurrence does not necessarily have to have a causal relationship with this treatment. An AE can therefore be any unfavorable and unintended sign (including an abnormal laboratory finding), symptom, or disease (new or exacerbated) temporally associated with the use of a medicinal product, whether or not considered related to the medicinal product.

Examples of an AE include:

- Exacerbation of a chronic or intermittent pre-existing condition, including either an increase in frequency and/or intensity of the condition
- **A new condition detected or diagnosed, even though it may have been present prior to this**
- Signs, symptoms, or the clinical sequelae of a suspected interaction
- Signs, symptoms, or the clinical sequelae of a suspected overdose of either study drug or a concurrent medication.

Examples of an AE do NOT include:

- A medical or surgical procedure (*e.g.* endoscopy); the condition that leads to the procedure is an AE
- Situations where an untoward medical occurrence did not occur (social and/or convenience admission to hospital)
- Anticipated day-to-day fluctuations of pre-existing disease(s) or condition(s) present or detected before informed consent is obtained, that do not worsen.

A priori, immunogenicity endpoints as specified in the protocol will not be considered as AEs except if, because of the course or severity or any other features of such events, the investigator, according to his/her best medical judgment, considers these events as exceptional in this medical condition.

9.5.6.2 Definition of Serious Adverse Events (SAE)

A SAE is any untoward medical occurrence that at any dose:

- Results in death or,
- Is life-threatening or,

  Note: The term "life-threatening" in the definition of "serious" refers to an event in which the subject was at risk of death at the time of the event; it does not refer to an event, which hypothetically might have caused death if it were more severe.

- Requires inpatient hospitalization or prolongation of existing hospitalization or,

  Note: In general, hospitalization signifies that the subject has been detained (usually involving at least an overnight stay) at the hospital or emergency ward for observation and/or treatment that would not have been appropriate in the physician's office or out-subject setting. Complications that occur during hospitalization are AEs. If a complication prolongs

hospitalization or fulfils any other serious criteria, the event is serious. When in doubt as to whether hospitalization occurred or was necessary, the AE should be considered serious. Hospitalization for elective treatment of a pre-existing condition that did not worsen from baseline is not considered an AE.

- Results in persistent or significant disability/incapacity or,

  Note: The term disability means a substantial disruption of a person's ability to conduct normal life functions. This definition is not intended to include experiences of relatively minor medical significance such as uncomplicated headache, nausea, vomiting, diarrhoea, influenza, and accidental trauma (e.g. sprained ankle), which may interfere or prevent everyday life functions but do not constitute a substantial disruption.

- Is a congenital anomaly/birth defect or,
- Is a medically important event

  Note: Medical and scientific judgment should be exercised in deciding whether expedited reporting is appropriate in other situations, such as important medical events that may not be immediately life threatening or result in death or hospitalization but may jeopardize the subject or may require intervention to prevent one of the other outcomes listed in the definition above. These should also be considered serious. Examples of such events are invasive or malignant cancers, intensive treatment in an emergency room or at home for allergic bronchospasm, blood dyscrasias or convulsions that do not result in hospitalisation, or development of drug dependency or drug abuse.

An AE fulfilling any one or more of these criteria should be reported as a SAE, irrespective of the dose of drug given, and even if it is the result of an interaction or drug abuse.

A distinction should be drawn between serious and severe AEs. The term 'severe' is often used to describe the intensity (severity) of a specific event (as in mild, moderate, or severe myocardial infarction); the event itself, however, may be of relatively minor medical significance (such as severe headache). This is not the same as 'serious,' which is based on subject's event outcome or action criteria usually associated with events that pose a threat to a subject's life or functioning. The seriousness (not severity) serves as a guide for defining regulatory reporting obligations.

9.5.6.3 Definition of Unexpected Adverse Reaction Definition

An unexpected adverse reaction is any untoward and unintended response that is related to the administration of the study agent, at any dose that is not consistent with the applicable product information (e.g., current version of the Investigator's Brochure for an unauthorized investigational medicinal product or summary of product characteristics for an authorized product).

9.5.6.4 Definition of Medically Attended Adverse Event

A Medically Attended Adverse Event (MAAE) is an AE with a medically-attended visit that is not a routine visit for physical examination or vaccination, such as visits for hospitalization, an emergency room visit, or an otherwise unscheduled visit to or from medical personnel (medical doctor) for any reason.

9.5.6.5 Definition of Adverse Events of Special Interest (AESI)

Adverse event of special interest (AESI) is further defined in Council for International Organizations of Medical Sciences (CIOMS) VII as [15]:

An adverse event of special interest (serious or non-serious) is one of scientific and medical concern specific to the sponsor's product or specific target disease, for which ongoing monitoring and rapid communication by the investigator to the sponsor could be appropriate. Such an event might require further investigation in order to characterize and understand it. Depending on the nature of the event, rapid communication by the trial sponsor to other parties (e.g., regulators) might also be warranted. Thus AESI should include potential immune medical condition (listed in **Table 9-6**), or any newly identified potential AESI followed through 12 months after participants' final vaccination. Complications of COVID-19 (listed in **Table 9-5**) should be considered and reported as AESIs.

Subjects will be assessed for diagnosis of an AESI at all visit. AESIs include potential ADE medical conditions, AE specific to complications of COVID-19, or other potential AEs that may be determined at any time by regulatory authorities as additional information concerning COVID-19 is obtained. Given the concern for "cytokine storm", an AESI of cytokine release syndrome will be included as an AE specific to COVID-19. Thus AESI included

AESIs specific to complications of potential disease enhancement for COVID-19, also termed ADE (antibody dependent enhancement), will also be monitored (see **Table 9-5**).

Table 9-5 Adverse Events of Special Interest Relevant to COVID-19[a]

Body System	Diagnoses[a]
Immunologic	Enhanced disease following immunisation,[b] cytokine release syndrome related to COVID-19[c], Multisystem inflammatory syndrome in children (MIS-C)
Respiratory	Acute respiratory distress syndrome (ARDS)
Cardiac	Acute cardiac injury including:<br>• Microangiopathy<br>• Heart failure and cardiogenic shock<br>• Stress cardiomyopathy<br>• Coronary artery disease<br>• Arrhythmia<br>• Myocarditis, pericarditis
Hematologic	Coagulation disorder<br>• Deep vein thrombosis<br>• Pulmonary embolus<br>• Cerebrovascular stroke<br>• Limb ischemia<br>• Hemorrhagic disease<br>• Thrombotic complications
Renal	Acute kidney injury
Gastrointestinal	Liver injury
Neurologic	Guillain-Barré Syndrome, anosmia, ageusia, meningoencephalitis
Dermatologic	Chilblain-like lesions, single organ cutaneous vasculitis, erythema multiforme

Abbreviations: AESI = adverse event of special interest; COVID-19 = coronavirus disease 2019; DAIDS = Division of AIDS; PCR = polymerase chain reaction; SARS-CoV2 = severe acute respiratory syndrome coronavirus 2.

[a] To be recorded as AESIs relevant to COVID-19, these complications should be associated with a positive PCR test for SARS-CoV-2.

[b] COVID-19 manifestations associated with more severe presentation and decompensation with consideration of enhanced disease potential.

[c] Cytokine release syndrome related to COVID-19 infection is a disorder characterised by nausea, headache, tachycardia, hypotension, rash, and/or shortness of breath.

Because it has been hypothesized that immunizations with or without adjuvant may be associated with autoimmunity, regulatory authorities have requested that sponsor instruct investigators to be especially vigilant regarding the PIMMC listed below (**Table 9-6**). Subjects encountered the medical condition of AESI listed in **Table 9-5 and 9-6** should check HLA and SARS-CoV-2 infection,

Table 9-6 Potential Immune-Mediated Medical Conditions (PIMMC)

**Categories**	**Diagnoses (as MedDRA Preferred Terms)**
Neuro-inflammatory Disroders	Acute disseminated encephalomyelitis (including site specific variants: e.g. Non-infectious encephalitis, encephalomyelitis, myelitis, myeloradiculomyelitis), cranial nerve disorders including paralyses/paresis (e.g. Bell’s palsy), generalized convulsion, Guillain-Barré syndrome (including Miller Fisher syndrome and other variants), immune-mediated peripheral neuropathies and plexopathies (including chronic inflammatory demyelination polyneuropathy, multi focal motor neuropathy and polyneurapthies associated with monoclonal gammopathy), myasthenia graves, multiple sclerosis, nacrolepsy, optic neuritis, transverse myelitis, uveitis.
Musculoskeletal and Connective Tissue Disorder	Antisynthetase syndrome, dermatomyositis, juvenile chronic arthritis (including Still’s disease), mixed connective tissue disorder, poly myalgia rheumatic, polymyositis, psoriatic arthropod hypertension, relapsing polychondritis, rheumatoid arthritis, scleroderma (including diffuse systemic form and CREST syndrome), spondyloarthritis (including ankylosis spondylitis, reactive arthritis (Reiter’s Syndrome) and undifferentiated spondyloarthritis), systemic lupus erythematosus, systemic sclerosis, Sjogren’s syndrome.
Vasculitides	Large vessels vasculitis (including giant cell arthritis such as Takayasu’s arthritis and temporal arthritis), medium sized and /or small vessels vasculitis (including poly arthritis no dose, Kawasaki’s disease, microscopic polyangitis, Wegener’s granule atoms is, Churg-Strauss syndrome (allergic granulomatous angiitis), Buerger’s disease (thromboangiitis obliterans), necrotizing vasculitis and ANCA-positive vasculitis (type unspecified), Henoch-Schonlein purpura, Bechet’s syndrome, leukocytoclastic vasculitis)
Gastrointestinal Disorders	Crohn’s disease, celiac disease, ulcerative colitis, ulcerative procitis.
Hepatic Disorders	Autoimmune hepatitis, autoimmune cholangitis, primary sclerosis cholangitis, primary biliary cirrhosis
Renal Disorders	Autoimmune glomerulonephritis (including IgA nephropathy, glomerulonephritis rapidly progressive, membranous glomerulonephritis, membranoproliferative glomerulonephritis, and mesangioproliferative glomerulonephritis)
Cardiac Disorders	Autoimmune myocarditis/cardiomyopathy

Skin Disorders	Alopecia areata, psoriasis, vitiligo, Raynaud's phenomenon, erythema no do sun, autoimmune bulbous skin disease (including pemphigus, pemphigoid and dermatitis herpetiformis), cutaneous lupus erythematosus, morphed, lichen plants, Stevens-Johnson syndrome, Sweet's syndrome
Hematologic Disorders	Autoimmune hemolytic anemia, autoimmune thrombocytopenia, antiphospholipid syndrome, thrombocytopenia
Metabolic Disorders	Autoimmune thyroiditis, Grave's or Basedow's disease, new onset Hashimoto thyroiditis, diabetes mellitus type 1, Addison's disease
Other Disorders	Goodpasture syndrome, idiopathic pulmonary fibrosis, pernicious anemia, sarcoidosis.

Abbreviations: ANCA: anti-neutrophil cytoplasmic antibody, IgA: immunoglobulin A.

9.5.6.6 Assessment of Severity

All AEs, except AEs in e-diary card, will be assessed according to the US NCI Common Terminology Criteria for Adverse Events (CTCAE) 5.0 (published on November 27, 2017) associated with the AE term. The following standard with 5 grades is to be used to measure the severity of adverse events in this study.

Table 9-7 Intensity scales of AE

Grades of AE	Description
Grade 1	Mild; asymptomatic or mild symptoms; clinical or diagnostic observations only; intervention not indicated.
Grade 2	Moderate; minimal, local or noninvasive intervention indicated; limiting age-appropriate instrumental ADL*
Grade 3	Severe or medically significant but not immediately life-threatening; hospitalization or prolongation of hospitalization indicated; disabling, limiting self care ADL**
Grade 4	Life-threatening consequences; urgent intervention indicated.
Grade 5	Death related to AE.

* Instrumental activities of daily living (ADL) refer to preparing meals, shopping for groceries or clothes, using the telephone, managing money, etc.

** Self-care ADL refer to bathing, dressing and undressing, feeding self, using the toilet, taking medication, and not bedridden.

9.5.6.7 The Relationship to Study Vaccine

The investigator will make an assessment of the relationship between investigational vaccine and the occurrence of each AE/SAE, except solicited reactions to vaccination. The reasonable possibility will be determined based on the investigator's clinical judgment. The causality should be considered as one of the categories described below.

Table 9-8 The relationship between AE and study vaccine

Causality term	Assessment criteria
Certain	• Event or laboratory test abnormality, with plausible time relationship to drug intake<br>• Cannot be explained by disease or other drugs<br>• Response to withdrawal plausible (pharmacologically, pathologically)<br>• Event definitive pharmacologically or phenomenologically (i.e. an objective and specific medical disorder or a recognized pharmacological phenomenon)<br>• Rechallenge satisfactory, if necessary
Probable / Likely	• Event or laboratory test abnormality, with reasonable time relationship to drug intake<br>• Unlikely to be attributed to disease or other drugs<br>• Response to withdrawal clinically reasonable<br>• Rechallenge not required
Possible	• Event or laboratory test abnormality, with reasonable time relationship to drug intake<br>• Could also be explained by disease or other drugs<br>• Information on drug withdrawal may be lacking or unclear
Unlikely	• Event or laboratory test abnormality, with a time to drug intake that makes a relationship improbable (but not impossible)<br>• Disease or other drugs provide plausible explanations
Unrelated	• Occurred before dosing<br>• Due wholly to factors other than study treatment

Each event should be followed until resolution or the event is considered stable. Both regular return and telephone contact will be acceptable.

9.5.6.8 Adverse Events Reporting

Documentation and Reporting of Adverse Events

All investigators should follow up subjects with AEs until the event is resolved or until, in the opinion of the investigator, the event is stabilized or determined to be chronic. Details of AE resolution must be documented in the CRF.

All AEs should be reported and documented in accordance with the procedures outlined in this section. All AEs occurring during the study must be documented on the relevant CRF pages.

Reporting of Serious Adverse Events

Any SAE must be reported by the investigator if it occurs during the clinical study, whether or not the SAE is considered to be related to the investigational product. An SAE report consists of the SAE form, the AE form, and the concomitant medication form. A copy of these forms must be emailed **within 24 hours** to Contract Research Organization (CRO), StatPlus Inc., and StatPlus will inform United Biomedical, Inc., Asia on the same day.

The investigator should not wait to receive additional information to document fully the event before notification of a SAE, though additional information may be requested. Where applicable, information from relevant laboratory results, hospital case records, and autopsy reports should be obtained.

Instances of death, congenital abnormality, or an event that is of such clinical concern as to influence the overall assessment of safety, if brought to the attention of the investigator at any time after cessation of study agent administration and linked by the investigator to this study, should be reported to the study monitor.

The sponsor and/or the appointed representative(s) will promptly notify all relevant investigators and the regulatory authorities of findings that could adversely affect the safety of subjects, impact on the conduct of the study, or alter the IEC/ IRB approval/favorable opinion of the study. In addition, the sponsor and/or the appointed representative(s), will expedite the reporting to all concerned investigators, to the IEC(s)/IRB(s), where required, and to the regulatory authorities of all adverse reactions that are both serious and unexpected.

Details of the procedures to be followed if a pregnancy occurs are also provided in Section 7.4.8.4.

Documentation and Reporting of SUSARs

All suspected unexpected serious adverse reactions (SUSARs) will be the subject of expedited reporting. The sponsor and/or the appointed representative(s) shall ensure that all relevant information about a SUSAR that is fatal or life-threatening is reported to the relevant competent authorities and IEC/IRB within 7 days after knowledge by the sponsor of such a case and that relevant follow-up information is communicated within an additional 8 days. All other SUSARs will be reported to the relevant competent authorities and IEC/IRB within 15 days after knowledge by the sponsor of such a case. All investigators should follow up SUSARs until the event is resolved or until, in the opinion of the investigator, the event is stabilized or determined to be chronic. Poststudy SUSARs that occur after the subject has completed the clinical study must be reported by the investigator to the sponsor.

Documentation and Reporting of SARS-CoV-2 infection

All subjects will be surveilled for potential COVID-19 illness from Visit 1 onwards. Potential COVID-19 illnesses and their sequelae that are consistent with the clinical endpoint definition should not be recorded as AEs or SAEs, even though the event may meet the definition of an SAE. These data will be captured as efficacy assessment data only on the relevant pages of the CRF, as these are expected endpoints.

Potential COVID-19 illness events and their sequelae will be reviewed by IDMC. Any SAE that is determined by the IDMC not to meet endpoint criteria is reported back to the SAE reporting process. The investigator’s SAE awareness date is the date on which events is confirmed not to meet endpoint criteria.

9.5.7 Immunogenicity Assessments

9.5.7.1 Surveillance and Laboratory Diagnosis of SARS-CoV-2 Infection during Clinical Trial

During the observation period of the clinical trial, the participants with fever, cough and other respiratory symptoms should immediately go to the designated hospital. The doctor or investigator may collect the nasopharyngeal/throat swabs and to perform CT and other imaging examinations to analyse whether it is caused by SARS-CoV-2 infection. In the event of SARS-CoV-2's infection during the clinical trial, it is necessary to conduct a case investigation, and the critically ill or dead cases need to continue to conduct a special investigation, mainly to analyze whether there is an ADE or VAERD phenomenon. Subjects encountered the medical condition listed in Table 9-5 should also check HLA and SARS-CoV-2 infection,

In addition to SARS-CoV-2 nucleic acid detection, multiple pathogens will be detected for differential diagnosis of swabs. The detail of detectable pathogens of test kit will be listed below:

- ✓ Adenovirus
- ✓ Coronavirus 229E
- ✓ Coronavirus HKU1
- ✓ Coronavirus NL63
- ✓ Coronavirus OC43
- ✓ Human Metapneumovirus
- ✓ Human Rhinovirus/Enterovirus
- ✓ Influenza virus A
- ✓ Influenza virus B
- ✓ Parainfluenza virus 1
- ✓ Parainfluenza virus 2
- ✓ Parainfluenza virus 3
- ✓ Parainfluenza virus 4

- ✓ Respiratory syncytial virus
- ✓ Bordetella parapertussis
- ✓ Bordetella pertussis
- ✓ Chlamydia pneumoniae
- ✓ Mycoplasma pneumoniae

The potential SARS-CoV-2 infection will be reviewed by IDMC.

9.5.7.2 Detection of ELISA Antibodies against S1-RBD of SARS-CoV-2

Anti-S1-RBD antibody titers will be measured by ELISA kit. A dilution microplate is used to prepare the serum dilutions per SOP for test serum samples. Anti-S1-RBD antibody level is expressed as linear titers of an end point dilution for a test sample. SoftMax Titer Calculation Program (Molecular Devices Co.) is used to calculate the titers. For seroconversion detected by UBI SARS-CoV2 S1-RBD ELISA, it is defined as a 4-fold increase in antibody titer from baseline.

9.5.7.3 Detection of Antibody Titers Which Inhibit S1-RBD:ACE2 binding

Antibody titers for ability to inhibit S1-RBD:ACE2 binding will be measured by ELISA kit. A dilution microplate is used to prepare the serum dilutions per SOP. Antibody levels which inhibit S1-RBD:ACE2 binding are expressed in μg/mL as titers for a test sample. SoftMax Titer Calculation Program (Molecular Devices Co.) is used to calculate the titers. Specimens that do not react in the test are considered with < 1.6 μg/mL titer in this SARS-CoV-2 qNeu Ab ELISA.

9.5.7.4 Neutralizing Antibody Titers against SARS-CoV-2

Neutralizing antibody titers will be measured by CPE-based live virus neutralization assay using Vero-E6 cells infected with SARS-CoV-2. The study will be conducted in the BSL-3 lab at Academia Sinica, Taipei. The levels of SARS-CoV-2 virus specific neutralization titers are measured based on the principle of VNT_{50} titer (≥ 50% reduction of virus-induced cytopathic effects). Virus neutralization titer of a serum specimen is defined as the reciprocal of the highest serum dilution at which 50% reduction in cytopathic effects are observed and results are calculated by the method of Reed and Muench. For seroconversion detected by live virus neutralization test, it is defined as a 4-fold increase in antibody titer from baseline.

9.5.7.5 Detection of antibody against SARS-CoV-2 antigens

Specific IgG antibodies against SARS-CoV-2 antigens derived from S2, N, and M proteins in the serum of subjects will be tested by UBI SARS-CoV-2 ELISA (antigen combinations) and Confirmatory SARS-CoV-2 ELISA (individual antigens). These ELISA tests can distinguish immune responses in infected versus vaccinated individuals.

9.5.7.6 Detection of T Cell Response

Human peripheral blood mononuclear cells will be used for evaluating vaccine-induced T cell responses. Antigen-specific interferon-gamma (IFN-γ) and IL-4 production will be measured by

ELISpot. Intracellular cytokine staining and flow cytometry will be used to evaluate $CD4^+$ and $CD8^+$ T cell responses. This assessment will be optional.

9.5.8 Communication and Use of Technology

This study may employ various methods, such as e-dairy, telephone, or other communication pathway, to contact with participants for record and maintain the study information. The communication items include but not limited to:

- Record 7-day solicited symptoms and 14-day skin allergic reaction after vaccination in reactogenicity e-diary
- Record unsolicited AEs or/and symptoms of COVID-19 on Day 8, Day 15, Day 22, Day 36, Day 43, Day 64, Day 71, Day 78, Day 85**, the day on 7 days after Visit 6 (only for subjects receiving 3rd dose),** Day 253, and Day 309 using telephone communication
- Record regular surveillance for COVID-19 illness in e-dairy. A prompt on their smartphone device to regularly surveillance for signs and symptoms of COVID-19 every week after Day 57. If any potential symptoms for COVID-19 illness is reported, a telephone call will be made between site staffs and subjects.
- Unscheduled visit set-up or visit reminders

9.5.9 Appropriateness of Measurements

The immunogenicity and safety assessments planned for this study are generally recognized as reliable, accurate, and relevant to the diagnostic modality and underlying disease/condition.

9.6 Independent Data Monitoring Committee (IDMC)

The Independent Data Monitoring Committee (IDMC) will act in an expert, independent advisory capacity to monitor participant safety and evaluate the efficacy of UB-612 vaccine against COVD-19 in this study. The IDMC will consist of at least two physicians and one statistician. An IDMC meeting will be held at:

(1) 1st time: when all adult immunogenicity data (350 evaluable young adults and 154 evaluable elderly subjects) for Day 57 are available
(2) 2nd time: when at least half of core group subjects will be completed Day 85 safety follow up. Adolescence could be enrolled after 2nd IDMC meeting.
(3) 3rd time: when all immunogenicity data for lot-to lot consistency of Day 57 are available.
(4) 4th time: when 350 evaluable adolescents will be completed Day 57 safety follow up.

The meeting schedule might be adjusted based on the progress of recruitment. The IDMC meetings may also be held at any time point if any situations after vaccination meet the study stopping criteria or when the sponsor considers it necessary. Unscheduled IDMC meetings may also be held if any potential COVID-19 illness events or obvious situations for the deterioration the subject safety or study conduct occur.

Responsibilities of the IDMC include the following:

- ✓ Protect the safety and confidentiality of the study participants;

- ✓ Evaluate the quality and validity of the data generated in this study;
- ✓ Consider factors external to the study when relevant information becomes available, such as scientific or therapeutic developments that may have an impact on the safety of the participants or the ethics of the trial;
- ✓ Make recommendations to the Sponsor and the Principal Investigator (PI) concerning continuation, termination or other modifications of the study based on factors including overwhelming effectiveness, futility or safety issues;
- ✓ Make recommendations and assist in the resolution of problems reported by the PI;
- ✓ Assist sponsor by commenting on any problems with study conduct and/or data collection;
- ✓ Maintain confidentiality regarding the study outside the IDMC (including, but not limited to the investigators, IRBs, regulatory agencies, or sponsor) except as authorized by the IDMC.

Detailed description of the IDMC responsibilities, data reviewed, meeting process, considerations and policies will be described in a separate IDMC charter.

9.6.1 Overall Study Stopping Rules

The following stopping rules are in place for all subjects, based on review of AE data and e-diary reactogenicity data.

1. An SAE that is assessed by the investigator as possibly related, or for which there is no alternative, plausible, attributable cause.
2. A Grade 4 local reaction, systemic adverse event or fever that is assessed as possibly related by the investigator, or for which there is no alternative, plausible, attributable cause.
3. If any 3 subjects vaccinated with UB-612 (at any dose level) report the same or similar* severe (Grade 3) AE (including laboratory abnormalities), assessed as possibly related by the investigator, or for which there is no alternative, plausible, attributable cause.

   * defined as same preferred term of current MedDRA
4. If a stopping rule is met, further dosing will be paused and the IDMC will determine whether dosing should resume.
5. If two of the same or similar *SAEs or Grade 4 local or systemic reactions occur, dosing will be suspended and both the IDMC and regulatory authorities will be consulted before resumption of dosing.

   * defined as same preferred term of current MedDRA

In the event that a stopping rule is met, the following actions will be taken:

- The stopping rule will PAUSE randomization and administration of study vaccine at all dose levels and all treatment groups.
- The IDMC will review the relevant safety data.

- For all subjects already vaccinated, all other routine study conduct activities, including ongoing data entry, reporting of AEs, electronic diary completion, blood sample collection, and subject follow-up, will continue during the pause.
- Data from placebo recipients will not contribute to the stopping rules.
- Electronic diary data confirmed by the investigator as having been entered in error will not contribute toward a stopping rule.

Should the study be terminated prematurely, the sponsor will provide written notification to all investigators and regulatory authorities specifying the reason(s) for early termination. The investigator must inform the institutional review board (IRB)/independent ethics committees (IEC) promptly and provide the reason(s) for the termination. Previously dosed subjects will be assessed through all planned study safety visits.

10 STATISTICAL METHODS

The statistical planning and analysis of the trial will be performed by the designated contract research organization.

10.1 Statistical and Analytical Plans

A statistical analysis plan will be prepared and finalized prior to database lock of the study. The statistical analysis plan will include full details of all planned statistical analyses.

10.2 Sample Size Determination

Consider a 10% drop-out rate, around 3850 adult subjects in core group will be recruited and randomized to UB-612 vaccine 100 µg or placebo with 6:1 allocation rate and around 385 adolescents will be recruited for supplementary group and randomized to UB-612 vaccine 100 µg or placebo with same allocation ratio.

10.2.1 Sample Size for Safety Evaluation

For safety outcomes, the following table shows the probability of observing at least 1 SAE for a given true event rate of a particular SAE. For example, if the true SAE rate is 0.01%, with 2400 adult subjects, 600 elderly subjects, and 300 adolescent subjects received select vaccine dose, there are 21.3%, 5.8%, and 3.0% probabilities of observing at least 1 SAE. Overall, probability of observing at least 1 SAE is 25.9% with true SAE rate of 0.01% for all 3000 subjects received study vaccine in safety group.

True SAE Rate	N=300	N=600	N=2400	N=3000
0.001%	0.003	0.006	0.024	0.030
0.010%	0.030	0.058	0.213	0.259
0.100%	0.259	0.451	0.909	0.950

10.2.2 Sample Size for Lot-to-Lot Consistency

The sample size is driven by the objective to demonstrate the consistency of GMT ratio (GMTR) with the 3 consecutive manufacturing lots of the UB-612 vaccine. The clinical lot-to-lot consistency will be tested for the three pair-wise comparisons by computing the two-sided 95% CI on the GMTR. If all confidence intervals are within the pre-defined clinical limits of [0.5, 2.0], one can conclude that the lots are consistent.

Assume the expected GMTR of 1 and a value of 0.6 for the standard deviation (SD) of the decimal logarithmic transformation (log base 10) of antibody titers (log10(GSD) is about 0.2 to 0.6; refer to following table of the V-122 interim result). In order to have at least 90% power to achieve the lot-to-lot consistency, the estimated sample size should ensure beta less than 3.3% (Bonferroni adjustment of beta for 3 comparisons between 3 lots). With the parameters above it estimates a minimum evaluable sample size per lot of 115 subjects. Consider a 10% drop-out rate, a total of 396 subjects (132 subjects per lot) will be needed for ensuring overall lot-to-lot consistency with at least 90% overall power.

Table GSD and Logarithmic Transformation of Neutralizing Antibody Titers and Anti-S1-RBD Antibody Titers at Day 56

Antibody Titers	**UB-612 10 µg**		**UB-612 30 µg**		**UB-612 100 µg**	
	GSD	log10(GSD)	GSD	log10(GSD)	GSD	log10(GSD)
Neutralizing	3.286	0.52	2.438	0.39	1.670	0.223
Anti-S1-RBD	4.185	0.62	3.112	0.49	1.436	0.16

Note: Only first six subjects in **100 µg** group had neutralizing antibody titer results at Day 56 until 2021-01-12

10.3 Analysis Populations

Enrolled Population

Enrolled Population includes subjects who have a signed ICF.

Randomized Population

Subjects who are assigned a random number be regarded as Randomized Population.

Evaluable Immunogenicity Population

Evaluable Immunogenicity Population will consist of all eligible randomized subjects who are assigned to **lot-to-lot and** immunogenicity group, receive two vaccinations within the predefined window, have a valid immunogenicity result at visit 4 (Day 57), have no major protocol deviations or protocol deviations having impact on immunogenicity data. Evaluable Immunogenicity Population will be regarded as primary population for immunogenicity evaluation on primary and secondary immunogenicity endpoints except lot-to-lot consistency.

Evaluable Lot-to-Lot Population

Evaluable Lot-to-Lot Population is a subset of Evaluable Immunogenicity but only includes subjects who are assigned to lot-to-lot consistency group and have immunogenicity determination at Day 57. Evaluable Lot-to-Lot Population will be used for evaluating lot-to-lot consistency only.

Evaluable Efficacy Population

All eligible randomized subjects who receive two vaccinations within the predefined window and have no major protocol deviations or protocol deviations **having impact on immunogenicity data** will be the Evaluable Efficacy Population. Evaluable Efficacy Population will be used to evaluate vaccine efficacy in exploratory analysis.

Available Immunogenicity Population

Available Immunogenicity Population will consist of all eligible randomized subjects who receive at least one vaccination, and have at least one post immunogenicity data determination. Available Immunogenicity Population will also be used to the immunogenicity evaluation except lot-to-lot consistency.

Evaluable Booster Population

All eligible randomized subjects who receive third dose of UB-612 within the predefined window and have no major protocol deviations or protocol deviations having impact on immunogenicity data will be the Evaluable Booster Population. Evaluable Booster

Population will be used to explore the immunogenicity evaluation after the booster vaccination.

Safety Population

Safety Population (SAF) will consist of all subjects who received at least one vaccination. The Safety Population is for safety evaluation in analysis.

10.4 Demographic and Other Baseline Characteristics

Demographic and baseline characteristic data will be summarized for each study group and overall. Descriptive statistics (N, mean, standard deviation, median, minimum, and maximum) will be presented for continuous variables. The number and percentage of subjects in each category will be presented for categorical variables. No formal testing of demographic or baseline characteristics will be performed.

10.5 Safety Evaluation

All safety assessments, including AEs, PEs, VS and clinical laboratory evaluations, where indicated, will be presented using descriptive statistics for each study group of UB-612. Data will be summarized for each study group.

Local Reactions and Systemic Events

Local reactions and systemic events recorded on e-diary will be summarized by the severity grading scales by study groups. Numbers and percentages of subjects experiencing each local reaction/systemic event will be presented for each symptom severity by study groups. Summary tables showing the occurrence of any local reactions and occurrence of any systemic event will also be presented.

Unsolicited Adverse Event

This analysis applies to all unsolicited adverse events occurring during the study, recorded in AE eCRF, with a start date on or after the date of vaccination. Unsolicited AEs occurring during the study will be coded using the most recent version of Medical Dictionary for Regulatory Activities (MedDRA). The adverse events will then be grouped by MedDRA preferred terms into frequency tables according to system organ class.

All reported unsolicited adverse events, as well as adverse events judged by the investigator as at least possibly related to study vaccine, will be summarized according to system organ class and preferred term within system organ class. When an unsolicited adverse event occurs more than once for a subject, the maximal severity and strongest relationship to the study group will be counted.

Separate summaries will be produced for the following categories, such as serious adverse events and unsolicited AEs related to vaccinations will be presented. Data listings of all unsolicited adverse events will be provided by subject.

Physical Examination

All physical examination findings will be listed and summarized by time point and study group. Shift table will be also presented, if appropriate.

Vital Signs

All vital sign findings will be listed and summarized by time point and study group.

Laboratory Evaluations

Laboratory safety data will be analysed descriptively by time point and study group. Shift table for laboratory data will be shown by visit using categorization of laboratory according to local laboratory's normal reference range.

10.5.1 Analysis of Primary Safety Endpoint(s)

- Local reactions for up to 7 days following each dose

  Local reactions for up to 7 days following each dose will be summarized with counts and percentages of participants with the indicated endpoint and the associated Clopper-Pearson 95% CIs by study group and each vaccination. The group comparison will be assessed by Fisher's exact test. Summary of descriptive statistics by age group will be also provided.

- Systemic events for up to 7 days following each dose

  Systemic events for up to 7 days following each dose will be summarized with counts and percentages of participants with the indicated endpoint and the associated Clopper-Pearson 95% CIs by study group and each vaccination. The group comparison will be assessed by Fisher's exact test. Summary of descriptive statistics by age group will be also provided.

- Unsolicited AEs from Day 1 to Day 57

  Unsolicited AEs will be presented by descriptive summary statistics (counts, percentages, and associated Clopper-Pearson 95% CIs) with system organ class and preferred term for each study group. The group comparison will be assessed by Fisher's exact test. Summary of descriptive statistics by age group will be also provided.

- MAAEs and SAEs from Day 1 to Day **365**

  MAAEs and SAEs will be summarized by counts, percentages, and associated Clopper-Pearson 95% CIs for each study group. The group comparison will be assessed by Fisher's exact test. Summary of descriptive statistics by age group will be also provided. **Additionally, the AESIs and ADEs occurred after third vaccination for subjects received three vaccinations will be also summarized separately.**

- AESIs and ADEs from Day 1 to Day 365

  AESIs and ADEs will be summarized by counts, percentages, and associated Clopper-Pearson 95% CIs for each study group. The group comparison will be assessed by Fisher's exact test. Summary of descriptive statistics by age group will be also provided. **Additionally, the AESIs and ADEs occurred after third vaccination for subjects received three vaccinations will be also summarized separately.**

10.5.2 Analysis of Secondary Safety Endpoint

- Changes of safety laboratory measures

Changes of safety laboratory measures will be summarized with descriptive statistics by study group and time point. ANCOVA model with laboratory baseline values as covariate will analyse changes of safety laboratory measures for testing the difference between study groups. Intra-group difference in safety laboratory measures will also be analysed by paired t test. Summary of descriptive statistics by age group will be also performed.

10.6 Immunogenicity Evaluation

10.6.1 Analysis of Primary Immunogenicity Endpoint(s)

- Geometric mean titer (GMT) of SARS-CoV-2 neutralizing antibody on Day 57

  For SARS-CoV-2 neutralizing antibody, GMT and 2-sided 95% CIs will be provided for each study group. Additionally, the difference between UB-612 vaccine and placebo groups will be analysed by ANCOVA model under log-transform data with baseline level as covariate and effect of study treatment, if appropriate. Summary of descriptive statistics by age group will be also performed.

- Seroconversion rate (SCR) of SARS-CoV-2 neutralizing antibody on Day 57

  The SCR will be presented as count and percentage in frequency table, and the 95% exact (Clopper-Pearson) confidence interval will be provided as well by study group. Fisher's exact test will be used for the comparison of UB-612 vaccine group and placebo group. Summary of descriptive statistics by age group will be also performed.

10.6.2 Analysis of Secondary Immunogenicity Endpoint(s)

- Seroconversion rate (SCR) of antigen-specific antibody (Anti-S1-RBD) on Day 57

  The SCR will be presented as count and percentage in frequency table, and the 95% exact (Clopper-Pearson) confidence interval will be provided as well by study group. Fisher's exact test will be used for the comparison of UB-612 vaccine group and placebo group. Summary of descriptive statistics by age group will be also performed.

- Geometric mean titer (GMT) of SARS-CoV-2 neutralizing antibody on Day 197 and 365

  GMT and 2-sided 95% CIs will be provided for each study group. Additionally, the difference between UB-612 vaccine and placebo groups will be analysed by ANCOVA model under log-transform data with baseline level as covariate and effect of study treatment, if appropriate. Summary of descriptive statistics by age group will be also performed.

  In addition, the GMT of SARS-CoV-2 neutralizing antibody on Day 365 for subjects received two vaccinations and three vaccinations should be analysed separately. Analysis of GMT of SARS-CoV-2 neutralizing antibody on Day 365 for subjects received third dose of UB-612 will be performed with the Evaluable Booster Population only.

- Geometric mean titer (GMT) of antigen-specific antibody (Anti-S1-RBD) on Day 57, 197, and 365

GMT and 2-sided 95% CIs will be provided for each study group. Additionally, the difference between UB-612 vaccine and placebo groups will be analysed by ANCOVA model under log-transform data with baseline level as covariate and effect of study treatment, if appropriate. Summary of descriptive statistics by age group will be also performed.

In addition, the GMT of Anti-S1-RBD on Day 365 for subjects received two vaccinations and three vaccinations should be analysed separately. Analysis of GMT of Anti-S1-RBD on Day 365 for subjects received three vaccinations will be performed with the Evaluable Booster Population only.

- Geometric mean fold increase in antigen-specific antibody (Anti-S1-RBD) and SARS-CoV-2 neutralizing antibody on Day 57, 197 and 365

  GMFI and 2-sided 95% CIs will be provided for each study group. Additionally, the difference between UB-612 vaccine and placebo groups will be analysed by Student's t test under log-transform data. Summary of descriptive statistics by age group will be also performed.

  In addition, the GMFI of Anti-S1-RBD on Day 365 for subjects received two vaccinations and three vaccinations should be analysed separately. Analysis of GMFI of Anti-S1-RBD on Day 365 for subjects received third dose of UB-612 will be performed with the Evaluable Booster Population only.

- Lot consistency as assessed by the comparisons of the GMT of SARS-CoV-2 neutralizing antibody on Day 57 induced by 3 independent UB-612 vaccine clinical materials. The 95% confidence intervals between groups will be within the margin of 0.5 to 2.

  For lot consistency, the 1 month after 2nd vaccination, for all pairs of lots, the two sided 95% CIs for the GMT ratios (GMTR) of neutralizing antibody will be calculated. For all pairs of lots, if the two-sided 95% CIs for the GMTR are within the [0.5,2.0] clinical limit interval, lot consistency will be concluded. The GMTR will be calculated as the mean of the difference of decimal logarithmically transformed antibody titers and exponentiating the mean with base 10. The associated 2-sided CIs will be obtained by calculating CIs using Student's t-distribution for the mean difference of the logarithmically transformed antibody titers and exponentiating the confidence limits with base 10.

10.7 Analysis of Exploratory Endpoint(s)

- T cell responses to UB-612 measured by ELISpot and flow cytometric assays on Day 57

  T cell responses to UB-612 measured by ELISpot and flow cytometric assays on Day 57 will be summarized by descriptive statistics.

- **T cell responses to UB-612 measured by ELISpot and flow cytometric assays on 14 days post 3rd dose of UB-612**

  T cell responses to UB-612 measured by ELISpot and flow cytometric assays on 14 days post 3rd dose of UB-612 will be summarized by descriptive statistics.

- **Geometric mean titer (GMT) of SARS-CoV-2 neutralizing antibody on 14 days post 3rd dose of UB-612**

  GMT of SARS-CoV-2 neutralizing antibody on 14 days post 3rd dose of UB-612 and 2-sided 95% CIs will be provided for subjects who received third dose of UB-612. Summary of descriptive statistics by age group will be also performed. Analysis of GMT of SARS-CoV-2 neutralizing antibody on 14 days post 3rd dose of UB-612 for subjects received third dose of UB-612 will be performed with the Evaluable Booster Population only. For subjects received third dose of UB-612, GMT of SARS-CoV-2 neutralizing antibody on 14 days post 3rd dose of UB-612 will compare with GMT of SARS-CoV-2 neutralizing antibody on Visit 6 (pre-3rd dose baseline) by paired t test. This analysis will be performed with the Evaluable Booster Population only.

- **Geometric mean titer (GMT) of antigen-specific antibody (Anti-S1-RBD) on 14 days post 3rd dose of UB-612**

  GMT of Anti-S1-RBD on 14 days post 3rd dose of UB-612 and 2-sided 95% CIs will be provided for subjects who received third dose of UB-612. Summary of descriptive statistics by age group will be also performed. Analysis of GMT of Anti-S1-RBD on 14 days post 3rd dose of UB-612 for subjects received third dose of UB-612 will be performed with the Evaluable Booster Population only. For subjects received third dose of UB-612, GMT of Anti-S1-RBD on 14 days post 3rd dose of UB-612 will compare with GMT of Anti-S1-RBD on Visit 6 (pre-3rd dose base line) by paired t test. This analysis will be performed with the Evaluable Booster Population only.

- **Geometric mean fold increase in SARS-CoV-2 neutralizing antibody and antigen-specific antibody (Anti-S1-RBD) on14 days post 3rd dose against pre-3rd dose baseline**

  GMFI and 2-sided 95% CIs will be provided. Summary of descriptive statistics by age group will be also performed. This analysis will be performed with the Evaluable Booster Population only.

- **Geometric mean titer (GMT) of SARS-CoV-2 neutralizing antibody and antigen-specific antibody (Anti-S1-RBD) on Day 57, Day 197 and Day 365 in adolescents**

  The analysis of GMT of SARS-CoV-2 neutralizing antibody on Day 57, Day 197, 14 days post 3rd dose and Day 365 in adolescents will follow the analysis described above in GMT.

- **Seroconversion rate (SCR) of SARS-CoV-2 neutralizing antibody on Day 57 and antigen-specific antibody (Anti-S1-RBD) in adolescents**

  The analysis of SCR of SARS-CoV-2 neutralizing antibody on Day 57 in adolescents will follow the analysis described above in SCR.

- **Geometric mean fold increase in SARS-CoV-2 neutralizing antibody and antigen-specific antibody (Anti-S1-RBD) on Day 57, Day 197, and Day 365 in adolescents**

The analysis of GMFI in SARS-CoV-2 neutralizing antibody and antigen-specific antibody (Anti-S1-RBD) on Day 57, Day 197, and Day 365 in adolescents will follow the analysis of secondary immunology endpoint in GMFI.

- Local reactions for up to 7 days following each dose in adolescents

  The analysis of local reactions for up to 7 days following each dose in adolescents will follow the analysis of local reactions in primary safety endpoints.

- Systemic events for up to 7 days following each dose in adolescents

  The analysis of systemic events for up to 7 days following each dose in adolescents will follow the analysis of systemic events in primary safety endpoints.

- Unsolicited AEs from Day 1 to Day 57 in adolescents

  The analysis of unsolicited AEs from Day 1 to Day 57 in adolescents will follow the analysis of unsolicited AEs in primary safety endpoints.

- **MAAEs and SAEs from Day 1 to Day 365 in adolescents**

  The analysis of MAAEs and SAEs from Day 1 to Day 365 in adolescents will follow the analysis of MAAEs and SAEs in primary safety endpoints.

- **AESIs and ADEs from Day 1 to Day 365 in adolescents**

  The analysis of AESIs and ADEs from Day 1 to Day 365 in adolescents will follow the analysis of AESIs and ADEs in primary safety endpoints.

- Changes of safety laboratory measures in adolescents

  The analysis of changes of safety laboratory measures in adolescents will follow the analysis of changes of safety laboratory measures in secondary safety endpoint.

- COVID-19 incidence per 1000 person-years of follow-up based on PCR test

  COVID-19 incidence per 1000 person-years of follow-up based on PCR test will be descriptively summarized by counts, percentages, and associated Clopper-Pearson 95% CIs for each study group.

- To describe the anti-S1-RBD IgG levels and SARS-CoV-2 neutralizing titers to UB-612 in confirmed and/or severe COVID-19 cases

  The anti-S1-RBD IgG levels and SARS-CoV-2 neutralizing titers to UB-612 in confirmed COVID-19 cases will be presented by descriptive summary statistics and associated 95% CIs for each study group.

- **To detect antibody against SARS-CoV-2 antigens derived from S2, N, and M protein**

The results of UBI SARS-CoV-2 ELISA will be presented by descriptive summary statistics

10.8 Subgroup Analysis

Additional study populations and subgroups will be assessed, with further information available in the SAP.

10.9 Interim Analysis

Six interim analyses and reports will be performed when (1) adult immunogenicity data (350 evaluable young adults and 154 evaluable elderly subjects) for Day 57 are available; (2) at least half of core group subjects (at least 3500 evaluable subjects) will be completed Day 85 safety follow up, as EUA application dossier; (3) all immunogenicity data for lot-to lot consistency of Day 57 are available, EUA application dossier; (4) 350 evaluable adolescents completed Day 57 safety follow up, as supplementary dossier for EUA**; (5) immunogenicity and safety data for young and elderly adult subjects receiving 3rd vaccination completed Visit 7, which is 14 days after 3rd vaccination; (6) immunogenicity and safety data for adolescent subjects receiving 3rd vaccination completed Visit 7, which is 14 days after 3rd vaccination.**

The first four analyses and reports will be analysed and drafted by the unblinded statistician, reviewed by the IDMC, and submitted to appropriate regulatory authorities for review if as EUA application dossier or supplementary dossier for EUA.

For the evaluation of safety, immunogenicity, and lot-to-lot consistency, the following endpoints will be included in the first four interim analyses.

- Local reactions, systemic events, unsolicited AEs, and SAEs
- Changes of safety laboratory measures
- Changes of vital signs and physical examinations
- Seroconversion rate of SARS-CoV-2 neutralizing antibody and antigen-specific antibody (Anti-S1-RBD) on Day 57
- Geometric mean titer of SARS-CoV-2 neutralizing antibody and antigen-specific antibody (Anti-S1-RBD) on Day 57
- Geometric mean fold increase in SARS-CoV-2 neutralizing antibody and antigen-specific antibody (Anti-S1-RBD) on Day 57
- Lot consistency by the comparisons of the GMT of SARS-CoV-2 neutralizing titers on Day 57 induced by 3 independent UB-612 vaccine clinical materials. The 95% confidence intervals between groups will be within the margin of 0.5 to 2.

For last two interim analyses (i.e., interim analysis (5) and (6)), the analyses demonstrate the immunogenicity and safety data on the adult and adolescent subjects received 3rd dose of UB-612 including (but not limited to) following endpoints.

- **Local reactions, systemic events, unsolicited AEs, and SAEs after 3rd dose of UB-612**
- **Changes of safety laboratory measures**
- **Changes of vital signs and physical examinations pre/post 3rd dose of UB-612**

- **GMT of SARS-CoV-2 neutralizing antibody on 14 days post 3rd dose of UB-612**
- **GMT of antigen-specific antibody (Anti-S1-RBD) on 14 days post 3rd dose of UB-612**
- **GMFIs in SARS-CoV-2 neutralizing antibody and antigen-specific antibody (Anti-S1-RBD) on14 days post 3rd dose against pre-3rd dose baseline**

10.10 Handling of Missing Data

All available data will be displayed and utilized in data analysis. No imputation will be considered for the missing observations.

10.11 Protocol Deviations

Protocol deviations will be categorized into important and non-important items, and definitions will be illustrated in the protocol deviation handling plan (PDHD). Events that beyond the PDHD will discuss with sponsor to determine the categorization.

11 QUALITY ASSURANCE AND QUALITY CONTROL

11.1 Audit and Inspection

Study centers and study documentation may be subject to Quality Assurance audit during the course of the study by the sponsor or its nominated representative. In addition, inspections may be conducted by regulatory authorities at their discretion.

11.2 Monitoring

Data for each subject will be recorded on an eCRF. Data collection must be completed for each subject who signs an ICF and is administered study agent.

Due to study design, monitoring activities are divided into blinded and unblinded team. Blinded team will follow the Blinded Monitoring Plan, while unblinded team should follow the Unblinded Monitoring Plan. Unblinded team will report to unblinded UBIA staff. **Members in blinded team should be blinded to immunogenicity data.**

In accordance with current good clinical practice (cGCP) and International Council for Harmonisation (ICH) guidelines, the study monitor will carry out source document verification at regular intervals to ensure that the data collected in the eCRF are accurate and reliable.

The investigator must permit the monitor, the IEC/IRB, the sponsor's internal auditors, and representatives from regulatory authorities direct access to all study-related documents and pertinent hospital or medical records for confirmation of data contained within the eCRFs.

11.3 Data Management and Coding

The sponsor and/or the appointed representative(s) will be responsible for activities associated with the data management of this study. This will include setting up a relevant database and data transfer mechanisms, along with appropriate validation of data and resolution of queries. Data generated within this clinical study will be handled according to the relevant standard operating procedures of the data management and biostatistics departments of the sponsor and/or the appointed representative(s).

Study centers will complete the eCRF. Data entered into the eCRF must be verifiable against source documents at the study center. Data to be recorded directly on the eCRF will be identified and the eCRF will be considered the source document. Any changes to the data entered into the data capture system will be compliant to FDA CFR 21 Part 11.

Medical coding will use Medical Dictionary for Regulatory Activities for AEs.

Missing or inconsistent data will be queried to the investigator for clarification. Subsequent modifications to the database will be documented.

12 RECORDS AND SUPPLIES

12.1 Drug Accountability

On receipt of the study agent (including rescue medication, if relevant), the investigator (or designee) will conduct an inventory of the supplies and verify that study agent supplies are received intact and in the correct amounts before completing a supplies receipt. The investigator will retain a copy of this receipt at the study center and return the original receipt to the study monitor. The monitor may check the study supplies at each study center at any time during the study.

It is the responsibility of the study monitor to ensure that the investigator (or designee) has correctly documented the amount of the study agent received, dispensed, and returned on the dispensing log that will be provided. A full drug accountability log will be maintained at the study center at all times. The study monitor will arrange collection of unused study agent returned by the subject. The study monitor will also perform an inventory of study agent at the close-out visit to the study center. All discrepancies must be accounted for and documented.

12.2 Financing and Insurance

Financing and insurance of this study will be outlined in a separate agreement between the contract research organization and the sponsor.

13 ETHICS

13.1 Independent Ethics Committee or Institutional Review Board

Before initiation of the study at each study center, the protocol, the ICF, other written material given to the subjects, and any other relevant study documentation will be submitted to the appropriate IEC/IRB. Written approval of the study and all relevant study information must be obtained before the study center can be initiated or the study agent is released to the investigator. Any necessary extensions or renewals of IEC/IRB approval must be obtained for changes to the study such as amendments to the protocol, the ICF or other study documentation. The written approval of the IEC/IRB together with the approved ICF must be filed in the study files.

The investigator will report promptly to the IEC/IRB any new information that may adversely affect the safety of the subjects or the conduct of the study. The investigator will submit written summaries of the study status to the IEC/IRB as required. On completion of the study, the IEC/IRB will be notified that the study has ended.

13.2 Regulatory Authorities

Relevant study documentation will be submitted to the regulatory authorities of the participating countries, according to local/national requirements, for review and approval before the beginning of the study. On completion of the study, the regulatory authorities will be notified that the study has ended.

13.3 Ethical Conduct of the Study

The investigator(s) and all parties involved in this study should conduct the study in adherence to the ethical principles based on the Declaration of Helsinki, cGCP, ICH guidelines, and the applicable national and local laws and regulatory requirements.

13.4 Informed Consent

The process of obtaining informed consent must be in accordance with applicable regulatory requirement(s) and must adhere to cGCP.

The investigator is responsible for ensuring that no subject undergoes any study related examination or activity before that subject has given written informed consent to participate in the study.

The investigator or designated personnel will inform the subject of the objectives, methods, anticipated benefits and potential risks and inconveniences of the study. The subject should be given every opportunity to ask for clarification of any points s/he does not understand and, if necessary, ask for more information. At the end of the interview, the subject will be given ample time to consider the study. Subjects will be required to sign and date the ICF. After signatures are obtained, the ICF will be kept and archived by the investigator in the investigator’s study file. A signed and dated copy of the subject ICF will be provided to the subject or their authorized representative.

It should be emphasized that the subject may refuse to enter the study or to withdraw from the study at any time, without consequences for their further care or penalty or loss of benefits to which

the subject is otherwise entitled. Subjects who refuse to give or who withdraw written informed consent should not be included or continue in the study.

If new information becomes available that may be relevant to the subject's willingness to continue participation in the study, a new ICF will be approved by the IEC(s)/IRB(s) (and regulatory authorities, if required). The study subjects will be informed about this new information and reconsent will be obtained.

13.5 Subject Confidentiality

Monitors, auditors, and other authorized agents of the sponsor and/or its designee, the IEC(s)/IRB(s) approving this research, and the United States (US) FDA, as well as that of any other applicable agency(ies), will be granted direct access to the study subjects' original medical records for verification of clinical study procedures and/or data, without violating the confidentiality of the subjects to the extent permitted by the law and regulations. In any presentations of the results of this study or in publications, the subjects' identity will remain confidential.

All personal data collected and processed for the purposes of this study should be managed by the investigator and his/her staff with adequate precautions to ensure confidentiality of those data, applicable to national and/or local laws and regulations on personal data protection.

14 REPORTING AND PUBLICATION, INCLUDING ARCHIVING

Essential documents are those documents that individually and collectively permit evaluation of the study and quality of the data produced. After completion of the study (end of study defined as the date of the last visit of the last subject), all documents and data relating to the study will be kept in an orderly manner by the investigator in a secure study file. This file will be available for inspection by the sponsor or its representatives. Essential documents should be retained for 2 years after the final marketing approval in an ICH region or for at least 2 years since the discontinuation of clinical development of the investigational product. It is the responsibility of the sponsor to inform the study center when these documents no longer need to be retained. The investigator must contact the sponsor before destroying any study related documentation. In addition, all subject medical records and other source documentation will be kept for the maximum time permitted by the hospital, institution, or medical practice.

The sponsor must review and approve any results of the study or abstracts for professional meetings prepared by the investigator(s). Published data must not compromise the objectives of the study. Data from individual study centers in multicenter studies must not be published separately.

15 REFERENCES

1. Hui DS, I Azhar E, Madani TA, et al. The continuing 2019-nCoV epidemic threat of novel coronaviruses to global health - The latest 2019 novel coronavirus outbreak in Wuhan, China. Int J Infect Dis. 2020;91:264-266.
2. Zhu N, Zhang D, Wang W, et al. A Novel Coronavirus from Patients with Pneumonia in China, 2019. N Engl J Med. 2020;382(8):727-733.
3. Guo YR, Cao QD, Hong ZS, et al. The origin, transmission and clinical therapies on coronavirus disease 2019 (COVID-19) outbreak - an update on the status. Mil Med Res. 2020;7(1):11. Published 2020 Mar 13.
4. Phan T. Novel coronavirus: From discovery to clinical diagnostics. Infect Genet Evol. 2020;79:104211.
5. Hoffmann M, Kleine-Weber H, Schroeder S, et al. SARS-CoV-2 Cell Entry Depends on ACE2 and TMPRSS2 and Is Blocked by a Clinically Proven Protease Inhibitor. Cell. 2020;181(2):271-280.e8.
6. Huang C, Wang Y, Li X, et al. Clinical features of patients infected with 2019 novel coronavirus in Wuhan, China [published correction appears in Lancet. 2020 Jan 30]. Lancet. 2020;395(10223):497-506.
7. Remy V, Largeron N, Quilici S, Carroll S. The Economic Value of Vaccination: Why Prevention Is Wealth. Value Health. 2014;17(7):A450.
8. Takashima Y, Osaki M, Ishimaru Y, Yamaguchi H, Harada A. Artificial molecular clamp: a novel device for synthetic polymerases. Angew Chem Int Ed Engl. 2011;50(33):7524-7528.
9. Liu H, Su D, Zhang J, et al. Improvement of Pharmacokinetic Profile of TRAIL via Trimer-Tag Enhances its Antitumor Activity in vivo. Sci Rep. 2017;7(1):8953.
10. Jiang S. Don't rush to deploy COVID-19 vaccines and drugs without sufficient safety guarantees. Nature. 2020;579(7799):321.
11. Peeples L. News Feature: Avoiding pitfalls in the pursuit of a COVID-19 vaccine. Proc Natl Acad Sci U S A. 2020;117(15):8218-8221.
12. Grifoni A, Weiskopf D, Ramirez SI, et al. Targets of T Cell Responses to SARS-CoV-2 Coronavirus in Humans with COVID-19 Disease and Unexposed Individuals. Cell. 2020;S0092-8674(20)30610-3.
13. Centers for Disease Control and Prevention. Cases and Deaths in the U.S. https://www.cdc.gov/coronavirus/2019-ncov/cases-updates/us-cases-deaths.html. Published 2020. Updated June 24, 2020. Accessed June 25, 2020.
14. Charlson M E, Pompei P, Ales K L, et al. A new method of classifying prognostic. comorbidity in longitudinal studies: development and validation. J Chronic Dis, 1987;40(5):373-83.
15. Barbara L., Miriam S. Priority List of Adverse Events of Special Interest: COVID-19. Safety Platform for Emergency Vaccines. V1.1, 05 March 2020
16. COVID-19 manifestations associated with more severe presentation and decompensation with

consideration of enhanced disease potential. The current listing is based on the Coalition for Epidemic Preparedness Innovations/Brighton Collaboration Consensus Meeting (12/13 March 2020) and expected to evolve as evidence accumulates (Lambert 2020)

17. Cytokine release syndrome related to COVID-19 infection is a disorder characterized by nausea, headache, tachycardia, hypotension, rash, and/or shortness of breath (DAIDS,2017)
