# Supplementary material for: Toward a pan-SARS-CoV-2 vaccine targeting conserved epitopes on spike and non-spike proteins for potent, broad and durable immune responses
Source: PLoS Pathog. 2023 Apr 20;19(4):e1010870. doi: 10.1371/journal.ppat.1010870 (PMC10153712; doi:10.1371/journal.ppat.1010870)
Supplement: S2 Appendix — (PDF) [file ppat.1010870.s015.pdf]

S2 Supplemental Appendix

Phase-2 study V-205 IRB approval letters

中國醫藥大學附設醫院
CHINA MEDICAL UNIVERSITY HOSPITAL
台中市北區育德路2號
2 Yude Road, Taichung, 40447, Taiwan (R.O.C.)
TEL : 886-4-22052121

中國醫藥大學暨附設醫院研究倫理委員會

Tel: 886-4-22052121 ext: 1925 Fax: 886-4-2207-1478 台中市北區育德路 2 號

計畫修正案通過證明書

計畫名稱：一個評估 UB-612 疫苗對於新型冠狀病毒於青少年、成年和老年健康受試者的免疫原性、安全性與耐受性的第二期、安慰劑控制、隨機分派、觀察者盲性臨床試驗

計畫編號/本會編號：V-205 / CMUH109-REC1-176(AR-5)

計畫主持人：兒童感染科黃高彬主治醫師

試驗機構：中國醫藥大學附設醫院

原計畫通過日期：2020 年 12 月 21 日至 2021 年 12 月 20 日

修正案通過日期：2021 年 09 月 21 日至 2021 年 12 月 20 日

計畫書：Version 3.0, Date: 06 September, 2021

中文摘要：Version 3.0, Date: 2021-09-06

英文摘要：Version 3.0, Date: 06 September, 2021

受試者同意書(成年免疫組)：Version 3.0, Date: Sep. 06, 2021

受試者同意書(青少年免疫組)：Version 3.0, Date: Sep. 06, 2021

受試者同意書(成年安全確認組)：Version 3.0, Date: Sep. 06, 2021

受試者同意書(青少年安全確認組)：Version 3.0, Date: Sep. 06, 2021

受試者同意書 (成年免疫第三劑組)：Version 3rd dose 1.0, Date: 20210906

受試者同意書 (青少年免疫第三劑組)：Version 3rd dose 1.0, Date: 20210906

受試者同意書 (成年安全確認第三劑組)：Version 3rd dose 1.0, Date: 20210906

受試者同意書 (青少年安全確認第三劑組)：Version 3rd dose 1.0, Date: 20210906

個案報告表：Version 4.0, Date: 06 September, 2021

The Committee is organized and operates in accordance with ICH6 GCP regulations and guideline.
本委員會組織與運作皆遵守 ICH6 GCP 規定

中國醫藥大學附設醫院
CHINA MEDICAL UNIVERSITY HOSPITAL
台中市北區育德路2號
2 Yude Road, Taichung, 40447, Taiwan (R.O.C.)
TEL : 886-4-22052121

中國醫藥大學暨附設醫院研究倫理委員會

Tel: 886-4-22052121 ext: 1925 Fax: 886-4-2207-1478 台中市北區育德路2號

上述計畫已於2021年09月15日經中國醫藥大學暨附設醫院研究倫理委員會第一審查委員會2021年第10次審查會議審查。本委員會的運作符合優良臨床試驗準則及國內相關法令。請在持續審查必須進行前二個月向本會檢送完整之期中報告。

此計畫任何部分若經更改，必須在執行前重新提交本會審查及核准。此外，計畫主持人必須依時通報嚴重不良事件及涉及受試者或其他人風險的非預期問題。

中國醫藥大學暨附設醫院 研究倫理委員會 台中市北區育德路2號

主任委員 傅茂祖

中華民國一一〇年九月二十八日

中國醫藥大學附設醫院
CHINA MEDICAL UNIVERSITY HOSPITAL
台中市北區育德路2號
2 Yude Road, Taichung, 40447, Taiwan (R.O.C.)
TEL : 886-4-22052121

Research Ethics Committee
China Medical University & Hospital, Taichung, Taiwan

Tel: 886-4-22052121 ext: 1925 Fax: 886-4-2207-1478

Clinical Trial/Human Research Approval

Amendment Review

Date：Sep. 28, 2021

Protocol Title：A Phase II, Placebo-controlled, Randomized, Observer-blind Study to Evaluate the Immunogenicity, Safety and Tolerability of UB-612 Vaccine against COVID-19 in Adolescent, Younger and Elderly Adult Volunteers

Protocol No. / CMUH REC No.：V-205 / CMUH109-REC1-176(AR-5)

Name of Principal Investigator：Kao-Pin Hwang (Attending Physician, Pediatric Infectious Diseases)

Name of Institution：China Medical University Hospital

Valid Date of Original Research Project: From Dec. 21, 2020 to Dec. 20, 2021

Valid Date of Amended Research Project: From Sep. 21, 2021 to Dec. 20, 2021

Protocol：Version 3.0, Date: 06 September, 2021

Chinese Synopsis：Version 3.0, Date: 2021-09-06

English Synopsis：Version 3.0, Date: 06 September, 2021

Informed Consent Form (Immunogenicity Group-Adult)：Version 3.0, Date: Sep. 06, 2021

Informed Consent Form (Immunogenicity Group-Adolescent)：Version 3.0, Date: Sep. 06, 2021

Informed Consent Form (Safety Check Group-Adult)：Version 3.0, Date: Sep. 06, 2021

Informed Consent Form (Safety Check Group-Adolescent)：Version 3.0, Date: Sep. 06, 2021

Informed Consent Form (3rd Dose Immunogenicity Group-Adult)：Version 3rd dose 1.0, Date: 20210906

Informed Consent Form (3rd Dose Immunogenicity Group-Adolescent)：Version 3rd dose 1.0, Date: 20210906

CMUH

中國醫藥大學附設醫院
CHINA MEDICAL UNIVERSITY HOSPITAL
台中市北區育德路2號
2 Yude Road, Taichung, 40447, Taiwan (R.O.C.)
TEL : 886-4-22052121

Research Ethics Committee

China Medical University & Hospital, Taichung, Taiwan

Tel: 886-4-22052121 ext: 1925 Fax: 886-4-2207-1478

Informed Consent Form (3rd Dose Safety Check Group-Adult)：Version 3rd dose 1.0, Date: 20210906

Informed Consent Form (3rd Dose Safety Check Group-Adolescent)：Version 3rd dose 1.0, Date: 20210906

Case Report Form：Version 4.0, Date: 06 September, 2021

This is to certify that the above referenced amended research project has been reviewed by the 2021 10th meeting of the Research Ethics Committee (REC) I of the China Medical University and Hospital on Sep. 15, 2021. The REC is organized under, and operates in accordance with, the Good Clinical Practices guidelines and the governmental laws and regulations. Please submit a completed progress report at least two months before the time at which continuing review must occur.

All the amendments to the research project should be re-submitted and approved by the REC BEFORE implementation. Also, the principal investigator is required to report all serious adverse events and unanticipated problems involving risks to the subjects or others on time.

CHINA MEDICAL UNIVERSITY & HOSPITAL
The Research Ethics Committee
NO. 2 Yude Road, Taichung, Taiwan, R.O.C.

Martin M-T Fuh MD, DMSci.
Chairman, Research Ethics Committee I
China Medical University & Hospital

臺北醫學大學

臺北醫學大學暨附屬醫院聯合人體研究倫理委員會

TMU-Joint Institutional Review Board

通過證明函 – 修正案(簡易審查-實質變更)

開立日期：民國110年10月07日

本會編號：N202102039

計畫名稱：一個評估UB-612疫苗對於新型冠狀病毒於青少年、成年和老年健康受試者的免疫原性、安全性與耐受性的第二期、安慰劑控制、隨機分派、觀察者盲性臨床試驗

計畫主持人：李垣樟

共同主持人：劉明哲、邱仲峯、張君照、曾頌惠、許信德、吳建志、李欣倫、黃姚儒、周百謙、鍾啟禮、徐上富、蕭世欣、林哲玄、黃宇銳、侯甚光、陳揚卿、莊涵琄、羅詩修、陳偉傑、劉欣怡

研究人員：周鳳儀、李盈蓁、余嘉莉、黃金燕、王昌弘、丁充逢、李心平、黃珮妤、蔡家榛、蔡宛均、劉家豪、鄭錦龍、陳美雪、楊純弦、王凱立、巫怡蓮、連瑋豪、林穎、張玟惠、王楚君、廖嘉甄、陳蘊書、張家綺、蔡宛玲、謝俊宇、涂儷蓉、賴珍伶、謝清心、徐珮娟、方敏晏

試驗/研究機構：臺北醫學大學附設醫院

申請書版本/日期：Version 3.2 Date:20210930

計畫書版本/日期：Version 3.0, Date: 20210906

中文摘要：Version 3.0, Date: 20210906

受試者同意書版本/日期：

- (成年免疫組)：Version 2.0, Date:20210914
- (青少年免疫組)：Version 2.0, Date:20210914
- (成年安全確認組)：Version 2.0, Date:20210914
- (青少年安全確認組)：Version 2.0, Date:20210914
- (成年免疫第三劑組)：Version 1.0, Date:20210914
- (青少年免疫第三劑組)：Version 1.0, Date:20210914
- (成年安全確認第三劑組)：Version 1.0, Date:20210914
- (青少年安全確認第三劑組)：Version 1.0, Date:20210914

個案報告表版本/日期：Version 4.0, Date: 20210906

緊急解盲指引：Version 1.2, Date:20210722

Note to File：Date: 20210324

上述計畫之修正，將於第110-10-4次會期核備(會議日期：110年10月21日)，特此證明。有效期限自民國110年10月07日至民國111年02月16日。試驗/研究期間應接受本會之監督。

臺北醫學大學暨附屬醫院聯合人體研究倫理委員會
Taipei Medical University
Joint Institutional Review Board

本會組織與執行皆符合適用法規
The TMU-Joint Institutional Review Board performs its functions according to written operating procedures and complies with GCP and with the applicable regulatory requirements.

依據衛生福利部與相關規定， 後續追蹤程序及要求如下：

1. 期中報告：本計畫期中繳交頻率為**每6個月**，應於有效期限到期**前二個月（民國110年12月16日）**繳交期中報告。有效期限屆滿時若尚未通過期中報告與效期展延審查者，試驗/研究不得繼續執行。
2. 結案報告：試驗/研究完成後，應將執行情形及結果依結案報告表要求送至本會審查。**核准期間到期三個月**仍未繳交者，**本會得撤銷本通過證明函，亦即撤銷本試驗/研究之核准，亦將依本會作業程序暫停主持人(含任何參與形式)申請新試驗/研究案之審查三個月**。
3. 嚴重不良事件(SAE)報告：執行人體試驗或臨床試驗之主持人應根據衛生福利部「藥品優良臨床試驗準則」和「嚴重藥物不良反應通報辦法」規定，辦理相關事宜。

主任委員：

陳中明

臺北醫學大學暨附屬醫院
聯合人體研究倫理委員會
Taipei Medical University
Joint Institutional Review Board

本會組織與執行皆符合適用法規
The TMU-Joint Institutional Review Board performs its functions according to written operating procedures and complies with GCP and with the applicable regulatory requirements.

Taipei Medical University

Certificate of TMU-JIRB Approval

Issue Date: 2021/10/07

TMU-JIRB No.: N202102039

Protocol Title: A Phase II, Placebo-controlled, Randomized, Observer-blind Study to Evaluate the Immunogenicity, Safety and Tolerability of UB-612 Vaccine against COVID-19 in Adolescent, Younger and Elderly Adult Volunteers

Principal Investigator: Yuarn-Jang Lee

CO- Investigator: Mingche Liu, Jeng-Fong, Chiou, Chun-Chao CHANG, TSENG,SUNG-HUI, Hsu, Hsin-Te, WU CHIEN-CHIH, Lee, Hsin-Lun, Yaoru Huang, Pai-Chien Chou, CHI-LI CHUNG, Shang-Fu Hsu, SHIH-HSIN HSIAO, Lin, Che-Hsuan, Yu-Jui Huang, Hou, Sen-Kuang, Chen YangChing, Chuang HanChuan, Lo shih hsiu, Chen, WeiChieh, Hsin-Yi LIU

Study Member: Fengyi Chou, Ying-Chen Lee, CHIA-LI YU, CHIN-YEN HUANG, Chang-Hung Wang, CHUNG-FENG TING, Sin Ping Lee, peiyuhuang, tsai chia chen, Wan-Chun Tsai, Chia-Hao, Liu, Chin-Lung Cheng, Mei-Syue, Chen, Chun-Hsien Yang, WANG KAI LI, WU YI LIEN, LIEN WEI HAO, Ying Lin, WEN-HUICHANGE, NO, Liao chian chen , YUN-SHU CHEN , Chia-Chi,Chang, Wan-Ling Tsai, Chun-Yu Hsieh, Tu LI Jung, Jhen-Ling Lai, HSIEH CHING HSIN, Pei Chuan Hsu, min yan hang

Study Site:Taipei Medical University Hospital

Application Form:Version 3.2 Date:20210930

Protocol Version/Date:Version 3.0, Date: 20210906

Chinese Synopsis：Version 3.0, Date: 20210906

Informed Consent Forms:

- (Immunogenicity Group -Adult):Version 2.0, Date:20210914
- (Immunogenicity Group -Adolescent):Version 2.0, Date:20210914
- (Safety Check Group -Adult):Version 2.0, Date:20210914
- (Safety Check Group -Adolescent):Version 2.0, Date:20210914
- (3rd Dose Immunogenicity Group -Adult):Version 1.0, Date:20210914
- (3rd Dose Immunogenicity Group -Adolescent):Version 1.0, Date:20210914
- (3rd Dose Safety Check Group -Adult):Version 1.0, Date:20210914
- (3rd Dose Safety Check Group -Adolescent):Version 1.0, Date:20210914

Case Report Forms:Version 4.0, Date: 20210906

Emergency Code Breaking Instruction：Version 1.2, Date:20210722

Note to File：Date: 20210324

The amendment of above study will be approve by the TMU-Joint Institutional Review Board in meeting #110-10-4(date:2021/10/21), duration of validity is from 2021/10/07 to 2022/02/16, and must be monitored by TMU-JIRB.

臺北醫學大學暨附屬醫院
聯合人體研究倫理委員會
Taipei Medical University
Joint Institutional Review Board

According to Ministry of Health and Welfare and the relevant regulations, follow-up procedures and requirements are as below:

1. Continuing Report: Frequency of the report of this trial/study is every 6 month, and should be submitted to TMU-JIRB for review 2 months prior to expiry date (2021-12-16) or the trial/study must be pending.
2. Final Report: The report should be submitted to TMU-JIRB for review once completed TMU-JIRB may withdraw the approval of the trial/study if the report didn't submitted within 3 months from the date of validity and will suspend PI from new application for 3 months per TMU-JIRB SOPs.
3. SAE: Serious Adverse Event(s) (SAE) Report: SAE report(s) should be submitted to related authorities according to "Regulations for Good Clinical Practice" as well as "Procedures for Reporting Serious Adverse Drug Reaction" by MOHW.

Chairman:

臺北醫學大學暨附屬醫院
聯合人體研究倫理委員會
Taipei Medical University
Joint Institutional Review Board

醫療財團法人徐元智先生醫藥基金會
亞東紀念醫院人體試驗審議委員會
Research Ethics Review Committee
Far Eastern Memorial Hospital
21, Sec. 2, Nanya S. Rd., Banciao Dist., New Taipei City 220, Taiwan (R.O.C.)
Tel：(02)7728-2152　Fax：(02)7728-1592
Email：irb@mail.femh.org.tw

試驗/研究變更許可書

案件編號：110025-I　　　　　計畫編號：V-205

計畫名稱：一個評估 UB-612 疫苗對於新型冠狀病毒於青少年、成年和老年健康受試者的免疫原性、安全性與耐受性的第二期、安慰劑控制、隨機分派、觀察者盲性臨床試驗

試驗/研究機構：醫療財團法人徐元智先生醫藥基金會亞東紀念醫院

試驗/研究委託者：聯亞生技開發股份有限公司

計畫主持人：廖俊星

協同主持人：陳泓恩、楊家瑞

研究成員：徐心穗、張嫚萱

試驗/研究人數：400

試驗/研究期限：2021 年 2 月 23 日至 2022 年 12 月 31 日

追蹤審查頻率：六個月

有效期限至二○二二年二月二十二日。(請於有效期限到期二個月前繳交持續審查報告以利本會進行審查)

主任委員 **張淑雯**

Permission of continuing review of Clinical Trial/ Research

FEMH No.: 110025-I　　　　　Protocol No.: V-205

Protocol Title: A Phase II, Placebo-controlled, Randomized, Observer-blind Study to Evaluate the Immunogenicity, Safety and Tolerability of UB-612 Vaccine against COVID-19 in Adolescent, Younger and Elderly Adult Volunteers

Trial/Research Institution: Far Eastern Memorial Hospital

Sponsor: United Biomedical, Inc., Asia

Principal investigator: Chun-Hsing Liao

Sub- investigator: Hong-An Chen, Chia-Jui Yang

Study Coordinator: Hsin-Sui Hsu, Man-Hsuan Chang

Number of subjects: 400

Trial period: February 23, 2021 to December 31, 2022

Continuing review frequency: Six months

The protocol is valid till February 22, 2022. (The investigator is required to apply for a continuing review

核准日期/ Approved date：2021-11-10

本委員會的運作符合優良臨床試驗準則及政府相關法律規章
The committee is organized under, and operates in accordance with, the Good Clinical Practice guidelines and governmental laws and regulations

醫療財團法人徐元智先生醫藥基金會 亞東紀念醫院 人體試驗審議委員會 TEL.(02)7728-2152

not less than two months prior to approval expiration date.)
Shu-Wen Chang M.D., Professor of Ophthalmology
Chairman
Research Ethics Review Committee

修正內容/版本 Reason for Amendment/Version:

- 計畫書版本 Protocol Version: Version 3.0, Date: 20210906
- 受試者說明及同意書版本 Informed Consent Form:
  (1.) 青少年免疫組 Immno young：Version 2.0, Date: 20211021
  (2.) 青少年安全確認組 non-Immno young：Version 2.0, Date: 20211021
  (3.) 成年免疫組 Immno adult：Version 2.0, Date: 20211021
  (4.) 成年安全確認組 non-Immno adult：Version 2.0, Date: 20211021
- 中文摘要 Chinese synopsis: Version 3.0, Date: 20210906
- 英文摘要 English synopsis: Version 3.0, Date: 20210906
- 個案報告表 Case report form: Version 4.0, Date: 20210906
- 新增受試者說明及同意書版本 Add Informed Consent Form:
  (1.) 青少年免疫第三劑組 Immno young 3rd dose：Version 3rd dose 1.0, Date: 20211013
  (2.) 青少年安全確認第三劑組 non-Immno young 3rd dose：Version 3rd dose 1.0, Date: 20211013
  (3.) 成年免疫第三劑組 Immno adult 3rd dose：Version 3rd dose 1.0, Date: 20211013
  (4.) 成年安全確認第三劑組 non-Immno adult 3rd dose：Version 3rd dose 1.0, Date: 20211013

已通過其它合法審查會之審查案件，符合得簡易程序審查範圍，不予入會
The protocol fits the criteria for Expedited Review and do not need to be discussed in this meeting.

上述計畫已於本院人體試驗審議委員會審查，同意人體試驗/研究進行
The protocol has been approved by the Research Ethics Review Committee of the Far Eastern Memorial Hospital.

核准日期/ Approved date：2021-11-10

本委員會的運作符合優良臨床試驗準則及政府相關法律規章
The committee is organized under, and operates in accordance with,
the Good Clinical Practice guidelines and governmental laws and regulations

醫療財團法人徐元智先生醫藥基金會 亞東紀念醫院 人體試驗審議委員會 TEL:(02)7728-2152

Institutional Review Board
National Cheng Kung University Hospital
138 Sheng-Li Rd, Tainan 704, Taiwan R.O.C.
TEL:886-6-2353535 ext.3635 FAX:886-6-2388190

國立成功大學醫學院附設醫院 A119(3)
第一人體研究倫理審查委員會
台灣.台南市勝利路138號
E-mail:em73635 @mail.hosp.ncku.edu.tw

同意計畫修正證明書

研究計畫名稱：一個評估 UB-612 疫苗對於新型冠狀病毒於青少年、成年和老年健康受試者的免疫原性、安全性與耐受性的第二期、安慰劑控制、隨機分派、觀察者盲性臨床試驗

計畫編號/本會編號: V-205 / AB-CR-110-011

研究執行期間:民國 110 年 02 月 23 日至民國 111 年 12 月 31 日

本次核准期間:民國 110 年 02 月 23 日至民國 111 年 02 月 22 日

核准內容/版本：

1. 計畫書：Version 3.0, Date: 20210906
2. 中文摘要：Version 3.0, Date: 20210906
3. 英文摘要：Version 3.0, Date: 20210906
4. 受試者同意書(成年免疫組)：Version 2.0, Date:20210916
5. 受試者同意書(青少年免疫組)：Version 2.0, Date:20210916
6. 受試者同意書(成年安全確認組)：Version 2.0, Date:20210916
7. 受試者同意書(青少年安全確認組)：Version 2.0, Date:20210916
8. 個案報告表：Version 4.0, Date: 20210906
9. 新增送審文件：
   - 受試者同意書(成年免疫第三劑組)：Version 3rd dose 1.0, Date: 20210916
   - 受試者同意書(青少年免疫第三劑組)：Version 3rd dose 1.0, Date: 20210916
   - 受試者同意書(成年安全確認第三劑組)：Version 3rd dose 1.0, Date: 20210916
   - 受試者同意書(青少年安全確認第三劑組)：Version 3rd dose 1.0, Date: 20210916
10. 變更協同主持人：郭正彥醫師退出，新增王冠元醫師

試驗機構：成大醫院

研究計畫主持人：楊宜青主任(社區健康照護中心)

協同主持人：柯文謙醫師、楊登棋醫師、劉介修醫師、羅玉岱醫師、周佑聰醫師、陳泓裕醫師、蘇斐琳醫師、吳怡萱醫師、周杰穎醫師、鄭翔如醫師、廖信閔醫師、王竣令醫師、林筱茹醫師、吳晉祥醫師、謝奇璋醫師、沈靜芬醫師、蔡孟哲醫師、林亭妤醫師、王冠元醫師

協同研究員：施譪衿研究助理、郭筱薇研究助理、賴虹樺研究助理、陳彥如研究助理、王端玲研究助理、姜美如研究助理

本會經中央衛生主管機關查核通過，組織與執行皆遵照法令及主管機關規範。

本修正計畫已於**民國 110 年 10 月 27 日**經本院人體研究倫理審查委員會審核通過，本次核准執行期間至**民國 111 年 02 月 22 日**，特此證明。

已完成之研究應於研究執行期間末日後三個月內繳交結案報告，除維護受試者安全之必要作為外，於核准期間末日後應停止執行所有受試者相關之研究程序。

計畫主持人逾核准期間末日仍未繳交報告者，列入逾期名單，本會將寄發本研究案之中止/終止通知書。逾期名單將提本會審查會議報告，經會議決議後，本會將暫停受理名單上人員所主持之新案審查申請，迨繳交應繳報告並經本會會議審查通過後，始得受理其新案審查申請。

追蹤/結案報告請以書面繳交；報告書請逕送本院人體研究倫理審查委員會辦公室；報告表格最新版本請至本會網頁(http://nckuhirb.med.ncku.edu.tw/)下載。

研究計畫內容有任何變更或修正(含研究執行期間變更)，須於研究執行期間內向本會提出申請，本會不受理未在研究執行期間內提出之變更或修正案。變更或修正未獲本會核准前，須依原核准範圍執行。

已獲本會同意之研究案，因故未開始執行或不繼續執行者，應申請中止/終止。

不論研究進行中或研究完成後，受試者若發生任何不良反應，須依 GCP 規範通報。

此致

國立成功大學醫學院附設醫院
人體研究倫理審查委員會
主任委員 張俊彥

中 華 民 國 110 年 10 月 28 日

Institutional Review Board
National Cheng Kung University Hospital
138 Sheng-Li Rd, Tainan 704, Taiwan R.O.C.
TEL:886-6-2353535 ext.3635 FAX:886-6-2388190

文件編號：8800-4-07-002

國立成功大學醫學院附設醫院 A119(3)
第一人體研究倫理審查委員會
台灣.台南市勝利路138號
E-mail:em73635 @mail.hosp.ncku.edu.tw

Human Study Amendment Approval

Date:2021.10.28

Title: A Phase II, Placebo-controlled, Randomized, Observer-blind Study to Evaluate the Immunogenicity, Safety and Tolerability of UB-612 Vaccine against COVID-19 in Adolescent, Younger and Elderly Adult Volunteers
Protocol No/ IRB No: V-205 / AB-CR-110-011
Period of Project: From 2021.02.23 to 2022.12.31
Period of Approval: From 2021.02.23 to 2022.02.22
Content/Version:

1. Protocol: Version 3.0, Date: 20210906
2. Chinese synopsis: Version 3.0, Date: 20210906
3. English synopsis: Version 3.0, Date: 20210906
4. Informed Consent Form (Immunogenicity group -Adult): Version 2.0, Date:20210916
5. Informed Consent Form (Immunogenicity group -Adolescent): Version 2.0, Date:20210916
6. Informed Consent Form (Safety check group -Adult): Version 2.0, Date:20210916
7. Informed Consent Form (Safety check group -Adolescent): Version 2.0, Date:20210916
8. Case Report Form: Version 4.0, Date: 20210906
9. Add Submission Documents:
   - Informed Consent Form (3rd dose Immunogenicity group -Adult): Version 3rd dose 1.0, Date: 20210916
   - Informed Consent Form (3rd dose Immunogenicity group -Adolescent): Version 3rd dose 1.0, Date: 20210916
   - Informed Consent Form (3rd dose Safety check group -Adult): Version 3rd dose 1.0, Date: 20210916
   - Informed Consent Form (3rd dose Safety check group -Adolescent): Version 3rd dose 1.0, Date: 20210916
10. Amend Co-Investigator: Withdraw Dr. Cheng-Yen Kuo, Add Dr. Kuan-Yuan Wang

Institute: National Cheng Kung University Hospital
Investigator: Director Yi-Ching Yang (Community Healthcare Center)
Sub-Investigator: Dr. Wen-Chien Ko, Dr. Deng-Chi Yang, Dr. Chieh-Hsiu Liu, Dr. Yu-Tai Lo, Dr. Yu-Tsung Chou, Dr. Hung-Yu Chen, Dr. Fei-Lin Su, Dr. I-Hsuan Wu, Dr. Chieh-Ying Chou, Dr. Hsiang-Ju Cheng, Dr. Jiun-Ling Wang, Dr. Shin-Ming Liao, Dr. Hsiao-Ju Lin, Dr Jin-Shang Wu, Dr. Chi-Chang Shieh, Dr. Ching-Fen Shen, Dr.Meng-Che Tsai, Dr. Ting-Yu Lin, Dr. Kuan-Yuan Wang
Co-Researcher: Research Assistant Hui-Chin Shih, Research Assistant Hsiao-Wei Kuo, Research Assistant Hung-Hua Lai, Research Assistant Yen-Ju Chen, Research Assistant Tuan-Ling Wang, Research Assistant Mei-Ru Chiang

The Institutional Review Board of National Cheng Kung University Hospital (NCKUH) is organized and operated according to the laws and regulations of ICH-GCP and of Central Competent Authorities.
This project is reviewed and approved by NCKUH IRB in **2021.10.27**. The period of approval is granted until **2022.02.22**.
Regarding completed project, the Final Report shall be submitted within three months of its approved expiry date. Except for the health of the participants, all the procedures of the project shall be terminated on its approved stated deadline.
If PI does not submit the Interim/Final Report on time, he/she will be recorded in the overdue list and received the suspension/ termination notice from NCKUH IRB. The overdue list will be reported to the IRB. After the resolution of the board meeting, NCKUH IRB will suspend all the new projects applied by PI until the Interim/Final Report is submitted.
Please submit the Interim/Final Report in written form and send to NCKUH IRB office. The lastest application forms can be downloaded in its website (http://nckuhirb.med.ncku.edu.tw/)
Any changes or amendments to the project (including the project period), please submit an amendment application to NCKUH IRB within its approved period. Any changes or amendments in any other way will not be accepted. Before the approval of the amendment application, the project is carried out according to its previously approved plan.
For some reasons projects granted approval by NCKUH IRB couldn't be implemented, PI shall apply for suspension/termination.
During or after the project is completed, please report any unfavorable occurrence in a human study participant according to GCP.

Yours sincerely,
Ting-Tsung Chang M.D.
Chairman

Institutional Review Board
National Cheng Kung University Hospital

經第 B053 次大會通過版

長庚醫療財團法人人體試驗倫理委員會

臨床試驗/研究同意證明書

地　　址：105台北市敦化北路199號
傳　　真：03-3494549
聯絡人及電話：薛育婷(03)3196200#3703
電子郵件信箱：sally6869@cgmh.org.tw

計畫名稱： 一個評估UB-612疫苗對於新型冠狀病毒於青少年、成年和老年健康受試者的免疫原性、安全性與耐受性的第二期、安慰劑控制、隨機分派、觀察者盲性臨床試驗

計畫編號： V-205

本院案號： 202100188A4C602

試驗期間： 2021年2月20日~2023年1月5日

本次核准執行期間：2021年10月12日~2022年2月19日

總主持人： 感染醫學科 黃景泰 學術組教授級主治醫師

主持人： 感染醫學科 李禎祥 學術組教授級主治醫師

協同主持人： 葉峻甫,李允吉,張鵬浩,黃文琦,黃柏諺,蘇庭儀,劉仲淇,鄭鈞文,陳怡君,謝顯森,吳彥穆,蔡青晏,郭泓頡

研究助理： 温鵬毅,王筠婷,李兆芸,洪慧雯,孫秀琪,莊惠姍,張芳瑋,莊培瑜,黃惠琳,莊淑婷,王儀鳳,郭筱音,王思怡,陳立君,陳品蓁,康瑋庭,吳珮嘉,丁秋煥,陳美真,趙潔欣,劉靜芬,林奕臻,許淑慧,劉清華,林奕君

試驗/研究機構： 林口長庚醫院,高雄長庚醫院

計畫文件版本日期：

(1)計畫書： Version 3.0, Date: 06 September, 2021

(2)中文摘要： Version 3.0, Date: 2021-09-06

(3)英文摘要： Version 3.0, Date: 06 September, 2021

(4)受試者同意書：
受試者同意書(成年安全確認第三劑組林口院區)：2021/09/16 Version 3rd dose 1.0
受試者同意書(成年安全確認第三劑組高雄院區)：2021/09/16 Version 3rd dose 1.0
受試者同意書(成年免疫第三劑組林口院區)：2021/09/16 Version 3rd dose 1.0
受試者同意書(青少年安全確認第三劑組高雄院區)：2021/09/16 Version 3rd dose 1.0
受試者同意書(青少年安全確認第三劑組林口院區)：2021/09/18 Version 3rd dose 1.0
受試者同意書(青少年免疫第三劑組林口院區)：2021/09/18 Version 3rd dose 1.0
受試者同意書(成年安全確認組林口院區)：2021/09/16 Version 2.0
受試者同意書(成年免疫組林口院區)：2021/09/16 Version 2.0
受試者同意書(青少年安全確認組林口院區)：2021/09/18 Version 2.0
受試者同意書(青少年免疫組林口院區)：2021/09/18

Version 2.0
受試者同意書(成年安全確認組高雄院區)：2021/09/16
Version 2.0
受試者同意書(青少年安全確認組高雄院區)：2021/09/16
Version 2.0

(5)其他文件： 人體試驗申請表
19A 檢體資訊
檢體外送及儲存: 新增外送及儲存機構 NEXELIS (機構地址: 525 Boul. Cartier Ouest Laval, Qulbec, Canada, H7V 3S8 (加拿大))，Vaccinology and Immunology Infection, Immunity & Inflammation Dept UCL GOS Institute of Childe Health (機構地址: UCL Great Ormond Street Institute of Child Health 30 Guilford Street London WC1N 1EH, England (英國))，VisMederi (機構地址: VisMederi Srl, Strada del Petriccio e Belriguardo, 35, 53100 Siena, Italy (義大利))
研究助理-許美秀退出

※本案須重新簽署受試者同意書

通過日期：2021年10月12日

DSMB/DSMP：已有DSMP,已有其他安全監測

期中報告繳交頻率：半年繳交一次

※本試驗計劃案業經本院人體試驗倫理委員會審核通過，同意追認其他合法審查會通過之研究計畫之核可。

※下次期中報告繳交截止日期：2022年2月19日，為免影響主持人執行研究之權益，請於截止日前六至八週繳交報告(期中報告繳交頻率為三個月者，得於試驗到期前一個月繳交報告)，以利審查作業進行。若主持人未繳交或延遲繳交期中報告，以致本會無法於核准執行期間到期前，核發下次試驗執行期間，所有的研究活動必須停止，包括：對已參與受試者之介入或各項互動，除非本會認為受試者繼續接受試驗介入或參與試驗顯有益於受試者安全或倫理上符合受試者最佳利益之情形，亦不得再納入新個案，直到期中報告核准為止。

本委員會組織與運作皆遵守GCP規定

長庚醫療財團法人

人體試驗倫理委員會謝燦堂主席

2021 年 10 月 12 日

Chang Gung Medical Foundation

Institutional Review Board

199, TUNG HWA NORTH ROAD,
TAIPEI, TAIWAN, 10507
REPUBLIC OF CHINA
Tel: (03) 3196200
Fax: (03) 3494549

Date 2021/10/12

Protocol Title: Phase II, Placebo-controlled, Randomized, Observer-blind Study to Evaluate the Immunogenicity, Safety, and Tolerability of UB-612 Vaccine against COVID-19 in Adolescent, Younger and Elderly Adult Volunteers

Protocol No.: V-205

IRB No.: 202100188A4C602

Chief Investigator(s): HUANG,CHING-TAI

Principal Investigator(s): LEE-CHEN-HSIANG

Co-Investigator(s): YEH, CHUN-FU,LI-YUN-CHI,CHANG, PENG-HAO,WEN CHI HUANG,HUANG, PO-YEN,SU, TING-YI,LAO CHONG KEI,CHENG,CHUN-WEN,YI-CHUN CHEN,SHIE,SHIAN-SEN,WU YEN-MU,TSAI, CHING-YEN,KUO, HONG-JIE

Study Coordinator(s): Wen Pengyi,WANG,YUN-TING,LI CHAO-YUN,HUI-WEN HUNG,HSIU CHIU SUN,HUI-SHAN CHUANG,CHANG FANG-WEI,Pei-Yu Chuang,HUANG HUI-LING,CHUANG, SHU-TING,Yi-Feng Wang,KUO,HSIAO-YING,WANG SZU YI,Li Chun Chen,PIN-CHEN CHEN,KANG WEI-TING,PEI-JIA WU,DING CIOU-HUAN,MEI-CHEN, CHEN,,,liu ching fen,LIN,YI-JHEN,HSU,SHU-HUI,LIU CHING HUA,Yichun Lin

Executing Institution: Linkou,Kaohsiung

Duration of Approval: From 2021/10/12 To 2022/2/19

Version/Date of documents:
(1)Protocol: Version 3.0, Date: 06 September, 2021
(2)Chinese Synopsis: Version 3.0, Date: 2021-09-06
(3)English Synopsis: Version 3.0, Date: 06 September, 2021
(4)Informed Consent Form: 2021/09/16 Version 3rd dose 1.0
2021/09/16 Version 3rd dose 1.0
2021/09/16 Version 3rd dose 1.0
2021/09/16 Version 3rd dose 1.0
2021/09/18 Version 3rd dose 1.0
2021/09/18 Version 3rd dose 1.0
2021/09/16 Version 2.0
2021/09/16 Version 2.0
2021/09/18 Version 2.0
2021/09/18 Version 2.0
2021/09/16 Version 2.0
2021/09/16 Version 2.0

(5)Others: 人體試驗申請表
19A 檢體資訊
檢體外送及儲存: 新增外送及儲存機構 NEXELIS (機構地址: 525 Boul. Cartier Ouest Laval, Qulbec, Canada, H7V 3S8 (加拿大))，Vaccinology and Immunology Infection, Immunity & Inflammation Dept UCL GOS Institute of Childe Health (機構地址: UCL Great Ormond Street Institute of Child Health 30 Guilford Street London WC1N 1EH, England (英國))，VisMederi (機構地址: VisMederi Srl, Strada del Petriccio e Belriguardo, 35, 53100 Siena, Italy (義大利))
研究助理-許美秀退出

Date of Approval: 2021/10/12
DSMB/DSMP: Already Had DSMP,Already had other safety monit
Frequency of Continuing Report: Every 6 months

※The re-consent process shall be required

※The trial research has been reviewed and approved by Chang Gung Medical Foundation Institutional Review Board, and the research has already been approved by other legal Institutional Review Board shall be recognized.

※Next Deadline of Continuing Report: 2022/02/19. To facilitate the review, please submit the report two months before the deadline (or one month before the expiration of the trial if a continuing report shall be provided every three months) in order not to influence the principal investigator’ s right to conduct the research. In the case that failure or delay to submit a continuing report makes the IRB unable to determine the next trial period by the deadline, the trial shall not be continuously conducted.If the Principal Investigator fails to submit a continuing report on time, rendering the Institutional Review Board unable to issue the next trial execution period before the previous trial execution period expires, all research activities, including the intervention or interaction with the participating trial subjects, must be suspended. Unless the Institutional Review Board considers that the continuation of trial intervention or trial participation is greatly beneficial to the trial subject's safety or in the best interest of the trial subject from a moral point of view, no new trial subject shall be included until the continuing report is formally approved.

The IRB is organized and operates in accordance with Good Clinical Practice and the applicable laws and regulations.

Tsang-Tang Hsieh,MD
Chairman
Institutional Review Board
Chang Gung Medical Foundation

高雄醫學大學附設中和紀念醫院
Kaohsiung Medical University Chung-Ho Memorial Hospital
人體試驗審查委員會
Institutional Review Board

807高雄市三民區自由一路100號
No. 100, Tzyou 1st Road, Kaohsiung 807, Taiwan
TEL： 886-7-3121101 ext.6646、886-7-3133525
FAX： 886-7-3221408

人體研究變更案同意證明書

計畫中文名稱：一個評估UB-612疫苗對於新型冠狀病毒於青少年、成年和老年健康受試者的免疫原性、安全性與耐受性的第二期、安慰劑控制、隨機分派、觀察者盲性臨床試驗

計畫主持人：盧柏樑

共同及協同主持人：蔡明儒、陳彥旭、林俊祐、林尚儀、黃崇豪、張雅婷、羅世豪、李雋元、李純瑩、張家禎、許超群、洪仁宇、鄭孟軒、陳家閔、陳惇杰、李敏生、陳昭儒、蔡毓德、李杰明

研究人員：許晏禎、江玟靜、江如萍、黃建豪、胡嘉桂、李依鴻、李茉華

機構名稱：高雄醫學大學附設中和紀念醫院

經費來源：聯亞生技開發股份有限公司

本會編號：KMUHIRB-F(II)-20210029

計畫編號：V-205

核准日期(審查通過日)：2021/10/15

計畫執行期間：2021/2/19-2022/12/31

本同意書有效期限：2022/2/18

本次修正版本：

計畫書：Version 3.0, Date: 06 September, 2021

中文摘要：Version 3.0, Date: 06 September, 2021

英文摘要：Version 3.0, Date: 06 September, 2021

個案報告表：Version 4.0, Date: 06 September, 2021

受試者同意書：

(1) 成年安全確認組：Version 2.0, Date: 20210917

(2) 青少年安全確認組：Version 2.0, Date: 20210917

本次新增版本：

第三劑受試者同意書：

(1) 成年安全確認組：Version 3rd dose 1.0, Date:20210917

(2) 青少年安全確認組：Version 3rd dose 1.0, Date:20210917

凡經衛生福利部列管之計畫案件，變更案須取得衛生福利部審核同意，方可執行變更後內容，並請確實依衛生福利部核准並符合本院人體試驗審查委員會同意之各文件版本執行。未預期事件或藥品嚴重不良反應通報後續定期追蹤之程序及應注意事項，請參閱背面。

高雄醫學大學 人體試驗 審查委員會 附設中和紀念醫院

高雄醫學大學附設中和紀念醫院
第一人體試驗審查委員會
主任委員： 顏學偉

西元 2021 年 10 月 15 日

高雄醫學大學附設中和紀念醫院
Kaohsiung Medical University Chung-Ho Memorial Hospital
人體試驗審查委員會
Institutional Review Board

807高雄市三民區自由一路100號
No. 100, Tzyou 1st Road, Kaohsiung 807, Taiwan
TEL： 886-7-3121101 ext.6646、886-7-3133525
FAX： 886-7-3221408

Approval of Clinical Trial/Research

Protocol Title: *A Phase II, Placebo-controlled, Randomized, Observer-blind Study to Evaluate the Immunogenicity, Safety, and Tolerability of UB-612 Vaccine against COVID-19 in Adolescent, Younger and Elderly Adult Volunteers*

Principal Investigator: *Po-Liang Lu*

Co_Investigator(s): *Ming-Ju Tsai, Yen-Hsu Chen, Chun-Yu Lin, Shang-Yi Lin, Chung-Hao Huang, Ya-Ting Chang, Shih-Hao Lo, Chun-Yuan Lee, Chun-Ying Lee, Chai-Ja Chang, Chau-Chyun Sheu, Jen-Yu Hung, Meng-Hsuan Cheng, Chia-Min Chen, Tun-Chieh Chen, Min-Sheng Lee, Chao-Ju Chen, Yu-Te Tsai, Chieh-Ming Lee*

Study Coordinator: *Yan-Zhen Hsu, Wen-Ching Jiang, Ru-Ping Jiang, Chien-Hao Huang, Chia-Kuei Hu, Yi-Hong Li, Mo-Hua Li*

Institution: *Kaohsiung Medical University Chung-Ho Memorial Hospital*

Source of Funding: *United Biomedical, Inc., Asia*

IRB Number: *KMUHIRB-F(II)-20210029*

Protocol Number: *V-205*

Approval Date: *2021/10/15*

Duration of Approval: *2021/2/19- 2022/12/31*

Expiration Date of Approval: *2022/2/18*

Amendment:

Protocol: Version 3.0, Date: 06 September, 2021
Chinese Protocol Synopsis: Version 3.0, Date: 06 September, 2021
English Protocol Synopsis: Version 3.0, Date: 06 September, 2021
Informed Consent Form:
(1) Informed Consent Form (Safety check group -Adult): Version 2.0, Date: 20210917
(2) Informed Consent Form (Safety check group -Adolescent): Version 2.0, Date: 20210917
Case Report Form: Version 4.0, Date: 06 September, 2021

Add:

Informed Consent Form (3rd dose):
(1) Informed Consent Form 3rd dose (Safety check group –Adult): Version 3rd dose 1.0, Date:20210917
(2) Informed Consent Form 3rd dose (Safety check group –Adolescent: Version 3rd dose 1.0, Date:20210917

See the back of this page for the procedures for reporting unanticipated problems, or drug serious adverse reactions, or interim, and other important notes.

Hsueh-Wei Yen

Hsueh-Wei Yen, MD
Chairman
Institutional Review Board- I
Kaohsiung Medical University
Chung-Ho Memorial Hospital

Kaohsiung Medical University Institutional Review Board Chung-Ho Memorial Hospital

Tel: 886-2-87923311 ext 10552
Fax:886-2-2792-9201
E-mail: tsghirb@ndmctsgh.edu.tw

國防醫學院三軍總醫院
人體試驗審議會

台北市 11490 內湖區成功路二 325 號醫療大樓五樓 5113 室
No.325,Sec.2, Cheng-Kung Rd.
Neihu 11490, Taipei, Taiwan, R.O.C

人體試驗/研究計畫同意函

本審議會案號：C202101005
計畫編號：V-205
計畫名稱：一個評估 UB-612 疫苗對於新型冠狀病毒於青少年、成年和老年健康受試者的免疫原性、安全性與耐受性的第二期、安慰劑控制、隨機分派、觀察者盲性臨床試驗
執行機構：三軍總醫院
計畫主持人：感染及熱帶醫學科張峰義醫師
共同主持人：陳相成醫師
協同主持人：葉富強醫師；方文輝醫師；陳韋良醫師；劉偉修醫師；張芳維醫師
通過類型：變更案
通過日期：2021 年 10 月 19 日
同意核准執行期間：2021/9/5～2022/3/4
持續審查報告繳交頻率：半年一次(中度風險)
※本案須經衛生福利部核准同意後，始得進行試驗。
※本案須重新簽署受試者同意書。
※下次持續審查報告繳交截止日期：2022/3/4，應於到期日至少 6 週前提出持續審查申請，本案需經持續審查，方可繼續執行，若於到期日前完成試驗/研究，請繳交結案報告。
計畫主持人須依國內相關法令及本院規定通報嚴重不良反應事件及非預期問題。
本審議會組織與運作皆遵守 GCP 規定

Letter of Approval

TSGHIRB No.：C202101005
Protocol No.：V-205
Protocol title：A Phase II, Placebo-controlled, Randomized, Observer-blind Study to Evaluate the Immunogenicity, Safety, and Tolerability of UB-612 Vaccine against COVID-19 in Adolescent, Younger and Elderly Adult Volunteers
Research institution：Tri-Service General Hospital
Principle investigator：Dr. Feng-Yee Chang
Co-investigator：Dr. Hsiang-Cheng Chen
Sub investigator：Dr. Fu-Chiang Yeh；Wen-Hui Fang；Wei-Liang Chen；Wei-Hsiu Liu；Fung-Wei Chang
Type of Approval： Amendment
Date of Approval：2021/10/19
Duration of Approval：2021/9/5～2022/3/4
Frequency of Continuing Report：follow-up review 6 months (medium risk)
※The project may not be implemented until the approval is granted by the Ministry of Health and Welfare.
※Re-consent process is required.
※Next Deadline of Continuing Report：2022/3/4. If the study is completed prior to the approved expiration date, provide the Final Report.
The investigator is required to report any Serious Adverse Events and Unanticipated Problems in accordance with the governmental laws and regulations requirements
The organization and operation of the IRB is in accordance with Good Clinical Practice (GCP) and the applicable laws and regulations.

三軍總醫院 TSGHIRB 人體試驗審議會

Institutional Review Board
余慕賢 YuMuHsien
Chairman________________________________.

Tel: 886-2-87923311 ext 10552
Fax:886-2-2792-9201
E-mail: tsghirb@ndmctsgh.edu.tw

國防醫學院三軍總醫院
人體試驗審議會

台北市 11490 內湖區成功路二 325 號醫療大樓五樓 5113 室
No.325,Sec.2, Cheng-Kung Rd.
Neihu 11490, Taipei, Taiwan, R.O.C

計畫文件版本日期 Version/Date of documents

變更項目：

1. 計畫書 Protocol：Version 3.0_06 September 2021
2. 中文摘要 Chinese Synopsis：Version 3.0_06 September 2021
3. 英文摘要 English Synopsis：Version 3.0_06 September 2021
4. 受試者同意書 Informed Consent Form：
   (1) 青少年安全確認組 Safety Check Group -Adolescent：v2.0/2021-09-17
   (2) 成年安全確認組 Safety Check Group -Adult：v2.0/2021-09-17
   (3) 青少年安全確認第三劑組 Safety Check Group 3rd dose -Adolescent：v1.0/2021-09-17
   (4) 成年安全確認第三劑組 Safety Check Group 3rd dose -Adult：v1.0/2021-09-17
5. 個案報告表 CRF：Version 4.0_06 September 2021

三軍總醫院 TSGHIRB 人體試驗審議會

臺 北 榮 民 總 醫 院
TAIPEI VETERANS GENERAL HOSPITAL
201 SHIH-PAI ROAD. SEC.2
TAIPEI, TAIWAN 11217
REPUBLIC OF CHINA
TEL:(886)-2-2871-2121

同 意 臨 床 試 驗 / 研 究 證 明 書

IRB 編號：2021-04-003AU#4

計畫編號：V-205

計畫名稱：一個評估 UB-612 疫苗對於新型冠狀病毒於青少年、成年和老年健康受試者的免疫原性、安全性與耐受性的第二期、安慰劑控制、隨機分派、觀察者盲性臨床試驗

部門/計畫主持人：感染科/ 王復德醫師

協同主持人：林邑璁醫師、黃信彰醫師、洪妙秋醫師、陳曾基醫師、張曉婷醫師、劉瑞瑤醫師、陳育群醫師、鄭博仁醫師、陳夙容醫師、林宜璁醫師、陳育民醫師、陳威志醫師、蘇剛正醫師、潘聖衛醫師、陽光耀醫師、余文光醫師、柯信國醫師、黃惠君醫師、黃鈴茹醫師、楊盈盈醫師

變更之計畫文件版本日期：

1. 受試者保護指引 Subject Protection Guidance: Version 1.2, Date: 20210902
2. 計畫書 Protocol：Version 3.0, Date: 20210906
3. 中文摘要 Chinese Synopsis：Version 3.0, Date: 20210906
4. 英文摘要 English Synopsis：Version 3.0, Date: 20210906
5. 受試者同意書 Informed Consent Form：
   - 受試者同意書 (成年安全確認組)：Version 2.0, Date: 20211008
   - 受試者同意書 (青少年安全確認組)：Version 2.0, Date: 20211008
6. 第三劑組受試者同意書 Informed Consent Form for 3rd dose：
   - 受試者同意書 (成年安全確認第三劑組)：Version 1.0, Date: 20211008
   - 受試者同意書 (青少年安全確認第三劑組)：Version 1.0, Date: 20211008
7. 個案報告表 Case Report Form：Version 4.0, Date: 20210906

依據本委員會標準作業程序、及政府相關法律規章，本計畫修正/變更案案經本院人體試驗委員會(一)110 年 11 月 01 日第 143 次會議，於 110 年 11 月 01 日審查通過，有效期限至 111 年 03 月 14 日止，特此證明。本委員會的運作符合藥品優良臨床試驗準則及政府相關法律規章。計畫主持人須依國內相關法令及本院規定通報嚴重不良反應事件及非預期問題。計畫主持人須於到期前 3 個月至 6 週（至少前 6 週）提出持續審查之申請，本案須經本院人體試驗委員會通過後，方可繼續執行。（凡需送衛生福利部審核之計畫案件，須取得衛生福利部審核同意函後方可開始執行）

臺北榮民總醫院人體試驗委員會 Institutional Review Board Taipei Veterans General Hospital

臺 北 榮 民 總 醫 院
人 體 試 驗 委 員 會
主 任 委 員
馬　　旭

中 華 民 國 1 1 0 年 1 1 月 0 5 日

臺 北 榮 民 總 醫 院
TAIPEI VETERANS GENERAL HOSPITAL
201 SHIH-PAI ROAD. SEC.2
TAIPEI, TAIWAN 11217
REPUBLIC OF CHINA
TEL:(886)-2-2871-2121

Clinical Trial/Research Approval Letter

Nov 05, 2021

IRB-TPEVGH No.: 2021-04-003AU#4
Protocol No:V-205
Protocol Title: A Phase II, Placebo-controlled, Randomized, Observer-blind Study to Evaluate the Immunogenicity, Safety and Tolerability of UB-612 Vaccine against COVID-19 in Adolescent, Younger and Elderly Adult Volunteers
Department/Principal Investigator: Division of Infectious Disease/ Fu-Der Wang, M.D.
Sub-Investigator: Yi-Tsung Lin, M.D., Shinn-Jang Hwang, M.D., Miao-Chiu Hung, M.D., Tzeng-Ji Chen, M.D., Hsiao-Ting Chang, M.D., Jui-Yao Liu, M.D., Yu-Chun Chen, M.D., Bo-ren Cheng, M.D., Su-Jung Chen, M.D., Yi-Tsong Lin, M.D., Yuh-Min Chen, M.D., Wei-Chih Chen, M.D., Kang-Cheng Su, M.D., Sheng-Wei Pan, M.D., Kuang-Yao Yang, M.D., Wen-Kuang Yu, M.D., Hsin-Kuo Ko, M.D., Hui-Chun Huang, M.D., Ling-Ju Huang, M.D., Ying-Ying Yang, M.D.
Version date of Amendment documents:

1. Subject Protection Guidance: Version 1.2, Date: 20210902
2. Protocol：Version 3.0, Date: 20210906
3. Chinese Synopsis：Version 3.0, Date: 20210906
4. English Synopsis：Version 3.0, Date: 20210906
5. Informed Consent Form：
   - Informed Consent Form (Safety check group -Adult)：Version 2.0, Date: 20211008
   - Informed Consent Form (Safety check group -Adolescent)：Version 2.0, Date: 20211008
6. Informed Consent Form for 3rd dose：
   - Informed Consent Form (Safety check group － Adult 3rd dose)：Version 1.0, Date: 20211008
   - Informed Consent Form (Safety check group － Adolescent 3rd dose)：Version 1.0, Date: 20211008
7. Case Report Form：Version 4.0, Date: 20210906

According to the written operating procedures, GCP, and the applicable regulatory requirements, this Amendment study project is reviewed by the143th meeting of the Institutional Review Board (1) of Taipei Veterans General Hospital on Nov 01, 2021, and approved on Nov 01, 2021. This approval is valid till Mar 14, 2022. The board is organized under, and operates according to International Conference on Harmonisation (ICH) / WHO Good Clinical Practice (GCP) and the applicable laws and regulations. The principal investigator is required to report Serious Adverse Events and Unanticipated Problems in accordance with the governmental laws and regulations and TPEVGH requirements.The principal investigator is required to submit the application for extension before the expiration date of 6 weeks to 3 months (at least 6 weeks). (If indicated by the regulations and laws, this project should be taken after the approval of Ministry of Health and Welfare, R.O.C.)

臺北榮民總醫院 人體試驗委員會 Institutional Review Board Taipei Veterans General Hospital

Hsu Ma, M.D.
Chairman
Institutional Review Board
Taipei Veterans General Hospital
Taiwan, R.O.C.

高雄榮民總醫院人體研究倫理審查委員會
KAOHSIUNG V.G.H. Institutional Review Board
TEL：07-3422121-71571
FAX：07-3468344
e-mail：irb@vghks.gov.tw

人體試驗計畫同意函(1/2)

計畫名稱：一個評估UB-612疫苗對於新型冠狀病毒於青少年、成年和老年健康受試者的免疫原性、安全性與耐受性的第二期、安慰劑控制、隨機分派、觀察者盲性臨床試驗

計畫編號：KSVGH21-CT3-05

計畫主持人：蔡宏津醫師(hctsai1011@yahoo.com.tw；0975-581737)

通過會期：第213次會議

通過日期：2021年10月6日

修正項目：

1. 計畫書：Version 3.0, Date: 20210906
2. 中文摘要：Version 3.0, Date: 20210906
3. 英文摘要：Version 3.0, Date: 20210906
4. 受試者同意書：
   (1) 受試者同意書 (成年安全確認組)：Version 2.0, Date: 20210915
   (2) 受試者同意書 (青少年安全確認組)：Version 2.0, Date: 20210915
5. 新增第三劑組受試者同意書：
   (1) 受試者同意書 (成年安全確認第三劑組)：Version 3rd dose 1.0, Date: 20210915
   (2) 受試者同意書 (青少年安全確認第三劑組)：Version 3rd dose 1.0, Date: 20210915
6. 個案報告表：Version 4.0, Date: 20210906

有效期限：2022年2月23日

試驗機構：高雄榮民總醫院

主任委員 陳金順

陳金順

2021年10月6日

＊計畫主持人須遵守之規定請見「計畫主持人之職責」。

本會組織與執行皆符合ICH-GCP
IRB-KSVGH performs its functions according to written operating procedures and complies with GCP and with the applicable regulatory requirements

高雄榮民總醫院人體研究倫理審查委員會
KAOHSIUNG V.G.H. Institutional Review Board
TEL：07-3422121-71571
FAX：07-3468344
e-mail：irb@vghks.gov.tw

Certificate of Approval(2/2)

Protocol Title：A Phase II, Placebo-controlled, Randomized, Observer-blind Study to Evaluate the Immunogenicity, Safety, and Tolerability of UB-612 Vaccine against COVID-19 in Adolescent, Younger and Elderly Adult Volunteers

IRB No.：KSVGH21-CT3-05

Principal Investigator：Dr. Hung-Chin Tsai
(hctsai1011@yahoo.com.tw；0975-581737)

Board Meeting：213th

Approval Date：Oct. 6, 2021

Reason for Amendment：

1. **Protocol：Version 3.0, Date: 20210906**
2. **Chinese Synopsis：Version 3.0, Date: 20210906**
3. **English Synopsis：Version 3.0, Date: 20210906**
4. **Informed Consent Form：**
   (1) Safety check group - Adult：Version 2.0, Date: 20210915
   (2) Safety check group - Adolescent：Version 2.0, Date: 20210915
5. **Informed Consent Form for 3rd dose：**
   (1) 3rd Dose Safety check group - Adult：Version 3rd dose 1.0, Date: 20210915
   (2) 3rd Dose Safety check group - Adolescent：Version 3rd dose 1.0, Date: 20210915
6. **Case Report Form：Version 4.0, Date: 20210906**

Study Approval Expires：Feb. 23, 2022

Site: Kaohsiung Veterans General Hospital

Jin-Shuen, Chen, M.D
Chairman

Jin-Shuen Chen

Oct. 6, 2021

***Please review and follow the responsibility of the Principal Investigator.**

本會組織與執行皆符合ICH-GCP
IRB-KSVGH performs its functions according to written operating procedures and complies with GCP and with the applicable regulatory requirements

彰化基督教醫院
CHANGHUA CHRISTIAN HOSPITAL

變更案同意臨床試驗證明書
Clinical Trials Approval Certificate(Amendments)

135 Nanxiao St., Changhua City, Changhua County 500, Taiwan (R.O.C.)
Tel :886-4-723-8595 ext.8442
E-mail:d9065@cch.org.tw
彰化基督教醫院 Changhua Christian Hospital

500 彰化市南校街 135 號
聯絡人：洪翠霞
Contact Person : Tsui-Hsia Hung
電話：(04)723-8595 轉 8442
E-mail: d9065@cch.org.tw

計畫中文名稱： 一個評估 UB-612 疫苗對於新型冠狀病毒於青少年、成年和老年健康受試者的免疫原性、安全性與耐受性的第二期、安慰劑控制、隨機分派、觀察者盲性臨床試驗

計畫主持人： 劉尊榮 / 協同主持人：陳昶華、陳婉真、許瑛救、李育霖、楊順成

試驗機構名稱： 彰化基督教醫療財團法人彰化基督教醫院

研究經費來源： 聯亞生技開發股份有限公司

計畫編號：V-205 / 本會編號：210304

核准日(審查通過日)： 西元 2021 年 10 月 02 日

核准臨床試驗期間： 西元 2021 年 10 月 02 日 至 西元 2022 年 03 月 21 日止

計畫書： v3.0, 06 September, 2021

受試者同意書：
1. 受試者同意書(成年安全確認組) : Version 2.0 Date:20210917
2. 受試者同意書(青少年安全確認組) : Version 2.0 Date:20210917
3. 受試者同意書(成年安全確認第三劑組) : Version 3rd dose 1.0, Date:20210917
4. 受試者同意書(青少年安全確認第三劑組) : Version 3rd dose 1.0, Date:20210917)

個案報告表： v4.0, 06 September, 2021

協同主持人退出試驗：陳賢孟

未預期事件或藥品嚴重不良反應通報、後續定期追蹤之程序及應注意事項，請參閱背面。

彰化基督教醫院 人體試驗委員會 Institutional Review Board Changhua Christian Hospital

彰化基督教醫院
CHANGHUA CHRISTIAN HOSPITAL

Protocol Title: A Phase II, Placebo-controlled, Randomized, Observer-blind Study to Evaluate the Immunogenicity, Safety and Tolerability of UB-612 Vaccine against COVID-19 in Adolescent, Younger and Elderly Adult Volunteers
Principal Investigator(s): Chun Eng Liu / Co Investigator：chen changhua、Wan-Chin CHEN、Ing-Moi HII、Yu-Lin Lee、Yang, Shun-Cheng
Institution: CHANGHUA CHRISTIAN HOSPITAL
Sponsor: 聯亞生技開發股份有限公司
Protocol No. : V-205 / CCH IRB No. : 210304
Date of Approval: Oct 02, 2021
Duration of Approval: from Oct 02, 2021 to Mar 21, 2022

Protocol: v3.0, 06 September, 2021
Informed Consent Form:
1. 受試者同意書(成年安全確認組) : Version 2.0 Date:20210917
2. 受試者同意書(青少年安全確認組) : Version 2.0 Date:20210917
3. 受試者同意書(成年安全確認第三劑組) : Version 3rd dose 1.0, Date:20210917
4. 受試者同意書(青少年安全確認第三劑組) : Version 3rd dose 1.0, Date:20210917)

Case Report Form: v4.0, 06 September, 2021
Co-Investigator withdraw: Hsien-Meng Chen

See the back of this page for the procedures for reporting unanticipated problems, or drug serious adverse reactions, or interim, and other important notes.

彰化基督教醫院
第一人體試驗委員會
主任委員： 蘇矢立

Sincerely Yours
ShihLi Su, Ph.D.
Chairman
Institutional Review Board Committee A
Changhua Christian Hospital, Taiwan

彰化基督教醫院 人體試驗委員會 Institutional Review Board Changhua Christian Hospital

Shih-Li Su 2021.Oct.02
(signature, date)

本會組織與執行皆符合 ICH-GCP
The Institutional Review Board performs its functions according to written Operating procedures and complies with ICH-GCP and with the applicable regulations.

臺中榮民總醫院第一/二人體研究倫理審查委員會
Institutional Review Board I&II of Taichung Veterans General Hospital
407219 臺 中市西屯區臺灣大道四段1650號
1650 Taiwan Boulevard Sect. 4, Taichung, Taiwan 407219 , ROC
TEL：886-4-23592525#4006 FAX: 886-4-23592525#4408
E-mail: irbtc@vghtc.gov.tw

人體研究/試驗計畫修正案許可書

開立日期：西元 2021 年 10 月 12 日

計畫名稱：一個評估 UB-612 疫苗對於新型冠狀病毒於青少年、成年和老年健康受試者的免疫原性、安全性與耐受性的第二期、安慰劑控制、隨機分派、觀察者盲性臨床試驗

試驗編號：V-205

IRB 編號：SC21074A#3

計畫主持人：感染管制中心施智源醫師

協同主持人：家庭醫學部許碧珊醫師、內科部胸腔內科楊宗穎醫師、家庭醫學部社區醫學科林鉅勝醫師、內科部感染科劉伯瑜醫師、護理部黃惠美護理長、內科部感染科林詩萍醫師、內科部呼吸治療科詹明澄醫師、內科部呼吸治療科徐國軒醫師、內科部胸腔內科黃彥翔醫師、內科部內分泌新陳代謝科王俊興醫師、兒童醫學中心兒童血液腫瘤科黃芳亮醫師

研究/試驗執行機構：臺中榮民總醫院

修正內容及版本：

1.計畫書版本及日期：Version 3.0, Date：06 Sep, 2021

2.中文摘要版本及日期：Version 3.0, Date：06 Sep, 2021

3.英文摘要版本及日期：Version 3.0, Date：06 Sep, 2021

4.受試者同意書版本及日期：

- 成年安全確認組：Version 3.0, Date：17 Sep, 2021
- 成年安全確認第三劑組：Version 1.0, Date：17 Sep, 2021
- 青少年安全確認組：Version 3.0, Date：17 Sep, 2021
- 青少年安全確認第三劑組：Version 1.0, Date：17 Sep, 2021

5.個案報告表版本及日期：Version 4.0, Date：06 Sep, 2021

通過會期：第一人體研究倫理審查委員會第 110-A-11 次會議

有效期限：2022 年 03 月 11 日

（此案追蹤審查頻率為半年一次，請主持人主動繳交追蹤審查報告。）

＊ 依照赫爾辛基宣言及 ICH-GCP 規定，臨床試驗每屆滿一年，人體研究倫理審查委員會必須定期重新審查臨床試驗後方可繼續進行。請於有效期限到期二個月前繳交追蹤審查報告以利本會進行審查。
＊ 受試者於試驗期間發生嚴重不良事件及疑似未預期之嚴重藥物不良反應，主持人應依衛生福利部法規於期限內通報主管機構及審查之人體研究倫理審查委員會。
＊ 計畫展延應於許可書期限截止前二個月提出申請。
＊ 結案報告應於許可書期限截止後三個月內繳交。
＊ 本會有暫停/終止本研究計畫及撤銷本執行許可書之權責。

臺中榮民總醫院第一人體研究倫理審查委員會

主任委員 林志堅

林志堅

臺中榮民總醫院
第一人體研究倫理審查委員會
臺中榮民總醫院

本會組織與執行皆符合國際醫藥法規協合會之藥品優良臨床試驗規範
TCVGH-IRB is organized and operates according to ICH-GCP and the applicable laws and regulations.

臺中榮民總醫院第一/二人體研究倫理審查委員會
Institutional Review Board I&II of Taichung Veterans General Hospital
407219 臺 中市西屯區臺灣大道四段1650號
1650 Taiwan Boulevard Sect. 4, Taichung, Taiwan 407219 , ROC
TEL：886-4-23592525#4006 FAX: 886-4-23592525#4408
E-mail: irbtc@vghtc.gov.tw

Certificate of Protocol Amendment

Date of Approval:12 Oct,2021

Protocol Title： A Phase II, Placebo-controlled, Randomized, Observer-blind Study to Evaluate the Immunogenicity, Safety, and Tolerability of UB-612 Vaccine against COVID-19 in Adolescent, Younger and Elderly Adult Volunteers

Protocol No.： V-205

TCVGH-IRB No.： SC21074A#3

Principal Investigator： Zhi-Yuan Shi

Sub-Investigator： Pi- Shan Hsu, Tsung-Ying Yang, Chu-Sheng Lin, Po-Yu Liu, Hui-Mei Huang, Shih-Ping Lin, Ming-Cheng Chan, Kuo-Hsuan Hsu, Yen-Hsiang Huang, Jun-Sing Wang, Fang-Liang Huang

Institute： Taichung Veterans General Hospital

Reason for Amendment & Version：

1.Protocol Version & Date： Version 3.0, Date：06 Sep, 2021

2.Protocol Synopsis Chinese Version & Date： Version 3.0, Date：06 Sep, 2021

3.Protocol Synopsis English Version & Date： Version 3.0, Date：06 Sep, 2021

4.Informed Consent Form Version & Date：

- Safety check group - Adult：Version 3.0, Date：17 Sep, 2021
- 3rd dose Safety check group - Adult：Version 1.0, Date：17 Sep, 2021
- Safety check group - Adolescent：Version 3.0, Date：17 Sep, 2021
- 3rd dose Safety check group - Adolescent：Version 1.0, Date：17 Sep, 2021

5.Case Report Form Version & Date： Version 4.0, Date：06 Sep, 2021

Board Meeting： Institutional Review Board (I) 110-A-11 Board Meeting

Approval Effective Period： 11 March 2022

Frequency of Continuing Review： 6 months

＊ In accordance with Declaration of Helsinki and ICH-GCP guidelines, PI is responsible for submitting a progress report to IRB two months prior to the expiry date for an annual review.
＊ Serious adverse events or SUSAR involving risk to participants must be reported to Ministry of Health and Welfare (MOHW) and IRB according to current regulation.
＊ Application for protocol extension should be submitted to IRB two months before the expiry date of the Certificate of Approval.
＊ Closing report should be submitted to IRB within three months after the expiry date of the Certificate of Approval.
＊ The IRB has authorization to suspend/terminate the protocol and to withdraw the Certificate of Approval.

Chih-Chien Lin, MD, MPH
Chair, Institutional Review Board (I), TCVGH

臺中榮民總醫院 第一人體研究倫理審查委員會 臺中榮民總醫院

Chih-Chien Lin
