# Supplementary material for: Toward a pan-SARS-CoV-2 vaccine targeting conserved epitopes on spike and non-spike proteins for potent, broad and durable immune responses
Source: PLoS Pathog. 2023 Apr 20;19(4):e1010870. doi: 10.1371/journal.ppat.1010870 (PMC10153712; doi:10.1371/journal.ppat.1010870)
Supplement: S3 Appendix — (PDF) [file ppat.1010870.s016.pdf]

S3 Supplemental Appendix

Phase-2 study V-205 Informed Consent Form (ICF)

中國醫藥大學暨附設醫院
受試者同意書
（成年免疫組）

您被邀請參與此研究。此同意書主要是提供您本研究之相關資訊，以便您決定是否參加本研究。計畫主持人或其指定之研究人員會為您說明研究內容並回答您的疑問。您可以提出任何和此研究有關的問題，在您的問題尚未獲得滿意的答覆之前，請不要簽署此同意書。如果您願意參與本研究，此文件將視為您的同意紀錄。即使在您同意後，您可以隨時退出本研究不需任何理由。

<table>
<tr><td colspan="2">計畫名稱
中文：一個評估 UB-612 疫苗對於新型冠狀病毒於青少年、成年和老年健康受試者的免疫原性、安全性與耐受性的第二期、安慰劑控制、隨機分派、觀察者盲性臨床試驗
英文：A Phase II, Placebo-controlled, Randomized, Observer-blind Study to Evaluate the Immunogenicity, Safety, and Tolerability of UB-612 Vaccine against COVID-19 in Adolescent, Younger and Elderly Adult Volunteers</td></tr>
<tr><td>執行單位：中國醫藥大學附設醫院感染科、家庭醫學科</td><td>委託單位/藥廠：聯亞生技開發股份有限公司
研究經費來源：聯亞生技開發股份有限公司
受託研究機構：晉加股份有限公司</td></tr>
<tr><td>計畫主持人：黃高彬</td><td>職稱：主治醫師</td></tr>
<tr><td>協同主持人：林文元</td><td>職稱：主治醫師</td></tr>
<tr><td>協同主持人：林伯昌</td><td>職稱：主治醫師</td></tr>
<tr><td>緊急聯絡人：黃高彬</td><td>電話：0975-681-950</td></tr>
<tr><td>受試者姓名：
性別：
身分證字號：
通訊地址：</td><td>病歷號碼：
出生日期：
聯絡電話：</td></tr>
<tr><td>法定代理人或有同意權人之姓名：
性別：
身分證字號：
通訊地址：</td><td>與受試者關係：
出生日期：
聯絡電話：</td></tr>
<tr><td colspan="2">(一)試驗簡介：
1. 本品/技術資料：</td></tr>
</table>

中國醫藥大學暨附設醫院
受試者同意書
（成年免疫組）

新型冠狀病毒(SARS-CoV-2)於2019年12月起造成中國湖北省武漢市發現多起病毒性肺炎群聚，隨後於2020年1月底台灣出現第一起境外移入確診個案。此疾病在全球擴散，世界衛生組織宣布將此疫情為「國際關注公共衛生緊急事件」。截至2020年底，全球僅有數間疫苗公司，如美國輝瑞藥廠等，取得緊急使用授權上市。

UB-612疫苗為聯亞生技開發股份有限公司所開發新型冠狀病毒預防性疫苗，疫苗含病毒棘狀融合蛋白和胜肽片段，可產生高親和力抗體與新型冠狀病毒結合，並誘發細胞免疫反應，進而達到預防新型冠狀病毒的感染。

UB-612第一期延伸性試驗顯示，接種第三劑UB-612疫苗可以誘發極高的中和抗體，在目前變種病毒的威脅之下，施打第三劑加強免疫反應，已是許多國家的選擇。

2. 本品上市狀況：

本品仍應用於人體試驗，尚未在我國上市。

3. 本試驗使用的UB-612疫苗對新型冠狀病毒的預防效果仍未確認。

(二)試驗目的：

主要試驗目的

- 評估UB-612疫苗誘發的新型冠狀病毒中和抗體效價。
- 評估接種UB-612疫苗後的安全性和耐受性。

次要試驗目的

- 評估在試驗期間對於新型冠狀病毒的免疫反應。
- 評估三批獨立批次疫苗的批次免疫一致性。

探索性試驗目的

- 評估UB-612疫苗誘發的T細胞功能。
- 評估UB-612疫苗在年輕受試者的安全性和免疫原性。
- 評估UB-612疫苗的療效。
- 描述UB-612疫苗於確診和/或嚴重感染新型冠狀病毒案例之血液學反應。
- **評估針對SARS-CoV-2抗原的抗體反應。**

（三)試驗之主要納入與排除條件：

中國醫藥大學暨附設醫院執行本研究計畫的醫師或相關研究人員將會與您討論有關參加本研究的必要條件。請您配合必須誠實告知我們您過去的健康情形，若您有不符合參加本研究的情況，將不能參加本研究計畫。

1. 參加本研究計畫的主要條件:

☐(1) 您為納入試驗時20~85歲之間健康男性或未懷孕的女性受試者。

☐(2) 您為具生育能力的女性與男性應於首次接種疫苗至最後一次疫苗後3個月同意進行有效的避孕方式。可接受的有效避孕方式包括:

☐a. 男性或女性以手術方法絕育、植入式避孕、或子宮避孕器。

☐b. 注射避孕、避孕藥、避孕貼片、避孕環加上一種屏障避孕法*。

☐c. 合併使用兩種屏障避孕法*。

*有效的屏障避孕法為避孕隔膜、男性或女性保險套、避孕海綿或殺精劑(含可殺精化學物質的藥膏或凝膠)。

☐(3) 您能理解受試者同意書內容的說明與可能的風險，提供簽名的受試者同意書。

☐(4) 您能夠理解與遵從本試驗程序與能夠參與每次訪視。

☐(5) 您的耳溫≤ 38.0°C。

☐(6) 您依據醫療病史、身體檢查和試驗主持人的臨床判斷為健康受試者**可符合納入試驗資格。經試驗主持人判斷，即便您的病史穩定或且控制良好，但伴隨病情惡化而有提高嚴重新型冠狀病毒感染的風險。

**健康受試者有先前存在的穩定疾病者可以納入試驗，定義為該疾病在納入試驗前12週內沒有惡化至需要治療或住院的顯著變化和在納入試驗6個月內沒有惡化至需要治療或住院的顯著變化。

2. 若您有下列任一情況，您將無法參加本研究計畫:

☐(1) 您有接種疫苗後需要醫療介入的過敏性休克、蕁麻疹或其他顯著不良反應的病史。

☐(2) 您在篩選時或接種每劑疫苗前已懷孕女性或懷孕檢測為陽性的女性。

☐(3) 您為正在哺乳的女性，或計畫從接種第一劑疫苗至最後一劑疫苗後60天哺乳的女性。

☐(4) 您在接種第一劑疫苗前3天內，經試驗主持人判斷，患有任何急性疾病。

☐(5) 您在接種第一劑疫苗前1個月內有重大手術。

☐(6) 您是已知為人類免疫缺乏病毒抗體陽性。

☐(7) 您是已知為活動性B型肝炎或C型肝炎。活動性肝炎定義為肝臟轉胺酶(天門冬胺酸轉胺酶和/或丙胺酸轉胺酶)大於3倍正常值上限和/或總膽紅素大於3倍正常值上限。

☐(8) 您是已知曾暴露於新型冠狀病毒，或曾接受預防新型冠狀病毒、中東呼吸症候群冠狀病毒、嚴重急性呼吸道症候群的試驗或已上市產品。

☐(9) 您有格林-巴利症候群的病史。

☐(10) 您在簽署受試者同意書前12周內參與其他的臨床試驗。

☐(11) 您為免疫缺乏/失調疾病，無論是否由基因缺陷、免疫缺乏症或免疫抑制療法所造成。

☐(12) 您計畫或正在進行抗癌症治療。

☐(13) 您患有血小板異常或其他凝血異常可能造成注射之禁忌症。

☐(14) 您在接種第一劑疫苗前6個月長期接受(≥14天連續使用)免疫抑制劑、皮質類固醇(相當於一天使用≥ 20 mg強的松(prednisone))或細胞毒性治療。

☐(15) 您在接種第一劑疫苗前4個月接受免疫球蛋白和/或任何血液製劑的治療。

☐(16) 您在接種試驗疫苗前14天接種任何季流感疫苗或新型流感疫苗，或前28天接種其他疫苗。

☐(17) 您預期在接種試驗疫苗後14天接種任何季流感疫苗或新型流感疫苗，或後28天接種其他疫苗。

☐(18) 您使用短期(<14天使用)全身性類固醇。應於中斷使用全身性類固醇至少28天後才可使用試驗疫苗。吸入/噴霧性、關節注射、囊內或局部(皮膚或眼用)類固醇可允許使用。

☐(19) 您在篩選期前3個月失血或捐血超過500毫升，或預計在試驗期間內捐血或輸血。

☐(20) 經試驗主持人判斷，您有任何醫療疾病或狀況，可能會影響試驗結果或參與試驗可能會對受試者引發額外風險。

☐(21) 您是直接參與本試驗執行的試驗主持人所屬機構**的試驗團隊**、試驗委託者或受託

研究機構(CRO)的員工。

（四）試驗方法及相關檢驗：

這是一個第二期、觀察者盲性、多中心、隨機分派、安慰劑控制試驗，以評估青少年，成年和老年受試者使用兩劑UB-612疫苗的免疫原性，耐受度和安全性。有一部份的受試者使用UB-612疫苗，而另外一部份的受試者則使用「安慰劑」。所謂「安慰劑」是不含有效成份的疫苗。至於誰使用試驗用藥或誰使用「安慰劑」，則像丟銅板或擲骰子一樣由機率決定，不管是您或是研究醫師都不知道您使用了那一種藥，只有分發跟施打疫苗的試驗人員才知道您使用哪一種疫苗，這叫做觀察者盲性。

總計約有3850位合格成年受試者組成核心組用於申請緊急使用授權，另外大約有385位青少年受試者組成補充組申請額外適應症。所有受試者將以6:1的比例，隨機分派至兩劑100微克劑量組別和安慰劑組，包括462位大於18歲至小於65歲可評估的受試者進入批次分析組。對於免疫分析，至少包括350位可評估的成年受試者(年齡大於18歲至小於65歲)和154位可評估的老年受試者(年齡≥65歲)進行描述性分析。免疫原性的受試者將會先納入試驗。所有的受試者將會納入安全性分析，其中至少770位隨機分派的受試者為≥65歲的分層。青少年組將在核心組招募完畢後，再開始納入試驗。約有385位青少年受試者將以6:1的比例隨機分派，其中包括154位可評估的青少年受試者將收集免疫原性數據，並和成年及老年受試者數據進行比較。

您若參與這個試驗，則為有進行免疫分析檢測的免疫原性或批次一致組。

試驗總共有8個訪視。若您參與本試驗，則至少包括第一次訪視(篩選訪視)、第二次訪視（第1天，基礎值，隨機分派，第一次接種疫苗)、第三次訪視(第29天，第二次接種疫苗)、第四次訪視(第57天)、第五次訪視(第197天)。

在第五次訪視時，預計將進行個別解盲。解盲後，得知您為施打疫苗的受試者，且您有意願且符合資格接種第三劑疫苗，將請您簽署另外一份受試者同意書，以進行後續程序；包含第六次訪視(第197~242天，第三次接種疫苗)，第七次訪視(接種疫苗後第14天)，及第八次訪視(第365天)。

若個別解盲後，得知您為施打疫苗的受試者，您不願意進行第三劑疫苗接種，將只進行第八次訪視(第365天)的安全性及抗體效價的追蹤。

若個別解盲後，得知您為施打安慰劑組的受試者，將結束您的試驗。

整個試驗期間，將預期您將參與試驗最長達13個月。

注意事項

中國醫藥大學暨附設醫院
受試者同意書
（成年免疫組）

1. 如果您同意參加本試驗，研究人員會請您簽署本份受試者同意書，並確認您符合參加本試驗的條件。
2. 從您參與試驗的當天開始，每次訪視都將有合格的試驗人員執行試驗流程與聯繫。
3. 若您有任何符合新型冠狀病毒感染的定義(您曾於過去 7 天內有出國，或是接觸疑似或確認武漢肺炎之病人，而有下列症狀: 發燒、開始咳嗽或惡化、開始呼吸急促或惡化、寒顫、開始肌肉疼痛或惡化、喉嚨痛、腹瀉、嘔吐、開始味覺/嗅覺異常)，**請依照中央疫情指揮中心規定進行自主健康管理或至指定院所進行篩檢。**
4. 若您在試驗期間感染新型冠狀病毒，將依法通報主管機關。

   您是否同意? ☐是 ☐否

   簽名：____________日期：______________
5. 於試驗期間，您不論任何理由提前退出試驗，試驗研究人員都將安排您完成最後一次的訪視之所有試驗項目。您有權利拒絕此項安排，您的決定不會引起任何影響日後醫師對您的醫療照護。

試驗步驟

第一次訪視(第-28~-1 天) -篩選訪視

在試驗醫師或試驗研究人員為您提供足夠的試驗資訊，並確保您有充分的時間考慮以及詢問任何問題後，您願意讓您參與本試驗，並由您簽署本受試者同意書。在確認您已完成受試者同意書簽署並且您也保有一份副本後，試驗醫師或試驗研究人員將會進行以下試驗程序：

(1) 記錄您簽署受試者同意書的日期
(2) 為您指定一組受試者篩選編號
(3) 確認您是否符合本試驗的納入排除條件
(4) 收集您的個人基本資料（例如生日、年齡及性別）
(5) 記錄您的醫療/用藥病史
(6) 進行身體檢查，包括身高體重
(7) 確認生命徵象
(8) 進行心電圖檢測
(9) 收集尿液檢體進行尿液檢測

(10) 收集血液檢體(共 **15.5** 毫升)，進行下列檢測:

- 常規血液檢測
- 血液生化學檢測
- 免疫學檢測**(抗核抗體)**
- **備血(作為測量新型冠狀病毒血清抗體和相關研究之用)**

第二次訪視(第 1 天)-基礎值，確認符合試驗條件，第一次接種疫苗

(1) 再度確認您是否符合本試驗的納入排除條件
(2) 隨機分派，給予您一組隨機分派號碼
(3) 記錄您的醫療/用藥病史
(4) 進行身體檢查
(5) 確認生命徵象
(6) 進行尿液懷孕檢測（具有生育能力女性）
(7) 若您有產生第三級以上的高血壓，您將被收集尿液檢體以檢測是否有蛋白尿存在或惡化
(8) 收集血液檢體(若不進行 T 細胞檢測，共 20 毫升)，進行下列檢測:

- 免疫**原性**檢測，包括 Anti-S1-RBD 免疫球蛋白 G 濃度，和新型冠狀病毒中和抗體效價
- 若您願意，將進行 T 細胞檢測(需額外抽血 56 毫升)

您是否同意? □是 □否

簽名：__________日期：____________

(9) 進行第一次疫苗接種(注射部位為非慣用手，採用肌肉注射方式)。接種疫苗後，受試者應留在試驗地點至少 30 分鐘，監測生命徵象和急性過敏症狀。
(10) 將詳細地指導您如何填寫電子日誌卡(包括注射後 7 天內預期性不良事件，14 天內的皮膚過敏反應日誌卡)
(11) 收集併用藥物/治療

第一次電話安全性追蹤(第 8, 15, 22 天)

將與您電話聯繫，以追蹤未預期不良事件和新型冠狀病毒感染症狀。

第三次訪視(第 29± 3 天)-第二次接種疫苗

(1) 進行第二次接種評估(有可能會延遲接種時間)
(2) 進行身體檢查

(3) 確認生命徵象

(4) 進行尿液懷孕檢測（具生育能力的女性）

(5) 若您有產生第三級以上的高血壓，您將被收集尿液檢體以檢測是否有蛋白尿存在或惡化。

(6) 若您為批次一致/免疫原組，將收集血液檢體(共 5 毫升)，進行下列檢測:

- 特定抗原 Anti-S1-RBD 抗體

(7) 進行第二次疫苗接種。

(8) 接種疫苗後，受試者應留在試驗地點至少 30 分鐘，監測生命徵象和急性過敏症狀。

(9) 將詳細地指導您如何填寫電子日誌卡(包括注射後 7 天內預期性不良事件，14 天內的皮膚過敏反應日誌卡)

(10) 收集併用藥物/治療

(11) 記錄上一次訪視至此次訪視之間的不良事件、嚴重不良事件型或新型冠狀病毒感染症狀

<u>第二次電話安全性追蹤(第 36, 43 天)</u>

將與您電話聯繫，以追蹤未預期不良事件和新型冠狀病毒感染症狀。

<u>第四次訪視(第 57±3 天) –追蹤訪視</u>

(1) 進行身體檢查

(2) 確認生命徵象

(3) 若您有產生第三級以上的高血壓，您將被收集尿液檢體以檢測是否有蛋白尿存在或惡化。

(4) 收集血液檢體(若不進行 T 細胞檢測，共 35 毫升) ，進行下列檢測:

- 免疫**原性**檢測，包括 Anti-S1-RBD 免疫球蛋白 G 濃度、和新型冠狀病毒中和抗體效價
- 若您願意，將進行 T 細胞檢測(需額外抽血 56 毫升)
- 常規血液檢測
- 血液生化學檢測
- 免疫學檢測**(抗核抗體)**
- **備血(作為測量新型冠狀病毒血清抗體和相關研究之用)**

(5) 收集併用藥物/治療

(6) 記錄上一次訪視至此次訪視之間的不良事件、嚴重不良事件或新型冠狀病毒感染症狀

(7) 在此次訪視後，您將每周接獲訊息提醒，以定期監測新型冠狀病毒感染症狀

第三次電話安全性追蹤(第 64, 71, 78, 85 天)

將與您電話聯繫，以追蹤安全性及新型冠狀病毒感染症狀。

第五次訪視(第 197±15 天) –個別解盲

(1) 進行身體檢查

(2) 確認生命徵象

(3) 若您有產生第三級以上的高血壓，您將被收集尿液檢體以檢測是否有蛋白尿存在或惡化。

(4) 將替您個別解盲，告知您接種到疫苗或安慰劑。若您為安慰劑組，您已完成本試驗，將協助您退出試驗。但若您為疫苗組且符合資格，我們將提供另外一份受試者同意書，邀請您參與後續的第三劑接種試驗。若您不同意接種第三劑，您仍需進行後續的安全性及抗體效價的追蹤。

(5) 收集血液檢體(共 20 毫升) ，進行下列檢測:

- 免疫**原性**檢測，包括 Anti-S1-RBD 免疫球蛋白 G 濃度、和新型冠狀病毒中和抗體效價

(6) 收集併用藥物/治療

(7) 記錄上一次訪視至此次訪視之間的不良事件、嚴重不良事件或新型冠狀病毒感染症狀

第四次電話安全性追蹤(第 253, 309 天)

第 197 天後將每兩個月與您電話聯繫，以追蹤安全性及新型冠狀病毒感染症狀。

最後一次訪視(第 365±45 天) –第 12 個月追蹤

(1) 確認生命徵象

(2) 收集血液檢體(共 20 毫升) ，進行下列檢測:

- 免疫**原性**檢測，包括 Anti-S1-RBD 免疫球蛋白 G 濃度、和新型冠狀病毒中和抗體效價

(3) 記錄上一次訪視至此次訪視之間的不良事件，包括特殊不良事件、醫療不良事件、嚴重不良事件或新型冠狀病毒感染症狀

受試者之檢體(含其衍生物)之保存、使用與再利用：

1. 檢體及剩餘檢體之保存與使用

(1) 檢體(含其衍生物)之保存與使用

為研究所需，我們所蒐集您的檢體，將依本研究計畫使用，檢體將保存於**聯亞生技開發股份有限公司(試驗委託者)**，直至20年保存期限屆滿，我們將依法銷毀。為了保護您的個人隱私，我們將以一個試驗編號來代替您的名字及相關個人資料，以確認您的檢體及與相關資料受到完整保密。如果您對檢體的使用有疑慮，或您有任何想要銷毀檢體的需求，請立即與我們聯絡(聯絡人：黃高彬醫師電話：0975-681-950），我們即會將您的檢體銷毀。您也可以聯繫中國醫藥大學暨附設醫院研究倫理委員會(電話：04-22052121 轉 1925、1926)，以協助您解決檢體在研究使用上的任何爭議。

(2) 剩餘檢體(含其衍生物)之再利用

您的生物檢體將會以專屬號碼進行編碼並在聯亞生技開發股份有限公司(試驗委託者)的控管下儲存最長20年，以研究UB-612 疫苗反應者的生物標記，及改善治療方式。

所有新的研究計畫都要再經由中國醫藥大學暨附設醫院研究倫理委員會審議通過，倫理審查委員會若認定新的研究超出您同意的範圍，將要求我們重新得到您的同意。

是否同意剩餘檢體保留提供未來新型冠狀病毒感染研究之用，並授權中國醫藥大學暨附設醫院研究倫理委員會審議是否需要再取得您的同意(擇一)

☐ 不同意保存我的剩餘檢體，試驗結束後請銷毀

☐ 同意以非去連結之方式保存我的剩餘檢體，逾越原同意使用範圍時，需再次得到我的同意才可使用我的檢體進行新的研究

2. 檢體及剩餘檢體之部分類型(檢體類型可依計畫書內容自行增減)

(1) 一般生化、血液檢驗/病毒檢測檢體

在試驗期間，會將您的檢體送往聯亞生技開發股份有限公司(試驗委託者)委託的中央實驗室中國醫藥大學暨附設醫院，此機構地址為台中市北區育德路2號，和大安聯合醫事檢驗所，此機構地址為台北市大安區復興南路二段151巷33號，中央實驗室會在分析後立即將分析結果提供給試驗中心，若有剩餘的檢體，**將儲存直到至少完成臨床試驗報告為止，最長將保存20年。**

(2) 抗體/細胞免疫試驗

在試驗期間，會將您的檢體送往聯亞生技開發股份有限公司(試驗委託者)分析實驗

室。完成試驗後，若有剩餘檢體，將儲存直到至少完成臨床試驗報告為止，最長將保存20年。

(3) 中和試驗(neutralization test, NT)

在試驗期間，會將您的檢體送往聯亞生技開發股份有限公司(試驗委託者)委託的中央實驗室中央研究院進行處置、處理與進一步分析。此機構地址為台北市南港區研究院路二段128號。完成試驗後，若有剩餘檢體，將儲存直到至少完成臨床試驗報告為止，最長將保存20年。

(4) 遺傳學檢體

在試驗期間，若發生嚴重不良反應或特定不良反應，您的檢體將用於HLA分型檢驗，會將您的檢體送往聯亞生技開發股份有限公司(試驗委託者)委託的中央實驗室有勁基因股份有限公司分析，此機構地址為新北市樹林區復興路376-5號，中央實驗室不會將分析結果提供給試驗中心，若有剩餘的檢體，將會儲存直到檢驗結果複驗完畢即銷毀，不會長期儲存。

(5) 探索性試驗檢體

在試驗期間，會將您的檢體送往聯亞生技開發股份有限公司(試驗委託者)委託的實驗室(表一)進行處理或進一步分析。完成試驗後，若有剩餘檢體，將儲存直到至少完成臨床試驗報告為止，最長將保存20年。

表一、實驗室名稱與機構地址

實驗室名稱	機構地址
聯亞生技開發(股)公司	新竹縣竹北市生醫路二段 6-1 號 5 樓
Viroclinics	Rotterdam Science Tower, Marconistraat 16, 3029 AK Rotterdam, The Netherlands(荷蘭)
DASA	Jonas Cruz de Araujo, Diagnostics da America S/A, Surubiju Avenue, 1890, Barueri, SP, Brazil(巴西), 06455-040
PHE Porton Down	Salisbury Wiltshire SP4 0JG, England(英國)
UTMB	University of Texas Medical Branch 301 University Boulevard Keiller Building, Room 2.150 Galveston, Texas, USA(美國)
Virology	University of São Paulo, Brazil Rua Dr EnnEn de Carvalho Aguiar 470, CEP 05403-000 (巴西)
VRDL	California Department of Public Health, 850 Marina Bay Parkway, Richmond, CA 94804, USA(美國)

中國醫藥大學暨附設醫院
受試者同意書
（成年免疫組）

**NEXELIS**	**525 Boul. Cartier Ouest Laval, Qulbec, Canada, H7V 3S8(加拿大)**
**Vaccinology and Immunology Infection, Immunity & Inflammation Dept UCL GOS Institute of Childe Health**	**UCL Great Ormond Street Institute of Child Health 30 Guilford Street London WC1N 1EH, England(英國)**
**VisMederi**	**VisMederi Srl, Strada del Petriccio e Belriguardo, 35, 53100 Siena, Italy(義大利)**

(五)可能產生之副作用、發生率及處理方法：

1. 與試驗藥物相關的風險（本試驗疫苗的副作用）：

冠狀病毒疫苗的開發

過去針對與SARS-CoV-2病毒相同屬於人類冠狀病毒的SARS-CoV(嚴重急性呼吸綜合症冠狀病毒(SARS冠狀病毒))的疫苗研究發現，接種過SARS-CoV疫苗的小鼠在暴露到SARS-CoV後會發生過度免疫反應而產生病變，因此不得不停止這種疫苗的開發。所以，成功的人類冠狀病毒疫苗不只要產生可以抑制病毒的免疫反應，更要避免過度免疫產生的副作用。

疫苗相關的風險: 第一期臨床試驗

接種疫苗可能會出現注射部位的不良反應(例如疼痛、硬化腫脹、皮疹發紅、過敏反應、蜂窩性組織炎)，或全身性不良反應(例如發燒、腹瀉、疲倦、噁心/嘔吐、厭食、咽喉痛、頭痛、咳嗽、關節痛、非注射部位疼痛、非注射部位搔癢、皮膚和黏膜異常、急性過敏反應、昏厥、急性支氣管痙攣、呼吸困難)。

第一期臨床試驗已經有60位受試者接種兩劑疫苗(含10微克、30微克、100微克融合蛋白)，安全實驗室數值並沒有顯示有任何的臨床顯著不正常數值，也沒有發生任何第三級以上與疫苗相關的預期性不良事件。大部分的預期性不良事件都是輕微的，大約於2天之內症狀都會緩解。也沒有任何的嚴重不良事件或特殊不良事件被通報。

疾病增強(disease enhancement) 的風險

SARS-CoV-2候選疫苗也可能會有引發疾病增強(disease enhancement) 的風險，包括抗體依賴性增強(antibody-dependent enhancement)或疫苗相關聯的增強的呼吸道疾病(vaccine-associated enhanced respiratory disease)。在先前研發SARS疫苗時，在數個SARS-CoV動物攻毒試驗(包括鼠類、雪貂、猴類)當中，有發現疾病增強的現象。疾病增強反應的免疫病理現象包括TH2偏向及嗜酸性白血球的肺部浸潤。但是目前已發表的新型冠狀病毒肺炎

疫苗研究，仍尚未發現類似的疾病增強現象。

本試驗疫苗於數個藥理試驗呈現不一致的TH1/TH2 (輔助型T細胞1/輔助型T細胞2)免疫反應偏向，試驗結果並未一致偏向TH2，而由小鼠之SARS-CoV-2動物攻毒試驗結果顯示，本試驗疫苗誘發疾病增強之風險不高。依據文獻指出，組成複雜或容易引起非中和抗體之抗原，如不活化病毒或整片段之蛋白(包含S蛋白與N蛋白)，與易引起偏向Th2免疫反應之佐劑成分，如鋁製佐劑，皆較有可能引起疾病增強。本試驗疫苗的主要抗原為S蛋白上之RBD 區域，已有多篇文獻指出，針對S-RBD設計之SARS與MERS疫苗從未於試驗動物模型上引發疾病增強現象。本試驗疫苗雖使用易引起偏向Th2免疫反應之佐劑，但由動物實驗證實，也同時引起偏向Th1之反應，因此發生疾病增強應屬低風險。且已於多種動物模型中證實，能誘發高效價之中和抗體，於細胞培養中亦能有效抑制新冠病毒感染。

建議您在有效疫苗上市前或本試驗疫苗的產品資訊有進一步更新前，盡量避免暴露於可能感染病毒的環境。研究團隊將會在試驗中執行相關安全性監測。若有任何關於本試驗疫苗與疾病增強風險相關之任何最新資訊，將即時更新並提供給您。

疫苗佐劑相關的風險

本試驗疫苗所使用的佐劑含Adju-Phos®，是屬於一種磷酸鋁類的佐劑。磷酸鋁類佐劑已經使用超過半個世紀，具有相當的安全性。由於此類佐劑可誘導免疫反應，因此可能會造成局部發炎反應，例如在注射部位產生輕微而短暫的疼痛、發紅以及腫脹。

2. 與試驗/研究過程相關的風險：

抽血

本試驗需要抽血檢驗。抽血可能引起一些不適和瘀血。整個試驗期間13個月，共需抽血**116 毫升**。若您願意抽血檢驗T細胞檢測，則會再額外抽血共112毫升。若您罹患新型冠狀病毒感染，**可能將於每次額外訪視抽血30毫升。**

在接種疫苗過程中，可能會出現一些尚未在已完成試驗中發現的副作用。一般而言，接種某一新疫苗總是會有一定的風險，但是計畫主持人會採取一切措施預防風險的發生。**計畫主持人鼓勵您報告您遇到的任何不適。**

(六)其他替代療法及說明：

您不是非參加不可，若不參加研究，由於目前尚未有疫苗可用來預防新型冠狀病毒感染，因此預防措施與其他呼吸道感染相同，包括：勤洗手、減少觸摸眼口鼻、注意咳嗽禮節、妥善處理口鼻分泌物等，避免出入公共場所，並不要接觸野生動物。

如果您對於本試驗疫苗有任何的疑問，您可以提出來向您的試驗醫師討論。

（七）試驗預期效益：

依據臨床前試驗結果，預期本試驗疫苗對您可能可以產生抗體，預防新型冠狀病毒感染，但因每個人體質不同也有可能不會產生療效，故參加本試驗可能不會有直接的好處。

但是您參加本試驗，可協助我們獲得更多資訊，以瞭解UB-612疫苗的安全性與免疫力。

（八）試驗進行中受試者之禁忌、限制與應配合之事項：

禁止使用的藥物

以下藥物請勿在試驗期間使用：

- 直到試驗第197天禁止使用免疫抑制劑、或細胞毒性治療
- 到試驗第197天禁止使用免疫球蛋白和/或任何血液製劑
- 整個試驗期間禁止使用試驗產品(包括藥物或疫苗)
- 到試驗第197天禁止使用全身性皮質類固醇(相當於一天使用≥ 20 mg強的松(prednisone))
- 接種試驗疫苗後14天禁止接種任何季流感疫苗或新型流感疫苗，或後28天禁止接種其他非試驗疫苗。整個試驗期間禁止使用任何已上市的新型冠狀病毒疫苗產品。

允許使用的藥物

若您的藥物或治療必須常規使用，經試驗醫師判斷不會影響本試驗疫苗的免疫原性、臨床療效與安全性，則可以正常使用。您有任何關於在試驗期間可允許使用何種藥物或治療的問題，請詢問您的試驗醫師。

懷孕或母乳哺乳的風險

目前未知本試驗疫苗對於未出生胎兒的影響，因此：

- 您為具生育能力的女性受試者 (除非手術絕育或停經)，或您為男性受試者應於接種疫苗至最後一次疫苗後 3 個月同意進行有效的避孕方式，同意進行有效的避孕方式(例如子宮內節育器、荷爾蒙療法或避孕套)。
- 若您為具生育能力的女性，將請您進行懷孕檢測，結果必須為陰性，方可參與試驗。
- 若您為懷孕的女性，將被告知不可參與本試驗。

- 若您在試驗期間懷孕，請盡速通知試驗人員，並且停止施打本疫苗。
- 基於安全性考量，若您為女性受試者而在試驗期間懷孕，或您為男性受試者而您的性伴侶在試驗期間懷孕(將請您的懷孕性伴侶需簽署另外一份同意書)，您與您的胎兒將會被追蹤監測至分娩，除非另有醫學指示。

您應向您的配偶或性伴侶告知您有參與此試驗與相關風險:

簽名：__________日期：__________________

（九)機密性：

中國醫藥大學附設醫院將依法把任何可辨識您的身分之紀錄與您的個人隱私資料視為機密來處理，不會公開。研究人員將以一個研究代碼代表您的身分，此代碼不會顯示您的姓名、國民身分證統一編號、住址等可識別資料。如果發表試驗/研究結果，您的身分仍將保密。您亦瞭解若簽署同意書即同意您的原始醫療紀錄可直接受監測者、稽核者、研究倫理委員會及主管機關檢閱，以確保臨床試驗/研究過程與數據符合相關法律及法規要求，上述人員並承諾絕不違反您的身分之機密性。除了上述機構依法有權檢視外，我們會小心維護您的隱私。由於試驗藥物可能同時申請美國臨床試驗，依美國藥品管理規定，試驗結果將公佈於公開的臨床試驗資訊網站：Clinicaltrials.gov (美國)，但您的個人資料仍將保密，該網站只會有試驗之結果摘要，您可以在任何時候搜尋該網站。

在試驗/研究期間，依據計畫類型與您所授權的內容，我們將會蒐集與您有關的病歷資料、醫療紀錄、量表、問卷等資料與資訊，並以一個編號來代替您的名字及相關個人資料。前述資料若為紙本型式，將會與本同意書分開存放於研究機構之上鎖櫃中；若為電子方式儲存或建檔以供統計與分析之用，將會存放於設有密碼與適當防毒軟體之專屬電腦內。這些研究資料與資訊將會保存至藥品於我國上市後至少兩年，若試驗疫苗終止研發則保存至試驗正式停止後至少二年，至多將保存至疫苗上市後或試驗正式停止後二年。

上述資料與資訊若傳輸至國外分析與統計，您仍會獲得與本國法規相符之保障，計畫主持人與相關團隊將盡力確保您的個人資料獲得妥善保護。

（十)損害補償與保險：

1. 如依本研究所訂臨床試驗計畫，因發生不良反應造成損害，由聯亞生技開發股份有限公司負補償責任。但本受試者同意書上所記載之可預期不良反應，不予

補償。

2. 如依本研究所訂臨床試驗計畫，因而發生不良反應或損害，贊助廠商將依法負責損害賠償責任。本醫院願意提供專業醫療照顧及醫療諮詢。您不必負擔治療不良反應或損害之必要醫療費用。
3. 除前二項補償及醫療照顧外，本研究不提供其他形式之補償。若您不願意接受這樣的風險，請勿參加試驗。
4. 您不會因為簽署本同意書，而喪失在法律上的任何權利。
5. 本研究有投保責任保險。

（十一）受試者權利：

1. 試驗過程中，與您的健康或是疾病有關，可能影響您繼續接受臨床試驗意願的任何重大發現，都將即時提供給您。
2. 如果您在試驗過程中對試驗工作性質產生疑問，對身為患者之權利有意見或懷疑因參與研究而受害時，可與本院之研究倫理委員會聯絡請求諮詢，其電話號碼為：04-22052121轉1925、1926。
3. 為進行試驗工作，您必須接受黃高彬醫師的照顧。如果您現在或於試驗期間有任何問題或狀況，請不必客氣，可與在中國醫藥大學附設醫院兒童感染科的黃高彬醫師聯絡（24小時聯繫電話：0975-681-950）。
4. 參加試驗研究計畫之補助：本計畫將在每次訪視提供交通費2000元及營養費1000元給您，整個試驗預計給予您18000元。若您願意參與T細胞檢測研究，將在該次訪視另外提供營養費500元給您。
5. 本同意書一式2份，醫師已將同意書副本交給您，並已完整說明本研究之性質與目的。醫師已回答您有關藥品與研究的問題。

（十二）試驗之退出與中止：

您可自由決定是否參加本試驗；試驗過程中也可隨時撤銷同意，退出試驗，不需任何理由，且不會引起任何不愉快或影響其日後醫師對您的醫療照顧。

計畫主持人或贊助廠商亦可能於必要時中止該試驗之進行。

（十三）簽名：

1. 計畫主持人、或協同主持人已詳細解釋有關本研究計畫中上述研究方法的性質與目的，及可能產生的危險與利益。

   計畫主持人/協同主持人簽名：____________日期：________年____月____日
2. 受試者已詳細瞭解上述研究方法及其所可能產生的危險與利益，有關本試驗計畫的疑問，業經試驗主持人詳細予以解釋。本人同意接受為臨床試驗計畫的自

願受試者。

受試者簽名：＿＿＿＿＿＿＿＿＿＿＿＿＿＿日期：＿＿＿＿年＿＿月＿＿日

法定代理人簽名：＿＿＿＿＿＿＿＿＿＿＿＿日期：＿＿＿＿年＿＿月＿＿日

* 受試者為無行為能力(未滿七歲之未成年人者或禁治產人)，由法定代理人為之；禁治產人，由監護人擔任其法定代理人。
* 受試者為限制行為人者(滿七歲以上之未成年人)，應得法定代理人之同意。

有同意權人簽名：＿＿＿＿＿＿＿＿＿＿＿＿日期：＿＿＿＿年＿＿月＿＿日

* 受試者雖非無行為能力或限制行為能力者，但因意識混亂或有精神與智能障礙，而無法進行有效溝通和判斷時，由有同意權之人為之。前項有同意權人為配偶及直系親屬。

3. 見證人

見證人簽名：＿＿＿＿＿＿＿＿＿＿＿＿＿＿日期：＿＿＿＿年＿＿月＿＿日

身分證字號：　　　　　　　　　　　聯絡電話：

通訊地址：

* 受試者、法定代理人或有同意權之人皆無法閱讀時，應由見證人在場參與所有有關受試者同意之討論。並確定受試者、法定代理人或有同意權之人之同意完全出於其自由意願後，應於受試者同意書簽名並載明日期。試驗相關人員不得為見證人。

中國醫藥大學暨附設醫院
受試者同意書
（成年免疫組）

流程表:

批次分析與免疫分析組

訪視	1[m]	2 [m]		3		4		5/提早退出試驗	6[t]		7		長期性追蹤
**檢測項目**	**篩選**	**第一次接種**		**第二次接種**		**追蹤** [g]		**個別解盲**	**第三次接種** [h]		**追蹤** [h]		**第 12 個月追蹤** [i]
**天數**	**-28~-1**	**1**	**8, 15, 22**	**29 ±3 天**	**36, 43**	**57 ±3 天**	**64, 71, 78,85**	**197 ±15 天**	**197~242**	**第六次訪視後 7 天**	**第六次訪視後 14 天 ±3 天**	**253, 309**	**365 ±45 天**
獲得受試者同意書	X								**X**[h]				
納入/排除條件	X	X											
隨機分派		X											
**接種評估**				X					**X** [h]				
基本資料	X												
醫療病史	X	X											
身體檢查 [a]	X	X		X		X		X	**X** [h]		**X** [h]		
生命徵象	X	X		X		X		X	**X** [h]		**X** [h]		**X**
心電圖	X												
**實驗室檢測 (安全性)**													
血液常規檢測 [j]	X					X			**X** [h]		**X** [h]		
血液生化學檢測 [j]	X					X			**X** [h]		**X** [h]		
免疫檢測 [j]	X					X			**X** [h]		**X** [h]		
懷孕檢測 [b]		X		X					**X** [h]				

訪視	1[m]	2 [m]		3		4		5/提早退出試驗	6[t]		7		長期性追蹤
**檢測項目**	**篩選**	**第一次接種**		**第二次接種**		**追蹤 [g]**		**個別解盲**	**第三次接種 [h]**		**追蹤 [h]**		**第 12 個月追蹤 [i]**
**天數**	**-28~-1**	**1**	**8, 15, 22**	**29 ±3 天**	**36, 43**	**57 ±3 天**	**64, 71, 78,85**	**197 ±15 天**	**197~242**	**第六次訪視後 7 天**	**第六次訪視後 14 天 ±3 天**	**253, 309**	**365 ±45 天**
尿液常規檢測 [b]	X	X [q]		X [q]		X [q]		X [q]	**X [h, q]**		**X [h, q]**		
**實驗室檢測(免疫原性)**													
免疫原性 [c]		X		X [n]		X		X					X
T 細胞反應 (可選擇 [o])		X				X							
**實驗室檢測(探索性試驗)**													
**T 細胞功能性檢測(可選擇 [u])**									**X [h]**		**X[h]**		
**備血**	X					X							
**免疫原性 [c]**									**X [h]**		**X[h]**		
疫苗接種		X		X					**X [h]**				
指導使用電子日誌卡		X		X					**X [h]**				
電話安全性追蹤 [d]			X		X		X			**X[s ,h]**		X	
不良事件/特殊不良事件 [p]/醫		X [k]	X [k]	X [k]	X [k]	X [k]	X [k]	X [l]	**X [l, h]**	**X [l, h]**	**X [l, h]**	X [l]	X [l]

中國醫藥大學暨附設醫院

受試者同意書

（成年免疫組）

訪視	1[m]	2 [m]		3		4		5/提早退出試驗	6[t]		7		長期性追蹤
檢測項目	篩選	第一次接種		第二次接種		追蹤 [g]		個別解盲	第三次接種 [h]		追蹤 [h]		第 12 個月追蹤 [i]
天數	-28~-1	1	8, 15, 22	29 ±3 天	36, 43	57 ±3 天	64, 71, 78,85	197 ±15 天	197~242	第六次訪視後 7 天	第六次訪視後 14 天 ±3 天	253, 309	365 ±45 天
療需求不良事件/嚴重不良事件													
新型冠種病毒感染監測		X	X	X	X	X [e]	X [e]	X [e]	**X** [e, h]	**X** [e, h]	**X** [e, h]	X [e]	X [e]
併用藥物		X		X		X		X [f]	**X** [f, h]		**X** [f, h]		

a: 身高與體重僅在第一次訪視測量。
b:於第 1, 29 天使用尿液懷孕檢測。若尿液檢測為陽性，應以血清懷孕檢測再次確認。以血清懷孕檢測替代尿液檢測將不視為試驗偏差。蛋白尿將透過尿液常規檢測確認，做為基礎值。
c: 針對 Anti-S1-RBD 免疫球蛋白 G 濃度和新型冠狀病毒中和抗體效價，以及抑制 S1-RBD: ACE2 的抗體效價。
d: 所有受試者將進行電話安全追蹤以監測非預期性不良事件，包括特殊不良事件，和監測新型冠狀病毒感染的症狀。
e: 受試者將在每周(為每 7 天)由手機接獲一條提示以規律監測新型冠狀病毒感染症狀或病徵。記錄疑似新冠病毒感染所使用的藥物至新冠病毒感染頁面。
f: 只記錄醫療需求不良事件和嚴重不良事件之併用藥物。
g: 第二次接種疫苗後第 28 天
h: 僅針對同意並進行施打第三劑試驗疫苗的疫苗組受試者
i: 第二次接種疫苗後第十二個月(第 365 天)
j: 安全性實驗室數值包括全血液計數(血紅素、血比容、紅血球計數)、白血球計數、血小板計數、肌酸酐、丙胺酸轉胺酶、天門冬胺酸轉胺酶、總膽紅素、直接膽紅素、高靈敏度 C 反應性蛋白、抗核抗體
k: 主動收集時期
l: 被動監測時期
m: 第一次訪視與第二次訪視可為同一次訪視。
n: 僅測量 Anti-S1-RBD IgG 濃度
o: 將在選定的試驗地點納入至少 100 位>18-<65 歲的受試者。
p : 包括接種最後一劑疫苗後 12 個月，可能的免疫媒介醫療狀況(PIMMC)或任何定義為可能的特殊不良事件。新冠病毒感染的併發症也將視為疾病增強事件，將被記錄與通報為特殊不良事件。
q: 若受試者有≥第三級以上的高血壓，受試者將確認是否有蛋白尿存在或惡化。
r: 備血將被冷凍存放做為 UBI 新型冠狀病毒酵素結合免疫吸附分析法，新型冠狀病毒確認酵素結合免疫吸附分析法，以及未來免疫學研究之用。
s: 若符合資格並接種第三劑疫苗的受試者將進行兩週的日誌卡追蹤紀錄，並將於接種後第 7 天進行電話安全性追蹤。

t: 第五次訪視和第六次訪視可為同一天。

u: 在選定之試驗地點中，將邀請大約 30 位年齡 >18-<65 歲的受試者和大約 30 位年齡 ≥65 歲的受試者。在適用的情況下，優先邀請曾進行過第 57 天 T 細胞功能評估的受試者。

中國醫藥大學暨附設醫院
受試者同意書
(成年安全確認組)

您被邀請參與此研究。此同意書主要是提供您本研究之相關資訊，以便您決定是否參加本研究。計畫主持人或其指定之研究人員會為您說明研究內容並回答您的疑問。您可以提出任何和此研究有關的問題，在您的問題尚未獲得滿意的答覆之前，請不要簽署此同意書。如果您願意參與本研究，此文件將視為您的同意紀錄。即使在您同意後，您可以隨時退出本研究不需任何理由。

<table>
<tr><td colspan="2">計畫名稱
中文：一個評估 UB-612 疫苗對於新型冠狀病毒於青少年、成年和老年健康受試者的免疫原性、安全性與耐受性的第二期、安慰劑控制、隨機分派、觀察者盲性臨床試驗
英文：A Phase II, Placebo-controlled, Randomized, Observer-blind Study to Evaluate the Immunogenicity, Safety and Tolerability of UB-612 Vaccine against COVID-19 in Adolescent, Younger and Elderly Adult Volunteers</td></tr>
<tr><td>執行單位：中國醫藥大學附設醫院感染科、家庭醫學科</td><td>委託單位/藥廠：聯亞生技開發股份有限公司
研究經費來源：聯亞生技開發股份有限公司
受託研究機構：晉加股份有限公司</td></tr>
<tr><td>計畫主持人：黃高彬</td><td>職稱：主治醫師</td></tr>
<tr><td>協同主持人：林文元</td><td>職稱：主治醫師</td></tr>
<tr><td>協同主持人：林伯昌</td><td>職稱：主治醫師</td></tr>
<tr><td>緊急聯絡人：黃高彬</td><td>電話：0975-681-950</td></tr>
<tr><td>受試者姓名：
性別：
身分證字號：
通訊地址：</td><td>病歷號碼：
出生日期：
聯絡電話：</td></tr>
<tr><td>法定代理人或有同意權人之姓名：
性別：
身分證字號：
通訊地址：</td><td>與受試者關係：
出生日期：
聯絡電話：</td></tr>
<tr><td colspan="2">(一)試驗簡介：
1. 本品/技術資料：</td></tr>
</table>

新型冠狀病毒(SARS-CoV-2)於2019年12月起造成中國湖北省武漢市發現多起病毒性肺炎群聚，隨後於2020年1月底台灣出現第一起境外移入確診個案。此疾病在全球擴散，世界衛生組織宣布將此疫情為「國際關注公共衛生緊急事件」。截至2020年底，全球僅有數間疫苗公司，如美國輝瑞藥廠等，取得緊急使用授權上市。

UB-612疫苗為聯亞生技開發股份有限公司所開發新型冠狀病毒預防性疫苗，疫苗含病毒棘狀融合蛋白和胜肽片段，可產生高親和力抗體與新型冠狀病毒結合，並誘發細胞免疫反應，進而達到預防新型冠狀病毒的感染。

UB-612第一期延伸性試驗顯示，接種第三劑UB-612疫苗可以誘發極高的中和抗體，在目前變種病毒的威脅之下，施打第三劑加強免疫反應，已是許多國家的選擇。

2. 本品上市狀況：

本品仍應用於人體試驗，尚未在我國上市。

3. 本試驗使用的UB-612疫苗對新型冠狀病毒的預防效果仍未確認。

(二)試驗目的：

主要試驗目的

- 評估UB-612疫苗誘發的新型冠狀病毒中和抗體效價。
- 評估接種UB-612疫苗後的安全性和耐受性。

次要試驗目的

- 評估在試驗期間對於新型冠狀病毒的免疫反應。
- 評估三批獨立批次疫苗的批次免疫一致性。

探索性試驗目的

- 評估UB-612疫苗誘發的T細胞功能。
- 評估UB-612疫苗在年輕受試者的安全性和免疫原性。
- 評估UB-612疫苗的療效。
- 描述UB-612疫苗於確診和/或嚴重感染新型冠狀病毒案例之血液學反應。
- **評估針對SARS-CoV-2抗原的抗體反應。**

（三)試驗之主要納入與排除條件：

中國醫藥大學暨附設醫院執行本研究計畫的醫師或相關研究人員將會與您討論有關參加本研究的必要條件。請您配合必須誠實告知我們您過去的健康情形，若您有不符合參加本研究的情況，將不能參加本研究計畫。

1. 參加本研究計畫的主要條件:

□(1) 您為納入試驗時20~85歲之間健康男性或未懷孕的女性受試者。

□(2) 您為具生育能力的女性與男性應於首次接種疫苗至最後一次疫苗後3個月同意進行有效的避孕方式。可接受的有效避孕方式包括:

□a. 男性或女性以手術方法絕育、植入式避孕、或子宮避孕器。

□b.注射避孕、避孕藥、避孕貼片、避孕環加上一種屏障避孕法*。

□c.合併使用兩種屏障避孕法*。

*有效的屏障避孕法為避孕隔膜、男性或女性保險套、避孕海綿或殺精劑(含可殺精化學物質的藥膏或凝膠)。

□(3) 您能理解受試者同意書內容的說明與可能的風險，提供簽名的受試者同意書。

□(4) 您能夠理解與遵從本試驗程序與能夠參與每次訪視。

□(5) 您的耳溫≤ 38.0°C。

□(6) 您依據醫療病史、身體檢查和試驗主持人的臨床判斷為健康受試者**可符合納入試驗資格。經試驗主持人判斷，即便您的病史穩定或且控制良好，但伴隨病情惡化而有提高嚴重新型冠狀病毒感染的風險。

**健康受試者有先前存在的穩定疾病者可以納入試驗，定義為該疾病在納入試驗前12週內沒有惡化至需要治療或住院的顯著變化和在納入試驗6個月內沒有惡化至需要治療或住院的顯著變化。

2. 若您有下列任一情況，您將無法參加本研究計畫:

□(1) 您有接種疫苗後需要醫療介入的過敏性休克、蕁麻疹或其他顯著不良反應的病史。

□(2) 您在篩選時或接種每劑疫苗前已懷孕女性或懷孕檢測為陽性的女性。

□(3) 您為正在哺乳的女性，或計畫從接種第一劑疫苗至最後一劑疫苗後60天哺乳的女性。

□(4) 您在接種第一劑疫苗前3天內，經試驗主持人判斷，患有任何急性疾病。

□(5) 您在接種第一劑疫苗前1個月內有重大手術。

□(6) 您是已知為人類免疫缺乏病毒抗體陽性。

□(7) 您是已知為活動性B型肝炎或C型肝炎。活動性肝炎定義為肝臟轉胺酶(天門冬胺酸轉胺酶和/或丙胺酸轉胺酶)大於3倍正常值上限和/或總膽紅素大於3倍正常值上限。

□(8) 您是已知曾暴露於新型冠狀病毒，或曾接受預防新型冠狀病毒、中東呼吸症候群冠狀病毒、嚴重急性呼吸道症候群的試驗或已上市產品。

□(9) 您有格林-巴利症候群的病史。

□(10) 您在簽署受試者同意書前12周內參與其他的臨床試驗。

□(11) 您為免疫缺乏/失調疾病，無論是否由基因缺陷、免疫缺乏症或免疫抑制療法所造成。

□(12) 您計畫或正在進行抗癌症治療。

□(13) 您患有血小板異常或其他凝血異常可能造成注射之禁忌症。

□(14) 您在接種第一劑疫苗前6個月長期接受(≥14天連續使用)免疫抑制劑、皮質類固醇(相當於一天使用≥ 20 mg強的松(prednisone))或細胞毒性治療。

□(15) 您在接種第一劑疫苗前4個月接受免疫球蛋白和/或任何血液製劑的治療。

□(16) 您在接種試驗疫苗前14天接種任何季流感疫苗或新型流感疫苗，或前28天接種其他疫苗。

□(17) 您預期在接種試驗疫苗後14天接種任何季流感疫苗或新型流感疫苗，或後28天接種其他疫苗。

□(18) 您使用短期(<14天使用)全身性類固醇。應於中斷使用全身性類固醇至少28天後才可使用試驗疫苗。吸入/噴霧性、關節注射、囊內或局部(皮膚或眼用)類固醇可允許使用。

□(19) 您在篩選期前3個月失血或捐血超過500毫升，或預計在試驗期間內捐血或輸血。

□(20) 經試驗主持人判斷，您有任何醫療疾病或狀況，可能會影響試驗結果或參與試驗可能會對受試者引發額外風險。

□(21) 您是直接參與本試驗執行的試驗主持人所屬機構**的試驗團隊、試驗委託者或受託**

研究機構(CRO)的員工。

（四）試驗方法及相關檢驗：

這是一個第二期、觀察者盲性、多中心、隨機分派、安慰劑控制試驗，以評估青少年，成年和老年受試者使用兩劑UB-612疫苗的免疫原性，耐受度和安全性。有一部份的受試者使用UB-612疫苗，而另外一部份的受試者則使用「安慰劑」。所謂「安慰劑」是不含有效成份的疫苗。至於誰使用試驗用藥或誰使用「安慰劑」，則像丟銅板或擲骰子一樣由機率決定，不管是您或是研究醫師都不知道您使用了那一種藥，只有分發跟施打疫苗的試驗人員才知道您使用哪一種疫苗，這叫做觀察者盲性。

總計約有3850位合格成年受試者組成核心組用於申請緊急使用授權，另外大約有385位青少年受試者組成補充組申請額外適應症。所有受試者將以6:1的比例，隨機分派至兩劑100微克劑量組別和安慰劑組，包括462位大於18歲至小於65歲可評估的受試者進入批次分析組。對於免疫分析，至少包括350位可評估的成年受試者(年齡大於18歲至小於65歲)和154位可評估的老年受試者(年齡≥65歲)進行描述性分析。免疫原性的受試者將會先納入試驗。所有的受試者將會納入安全性分析，其中至少770位隨機分派的受試者為≥65歲的分層。青少年組將在核心組招募完畢後，再開始納入試驗。約有385位青少年受試者將以6:1的比例隨機分派，其中包括154位可評估的青少年受試者將收集免疫原性數據，並和成年及老年受試者數據進行比較。

您若參與這個試驗，則為進行安全性確認的安全確認組。

試驗總共有8個訪視。若您參與本試驗，則至少包括第一次訪視(篩選訪視)、第二次訪視（第1天，基礎值，隨機分派，第一次接種疫苗)、第三次訪視(第29天，第二次接種疫苗)、第四次訪視(第57天)、第五次訪視(第197天)。

在第五次訪視時，預計將進行個別解盲。解盲後，得知您為施打疫苗的受試者，且您有意願且符合資格接種第三劑疫苗，將請您簽署另外一份受試者同意書，以進行後續程序；包含第六次訪視(第197~242天，第三次接種疫苗)，第七次訪視(接種疫苗後第14天)，及第八次訪視(第365天)。

若個別解盲後，得知您為施打疫苗的受試者，您不願意進行第三劑疫苗接種，將進行第八次訪視(第365天)的安全性追蹤。

若個別解盲後，得知您為施打安慰劑組的受試者，將結束您的試驗。

整個試驗期間，將預期您將參與試驗最長達13個月。

中國醫藥大學暨附設醫院

受試者同意書

（成年安全確認組）

注意事項

1. 如果您同意參加本試驗，研究人員會請您簽署本份受試者同意書，並確認您符合參加本試驗的條件。
2. 從您參與試驗的當天開始，每次訪視都將有合格的試驗人員執行試驗流程與聯繫。
3. 若您有任何符合新型冠狀病毒感染的定義(您曾於過去 7 天內有出國，或是接觸疑似或確認武漢肺炎之病人，而有下列症狀: 發燒、開始咳嗽或惡化、開始呼吸急促或惡化、寒顫、開始肌肉疼痛或惡化、喉嚨痛、腹瀉、嘔吐、開始味覺/嗅覺異常)，請**依照中央疫情指揮中心規定進行自主健康管理或至指定院所進行篩檢。**
4. 若您在試驗期間感染新型冠狀病毒，將依法通報主管機關。

   您是否同意? □是 □否

   簽名：__________日期：__________
5. 於試驗期間，您不論任何理由提前退出試驗，試驗研究人員都將安排您完成最後一次的訪視之所有試驗項目。您有權利拒絕此項安排，您的決定不會引起任何影響日後醫師對您的醫療照護。

試驗步驟

第一次訪視(第-28~-1 天) -篩選訪視

在試驗醫師或試驗研究人員為您提供足夠的試驗資訊，並確保您有充分的時間考慮以及詢問任何問題後，您願意讓您參與本試驗，並由您簽署本受試者同意書。在確認您已完成受試者同意書簽署並且您也保有一份副本後，試驗醫師或試驗研究人員將會進行以下試驗程序：

(1) 記錄您簽署受試者同意書的日期

(2) 為您指定一組受試者篩選編號

(3) 確認您是否符合本試驗的納入排除條件

(4) 收集您的個人基本資料（例如生日、年齡及性別）

(5) 記錄您的醫療/用藥病史

(6) 進行身體檢查，包括身高體重

(7) 確認生命徵象

(8) 進行心電圖檢測

(9) 收集尿液檢體進行尿液檢測

(10) 收集血液檢體(共 **15.5** 毫升)，進行下列檢測:

- 常規血液檢測
- 血液生化學檢測
- 免疫學檢測**(抗核抗體)**
- **備血(作為測量新型冠狀病毒血清抗體和相關研究之用)**

第二次訪視(第 1 天)-基礎值，確認符合試驗條件，第一次接種疫苗

(1) 再度確認您是否符合本試驗的納入排除條件

(2) 隨機分派，給予您一組隨機分派號碼

(3) 記錄您的醫療/用藥病史

(4) 進行身體檢查

(5) 確認生命徵象

(6) 進行尿液懷孕檢測（具有生育能力女性）

(7) 若您有產生第三級以上的高血壓，您將被收集尿液檢體以檢測是否有蛋白尿存在或惡化

(8) 進行第一次疫苗接種(注射部位為非慣用手，採用肌肉注射方式)。接種疫苗後，受試者應留在試驗地點至少 30 分鐘，監測生命徵象和急性過敏症狀。

(9) 將詳細地指導您如何填寫電子日誌卡(包括注射後 7 天內預期性不良事件，14 天內的皮膚過敏反應日誌卡)

(10) 收集併用藥物/治療

第一次電話安全性追蹤(第 8, 15, 22 天)

將與您電話聯繫，以追蹤未預期不良事件和新型冠狀病毒感染症狀。

第三次訪視(第 29± 3 天)-第二次接種疫苗

(1) 進行第二次接種評估(有可能會延遲接種時間)

(2) 進行身體檢查

(3) 確認生命徵象

(4) 進行尿液懷孕檢測（具生育能力的女性）

(5) 若您有產生第三級以上的高血壓，您將被收集尿液檢體以檢測是否有蛋白尿存在或惡化。

(6) 進行第二次疫苗接種。

(7) 接種疫苗後，受試者應留在試驗地點至少 30 分鐘，監測生命徵象和急性過敏症

狀。

(8) 將詳細地指導您如何填寫電子日誌卡(包括注射後 7 天內預期性不良事件，14 天內的皮膚過敏反應日誌卡)

(9) 收集併用藥物/治療

(10) 記錄上一次訪視至此次訪視之間的不良事件、嚴重不良事件型或新型冠狀病毒感染症狀

<u>第二次電話安全性追蹤(第 36, 43 天)</u>

將與您電話聯繫，以追蹤未預期不良事件和新型冠狀病毒感染症狀。

<u>第四次訪視(第 57±3 天) –追蹤訪視</u>

(1) 進行身體檢查

(2) 確認生命徵象

(3) 若您有產生第三級以上的高血壓，您將被收集尿液檢體以檢測是否有蛋白尿存在或惡化。

(4) 收集血液檢體(共 **15.5** 毫升) ，進行下列檢測:

- 常規血液檢測
- 血液生化學檢測
- 免疫學檢測**(抗核抗體)**
- **備血(作為測量新型冠狀病毒血清抗體和相關研究之用)**

(5) 收集併用藥物/治療

(6) 記錄上一次訪視至此次訪視之間的不良事件、嚴重不良事件或新型冠狀病毒感染症狀

(7) 在此次訪視後，您將每周接獲訊息提醒，以定期監測新型冠狀病毒感染症狀至第 197 天。

<u>第三次電話安全性追蹤(第 64, 71, 78, 85 天)</u>

將與您電話聯繫，以追蹤安全性及新型冠狀病毒感染症狀。

<u>第五次訪視(第 197±15 天) –個別解盲</u>

(1) 進行身體檢查

(2) 確認生命徵象

(3) 若您有產生第三級以上的高血壓，您將被收集尿液檢體以檢測是否有蛋白尿存在或惡化。

(4) 將替您個別解盲，告知您接種到疫苗或安慰劑。若您為安慰劑組，您已完成本試驗，將協助您退出試驗。但若您為疫苗組且符合資格，我們將提供另外一份受試

者同意書，邀請您參與後續的第三劑接種試驗。若您不同意接種第三劑，您仍需進行後續的安全性追蹤。

(5) 收集併用藥物/治療

(6) 記錄上一次訪視至此次訪視之間的不良事件、嚴重不良事件或新型冠狀病毒感染症狀

第四次電話安全性追蹤(第 253, 309 天)

第 197 天後將每兩個月與您電話聯繫，以追蹤安全性及新型冠狀病毒感染症狀。

最後一次訪視(第 365±45 天) –第 12 個月追蹤

(1) 確認生命徵象

(2) 記錄上一次訪視至此次訪視之間的不良事件，包括特殊不良事件、醫療不良事件、嚴重不良事件或新型冠狀病毒感染症狀

受試者之檢體(含其衍生物)之保存、使用與再利用：

1. 檢體及剩餘檢體之保存與使用

(1) 檢體(含其衍生物)之保存與使用

為研究所需，我們所蒐集您的檢體，將依本研究計畫使用，檢體將保存於**聯亞生技開發股份有限公司(試驗委託者)**，直至 20 年保存期限屆滿，我們將依法銷毀。為了保護您的個人隱私，我們將以一個試驗編號來代替您的名字及相關個人資料，以確認您的檢體及與相關資料受到完整保密。如果您對檢體的使用有疑慮，或您有任何想要銷毀檢體的需求，請立即與我們聯絡(聯絡人：黃高彬醫師電話：0975-681-950)，我們即會將您的檢體銷毀。您也可以聯繫中國醫藥大學暨附設醫院研究倫理委員會(電話：04-22052121 轉 1925、1926)，以協助您解決檢體在研究使用上的任何爭議。

(2) 剩餘檢體(含其衍生物)之再利用

您的生物檢體將會以專屬號碼進行編碼並在**聯亞生技開發股份有限公司(試驗委託者)**的控管下儲存最長20年，以研究UB-612 疫苗反應者的生物標記，及改善治療方式。

所有新的研究計畫都要再經由中國醫藥大學暨附設醫院研究倫理委員會審議通過，倫理審查委員會若認定新的研究超出您同意的範圍，將要求我們重新得到您的同意。

是否同意剩餘檢體保留提供未來新型冠狀病毒感染研究之用，並授權中國醫藥大學

暨附設醫院研究倫理委員會審議是否需要再取得您的同意(擇一)

☐ 不同意保存我的剩餘檢體，試驗結束後請銷毀

☐ 同意以非去連結之方式保存我的剩餘檢體，逾越原同意使用範圍時，需再次得到我的同意才可使用我的檢體進行新的研究

2. 檢體及剩餘檢體之部分類型(檢體類型可依計畫書內容自行增減)

(1) 一般生化、血液檢驗/病毒檢測檢體

在試驗期間，會將您的檢體送往聯亞生技開發股份有限公司(試驗委託者)委託的中央實驗室中國醫藥大學暨附設醫院，此機構地址為台中市北區育德路2號，和大安聯合醫事檢驗所，此機構地址為台北市大安區復興南路二段151巷33號，中央實驗室會在分析後立即將分析結果提供給試驗中心，若有剩餘的檢體，**將儲存直到至少完成臨床試驗報告為止，最長將保存20年。**

若在研究期間安排您回診確認COVID-19感染，剩餘檢體保存與使用說明如下：

(2) 抗體/細胞免疫試驗

在試驗期間，會將您的檢體送往聯亞生技開發股份有限公司(試驗委託者)分析實驗室。完成試驗後，若有剩餘檢體，將儲存直到至少完成臨床試驗報告為止，最長將保存20年。

(3) 中和試驗(neutralization test, NT)

在試驗期間，會將您的檢體送往聯亞生技開發股份有限公司(試驗委託者)委託的中央實驗室中央研究院進行處置、處理與進一步分析。此機構地址為台北市南港區研究院路二段 128 號。完成試驗後，若有剩餘檢體，將儲存直到至少完成臨床試驗報告為止，最長將保存 20 年。

(4) 遺傳學檢體

在試驗期間，若發生嚴重不良反應或特定不良反應，您的檢體將用於 HLA 分型檢驗，會將您的檢體送往聯亞生技開發股份有限公司(試驗委託者)委託的中央實驗室有勁基因股份有限公司分析，此機構地址為新北市樹林區復興路 376-5 號，中央實驗室不會將分析結果提供給試驗中心，若有剩餘的檢體，將會儲存直到檢驗結果複驗完畢即銷毀，

不會長期儲存。

(5) 探索性試驗檢體

在試驗期間，會將您的檢體送往聯亞生技開發股份有限公司(試驗委託者)委託的實驗室(表一)進行處理或進一步分析。完成試驗後，若有剩餘檢體，將儲存直到至少完成臨床試驗報告為止，最長將保存 20 年。

表一、實驗室名稱與機構地址

實驗室名稱	機構地址
聯亞生技開發(股)公司	新竹縣竹北市生醫路二段 6-1 號 5 樓
Viroclinics	Rotterdam Science Tower, Marconistraat 16, 3029 AK Rotterdam, The Netherlands(荷蘭)
DASA	Jonas Cruz de Araujo, Diagnostics da America S/A, Surubiju Avenue, 1890, Barueri, SP, Brazil(巴西), 06455-040
PHE Porton Down	Salisbury Wiltshire SP4 0JG, England(英國)
UTMB	University of Texas Medical Branch 301 University Boulevard Keiller Building, Room 2.150 Galveston, Texas, USA(美國
Virology	University of São Paulo, Brazil Rua Dr EnnEn de Carvalho Aguiar 470, CEP 05403-000 (巴西)
VRDL	California Department of Public Health, 850 Marina Bay Parkway, Richmond, CA 94804, USA(美國)
NEXELIS	525 Boul. Cartier Ouest Laval, Qulbec, Canada, H7V 3S8(加拿大)
Vaccinology and Immunology Infection, Immunity & Inflammation Dept UCL GOS Institute of Childe Health	UCL Great Ormond Street Institute of Child Health 30 Guilford Street London WC1N 1EH, England(英國)
VisMederi	VisMederi Srl, Strada del Petriccio e Belriguardo, 35, 53100 Siena, Italy(義大利)

(五)可能產生之副作用、發生率及處理方法：

1. 與試驗藥物相關的風險（本試驗疫苗的副作用）：

<u>**冠狀病毒疫苗的開發**</u>

過去針對與SARS-CoV-2病毒相同屬於人類冠狀病毒的SARS-CoV(嚴重急性呼吸綜合症冠狀病毒(SARS冠狀病毒))的疫苗研究發現，接種過SARS-CoV疫苗的小鼠在暴露到SARS-CoV後會發生過度免疫反應而產生病變，因此不得不停止這種疫苗的開發。所以，

成功的人類冠狀病毒疫苗不只要產生可以抑制病毒的免疫反應，更要避免過度免疫產生的副作用。

疫苗相關的風險: 第一期臨床試驗

接種疫苗可能會出現注射部位的不良反應(例如疼痛、硬化腫脹、皮疹發紅、過敏反應、蜂窩性組織炎)，或全身性不良反應(例如發燒、腹瀉、疲倦、噁心/嘔吐、厭食、咽喉痛、頭痛、咳嗽、關節痛、非注射部位疼痛、非注射部位搔癢、皮膚和黏膜異常、急性過敏反應、昏厥、急性支氣管痙攣、呼吸困難)。

第一期臨床試驗已經有60位受試者接種兩劑疫苗(含10微克、30微克、100微克融合蛋白)，安全實驗室數值並沒有顯示有任何的臨床顯著不正常數值，也沒有發生任何第三級以上與疫苗相關的預期性不良事件。大部分的預期性不良事件都是輕微的，症狀在大約於2天之內都會緩解。試驗中也沒有任何的嚴重不良事件或特殊不良事件被通報。

疾病增強(disease enhancement) 的風險

SARS-CoV-2候選疫苗也可能會有引發疾病增強(disease enhancement) 的風險，包括抗體依賴性增強(antibody-dependent enhancement)或疫苗相關聯的增強的呼吸道疾病(vaccine-associated enhanced respiratory disease)。在先前研發SARS疫苗時，在數個SARS-CoV動物攻毒試驗(包括鼠類、雪貂、猴類)當中，有發現疾病增強的現象。疾病增強反應的免疫病理現象包括TH2偏向及嗜酸性白血球的肺部浸潤。但是目前已發表的新型冠狀病毒肺炎疫苗研究，仍尚未發現類似的疾病增強現象。

本試驗疫苗於數個藥理試驗呈現不一致的TH1/TH2 (輔助型T細胞1/輔助型T細胞2)免疫反應偏向，試驗結果並未一致偏向TH2，而由小鼠之SARS-CoV-2動物攻毒試驗結果顯示，本試驗疫苗誘發疾病增強之風險不高。依據文獻指出，組成複雜或容易引起非中和抗體之抗原，如不活化病毒或整片段之蛋白(包含S蛋白與N蛋白)，與易引起偏向Th2免疫反應之佐劑成分，如鋁製佐劑，皆較有可能引起疾病增強。本試驗疫苗的主要抗原為S蛋白上之RBD 區域，已有多篇文獻指出，針對S-RBD設計之SARS與MERS疫苗從未於試驗動物模型上引發疾病增強現象。本試驗疫苗雖使用易引起偏向Th2免疫反應之佐劑，但由動物實驗證實，也同時引起偏向Th1之反應，因此發生疾病增強應屬低風險。且已於多種動物模型中證實，能誘發高效價之中和抗體，於細胞培養中亦能有效抑制新冠病毒感染。

建議您在有效疫苗上市前或本試驗疫苗的產品資訊有進一步更新前，盡量避免暴露於可能感染病毒的環境。研究團隊將會在試驗中執行相關安全性監測。若有任何關於本試驗疫苗與疾病增強風險相關之任何最新資訊，將即時更新並提供給您。

疫苗佐劑相關的風險

本試驗疫苗所使用的佐劑含Adju-Phos®，是屬於一種磷酸鋁類的佐劑。磷酸鋁類佐劑已

經使用超過半個世紀，具有相當的安全性。由於此類佐劑可誘導免疫反應，因此可能會造成局部發炎反應，例如在注射部位產生輕微而短暫的疼痛、發紅以及腫脹。

2. 與試驗/研究過程相關的風險：

抽血

本試驗需要抽血檢驗。抽血可能引起一些不適和瘀血。整個試驗期間13個月，共需抽血**31毫升**。若您罹患新型冠狀病毒感染，可能將於每次額外訪視抽血30毫升，預計共額外抽血60毫升。

在接種疫苗過程中，可能會出現一些尚未在已完成試驗中發現的副作用。一般而言，接種某一新疫苗總是會有一定的風險，但是計畫主持人會採取一切措施預防風險的發生。**計畫主持人鼓勵您報告您遇到的任何不適。**

（六）其他替代療法及說明：

您不是非參加不可，若不參加研究，由於目前尚未有疫苗可用來預防新型冠狀病毒感染，因此預防措施與其他呼吸道感染相同，包括：勤洗手、減少觸摸眼口鼻、注意咳嗽禮節、妥善處理口鼻分泌物等，避免出入公共場所，並不要接觸野生動物。

如果您對於本試驗疫苗有任何的疑問，您可以提出來向您的試驗醫師討論。

（七）試驗預期效益：

依據臨床前試驗結果，預期本試驗疫苗對您可能可以產生抗體，預防新型冠狀病毒感染，但因每個人體質不同也有可能不會產生療效，故參加本試驗可能不會有直接的好處。

但是您參加本試驗，可協助我們獲得更多資訊，以瞭解UB-612疫苗的安全性與免疫力。

（八）試驗進行中受試者之禁忌、限制與應配合之事項：

禁止使用的藥物

以下藥物請勿在試驗期間使用：

- 直到試驗第57天禁止使用免疫抑制劑、或細胞毒性治療
- 到試驗第57天禁止使用免疫球蛋白和/或任何血液製劑
- 整個試驗期間禁止使用試驗產品(包括藥物或疫苗)
- 到試驗第57天禁止使用全身性皮質類固醇(相當於一天使用≥ 20 mg強的松

(prednisone))

- 接種試驗疫苗後14天禁止接種任何季流感疫苗或新型流感疫苗，或後28天禁止接種其他非試驗疫苗。整個試驗期間禁止使用任何已上市的新型冠狀病毒疫苗產品。

允許使用的藥物

若您的藥物或治療必須常規使用，經試驗醫師判斷不會影響本試驗疫苗的安全性，則可以正常使用。您有任何關於在試驗期間可允許使用何種藥物或治療的問題，請詢問您的試驗醫師。

懷孕或母乳哺乳的風險

目前未知本試驗疫苗對於未出生胎兒的影響，因此：

- 您為具生育能力的女性受試者 (除非手術絕育或停經)，或您為男性受試者應於接種疫苗至最後一次疫苗後 3 個月同意進行有效的避孕方式，同意進行有效的避孕方式(例如子宮內節育器、荷爾蒙療法或避孕套)。
- 若您為具生育能力的女性，將請您進行懷孕檢測，結果必須為陰性，方可參與試驗。
- 若您為懷孕的女性，將被告知不可參與本試驗。
- 若您在試驗期間懷孕，請盡速通知試驗人員，並且停止施打本疫苗。
- 基於安全性考量，若您為女性受試者而在試驗期間懷孕，或您為男性受試者而您的性伴侶在試驗期間懷孕(將請您的懷孕性伴侶需簽署另外一份同意書)，您與您的胎兒將會被追蹤監測至分娩，除非另有醫學指示。

您應向您的配偶或性伴侶告知您有參與此試驗與相關風險:

簽名：__________日期：__________________

(九)機密性：

中國醫藥大學附設醫院將依法把任何可辨識您的身分之紀錄與您的個人隱私資料視為機密來處理，不會公開。研究人員將以一個研究代碼代表您的身分，此代碼不會顯示您的姓名、國民身分證統一編號、住址等可識別資料。如果發表試驗/研究結果，您的身分仍將保密。您亦瞭解若簽署同意書即同意您的原始醫療紀錄可直接受監測者、稽核者、研究倫理委員會及主管機關檢閱，以確保臨床試驗/研究過程與數據符合相關法律及法規要

求，上述人員並承諾絕不違反您的身分之機密性。除了上述機構依法有權檢視外，我們會小心維護您的隱私。由於試驗藥物可能同時申請美國臨床試驗，依美國藥品管理規定，試驗結果將公佈於公開的臨床試驗資訊網站：Clinicaltrials.gov (美國)，但您的個人資料仍將保密，該網站只會有試驗之結果摘要，您可以在任何時候搜尋該網站。

在試驗/研究期間，依據計畫類型與您所授權的內容，我們將會蒐集與您有關的病歷資料、醫療紀錄、量表、問卷等資料與資訊，並以一個編號來代替您的名字及相關個人資料。前述資料若為紙本型式，將會與本同意書分開存放於研究機構之上鎖櫃中；若為電子方式儲存或建檔以供統計與分析之用，將會存放於設有密碼與適當防毒軟體之專屬電腦內。這些研究資料與資訊將會保存至藥品於我國上市後至少兩年，若試驗疫苗終止研發則保存至試驗正式停止後至少二年，至多將保存至疫苗上市後或試驗正式停止後二年。

上述資料與資訊若傳輸至國外分析與統計，您仍會獲得與本國法規相符之保障，計畫主持人與相關團隊將盡力確保您的個人資料獲得妥善保護。

（十）損害補償與保險：

1. 如依本研究所訂臨床試驗計畫，因發生不良反應造成損害，由聯亞生技開發股份有限公司負補償責任。但本受試者同意書上所記載之可預期不良反應，不予補償。
2. 如依本研究所訂臨床試驗計畫，因而發生不良反應或損害，贊助廠商將依法負責損害賠償責任。本醫院願意提供專業醫療照顧及醫療諮詢。您不必負擔治療不良反應或損害之必要醫療費用。
3. 除前二項補償及醫療照顧外，本研究不提供其他形式之補償。若您不願意接受這樣的風險，請勿參加試驗。
4. 您不會因為簽署本同意書，而喪失在法律上的任何權利。
5. 本研究有投保責任保險。

（十一）受試者權利：

1. 試驗過程中，與您的健康或是疾病有關，可能影響您繼續接受臨床試驗意願的任何重大發現，都將即時提供給您。
2. 如果您在試驗過程中對試驗工作性質產生疑問，對身為患者之權利有意見或懷疑因參與研究而受害時，可與本院之研究倫理委員會聯絡請求諮詢，其電話號碼為：04-22052121轉1925、1926。
3. 為進行試驗工作，您必須接受黃高彬醫師的照顧。如果您現在或於試驗期間有任何問題或狀況，請不必客氣，可與在中國醫藥大學附設醫院兒童感染科的黃高彬醫師聯絡（24小時聯繫電話：0975-681-950）。
4. 參加試驗研究計畫之補助：本計畫將在每次訪視提供交通費2000元給您，整個試

驗預計給予您12000元。

5. 本同意書一式2份，醫師已將同意書副本交給您，並已完整說明本研究之性質與目的。醫師已回答您有關藥品與研究的問題。

（十二）試驗之退出與中止：

您可自由決定是否參加本試驗；試驗過程中也可隨時撤銷同意，退出試驗，不需任何理由，且不會引起任何不愉快或影響其日後醫師對您的醫療照顧。

計畫主持人或贊助廠商亦可能於必要時中止該試驗之進行。

（十三）簽名：

1. 計畫主持人、或協同主持人已詳細解釋有關本研究計畫中上述研究方法的性質與目的，及可能產生的危險與利益。

   計畫主持人/協同主持人簽名：____________日期：________年____月____日

2. 受試者已詳細瞭解上述研究方法及其所可能產生的危險與利益，有關本試驗計畫的疑問，業經試驗主持人詳細予以解釋。本人同意接受為臨床試驗計畫的自願受試者。

   受試者簽名：___________________________日期：________年____月____日

   法定代理人簽名：_______________________日期：________年____月____日

   * 受試者為無行為能力(未滿七歲之未成年人者或禁治產人)，由法定代理人為之；禁治產人，由監護人擔任其法定代理人。
   * 受試者為限制行為人者(滿七歲以上之未成年人)，應得法定代理人之同意。

   有同意權人簽名：_______________________日期：________年____月____日

   * 受試者雖非無行為能力或限制行為能力者，但因意識混亂或有精神與智能障礙，而無法進行有效溝通和判斷時，由有同意權之人為之。前項有同意權人為配偶及直系親屬。

3. 見證人

   見證人簽名：____________________________日期：________年____月____日

   身分證字號：　　　　　　　　　　　　聯絡電話：

   通訊地址：

   * 受試者、法定代理人或有同意權之人皆無法閱讀時，應由見證人在場參與所有有關受試者同意之討論。並確定受試者、法定代理人或有同意權之人之同意完全出於其自由意願後，應於受試者同意書簽名並載明日期。試驗相關人員不得為見證人。

中國醫藥大學暨附設醫院
受試者同意書
（成年安全確認組）

流程表:

安全確認組

**訪視**	**1[l]**	**2[l]**		**3**		**4**		**5/提早退出試驗**	**6[t]**		**7**		**長期性追蹤**
**檢測項目**	**篩選**	**第一次接種**		**第二次接種**		**追蹤 [f]**		**個別解盲**	**第三次接種 [g]**		**追蹤 [g]**		**第 12 個月追蹤 [h]**
**天數**	**-28~-1**	**1**	**8, 15, 22**	**29 ±3 天**	**36, 43**	**57 ±3 天**	**64, 71, 78, 85**	**197 ±15 天**	**197~242**	**第六次訪視後 7 天**	**第六次訪視後 14 天 ±3 天**	**253, 309**	**365 ±45 天**
獲得受試者同意書	X								**X [g]**				
納入/排除條件	X	X											
隨機分派		X											
**接種評估**				X					**X [g]**				
基本資料	X												
醫療病史	X	X											
身體檢查 [a]	X	X		X		X		X	**X [g]**		**X [g]**		**X**
生命徵象	X	X		X		X		X	**X [g]**		**X [g]**		
心電圖	X												
**實驗室檢測 (安全性)**													
血液常規檢測 [i]	X					X			**X[g]**		**X[g]**		
血液生化學檢測 [i]	X					X			**X[g]**		**X[g]**		
免疫檢測 [i]	X					X			**X[g]**		**X[g]**		
懷孕檢測 [b]		X		X					**X[g]**				

中國醫藥大學暨附設醫院
受試者同意書
（成年安全確認組）

訪視	1[l]	2[l]		3		4		5/提早退出試驗	6[t]		7		長期性追蹤
**檢測項目**	**篩選**	**第一次接種**		**第二次接種**		**追蹤** [f]		**個別解盲**	**第三次接種** [g]		**追蹤** [g]		**第 12 個月追蹤** [h]
**天數**	**-28~-1**	**1**	**8, 15, 22**	**29 ±3 天**	**36, 43**	**57 ±3 天**	**64, 71, 78, 85**	**197 ±15 天**	**197~242**	**第六次訪視後 7 天**	**第六次訪視後 14 天 ±3 天**	**253, 309**	**365 ±45 天**
尿液常規檢測 [b]	X	X [n]		X [n]		X [n]		X [n]	**X**[g, n]		**X**[g, n]		
**實驗室檢測(探索性試驗)**													
**備血**	**X**					**X**							
**免疫原性** [c]									**X** [g]		**X** [g]		**X** [g]
疫苗接種		X		X					**X** [g]				
指導使用電子日誌卡		X		X					**X** [g]				
電話安全性追蹤 [c]			X		X		X			**X**[p, g]		X	
不良事件/特殊不良事件 [m]/醫療需求不良事件/嚴重不良事件		X [j]	X [j]	X [j]	X [j]	X [j]	X [j]	X [k]	**X** [k, g]	**X** [k, g]	**X** [k ,g]	X [k]	X [k]
新型冠種病毒感染監測		X	X	X	X	X [d]	X [d]	X [d]	**X** [d, g]	**X** [d, g]	**X** [d, g]	X [d]	X [d]
併用藥物		X		X		X		X [e]	**X** [e, g]		**X** [e, g]		

a: 身高與體重僅在第一次訪視測量。
b: 於第 1, 29 天使用尿液懷孕檢測。若尿液檢測為陽性，應以血清懷孕檢測再次確認。以血清懷孕檢測替代尿液檢測將不視為試驗偏差。蛋白尿將透過尿液常規檢測確認，做為基礎值。

c: 所有受試者將進行電話安全追蹤以監測非預期性不良事件，包括特殊不良事件，和監測新型冠狀病毒感染的症狀。
d: 受試者將在每周(為每 7 天)由手機接獲一條提示以規律監測新型冠狀病毒感染症狀或病徵。記錄疑似新冠病毒感染所使用的藥物至新冠病毒感染頁面。
e: 只記錄醫療需求不良事件和嚴重不良事件之併用藥物。
f: 第二次接種疫苗後第 28 天
g: **針對同意並進行施打第三劑試驗疫苗的疫苗組受試者**
h: 第二次接種疫苗後第十二個月(第 365 天)
i: 安全性實驗室數值包括全血液計數(血紅素、血比容、紅血球計數)、白血球計數、血小板計數、肌酸酐、丙胺酸轉胺酶、天門冬胺酸轉胺酶、總膽紅素、直接膽紅素、高靈敏度 C 反應性蛋白、抗核抗體
j: 主動收集時期
k: 被動監測時期
l: 第一次訪視與第二次訪視可為同一次訪視。
m: 包括接種最後一劑疫苗後 12 個月，可能的免疫媒介醫療狀況(PIMMC)或任何定義為可能的特殊不良事件。新冠病毒感染的併發症也將視為疾病增強事件，將被記錄與通報為特殊不良事件。
n: 若受試者有≥第三級以上的高血壓，受試者將或熱是否有蛋白尿存在或惡化。
o: 備血將被冷凍存放做為 UBI 新型冠狀病毒酵素結合免疫吸附分析法，新型冠狀病毒確認酵素結合免疫吸附分析法，以及未來免疫學研究之用。
p: 若符合資格並接種第三劑疫苗的受試者將進行兩週的日誌卡追蹤紀錄，並將於接種後第 7 天進行電話安全性追蹤。
q: 第五次訪視和第六次訪視可為同一天。

中國醫藥大學暨附設醫院
受試者同意書
（成年免疫第三劑組）

您被邀請參與此研究。此同意書主要是提供您本研究之相關資訊，以便您決定是否參加本研究。計畫主持人或其指定之研究人員會為您說明研究內容並回答您的疑問。您可以提出任何和此研究有關的問題，在您的問題尚未獲得滿意的答覆之前，請不要簽署此同意書。如果您願意參與本研究，此文件將視為您的同意紀錄。即使在您同意後，您可以隨時退出本研究不需任何理由。

<table>
<tr><td colspan="2">計畫名稱
中文：一個評估 UB-612 疫苗對於新型冠狀病毒於青少年、成年和老年健康受試者的免疫原性、安全性與耐受性的第二期、安慰劑控制、隨機分派、觀察者盲性臨床試驗
英文：A Phase II, Placebo-controlled, Randomized, Observer-blind Study to Evaluate the Immunogenicity, Safety, and Tolerability of UB-612 Vaccine against COVID-19 in Adolescent, Younger and Elderly Adult Volunteers</td></tr>
<tr><td>執行單位：中國醫藥大學附設醫院感染科、家庭醫學科</td><td>委託單位/藥廠：聯亞生技開發股份有限公司
研究經費來源：聯亞生技開發股份有限公司
受託研究機構：晉加股份有限公司</td></tr>
<tr><td>計畫主持人：黃高彬</td><td>職稱：主治醫師</td></tr>
<tr><td>協同主持人：林文元</td><td>職稱：主治醫師</td></tr>
<tr><td>協同主持人：林伯昌</td><td>職稱：主治醫師</td></tr>
<tr><td>緊急聯絡人：黃高彬</td><td>電話：0975-681-950</td></tr>
<tr><td>受試者姓名：
性別：
身分證字號：
通訊地址：</td><td>病歷號碼：
出生日期：
聯絡電話：</td></tr>
<tr><td>法定代理人或有同意權人之姓名：
性別：
身分證字號：
通訊地址：</td><td>與受試者關係：
出生日期：
聯絡電話：</td></tr>
<tr><td colspan="2">(一)試驗簡介：
1. 本品/技術資料：</td></tr>
</table>

新型冠狀病毒(SARS-CoV-2)於2019年12月起造成中國湖北省武漢市發現多起病毒性肺炎群聚，隨後於2020年1月底台灣出現第一起境外移入確診個案。此疾病在全球擴散，世界衛生組織宣布將此疫情為「國際關注公共衛生緊急事件」。截至2020年底，全球僅有數間疫苗公司，如美國輝瑞藥廠等，取得緊急使用授權上市。

UB-612疫苗為聯亞生技開發股份有限公司所開發新型冠狀病毒預防性疫苗，疫苗含病毒棘狀融合蛋白和胜肽片段，可產生高親和力抗體與新型冠狀病毒結合，並誘發細胞免疫反應，進而達到預防新型冠狀病毒的感染。

UB-612第一期延伸性試驗顯示，接種第三劑UB-612疫苗可以誘發極高的中和抗體，在目前變種病毒的威脅之下，施打第三劑加強免疫反應，已是許多國家的選擇。

2. 本品上市狀況：

本品仍應用於人體試驗，尚未在我國上市。

3. 本試驗使用的UB-612疫苗對新型冠狀病毒的預防效果仍未確認。

4. 您簽署這份受試者同意書是由於您願意接種第三劑UB-612疫苗。

(二)試驗目的：

主要試驗目的

- 評估UB-612疫苗誘發的新型冠狀病毒中和抗體效價。
- 評估接種UB-612疫苗後的安全性和耐受性。

次要試驗目的

- 評估在試驗期間對於新型冠狀病毒的免疫反應。
- 評估三批獨立批次疫苗的批次免疫一致性。

探索性試驗目的

- 評估UB-612疫苗誘發的T細胞功能。
- 評估UB-612疫苗在年輕受試者的安全性和免疫原性。
- 評估UB-612疫苗的療效。
- 描述UB-612疫苗於確診和/或嚴重感染新型冠狀病毒案例之血液學反應。
- **評估針對SARS-CoV-2抗原的抗體反應。**

中國醫藥大學暨附設醫院
受試者同意書
（成年免疫第三劑組）

(三)試驗之主要納入與排除條件：

中國醫藥大學暨附設醫院執行本研究計畫的醫師或相關研究人員將會與您討論有關參加本研究的必要條件。請您配合必須誠實告知我們您過去的健康情形，若您有不符合參加本研究的情況，將不能參加本研究計畫。

1. 參加本研究計畫的主要條件:

☐(1) 您為納入試驗時20~85歲之間健康男性或未懷孕的女性受試者。

☐(2) 您為具生育能力的女性與男性應於首次接種疫苗至最後一次疫苗後3個月同意進行有效的避孕方式。可接受的有效避孕方式包括:

☐a. 男性或女性以手術方法絕育、植入式避孕、或子宮避孕器。

☐b. 注射避孕、避孕藥、避孕貼片、避孕環加上一種屏障避孕法*。

☐c. 合併使用兩種屏障避孕法*。

*有效的屏障避孕法為避孕隔膜、男性或女性保險套、避孕海綿或殺精劑(含可殺精化學物質的藥膏或凝膠)。

☐(3) 您能理解受試者同意書內容的說明與可能的風險，提供簽名的受試者同意書。

☐(4) 您能夠理解與遵從本試驗程序與能夠參與每次訪視。

☐(5) 您的耳溫≤ 38.0°C。

☐(6) 您依據醫療病史、身體檢查和試驗主持人的臨床判斷為健康受試者**可符合納入試驗資格。經試驗主持人判斷，即便您的病史穩定或且控制良好，但伴隨病情惡化而有提高嚴重新型冠狀病毒感染的風險。

**健康受試者有先前存在的穩定疾病者可以納入試驗，定義為該疾病在納入試驗前12週內沒有惡化至需要治療或住院的顯著變化和在納入試驗6個月內沒有惡化至需要治療或住院的顯著變化。

2. 若您有下列任一情況，您將無法參加本研究計畫:

☐(1) 您有接種疫苗後需要醫療介入的過敏性休克、蕁麻疹或其他顯著不良反應的病史。

☐(2) 您在篩選時或接種每劑疫苗前已懷孕女性或懷孕檢測為陽性的女性。

☐(3) 您為正在哺乳的女性，或計畫從接種第一劑疫苗至最後一劑疫苗後60天哺乳的女性。

☐(4) 您在接種第一劑疫苗前3天內，經試驗主持人判斷，患有任何急性疾病。

☐(5) 您在接種第一劑疫苗前1個月內有重大手術。

☐(6) 您是已知為人類免疫缺乏病毒抗體陽性。

☐(7) 您是已知為活動性B型肝炎或C型肝炎。活動性肝炎定義為肝臟轉胺酶(天門冬胺酸轉胺酶和/或丙胺酸轉胺酶)大於3倍正常值上限和/或總膽紅素大於3倍正常值上限。

☐(8) 您是已知曾暴露於新型冠狀病毒，或曾接受預防新型冠狀病毒、中東呼吸症候群冠狀病毒、嚴重急性呼吸道症候群的試驗或已上市產品。

☐(9) 您有格林-巴利症候群的病史。

☐(10) 您在簽署受試者同意書前12周內參與其他的臨床試驗。

☐(11) 您為免疫缺乏/失調疾病，無論是否由基因缺陷、免疫缺乏症或免疫抑制療法所造成。

☐(12) 您計畫或正在進行抗癌症治療。

☐(13) 您患有血小板異常或其他凝血異常可能造成注射之禁忌症。

☐(14) 您在接種第一劑疫苗前6個月長期接受(≥14天連續使用)免疫抑制劑、皮質類固醇(相當於一天使用≥ 20 mg強的松(prednisone))或細胞毒性治療。

☐(15) 您在接種第一劑疫苗前4個月接受免疫球蛋白和/或任何血液製劑的治療。

☐(16) 您在接種試驗疫苗前14天接種任何季流感疫苗或新型流感疫苗，或前28天接種其他疫苗。

☐(17) 您預期在接種試驗疫苗後14天接種任何季流感疫苗或新型流感疫苗，或後28天接種其他疫苗。

☐(18) 您使用短期(<14天使用)全身性類固醇。應於中斷使用全身性類固醇至少28天後才可使用試驗疫苗。吸入/噴霧性、關節注射、囊內或局部(皮膚或眼用)類固醇可允許使用。

☐(19) 您在篩選期前3個月失血或捐血超過500毫升，或預計在試驗期間內捐血或輸血。

☐(20) 經試驗主持人判斷，您有任何醫療疾病或狀況，可能會影響試驗結果或參與試驗可能會對受試者引發額外風險。

☐(21) 您是直接參與本試驗執行的試驗主持人所屬機構**的試驗團隊**、試驗委託者或受託

研究機構(CRO)的員工。

(四)試驗方法及相關檢驗：

這是一個第二期、觀察者盲性、多中心、隨機分派、安慰劑控制試驗，以評估青少年，成年和老年受試者使用兩劑UB-612疫苗的免疫原性，耐受度和安全性。有一部份的受試者使用UB-612疫苗，而另外一部份的受試者則使用「安慰劑」。所謂「安慰劑」是不含有效成份的疫苗。至於誰使用試驗用藥或誰使用「安慰劑」，則像丟銅板或擲骰子一樣由機率決定，不管是您或是研究醫師都不知道您使用了那一種藥，只有分發跟施打疫苗的試驗人員才知道您使用哪一種疫苗，這叫做觀察者盲性。

總計約有3850位合格成年受試者組成核心組用於申請緊急使用授權，另外大約有385位青少年受試者組成補充組申請額外適應症。所有受試者將以6:1的比例，隨機分派至兩劑100微克劑量組別和安慰劑組，包括462位大於18歲至小於65歲可評估的受試者進入批次分析組。對於免疫分析，至少包括350位可評估的成年受試者(年齡大於18歲至小於65歲)和154位可評估的老年受試者(年齡≥65歲)進行描述性分析。免疫原性的受試者將會先納入試驗。所有的受試者將會納入安全性分析，其中至少770位隨機分派的受試者為≥65歲的分層。青少年組將在核心組招募完畢後，再開始納入試驗。約有385位青少年受試者將以6:1的比例隨機分派，其中包括154位可評估的青少年受試者將收集免疫原性數據，並和成年及老年受試者數據進行比較。

您若參與這個試驗，則為有進行免疫分析檢測的免疫原性或批次一致組。

試驗總共有8個訪視。若您參與本試驗，則至少包括第一次訪視(篩選訪視)、第二次訪視（第1天，基礎值，隨機分派，第一次接種疫苗)、第三次訪視(第29天，第二次接種疫苗)、第四次訪視(第57天)、第五次訪視(第197天)。

在第五次訪視時，預計將進行個別解盲。若個別解盲後，得知您為施打疫苗的受試者，且你有意願且符合資格接種第三劑疫苗，將進入第六次訪視(第197~242天，第三次接種疫苗)，第七次訪視(接種疫苗後第14天)，及第八次訪視(第365天)。

整個試驗期間，預期您將參與試驗最長達13個月。

注意事項

1. 如果您同意參加本試驗，研究人員會請您簽署本份受試者同意書，並確認您符合參加本試驗的條件。
2. 從您參與試驗的當天開始，每次訪視都將有合格的試驗人員執行試驗流程與聯繫。

3. 若您有任何符合新型冠狀病毒感染的定義(您曾於過去 7 天內有出國，或是接觸疑似或確認武漢肺炎之病人，而有下列症狀: 發燒、開始咳嗽或惡化、開始呼吸急促或惡化、寒顫、開始肌肉疼痛或惡化、喉嚨痛、腹瀉、嘔吐、開始味覺/嗅覺異常)，請**依照中央疫情指揮中心規定進行自主健康管理或至指定院所進行篩檢。**

4. 若您在試驗期間感染新型冠狀病毒，將依法通報主管機關。

您是否同意? □是 □否

簽名：___________日期：_____________

5. 於試驗期間，您不論任何理由提前退出試驗，試驗研究人員都將安排您完成最後一次的訪視之所有試驗項目。您有權利拒絕此項安排，您的決定不會引起任何影響日後醫師對您的醫療照護。

試驗步驟

第一次訪視(第-28~-1 天) -篩選訪視

在試驗醫師或試驗研究人員為您提供足夠的試驗資訊，並確保您有充分的時間考慮以及詢問任何問題後，您願意讓您參與本試驗，並由您簽署本受試者同意書。在確認您已完成受試者同意書簽署並且您也保有一份副本後，試驗醫師或試驗研究人員將會進行以下試驗程序：

(1) 記錄您簽署受試者同意書的日期
(2) 為您指定一組受試者篩選編號
(3) 確認您是否符合本試驗的納入排除條件
(4) 收集您的個人基本資料（例如生日、年齡及性別）
(5) 記錄您的醫療/用藥病史
(6) 進行身體檢查，包括身高體重
(7) 確認生命徵象
(8) 進行心電圖檢測
(9) 收集尿液檢體進行尿液檢測
(10) 收集血液檢體(共 **15.5** 毫升)，進行下列檢測:

- 常規血液檢測
- 血液生化學檢測
- 免疫學檢測**(抗核抗體)**

- 備血(作為測量新型冠狀病毒血清抗體和相關研究之用)

第二次訪視(第 1 天)-基礎值，確認符合試驗條件，第一次接種疫苗

(1) 再度確認您是否符合本試驗的納入排除條件
(2) 隨機分派，給予您一組隨機分派號碼
(3) 記錄您的醫療/用藥病史
(4) 進行身體檢查
(5) 確認生命徵象
(6) 進行尿液懷孕檢測（具有生育能力女性）
(7) 若您有產生第三級以上的高血壓，您將被收集尿液檢體以檢測是否有蛋白尿存在或惡化
(8) 收集血液檢體(若不進行 T 細胞檢測，共 20 毫升)，進行下列檢測:
   - 免疫原性檢測，包括 Anti-S1-RBD 免疫球蛋白 G 濃度，和新型冠狀病毒中和抗體效價
   - 若您願意，將進行 T 細胞檢測(需額外抽血 56 毫升)

您是否同意? ☐是 ☐否

簽名：＿＿＿＿＿＿日期：＿＿＿＿＿＿＿

(9) 進行第一次疫苗接種(注射部位為非慣用手，採用肌肉注射方式)。接種疫苗後，受試者應留在試驗地點至少 30 分鐘，監測生命徵象和急性過敏症狀。
(10) 將詳細地指導您如何填寫電子日誌卡(包括注射後 7 天內預期性不良事件，14 天內的皮膚過敏反應日誌卡)
(11) 收集併用藥物/治療

第一次電話安全性追蹤(第 8, 15, 22 天)

將與您電話聯繫，以追蹤未預期不良事件和新型冠狀病毒感染症狀。

第三次訪視(第 29± 3 天)-第二次接種疫苗

(1) 進行第二次接種評估(有可能會延遲接種時間)
(2) 進行身體檢查
(3) 確認生命徵象
(4) 進行尿液懷孕檢測（具生育能力的女性）
(5) 若您有產生第三級以上的高血壓，您將被收集尿液檢體以檢測是否有蛋白尿存在或惡化。

(6) 若您為批次一致/免疫原組，將收集血液檢體(共 5 毫升)，進行下列檢測:

- 特定抗原 Anti-S1-RBD 抗體

(7) 進行第二次疫苗接種。

(8) 接種疫苗後，受試者應留在試驗地點至少 30 分鐘，監測生命徵象和急性過敏症狀。

(9) 將詳細地指導您如何填寫電子日誌卡(包括注射後 7 天內預期性不良事件，14 天內的皮膚過敏反應日誌卡)

(10) 收集併用藥物/治療

(11) 記錄上一次訪視至此次訪視之間的不良事件、嚴重不良事件型或新型冠狀病毒感染症狀

第二次電話安全性追蹤(第 36, 43 天)

將與您電話聯繫，以追蹤未預期不良事件和新型冠狀病毒感染症狀。

第四次訪視(第 57±3 天) –追蹤訪視

(1) 進行身體檢查

(2) 確認生命徵象

(3) 若您有產生第三級以上的高血壓，您將被收集尿液檢體以檢測是否有蛋白尿存在或惡化。

(4) 收集血液檢體(若不進行 T 細胞檢測，共 **35.5** 毫升) ，進行下列檢測:

- 免疫**原性**檢測，包括 Anti-S1-RBD 免疫球蛋白 G 濃度、和新型冠狀病毒中和抗體效價
- 若您願意，將進行 T 細胞檢測(需額外抽血 56 毫升)
- 常規血液檢測
- 血液生化學檢測
- 免疫學檢測**(抗核抗體)**
- **備血(作為測量新型冠狀病毒血清抗體和相關研究之用)**

(5) 收集併用藥物/治療

(6) 記錄上一次訪視至此次訪視之間的不良事件、嚴重不良事件或新型冠狀病毒感染症狀

(7) 在此次訪視後，您將每周接獲訊息提醒，以定期監測新型冠狀病毒感染症狀

第三次電話安全性追蹤(第 64, 71, 78, 85 天)

將與您電話聯繫，以追蹤安全性及新型冠狀病毒感染症狀。

第五次訪視(第 197±15 天) –個別解盲

(1) 進行身體檢查
(2) 確認生命徵象
(3) 若您有產生第三級以上的高血壓，您將被收集尿液檢體以檢測是否有蛋白尿存在或惡化。
(4) 將替您個別解盲，告知您接種到疫苗或安慰劑。
(5) 收集血液檢體(共 20 毫升) ，進行下列檢測:
- 免疫**原性**檢測，包括 Anti-S1-RBD 免疫球蛋白 G 濃度、和新型冠狀病毒中和抗體效價

(6) 收集併用藥物/治療
(7) 記錄上一次訪視至此次訪視之間的不良事件、嚴重不良事件或新型冠狀病毒感染症狀

第六次訪視(第 197~242 天，第三次接種疫苗)

(1) 記錄您簽署受試者同意書的日期
(2) 確認您是否符合接種第三劑疫苗的資格，包括第三劑接種疫苗的禁忌症，或有延遲第三劑接種時間的條件
(3) 進行身體檢查
(4) 確認生命徵象
(5) 若您有產生第三級以上的高血壓，您將被收集尿液檢體以檢測是否有蛋白尿存在或惡化
(6) 收集血液檢體(共 20.5 毫升)，進行下列檢測:
- **常規血液檢測**
- **血液生化學檢測**
- **免疫學檢測(抗核抗體)**
- **探索性試驗免疫反應檢測**

(7) 進行尿液懷孕檢測（具有生育能力女性）
(8) 進行第三次疫苗接種。
(9) 接種疫苗後，受試者應留在試驗地點至少 30 分鐘，監測生命徵象和急性過敏症狀。
將詳細地指導您如何填寫電子日誌卡(包括注射後 7 天內預期性不良事件，14 天內的皮膚過敏反應日誌卡)
(10) 收集併用藥物/治療
(11) 進行新型冠狀病毒監測

(12) 記錄上一次訪視至此次訪視之間的不良事件、嚴重不良事件型或新型冠狀病毒感染症狀

第六次訪視後第 7 天電話安全性追蹤

將與您電話聯繫，以追蹤未預期不良事件和新型冠狀病毒感染症狀。此外，還將監測皮膚過敏反應，或其他非預期的過敏反應。若您有發生任何第三級以上的過敏事件，試驗人員可能將安排您額外的回診。

第七次訪視 (第六次訪視後第 14± 3 天)

(1) 進行身體檢查
(2) 確認生命徵象
(3) 若您有產生第三級以上的高血壓，您將被收集尿液檢體以檢測是否有蛋白尿存在或惡化。
(4) 收集血液檢體(共 20.5 毫升)，進行下列檢測:
- 常規血液檢測
- 血液生化學檢測
- 免疫學檢測(抗核抗體)
- 探索性試驗免疫反應檢測

(5) 收集併用藥物/治療
(6) 進行新型冠狀病毒監測
(7) 記錄上一次訪視至此次訪視之間的不良事件、嚴重不良事件型或新型冠狀病毒感染症狀

追蹤期電話安全性追蹤(第 253, 309 天)

第七次訪視後將每兩個月與您電話聯繫，以追蹤安全性及新型冠狀病毒感染症狀。

第八次訪視(第 365±45 天) –第 12 個月追蹤

(1) 確認生命徵象
(2) 收集血液檢體(共 20 毫升) ，進行下列檢測:
- 免疫原性檢測，包括 Anti-S1-RBD 免疫球蛋白 G 濃度、和新型冠狀病毒中和抗體效價

(3) 記錄上一次訪視至此次訪視之間的不良事件，包括特殊不良事件、醫療不良事件、嚴重不良事件或新型冠狀病毒感染症狀

受試者之檢體(含其衍生物)之保存、使用與再利用：

1. 檢體及剩餘檢體之保存與使用

(1) 檢體(含其衍生物)之保存與使用

為研究所需，我們所蒐集您的檢體，將依本研究計畫使用，檢體將保存於**聯亞生技開發股份有限公司(試驗委託者)**，直至 20 年保存期限屆滿，我們將依法銷毀。為了保護您的個人隱私，我們將以一個試驗編號來代替您的名字及相關個人資料，以確認您的檢體及與相關資料受到完整保密。如果您對檢體的使用有疑慮，或您有任何想要銷毀檢體的需求，請立即與我們聯絡(聯絡人：黃高彬醫師電話：0975-681-950)，我們即會將您的檢體銷毀。您也可以聯繫中國醫藥大學暨附設醫院研究倫理委員會(電話：04-22052121 轉 1925、1926)，以協助您解決檢體在研究使用上的任何爭議。

(2) 剩餘檢體(含其衍生物)之再利用

您的生物檢體將會以專屬號碼進行編碼並在**聯亞生技開發股份有限公司(試驗委託者)**的控管下儲存最長20年，以研究UB-612 疫苗反應者的生物標記，及改善治療方式。

所有新的研究計畫都要再經由中國醫藥大學暨附設醫院研究倫理委員會審議通過，倫理審查委員會若認定新的研究超出您同意的範圍，將要求我們重新得到您的同意。

是否同意剩餘檢體保留提供未來新型冠狀病毒感染研究之用，並授權中國醫藥大學暨附設醫院研究倫理委員會審議是否需要再取得您的同意(擇一)

☐ 不同意保存我的剩餘檢體，試驗結束後請銷毀

☐ 同意以非去連結之方式保存我的剩餘檢體，逾越原同意使用範圍時，需再次得到我的同意才可使用我的檢體進行新的研究

2. 檢體及剩餘檢體之部分類型(檢體類型可依計畫書內容自行增減)

(1) 一般生化、血液檢驗/病毒檢測檢體

在試驗期間，會將您的檢體送往聯亞生技開發股份有限公司(試驗委託者)委託的中央實驗室中國醫藥大學暨附設醫院，此機構地址為台中市北區育德路2號，和大安聯合醫事檢驗所，此機構地址為台北市大安區復興南路二段151巷33號，中央實驗室會在分析後立即將分析結果提供給試驗中心，若有剩餘的檢體，**將儲存直到至少完成臨床試驗報告為止，最長將保存20年。**

(2) 抗體/細胞免疫試驗

在試驗期間，會將您的檢體送往聯亞生技開發股份有限公司(試驗委託者)分析實驗室。完成試驗後，若有剩餘檢體，將儲存直到至少完成臨床試驗報告為止，最長將保存20年。

(3) 中和試驗(neutralization test, NT)

在試驗期間，會將您的檢體送往聯亞生技開發股份有限公司(試驗委託者)委託的中央實驗室中央研究院進行處置、處理與進一步分析。此機構地址為台北市南港區研究院路二段128號。完成試驗後，若有剩餘檢體，將儲存直到至少完成臨床試驗報告為止，最長將保存20年。

(4) 遺傳學檢體

在試驗期間，若發生嚴重不良反應或特定不良反應，您的檢體將用於HLA分型檢驗，會將您的檢體送往聯亞生技開發股份有限公司(試驗委託者)委託的中央實驗室有勁基因股份有限公司分析，此機構地址為新北市樹林區復興路376-5號，中央實驗室不會將分析結果提供給試驗中心，若有剩餘的檢體，將會儲存直到檢驗結果複驗完畢即銷毀，不會長期儲存。

(5) 探索性試驗檢體

在試驗期間，會將您的檢體送往聯亞生技開發股份有限公司(試驗委託者)委託的實驗室(表一)進行處理或進一步分析。完成試驗後，若有剩餘檢體，將儲存直到至少完成臨床試驗報告為止，最長將保存20年。

表一、實驗室名稱與機構地址

實驗室名稱	機構地址
聯亞生技開發(股)公司	新竹縣竹北市生醫路二段 6-1 號 5 樓
Viroclinics	Rotterdam Science Tower, Marconistraat 16, 3029 AK Rotterdam, The Netherlands(荷蘭)
DASA	Jonas Cruz de Araujo, Diagnostics da America S/A, Surubiju Avenue, 1890, Barueri, SP, Brazil(巴西), 06455-040
PHE Porton Down	Salisbury Wiltshire SP4 0JG, England(英國)
UTMB	University of Texas Medical Branch 301 University Boulevard Keiller Building, Room 2.150 Galveston, Texas, USA(美國

**Virology**	**University of São Paulo, Brazil Rua Dr EnnEn de Carvalho Aguiar 470, CEP 05403-000 (巴西)**
**VRDL**	**California Department of Public Health, 850 Marina Bay Parkway, Richmond, CA 94804, USA(美國)**
**NEXELIS**	**525 Boul. Cartier Ouest Laval, Qulbec, Canada, H7V 3S8(加拿大)**
**Vaccinology and Immunology Infection, Immunity & Inflammation Dept UCL GOS Institute of Childe Health**	**UCL Great Ormond Street Institute of Child Health 30 Guilford Street London WC1N 1EH, England(英國)**
**VisMederi**	**VisMederi Srl, Strada del Petriccio e Belriguardo, 35, 53100 Siena, Italy(義大利)**

(五)可能產生之副作用、發生率及處理方法：

1. 與試驗藥物相關的風險（本試驗疫苗的副作用）：

冠狀病毒疫苗的開發

過去針對與SARS-CoV-2病毒相同屬於人類冠狀病毒的SARS-CoV(嚴重急性呼吸綜合症冠狀病毒(SARS冠狀病毒))的疫苗研究發現，接種過SARS-CoV疫苗的小鼠在暴露到SARS-CoV後會發生過度免疫反應而產生病變，因此不得不停止這種疫苗的開發。所以，成功的人類冠狀病毒疫苗不只要產生可以抑制病毒的免疫反應，更要避免過度免疫產生的副作用。

疫苗相關的風險: 第一期臨床試驗

接種疫苗可能會出現注射部位的不良反應(例如疼痛、硬化腫脹、皮疹發紅、過敏反應、蜂窩性組織炎)，或全身性不良反應(例如發燒、腹瀉、疲倦、噁心/嘔吐、厭食、咽喉痛、頭痛、咳嗽、關節痛、非注射部位疼痛、非注射部位搔癢、皮膚和黏膜異常、急性過敏反應、昏厥、急性支氣管痙攣、呼吸困難)。

第一期臨床試驗已經有60位受試者接種兩劑疫苗(含10微克、30微克、100微克融合蛋白)，安全實驗室數值並沒有顯示有任何的臨床顯著不正常數值，也沒有發生任何第三級以上與疫苗相關的預期性不良事件。大部分的預期性不良事件都是輕微的，大約於2天之內症狀都會緩解。也沒有任何的嚴重不良事件或特殊不良事件被通報。

疾病增強(disease enhancement) 的風險

SARS-CoV-2候選疫苗也可能會有引發疾病增強(disease enhancement) 的風險，包括抗體依賴性增強(antibody-dependent enhancement)或疫苗相關聯的增強的呼吸道疾病(vaccine-associated enhanced respiratory disease)。在先前研發SARS疫苗時，在數個SARS-CoV動物

攻毒試驗(包括鼠類、雪貂、猴類)當中，有發現疾病增強的現象。疾病增強反應的免疫病理現象包括TH2偏向及嗜酸性白血球的肺部浸潤。但是目前已發表的新型冠狀病毒肺炎疫苗研究，仍尚未發現類似的疾病增強現象。

本試驗疫苗於數個藥理試驗呈現不一致的TH1/TH2 (輔助型T細胞1/輔助型T細胞2)免疫反應偏向，試驗結果並未一致偏向TH2，而由小鼠之SARS-CoV-2動物攻毒試驗結果顯示，本試驗疫苗誘發疾病增強之風險不高。依據文獻指出，組成複雜或容易引起非中和抗體之抗原，如不活化病毒或整片段之蛋白(包含S蛋白與N蛋白)，與易引起偏向Th2免疫反應之佐劑成分，如鋁製佐劑，皆較有可能引起疾病增強。本試驗疫苗的主要抗原為S蛋白上之RBD 區域，已有多篇文獻指出，針對S-RBD設計之SARS與MERS疫苗從未於試驗動物模型上引發疾病增強現象。本試驗疫苗雖使用易引起偏向Th2免疫反應之佐劑，但由動物實驗證實，也同時引起偏向Th1之反應，因此發生疾病增強應屬低風險。且已於多種動物模型中證實，能誘發高效價之中和抗體，於細胞培養中亦能有效抑制新冠病毒感染。

建議您在有效疫苗上市前或本試驗疫苗的產品資訊有進一步更新前，盡量避免暴露於可能感染病毒的環境。研究團隊將會在試驗中執行相關安全性監測。若有任何關於本試驗疫苗與疾病增強風險相關之任何最新資訊，將即時更新並提供給您。

疫苗佐劑相關的風險

本試驗疫苗所使用的佐劑含Adju-Phos®，是屬於一種磷酸鋁類的佐劑。磷酸鋁類佐劑已經使用超過半個世紀，具有相當的安全性。由於此類佐劑可誘導免疫反應，因此可能會造成局部發炎反應，例如在注射部位產生輕微而短暫的疼痛、發紅以及腫脹。

2. 與試驗/研究過程相關的風險：

抽血

本試驗需要抽血檢驗。抽血可能引起一些不適和瘀血。整個試驗期間13個月，共需抽血**157毫升**。若您願意抽血檢驗T細胞檢測，則會再額外抽血共112毫升。若您罹患新型冠狀病毒感染，**可能將於每次額外訪視抽血30毫升。**

在接種疫苗過程中，可能會出現一些尚未在已完成試驗中發現的副作用。一般而言，接種某一新疫苗總是會有一定的風險，但是計畫主持人會採取一切措施預防風險的發生。**計畫主持人鼓勵您報告您遇到的任何不適。**

（六）其他替代療法及說明：

您不是非參加不可，若不參加研究，由於目前尚未有疫苗可用來預防新型冠狀病毒感染，因此預防措施與其他呼吸道感染相同，包括：勤洗手、減少觸摸眼口鼻、注意咳嗽禮節、

妥善處理口鼻分泌物等，避免出入公共場所，並不要接觸野生動物。

如果您對於本試驗疫苗有任何的疑問，您可以提出來向您的試驗醫師討論。

(七)試驗預期效益：

依據臨床前試驗結果，預期本試驗疫苗對您可能可以產生抗體，預防新型冠狀病毒感染，但因每個人體質不同也有可能不會產生療效，故參加本試驗可能不會有直接的好處。

但是您參加本試驗，可協助我們獲得更多資訊，以瞭解UB-612疫苗的安全性與免疫力。

(八)試驗進行中受試者之禁忌、限制與應配合之事項：

禁止使用的藥物

以下藥物請勿在試驗期間使用：

- 直到試驗第197天禁止使用免疫抑制劑、或細胞毒性治療
- 到試驗第197天禁止使用免疫球蛋白和/或任何血液製劑
- 整個試驗期間禁止使用試驗產品(包括藥物或疫苗)
- 到試驗第197天禁止使用全身性皮質類固醇(相當於一天使用≥ 20 mg強的松(prednisone))
- 接種試驗疫苗後14天禁止接種任何季流感疫苗或新型流感疫苗，或後28天禁止接種其他非試驗疫苗。整個試驗期間禁止使用任何已上市的新型冠狀病毒疫苗產品。

允許使用的藥物

若您的藥物或治療必須常規使用，經試驗醫師判斷不會影響本試驗疫苗的免疫原性、臨床療效與安全性，則可以正常使用。您有任何關於在試驗期間可允許使用何種藥物或治療的問題，請詢問您的試驗醫師。

懷孕或母乳哺乳的風險

目前未知本試驗疫苗對於未出生胎兒的影響，因此：

- 您為具生育能力的女性受試者 (除非手術絕育或停經)，或您為男性受試者應於接種疫苗至最後一次疫苗後 3 個月同意進行有效的避孕方式，同意進行有效的避孕方式(例如子宮內節育器、荷爾蒙療法或避孕套)。

- 若您為具生育能力的女性，將請您進行懷孕檢測，結果必須為陰性，方可參與試驗。
- 若您為懷孕的女性，將被告知不可參與本試驗。
- 若您在試驗期間懷孕，請盡速通知試驗人員，並且停止施打本疫苗。
- 基於安全性考量，若您為女性受試者而在試驗期間懷孕，或您為男性受試者而您的性伴侶在試驗期間懷孕(將請您的懷孕性伴侶需簽署另外一份同意書)，您與您的胎兒將會被追蹤監測至分娩，除非另有醫學指示。

您應向您的配偶或性伴侶告知您有參與此試驗與相關風險:

簽名：__________日期：__________________

（九)機密性：

中國醫藥大學附設醫院將依法把任何可辨識您的身分之紀錄與您的個人隱私資料視為機密來處理，不會公開。研究人員將以一個研究代碼代表您的身分，此代碼不會顯示您的姓名、國民身分證統一編號、住址等可識別資料。如果發表試驗/研究結果，您的身分仍將保密。您亦瞭解若簽署同意書即同意您的原始醫療紀錄可直接受監測者、稽核者、研究倫理委員會及主管機關檢閱，以確保臨床試驗/研究過程與數據符合相關法律及法規要求，上述人員並承諾絕不違反您的身分之機密性。除了上述機構依法有權檢視外，我們會小心維護您的隱私。由於試驗藥物可能同時申請美國臨床試驗，依美國藥品管理規定，試驗結果將公佈於公開的臨床試驗資訊網站：Clinicaltrials.gov (美國)，但您的個人資料仍將保密，該網站只會有試驗之結果摘要，您可以在任何時候搜尋該網站。
在試驗/研究期間，依據計畫類型與您所授權的內容，我們將會蒐集與您有關的病歷資料、醫療紀錄、量表、問卷等資料與資訊，並以一個編號來代替您的名字及相關個人資料。前述資料若為紙本型式，將會與本同意書分開存放於研究機構之上鎖櫃中；若為電子方式儲存或建檔以供統計與分析之用，將會存放於設有密碼與適當防毒軟體之專屬電腦內。這些研究資料與資訊將會保存至藥品於我國上市後至少兩年，若試驗疫苗終止研發則保存至試驗正式停止後至少二年，至多將保存至疫苗上市後或試驗正式停止後二年。
上述資料與資訊若傳輸至國外分析與統計，您仍會獲得與本國法規相符之保障，計畫主持人與相關團隊將盡力確保您的個人資料獲得妥善保護。

(十)損害補償與保險:

1. 如依本研究所訂臨床試驗計畫，因發生不良反應造成損害，由聯亞生技開發股份有限公司負補償責任。但本受試者同意書上所記載之可預期不良反應，不予補償。
2. 如依本研究所訂臨床試驗計畫，因而發生不良反應或損害，贊助廠商將依法負責損害賠償責任。本醫院願意提供專業醫療照顧及醫療諮詢。您不必負擔治療不良反應或損害之必要醫療費用。
3. 除前二項補償及醫療照顧外，本研究不提供其他形式之補償。若您不願意接受這樣的風險，請勿參加試驗。
4. 您不會因為簽署本同意書，而喪失在法律上的任何權利。
5. 本研究有投保責任保險。

(十一)受試者權利:

1. 試驗過程中，與您的健康或是疾病有關，可能影響您繼續接受臨床試驗意願的任何重大發現，都將即時提供給您。
2. 如果您在試驗過程中對試驗工作性質產生疑問，對身為患者之權利有意見或懷疑因參與研究而受害時，可與本院之研究倫理委員會聯絡請求諮詢，其電話號碼為:04-22052121轉1925、1926。
3. 為進行試驗工作，您必須接受黃高彬醫師的照顧。如果您現在或於試驗期間有任何問題或狀況，請不必客氣，可與在中國醫藥大學附設醫院兒童感染科的黃高彬醫師聯絡(24小時聯繫電話:0975-681-950)。
4. 參加試驗研究計畫之補助:**本計畫將在每次訪視提供交通費及營養費給您，新增的兩個診次(第六次返診、第七次返診)將各提供費用1500元;整個試驗預計給予您21000元。若您願意參與T細胞檢測研究，將在該次訪視另外提供營養費500元給您。**
5. 本同意書一式2份，醫師已將同意書副本交給您，並已完整說明本研究之性質與目的。醫師已回答您有關藥品與研究的問題。

(十二)試驗之退出與中止:

您可自由決定是否參加本試驗;試驗過程中也可隨時撤銷同意，退出試驗，不需任何理由，且不會引起任何不愉快或影響其日後醫師對您的醫療照顧。

計畫主持人或贊助廠商亦可能於必要時中止該試驗之進行。

(十三)簽名:

1. 計畫主持人、或協同主持人已詳細解釋有關本研究計畫中上述研究方法的性質

與目的，及可能產生的危險與利益。

計畫主持人/協同主持人簽名：____________日期：________年____月____日

2. 受試者已詳細瞭解上述研究方法及其所可能產生的危險與利益，有關本試驗計畫的疑問，業經試驗主持人詳細予以解釋。本人同意接受為臨床試驗計畫的自願受試者。

受試者簽名：___________________________日期：________年____月____日

法定代理人簽名：_______________________日期：________年____月____日

* 受試者為無行為能力(未滿七歲之未成年人者或禁治產人)，由法定代理人為之；禁治產人，由監護人擔任其法定代理人。

* 受試者為限制行為人者(滿七歲以上之未成年人)，應得法定代理人之同意。

有同意權人簽名：_______________________日期：________年____月____日

* 受試者雖非無行為能力或限制行為能力者，但因意識混亂或有精神與智能障礙，而無法進行有效溝通和判斷時，由有同意權之人為之。前項有同意權人為配偶及直系親屬。

3. 見證人

見證人簽名：___________________________日期：________年____月____日

身分證字號： 聯絡電話：

通訊地址：

* 受試者、法定代理人或有同意權之人皆無法閱讀時，應由見證人在場參與所有有關受試者同意之討論。並確定受試者、法定代理人或有同意權之人之同意完全出於其自由意願後，應於受試者同意書簽名並載明日期。試驗相關人員不得為見證人。

中國醫藥大學暨附設醫院
受試者同意書
（成年免疫第三劑組）

流程表:

批次分析與免疫分析組

訪視	1[m]	2[m]		3		4		5/提早退出試驗	6[t]		7		長期性追蹤
**檢測項目**	**篩選**	**第一次接種**		**第二次接種**		**追蹤[g]**		**個別解盲**	**第三次接種[h]**		**追蹤[h]**		**第12個月追蹤[i]**
**天數**	**-28~-1**	**1**	**8, 15, 22**	**29 ±3天**	**36, 43**	**57 ±3天**	**64, 71, 78,85**	**197 ±15天**	**197~242**	**第六次訪視後7天**	**第六次訪視後14天 ±3天**	**253, 309**	**365 ±45天**
獲得受試者同意書	X								**X[h]**				
納入/排除條件	X	X											
隨機分派		X											
**接種評估**				X					**X[h]**				
基本資料	X												
醫療病史	X	X											
身體檢查[a]	X	X		X		X		X	**X[h]**		**X[h]**		
生命徵象	X	X		X		X		X	**X[h]**		**X[h]**		**X**
心電圖	X												
**實驗室檢測(安全性)**													
血液常規檢測[j]	X					X			**X[h]**		**X[h]**		
血液生化學檢測[j]	X					X			**X[h]**		**X[h]**		
免疫檢測[j]	X					X			**X[h]**		**X[h]**		
懷孕檢測[b]		X		X					**X[h]**				

訪視	1[m]	2[m]		3		4		5/提早退出試驗	6[t]		7		長期性追蹤
檢測項目	篩選	第一次接種		第二次接種		追蹤[g]		個別解盲	第三次接種[h]		追蹤[h]		第12個月追蹤[i]
天數	-28~-1	1	8, 15, 22	29 ±3天	36, 43	57 ±3天	64, 71, 78,85	197 ±15天	197~242	第六次訪視後7天	第六次訪視後14天 ±3天	253, 309	365 ±45天
尿液常規檢測[b]	X	X[q]		X[q]		X[q]		X[q]	**X**[h, q]		**X**[h, q]		
**實驗室檢測(免疫原性)**													
免疫原性[c]		X		X[n]		X		X					X
T細胞反應(可選擇[o])		X				X							
**實驗室檢測(探索性試驗)**													
**T細胞功能性檢測(可選擇[u])**									**X**[h]		**X**[h]		
**備血**	X					X							
**免疫原性[c]**									**X**[h]		**X**[h]		
疫苗接種		X		X					**X**[h]				
指導使用電子日誌卡		X		X					**X**[h]				
電話安全性追蹤[d]			X		X		X			**X**[s,h]		X	
不良事件/特殊不良事件[p]/醫		X[k]	X[k]	X[k]	X[k]	X[k]	X[k]	X[l]	**X**[l, h]	**X**[l, h]	**X**[l, h]	X[l]	X[l]

中國醫藥大學暨附設醫院

受試者同意書

(成年免疫第三劑組)

訪視	1[m]	2[m]		3		4		5/提早退出試驗	6[t]		7		長期性追蹤
檢測項目	篩選	第一次接種		第二次接種		追蹤[g]		個別解盲	第三次接種[h]		追蹤[h]		第12個月追蹤[i]
天數	-28~-1	1	8, 15, 22	29 ±3天	36, 43	57 ±3天	64, 71, 78,85	197 ±15天	197~242	第六次訪視後7天	第六次訪視後14天 ±3天	253, 309	365 ±45天
療需求不良事件/嚴重不良事件													
新型冠種病毒感染監測		X	X	X	X	X[e]	X[e]	X[e]	**X**[e, h]	**X**[e, h]	**X**[e, h]	X[e]	X[e]
併用藥物		X		X		X		X[f]	**X**[f, h]		**X**[f, h]		

a: 身高與體重僅在第一次訪視測量。
b:於第 1, 29 天使用尿液懷孕檢測。若尿液檢測為陽性，應以血清懷孕檢測再次確認。以血清懷孕檢測替代尿液檢測將不視為試驗偏差。蛋白尿將透過尿液常規檢測確認，做為基礎值。
c: 針對 Anti-S1-RBD 免疫球蛋白 G 濃度和新型冠狀病毒中和抗體效價，以及抑制 S1-RBD: ACE2 的抗體效價。
d: 所有受試者將進行電話安全追蹤以監測非預期性不良事件，包括特殊不良事件，和監測新型冠狀病毒感染的症狀。
e: 受試者將在每周(為每 7 天)由手機接獲一條提示以規律監測新型冠狀病毒感染症狀或病徵。記錄疑似新冠病毒感染所使用的藥物至新冠病毒感染頁面。
f: 只記錄醫療需求不良事件和嚴重不良事件之併用藥物。
g: 第二次接種疫苗後第 28 天
h: 僅針對同意並進行施打第三劑試驗疫苗的疫苗組受試者
i: 第二次接種疫苗後第十二個月(第 365 天)
j: 安全性實驗室數值包括全血液計數(血紅素、血比容、紅血球計數)、白血球計數、血小板計數、肌酸酐、丙胺酸轉胺酶、天門冬胺酸轉胺酶、總膽紅素、直接膽紅素、高靈敏度 C 反應性蛋白、抗核抗體
k: 主動收集時期
l: 被動監測時期
m: 第一次訪視與第二次訪視可為同一次訪視。
n: 僅測量 Anti-S1-RBD IgG 濃度
o: 將在選定的試驗地點納入至少 100 位>18-<65 歲的受試者。
p: 包括接種最後一劑疫苗後 12 個月，可能的免疫媒介醫療狀況(PIMMC)或任何定義為可能的特殊不良事件。新冠病毒感染的併發症也將視為疾病增強事件，將被記錄與通報為特殊不良事件。
q: 若受試者有≥第三級以上的高血壓，受試者將確認是否有蛋白尿存在或惡化。
r: 備血將被冷凍存放做為 UBI 新型冠狀病毒酵素結合免疫吸附分析法，新型冠狀病毒確認酵素結合免疫吸附分析法，以及未來免疫學研究之用。
s: 若符合資格並接種第三劑疫苗的受試者將進行兩週的日誌卡追蹤紀錄，並將於接種後第 7 天進行電話安全性追蹤。

t: 第五次訪視和第六次訪視可為同一天。

u: 在選定之試驗地點中，將邀請大約 30 位年齡 >18-<65 歲的受試者和大約 30 位年齡 ≥65 歲的受試者。在適用的情況下，優先邀請曾進行過第 57 天 T 細胞功能評估的受試者。

中國醫藥大學暨附設醫院
受試者同意書
（成年安全確認第三劑組）

您被邀請參與此研究。此同意書主要是提供您本研究之相關資訊，以便您決定是否參加本研究。計畫主持人或其指定之研究人員會為您說明研究內容並回答您的疑問。您可以提出任何和此研究有關的問題，在您的問題尚未獲得滿意的答覆之前，請不要簽署此同意書。如果您願意參與本研究，此文件將視為您的同意紀錄。即使在您同意後，您可以隨時退出本研究不需任何理由。

<table>
<tr><td colspan="2">計畫名稱
中文：一個評估 UB-612 疫苗對於新型冠狀病毒於青少年、成年和老年健康受試者的免疫原性、安全性與耐受性的第二期、安慰劑控制、隨機分派、觀察者盲性臨床試驗
英文：A Phase II, Placebo-controlled, Randomized, Observer-blind Study to Evaluate the Immunogenicity, Safety and Tolerability of UB-612 Vaccine against COVID-19 in Adolescent, Younger and Elderly Adult Volunteers</td></tr>
<tr><td>執行單位：中國醫藥大學附設醫院感染科、家庭醫學科</td><td>委託單位/藥廠：聯亞生技開發股份有限公司
研究經費來源：聯亞生技開發股份有限公司
受託研究機構：晉加股份有限公司</td></tr>
<tr><td>計畫主持人：黃高彬</td><td>職稱：主治醫師</td></tr>
<tr><td>協同主持人：林文元</td><td>職稱：主治醫師</td></tr>
<tr><td>協同主持人：林伯昌</td><td>職稱：主治醫師</td></tr>
<tr><td>緊急聯絡人：黃高彬</td><td>電話：0975-681-950</td></tr>
<tr><td>受試者姓名：
性別：
身分證字號：
通訊地址：</td><td>病歷號碼：
出生日期：
聯絡電話：</td></tr>
<tr><td>法定代理人或有同意權人之姓名：
性別：
身分證字號：
通訊地址：</td><td>與受試者關係：
出生日期：
聯絡電話：</td></tr>
<tr><td colspan="2">(一)試驗簡介：
1. 本品/技術資料：</td></tr>
</table>

中國醫藥大學暨附設醫院
受試者同意書
（成年安全確認第三劑組）

新型冠狀病毒(SARS-CoV-2)於2019年12月起造成中國湖北省武漢市發現多起病毒性肺炎群聚，隨後於2020年1月底台灣出現第一起境外移入確診個案。此疾病在全球擴散，世界衛生組織宣布將此疫情為「國際關注公共衛生緊急事件」。截至2020年底，全球僅有數間疫苗公司，如美國輝瑞藥廠等，取得緊急使用授權上市。

UB-612疫苗為聯亞生技開發股份有限公司所開發新型冠狀病毒預防性疫苗，疫苗含病毒棘狀融合蛋白和胜肽片段，可產生高親和力抗體與新型冠狀病毒結合，並誘發細胞免疫反應，進而達到預防新型冠狀病毒的感染。

UB-612第一期延伸性試驗顯示，接種第三劑UB-612疫苗可以誘發極高的中和抗體，在目前變種病毒的威脅之下，施打第三劑加強免疫反應，已是許多國家的選擇。

2. 本品上市狀況：

本品仍應用於人體試驗，尚未在我國上市。

3. 本試驗使用的UB-612疫苗對新型冠狀病毒的預防效果仍未確認。

4. **您簽署這份受試者同意書是由於您願意接種第三劑UB-612疫苗。**

（二）試驗目的：

主要試驗目的

- 評估UB-612疫苗誘發的新型冠狀病毒中和抗體效價。
- 評估接種UB-612疫苗後的安全性和耐受性。

次要試驗目的

- 評估在試驗期間對於新型冠狀病毒的免疫反應。
- 評估三批獨立批次疫苗的批次免疫一致性。

探索性試驗目的

- 評估UB-612疫苗誘發的T細胞功能。
- 評估UB-612疫苗在年輕受試者的安全性和免疫原性。
- 評估UB-612疫苗的療效。
- 描述UB-612疫苗於確診和/或嚴重感染新型冠狀病毒案例之血液學反應。
- **評估針對SARS-CoV-2抗原的抗體反應。**

中國醫藥大學暨附設醫院
受試者同意書
（成年安全確認第三劑組）

（三)試驗之主要納入與排除條件：

中國醫藥大學暨附設醫院執行本研究計畫的醫師或相關研究人員將會與您討論有關參加本研究的必要條件。請您配合必須誠實告知我們您過去的健康情形，若您有不符合參加本研究的情況，將不能參加本研究計畫。

1. 參加本研究計畫的主要條件:

☐(1) 您為納入試驗時20~85歲之間健康男性或未懷孕的女性受試者。

☐(2) 您為具生育能力的女性與男性應於首次接種疫苗至最後一次疫苗後3個月同意進行有效的避孕方式。可接受的有效避孕方式包括:

☐a. 男性或女性以手術方法絕育、植入式避孕、或子宮避孕器。

☐b.注射避孕、避孕藥、避孕貼片、避孕環加上一種屏障避孕法*。

☐c.合併使用兩種屏障避孕法*。

*有效的屏障避孕法為避孕隔膜、男性或女性保險套、避孕海綿或殺精劑(含可殺精化學物質的藥膏或凝膠)。

☐(3) 您能理解受試者同意書內容的說明與可能的風險，提供簽名的受試者同意書。

☐(4) 您能夠理解與遵從本試驗程序與能夠參與每次訪視。

☐(5) 您的耳溫≤ 38.0°C。

☐(6) 您依據醫療病史、身體檢查和試驗主持人的臨床判斷為健康受試者**可符合納入試驗資格。經試驗主持人判斷，即便您的病史穩定或且控制良好，但伴隨病情惡化而有提高嚴重新型冠狀病毒感染的風險。

**健康受試者有先前存在的穩定疾病者可以納入試驗，定義為該疾病在納入試驗前12週內沒有惡化至需要治療或住院的顯著變化和在納入試驗6個月內沒有惡化至需要治療或住院的顯著變化。

2. 若您有下列任一情況，您將無法參加本研究計畫:

☐(1) 您有接種疫苗後需要醫療介入的過敏性休克、蕁麻疹或其他顯著不良反應的病史。

☐(2) 您在篩選時或接種每劑疫苗前已懷孕女性或懷孕檢測為陽性的女性。

☐(3) 您為正在哺乳的女性，或計畫從接種第一劑疫苗至最後一劑疫苗後60天哺乳的女性。

☐(4) 您在接種第一劑疫苗前3天內，經試驗主持人判斷，患有任何急性疾病。

☐(5) 您在接種第一劑疫苗前1個月內有重大手術。

☐(6) 您是已知為人類免疫缺乏病毒抗體陽性。

☐(7) 您是已知為活動性B型肝炎或C型肝炎。活動性肝炎定義為肝臟轉胺酶(天門冬胺酸轉胺酶和/或丙胺酸轉胺酶)大於3倍正常值上限和/或總膽紅素大於3倍正常值上限。

☐(8) 您是已知曾暴露於新型冠狀病毒，或曾接受預防新型冠狀病毒、中東呼吸症候群冠狀病毒、嚴重急性呼吸道症候群的試驗或已上市產品。

☐(9) 您有格林-巴利症候群的病史。

☐(10) 您在簽署受試者同意書前12周內參與其他的臨床試驗。

☐(11) 您為免疫缺乏/失調疾病，無論是否由基因缺陷、免疫缺乏症或免疫抑制療法所造成。

☐(12) 您計畫或正在進行抗癌症治療。

☐(13) 您患有血小板異常或其他凝血異常可能造成注射之禁忌症。

☐(14) 您在接種第一劑疫苗前6個月長期接受(≥14天連續使用)免疫抑制劑、皮質類固醇(相當於一天使用≥ 20 mg強的松(prednisone))或細胞毒性治療。

☐(15) 您在接種第一劑疫苗前4個月接受免疫球蛋白和/或任何血液製劑的治療。

☐(16) 您在接種試驗疫苗前14天接種任何季流感疫苗或新型流感疫苗，或前28天接種其他疫苗。

☐(17) 您預期在接種試驗疫苗後14天接種任何季流感疫苗或新型流感疫苗，或後28天接種其他疫苗。

☐(18) 您使用短期(<14天使用)全身性類固醇。應於中斷使用全身性類固醇至少28天後才可使用試驗疫苗。吸入/噴霧性、關節注射、囊內或局部(皮膚或眼用)類固醇可允許使用。

☐(19) 您在篩選期前3個月失血或捐血超過500毫升，或預計在試驗期間內捐血或輸血。

☐(20) 經試驗主持人判斷，您有任何醫療疾病或狀況，可能會影響試驗結果或參與試驗可能會對受試者引發額外風險。

☐(21) 您是直接參與本試驗執行的試驗主持人所屬機構**的試驗團隊**、試驗委託者或受託

研究機構(CRO)的員工。

（四）試驗方法及相關檢驗：

這是一個第二期、觀察者盲性、多中心、隨機分派、安慰劑控制試驗，以評估青少年，成年和老年受試者使用兩劑UB-612疫苗的免疫原性，耐受度和安全性。有一部份的受試者使用UB-612疫苗，而另外一部份的受試者則使用「安慰劑」。所謂「安慰劑」是不含有效成份的疫苗。至於誰使用試驗用藥或誰使用「安慰劑」，則像丟銅板或擲骰子一樣由機率決定，不管是您或是研究醫師都不知道您使用了那一種藥，只有分發跟施打疫苗的試驗人員才知道您使用哪一種疫苗，這叫做觀察者盲性。

總計約有3850位合格成年受試者組成核心組用於申請緊急使用授權，另外大約有385位青少年受試者組成補充組申請額外適應症。所有受試者將以6:1的比例，隨機分派至兩劑100微克劑量組別和安慰劑組，包括462位大於18歲至小於65歲可評估的受試者進入批次分析組。對於免疫分析，至少包括350位可評估的成年受試者(年齡大於18歲至小於65歲)和154位可評估的老年受試者(年齡≥65歲)進行描述性分析。免疫原性的受試者將會先納入試驗。所有的受試者將會納入安全性分析，其中至少770位隨機分派的受試者為≥65歲的分層。青少年組將在核心組招募完畢後，再開始納入試驗。約有385位青少年受試者將以6:1的比例隨機分派，其中包括154位可評估的青少年受試者將收集免疫原性數據，並和成年及老年受試者數據進行比較。

您若參與這個試驗，則為進行安全性確認的安全確認組。

試驗總共有8個訪視。若您參與本試驗，則至少包括第一次訪視(篩選訪視)、第二次訪視（第1天，基礎值，隨機分派，第一次接種疫苗)、第三次訪視(第29天，第二次接種疫苗)、第四次訪視(第57天)、第五次訪視(第197天)。

在第五次訪視時，預計將進行個別解盲。若個別解盲後，得知您為施打疫苗的受試者，且你有意願且符合資格接種第三劑疫苗，將進入第六次訪視(第197~242天，第三次接種疫苗)，第七次訪視(接種疫苗後第14天)，及第八次訪視(第365天)。

整個試驗期間，預期您將參與試驗最長達13個月。

注意事項

1. 如果您同意參加本試驗，研究人員會請您簽署本份受試者同意書，並確認您符合參加本試驗的條件。
2. 從您參與試驗的當天開始，每次訪視都將有合格的試驗人員執行試驗流程與聯繫。

中國醫藥大學暨附設醫院

受試者同意書

（成年安全確認第三劑組）

3. 若您有任何符合新型冠狀病毒感染的定義(您曾於過去 7 天內有出國，或是接觸疑似或確認武漢肺炎之病人，而有下列症狀: 發燒、開始咳嗽或惡化、開始呼吸急促或惡化、寒顫、開始肌肉疼痛或惡化、喉嚨痛、腹瀉、嘔吐、開始味覺/嗅覺異常)，請**依照中央疫情指揮中心規定進行自主健康管理或至指定院所進行篩檢。**

4. 若您在試驗期間感染新型冠狀病毒，將依法通報主管機關。

   您是否同意? □是 □否

   簽名：__________日期：__________

5. 於試驗期間，您不論任何理由提前退出試驗，試驗研究人員都將安排您完成最後一次的訪視之所有試驗項目。您有權利拒絕此項安排，您的決定不會引起任何影響日後醫師對您的醫療照護。

試驗步驟

第一次訪視(第-28~-1 天) -篩選訪視

在試驗醫師或試驗研究人員為您提供足夠的試驗資訊，並確保您有充分的時間考慮以及詢問任何問題後，您願意讓您參與本試驗，並由您簽署本受試者同意書。在確認您已完成受試者同意書簽署並且您也保有一份副本後，試驗醫師或試驗研究人員將會進行以下試驗程序：

(1) 記錄您簽署受試者同意書的日期
(2) 為您指定一組受試者篩選編號
(3) 確認您是否符合本試驗的納入排除條件
(4) 收集您的個人基本資料（例如生日、年齡及性別）
(5) 記錄您的醫療/用藥病史
(6) 進行身體檢查，包括身高體重
(7) 確認生命徵象
(8) 進行心電圖檢測
(9) 收集尿液檢體進行尿液檢測
(10) 收集血液檢體(共 **15.5** 毫升)，進行下列檢測:

- 常規血液檢測
- 血液生化學檢測
- 免疫學檢測**(抗核抗體)**

- 備血(作為測量新型冠狀病毒血清抗體和相關研究之用)

第二次訪視(第 1 天)-基礎值，確認符合試驗條件，第一次接種疫苗

(1) 再度確認您是否符合本試驗的納入排除條件
(2) 隨機分派，給予您一組隨機分派號碼
(3) 記錄您的醫療/用藥病史
(4) 進行身體檢查
(5) 確認生命徵象
(6) 進行尿液懷孕檢測（具有生育能力女性）
(7) 若您有產生第三級以上的高血壓，您將被收集尿液檢體以檢測是否有蛋白尿存在或惡化
(8) 進行第一次疫苗接種(注射部位為非慣用手，採用肌肉注射方式)。接種疫苗後，受試者應留在試驗地點至少 30 分鐘，監測生命徵象和急性過敏症狀。
(9) 將詳細地指導您如何填寫電子日誌卡(包括注射後 7 天內預期性不良事件，14 天內的皮膚過敏反應日誌卡)
(10) 收集併用藥物/治療

第一次電話安全性追蹤(第 8, 15, 22 天)

將與您電話聯繫，以追蹤未預期不良事件和新型冠狀病毒感染症狀。

第三次訪視(第 29± 3 天)-第二次接種疫苗

(1) 進行第二次接種評估(有可能會延遲接種時間)
(2) 進行身體檢查
(3) 確認生命徵象
(4) 進行尿液懷孕檢測（具生育能力的女性）
(5) 若您有產生第三級以上的高血壓，您將被收集尿液檢體以檢測是否有蛋白尿存在或惡化。
(6) 進行第二次疫苗接種。
(7) 接種疫苗後，受試者應留在試驗地點至少 30 分鐘，監測生命徵象和急性過敏症狀。
(8) 將詳細地指導您如何填寫電子日誌卡(包括注射後 7 天內預期性不良事件，14 天內的皮膚過敏反應日誌卡)
(9) 收集併用藥物/治療
(10) 記錄上一次訪視至此次訪視之間的不良事件、嚴重不良事件型或新型冠狀病毒感染症狀

第二次電話安全性追蹤(第 36, 43 天)

將與您電話聯繫，以追蹤未預期不良事件和新型冠狀病毒感染症狀。

第四次訪視(第 57±3 天) –追蹤訪視

(1) 進行身體檢查
(2) 確認生命徵象
(3) 若您有產生第三級以上的高血壓，您將被收集尿液檢體以檢測是否有蛋白尿存在或惡化。
(4) 收集血液檢體(共 **15.5** 毫升) ，進行下列檢測:
- 常規血液檢測
- 血液生化學檢測
- 免疫學檢測**(抗核抗體)**
- **備血(作為測量新型冠狀病毒血清抗體和相關研究之用)**

(5) 收集併用藥物/治療
(6) 記錄上一次訪視至此次訪視之間的不良事件、嚴重不良事件或新型冠狀病毒感染症狀
(7) 在此次訪視後，您將每周接獲訊息提醒，以定期監測新型冠狀病毒感染症狀至第 197 天。

第三次電話安全性追蹤(第 64, 71, 78, 85 天)

將與您電話聯繫，以追蹤安全性及新型冠狀病毒感染症狀。

第五次訪視(第 197±15 天) –個別解盲

(1) 進行身體檢查
(2) 確認生命徵象
(3) 若您有產生第三級以上的高血壓，您將被收集尿液檢體以檢測是否有蛋白尿存在或惡化。
(4) 將替您個別解盲，告知您接種到疫苗或安慰劑。
(5) 收集併用藥物/治療
(6) 記錄上一次訪視至此次訪視之間的不良事件、嚴重不良事件或新型冠狀病毒感染症狀

第六次訪視(第 197~242 天，第三次接種疫苗)

(1) 記錄您簽署受試者同意書的日期
(2) 確認您是否符合接種第三劑疫苗的資格，包括第三劑接種疫苗的禁忌症，或有延

遲第三劑接種時間的條件

(3) 進行身體檢查

(4) 確認生命徵象

(5) 若您有產生第三級以上的高血壓，您將被收集尿液檢體以檢測是否有蛋白尿存在或惡化

(6) 收集血液檢體(共 20.5 毫升)，進行下列檢測:

- 常規血液檢測
- 血液生化學檢測
- 免疫學檢測(抗核抗體)
- 探索性試驗免疫反應檢測

(7) 進行尿液懷孕檢測（具有生育能力女性）

(8) 進行第三次疫苗接種。

(9) 接種疫苗後，受試者應留在試驗地點至少 30 分鐘，監測生命徵象和急性過敏症狀。

將詳細地指導您如何填寫電子日誌卡(包括注射後 7 天內預期性不良事件，14 天內的皮膚過敏反應日誌卡)

(10) 收集併用藥物/治療

(11) 進行新型冠狀病毒監測

(12) 記錄上一次訪視至此次訪視之間的不良事件、嚴重不良事件型或新型冠狀病毒感染症狀

<u>第六次訪視後第 7 天電話安全性追蹤</u>

將與您電話聯繫，以追蹤未預期不良事件和新型冠狀病毒感染症狀。此外，還將監測皮膚過敏反應，或其他非預期的過敏反應。若您有發生任何第三級以上的過敏事件，試驗人員可能將安排您額外的回診。

<u>第七次訪視 (第六次訪視後第 14± 3 天)</u>

(1) 進行身體檢查

(2) 確認生命徵象

(3) 若您有產生第三級以上的高血壓，您將被收集尿液檢體以檢測是否有蛋白尿存在或惡化。

(4) 收集血液檢體(共 20.5 毫升)，進行下列檢測:

- 常規血液檢測
- 血液生化學檢測

- **免疫學檢測(抗核抗體)**
- **探索性試驗免疫反應檢測**

(5) **收集併用藥物/治療**

(6) **進行新型冠狀病毒監測**

(7) **記錄上一次訪視至此次訪視之間的不良事件、嚴重不良事件型或新型冠狀病毒感染症狀**

追蹤期電話安全性追蹤(第 253, 309 天)

第七次訪視後將每兩個月與您電話聯繫，以追蹤安全性及新型冠狀病毒感染症狀。

第八次訪視(第 365±45 天) –第 12 個月追蹤

(1) 確認生命徵象

(2) **收集血液檢體(共 10 毫升)，進行下列檢測:**

- **探索性試驗免疫反應檢測**

(3) 記錄上一次訪視至此次訪視之間的不良事件，包括特殊不良事件、醫療不良事件、嚴重不良事件或新型冠狀病毒感染症狀

受試者之檢體(含其衍生物)之保存、使用與再利用：

1. 檢體及剩餘檢體之保存與使用

(1) 檢體(含其衍生物)之保存與使用

為研究所需，我們所蒐集您的檢體，將依本研究計畫使用，檢體將保存於**聯亞生技開發股份有限公司(試驗委託者)**，直至 20 年保存期限屆滿，我們將依法銷毀。為了保護您的個人隱私，我們將以一個試驗編號來代替您的名字及相關個人資料，以確認您的檢體及與相關資料受到完整保密。如果您對檢體的使用有疑慮，或您有任何想要銷毀檢體的需求，請立即與我們聯絡(聯絡人：黃高彬醫師電話：0975-681-950)，我們即會將您的檢體銷毀。您也可以聯繫中國醫藥大學暨附設醫院研究倫理委員會(電話：04-22052121 轉 1925、1926)，以協助您解決檢體在研究使用上的任何爭議。

(2) 剩餘檢體(含其衍生物)之再利用

您的生物檢體將會以專屬號碼進行編碼並在**聯亞生技開發股份有限公司(試驗委託者)**的控管下儲存最長20年，以研究UB-612 疫苗反應者的生物標記，及改善治療方式。

所有新的研究計畫都要再經由中國醫藥大學暨附設醫院研究倫理委員會審議通過，

倫理審查委員會若認定新的研究超出您同意的範圍，將要求我們重新得到您的同意。

是否同意剩餘檢體保留提供未來新型冠狀病毒感染研究之用，並授權中國醫藥大學暨附設醫院研究倫理委員會審議是否需要再取得您的同意(擇一)

☐ 不同意保存我的剩餘檢體，試驗結束後請銷毀

☐ 同意以非去連結之方式保存我的剩餘檢體，逾越原同意使用範圍時，需再次得到我的同意才可使用我的檢體進行新的研究

2. 檢體及剩餘檢體之部分類型(檢體類型可依計畫書內容自行增減)

(1) 一般生化、血液檢驗/病毒檢測檢體

在試驗期間，會將您的檢體送往聯亞生技開發股份有限公司(試驗委託者)委託的中央實驗室中國醫藥大學暨附設醫院，此機構地址為台中市北區育德路2號，和大安聯合醫事檢驗所，此機構地址為台北市大安區復興南路二段151巷33號，中央實驗室會在分析後立即將分析結果提供給試驗中心，若有剩餘的檢體，**將儲存直到至少完成臨床試驗報告為止，最長將保存20年。**

(2) 抗體/細胞免疫試驗

在試驗期間，會將您的檢體送往聯亞生技開發股份有限公司(試驗委託者)分析實驗室。完成試驗後，若有剩餘檢體，將儲存直到至少完成臨床試驗報告為止，最長將保存20年。

(3) 中和試驗(neutralization test, NT)

在試驗期間，會將您的檢體送往聯亞生技開發股份有限公司(試驗委託者)委託的中央實驗室中央研究院進行處置、處理與進一步分析。此機構地址為台北市南港區研究院路二段 128 號。完成試驗後，若有剩餘檢體，將儲存直到至少完成臨床試驗報告為止，最長將保存 20 年。

(4) 遺傳學檢體

在試驗期間，若發生嚴重不良反應或特定不良反應，您的檢體將用於 HLA 分型檢驗，會將您的檢體送往聯亞生技開發股份有限公司(試驗委託者)委託的中央實驗室有勁基因股份有限公司分析，此機構地址為新北市樹林區復興路 376-5 號，中央實驗室不會將分析結果提供給試驗中心，若有剩餘的檢體，將會儲存直到檢驗結果複驗完畢即銷毀，

不會長期儲存。

(5) 探索性試驗檢體

在試驗期間，會將您的檢體送往聯亞生技開發股份有限公司(試驗委託者)委託的相關實驗室(表一)進行處理或進一步分析。完成試驗後，若有剩餘檢體，將儲存直到至少完成臨床試驗報告為止，最長將保存 20 年。

表一、實驗室名稱與機構地址

實驗室名稱	機構地址
聯亞生技開發(股)公司	新竹縣竹北市生醫路二段 6-1 號 5 樓
Viroclinics	Rotterdam Science Tower, Marconistraat 16, 3029 AK Rotterdam, The Netherlands(荷蘭)
DASA	Jonas Cruz de Araujo, Diagnostics da America S/A, Surubiju Avenue, 1890, Barueri, SP, Brazil(巴西), 06455-040
PHE Porton Down	Salisbury Wiltshire SP4 0JG, England(英國)
UTMB	University of Texas Medical Branch 301 University Boulevard Keiller Building, Room 2.150 Galveston, Texas, USA(美國
Virology	University of São Paulo, Brazil Rua Dr EnnEn de Carvalho Aguiar 470, CEP 05403-000 (巴西)
VRDL	California Department of Public Health, 850 Marina Bay Parkway, Richmond, CA 94804, USA(美國)
NEXELIS	525 Boul. Cartier Ouest Laval, Qulbec, Canada, H7V 3S8(加拿大)
Vaccinology and Immunology Infection, Immunity & Inflammation Dept UCL GOS Institute of Childe Health	UCL Great Ormond Street Institute of Child Health 30 Guilford Street London WC1N 1EH, England(英國)
VisMederi	VisMederi Srl, Strada del Petriccio e Belriguardo, 35, 53100 Siena, Italy(義大利)

（五)可能產生之副作用、發生率及處理方法：

1. 與試驗藥物相關的風險（本試驗疫苗的副作用）：

<u>冠狀病毒疫苗的開發</u>

過去針對與SARS-CoV-2病毒相同屬於人類冠狀病毒的SARS-CoV(嚴重急性呼吸綜合症冠狀病毒(SARS冠狀病毒))的疫苗研究發現，接種過SARS-CoV疫苗的小鼠在暴露到SARS-CoV後會發生過度免疫反應而產生病變，因此不得不停止這種疫苗的開發。所以，

成功的人類冠狀病毒疫苗不只要產生可以抑制病毒的免疫反應，更要避免過度免疫產生的副作用。

疫苗相關的風險: 第一期臨床試驗

接種疫苗可能會出現注射部位的不良反應(例如疼痛、硬化腫脹、皮疹發紅、過敏反應、蜂窩性組織炎)，或全身性不良反應(例如發燒、腹瀉、疲倦、噁心/嘔吐、厭食、咽喉痛、頭痛、咳嗽、關節痛、非注射部位疼痛、非注射部位搔癢、皮膚和黏膜異常、急性過敏反應、昏厥、急性支氣管痙攣、呼吸困難)。

第一期臨床試驗已經有60位受試者接種兩劑疫苗(含10微克、30微克、100微克融合蛋白)，安全實驗室數值並沒有顯示有任何的臨床顯著不正常數值，也沒有發生任何第三級以上與疫苗相關的預期性不良事件。大部分的預期性不良事件都是輕微的，症狀在大約於2天之內都會緩解。試驗中也沒有任何的嚴重不良事件或特殊不良事件被通報。

疾病增強(disease enhancement) 的風險

SARS-CoV-2候選疫苗也可能會有引發疾病增強(disease enhancement) 的風險，包括抗體依賴性增強(antibody-dependent enhancement)或疫苗相關聯的增強的呼吸道疾病(vaccine-associated enhanced respiratory disease)。在先前研發SARS疫苗時，在數個SARS-CoV動物攻毒試驗(包括鼠類、雪貂、猴類)當中，有發現疾病增強的現象。疾病增強反應的免疫病理現象包括TH2偏向及嗜酸性白血球的肺部浸潤。但是目前已發表的新型冠狀病毒肺炎疫苗研究，仍尚未發現類似的疾病增強現象。

本試驗疫苗於數個藥理試驗呈現不一致的TH1/TH2 (輔助型T細胞1/輔助型T細胞2)免疫反應偏向，試驗結果並未一致偏向TH2，而由小鼠之SARS-CoV-2動物攻毒試驗結果顯示，本試驗疫苗誘發疾病增強之風險不高。依據文獻指出，組成複雜或容易引起非中和抗體之抗原，如不活化病毒或整片段之蛋白(包含S蛋白與N蛋白)，與易引起偏向Th2免疫反應之佐劑成分，如鋁製佐劑，皆較有可能引起疾病增強。本試驗疫苗的主要抗原為S蛋白上之RBD 區域，已有多篇文獻指出，針對S-RBD設計之SARS與MERS疫苗從未於試驗動物模型上引發疾病增強現象。本試驗疫苗雖使用易引起偏向Th2免疫反應之佐劑，但由動物實驗證實，也同時引起偏向Th1之反應，因此發生疾病增強應屬低風險。且已於多種動物模型中證實，能誘發高效價之中和抗體，於細胞培養中亦能有效抑制新冠病毒感染。

建議您在有效疫苗上市前或本試驗疫苗的產品資訊有進一步更新前，盡量避免暴露於可能感染病毒的環境。研究團隊將會在試驗中執行相關安全性監測。若有任何關於本試驗疫苗與疾病增強風險相關之任何最新資訊，將即時更新並提供給您。

疫苗佐劑相關的風險

本試驗疫苗所使用的佐劑含Adju-Phos®，是屬於一種磷酸鋁類的佐劑。磷酸鋁類佐劑已

經使用超過半個世紀，具有相當的安全性。由於此類佐劑可誘導免疫反應，因此可能會造成局部發炎反應，例如在注射部位產生輕微而短暫的疼痛、發紅以及腫脹。

2. 與試驗/研究過程相關的風險：

抽血

本試驗需要抽血檢驗。抽血可能引起一些不適和瘀血。整個試驗期間13個月，共需抽血**82毫升**。若您罹患新型冠狀病毒感染，**可能將於每次額外訪視抽血30毫升。**

在接種疫苗過程中，可能會出現一些尚未在已完成試驗中發現的副作用。一般而言，接種某一新疫苗總是會有一定的風險，但是計畫主持人會採取一切措施預防風險的發生。**計畫主持人鼓勵您報告您遇到的任何不適。**

(六)其他替代療法及說明：

您不是非參加不可，若不參加研究，由於目前尚未有疫苗可用來預防新型冠狀病毒感染，因此預防措施與其他呼吸道感染相同，包括：勤洗手、減少觸摸眼口鼻、注意咳嗽禮節、妥善處理口鼻分泌物等，避免出入公共場所，並不要接觸野生動物。

如果您對於本試驗疫苗有任何的疑問，您可以提出來向您的試驗醫師討論。

(七)試驗預期效益：

依據臨床前試驗結果，預期本試驗疫苗對您可能可以產生抗體，預防新型冠狀病毒感染，但因每個人體質不同也有可能不會產生療效，故參加本試驗可能不會有直接的好處。

但是您參加本試驗，可協助我們獲得更多資訊，以瞭解UB-612疫苗的安全性與免疫力。

(八)試驗進行中受試者之禁忌、限制與應配合之事項：

禁止使用的藥物

以下藥物請勿在試驗期間使用：

- 直到試驗第57天禁止使用免疫抑制劑、或細胞毒性治療
- 到試驗第57天禁止使用免疫球蛋白和/或任何血液製劑
- 整個試驗期間禁止使用試驗產品(包括藥物或疫苗)
- 到試驗第57天禁止使用全身性皮質類固醇(相當於一天使用≥ 20 mg強的松(prednisone))

- 接種試驗疫苗後14天禁止接種任何季流感疫苗或新型流感疫苗，或後28天禁止接種其他非試驗疫苗。整個試驗期間禁止使用任何已上市的新型冠狀病毒疫苗產品。

允許使用的藥物

若您的藥物或治療必須常規使用，經試驗醫師判斷不會影響本試驗疫苗的安全性，則可以正常使用。您有任何關於在試驗期間可允許使用何種藥物或治療的問題，請詢問您的試驗醫師。

懷孕或母乳哺乳的風險

目前未知本試驗疫苗對於未出生胎兒的影響，因此：

- 您為具生育能力的女性受試者 (除非手術絕育或停經)，或您為男性受試者應於接種疫苗至最後一次疫苗後 3 個月同意進行有效的避孕方式，同意進行有效的避孕方式(例如子宮內節育器、荷爾蒙療法或避孕套)。
- 若您為具生育能力的女性，將請您進行懷孕檢測，結果必須為陰性，方可參與試驗。
- 若您為懷孕的女性，將被告知不可參與本試驗。
- 若您在試驗期間懷孕，請盡速通知試驗人員，並且停止施打本疫苗。
- 基於安全性考量，若您為女性受試者而在試驗期間懷孕，或您為男性受試者而您的性伴侶在試驗期間懷孕(將請您的懷孕性伴侶需簽署另外一份同意書)，您與您的胎兒將會被追蹤監測至分娩，除非另有醫學指示。

您應向您的配偶或性伴侶告知您有參與此試驗與相關風險:

簽名：__________ 日期：____________________

（九)機密性：

中國醫藥大學附設醫院將依法把任何可辨識您的身分之紀錄與您的個人隱私資料視為機密來處理，不會公開。研究人員將以一個研究代碼代表您的身分，此代碼不會顯示您的姓名、國民身分證統一編號、住址等可識別資料。如果發表試驗/研究結果，您的身分仍將保密。您亦瞭解若簽署同意書即同意您的原始醫療紀錄可直接受監測者、稽核者、研究倫理委員會及主管機關檢閱，以確保臨床試驗/研究過程與數據符合相關法律及法規要求，上述人員並承諾絕不違反您的身分之機密性。除了上述機構依法有權檢視外，我們

會小心維護您的隱私。由於試驗藥物可能同時申請美國臨床試驗，依美國藥品管理規定，試驗結果將公佈於公開的臨床試驗資訊網站：Clinicaltrials.gov (美國)，但您的個人資料仍將保密，該網站只會有試驗之結果摘要，您可以在任何時候搜尋該網站。

在試驗/研究期間，依據計畫類型與您所授權的內容，我們將會蒐集與您有關的病歷資料、醫療紀錄、量表、問卷等資料與資訊，並以一個編號來代替您的名字及相關個人資料。前述資料若為紙本型式，將會與本同意書分開存放於研究機構之上鎖櫃中；若為電子方式儲存或建檔以供統計與分析之用，將會存放於設有密碼與適當防毒軟體之專屬電腦內。這些研究資料與資訊將會保存至藥品於我國上市後至少兩年，若試驗疫苗終止研發則保存至試驗正式停止後至少二年，至多將保存至疫苗上市後或試驗正式停止後二年。

上述資料與資訊若傳輸至國外分析與統計，您仍會獲得與本國法規相符之保障，計畫主持人與相關團隊將盡力確保您的個人資料獲得妥善保護。

（十）損害補償與保險：

1. 如依本研究所訂臨床試驗計畫，因發生不良反應造成損害，由聯亞生技開發股份有限公司負補償責任。但本受試者同意書上所記載之可預期不良反應，不予補償。
2. 如依本研究所訂臨床試驗計畫，因而發生不良反應或損害，贊助廠商將依法負責損害賠償責任。本醫院願意提供專業醫療照顧及醫療諮詢。您不必負擔治療不良反應或損害之必要醫療費用。
3. 除前二項補償及醫療照顧外，本研究不提供其他形式之補償。若您不願意接受這樣的風險，請勿參加試驗。
4. 您不會因為簽署本同意書，而喪失在法律上的任何權利。
5. 本研究有投保責任保險。

（十一）受試者權利：

1. 試驗過程中，與您的健康或是疾病有關，可能影響您繼續接受臨床試驗意願的任何重大發現，都將即時提供給您。
2. 如果您在試驗過程中對試驗工作性質產生疑問，對身為患者之權利有意見或懷疑因參與研究而受害時，可與本院之研究倫理委員會聯絡請求諮詢，其電話號碼為：04-22052121轉1925、1926。
3. 為進行試驗工作，您必須接受黃高彬醫師的照顧。如果您現在或於試驗期間有任何問題或狀況，請不必客氣，可與在中國醫藥大學附設醫院兒童感染科的黃高彬醫師聯絡（24小時聯繫電話：0975-681-950）。
4. 參加試驗研究計畫之補助：**本計畫將在每次訪視提供交通費及營養費給您，新增的兩個診次(第六次返診、第七次返診)將各提供費用1000元；整個試驗預計給予**

中國醫藥大學暨附設醫院
受試者同意書
（成年安全確認第三劑組）

您14000元。

5. 本同意書一式2份，醫師已將同意書副本交給您，並已完整說明本研究之性質與目的。醫師已回答您有關藥品與研究的問題。

（十二）試驗之退出與中止：

您可自由決定是否參加本試驗；試驗過程中也可隨時撤銷同意，退出試驗，不需任何理由，且不會引起任何不愉快或影響其日後醫師對您的醫療照顧。

計畫主持人或贊助廠商亦可能於必要時中止該試驗之進行。

（十三）簽名：

1. 計畫主持人、或協同主持人已詳細解釋有關本研究計畫中上述研究方法的性質與目的，及可能產生的危險與利益。

   計畫主持人/協同主持人簽名：____________日期：________年____月____日

2. 受試者已詳細瞭解上述研究方法及其所可能產生的危險與利益，有關本試驗計畫的疑問，業經試驗主持人詳細予以解釋。本人同意接受為臨床試驗計畫的自願受試者。

   受試者簽名：____________________________日期：________年____月____日

   法定代理人簽名：________________________日期：________年____月____日

   * 受試者為無行為能力(未滿七歲之未成年人者或禁治產人)，由法定代理人為之；禁治產人，由監護人擔任其法定代理人。

   * 受試者為限制行為人者(滿七歲以上之未成年人)，應得法定代理人之同意。

   有同意權人簽名：________________________日期：________年____月____日

   * 受試者雖非無行為能力或限制行為能力者，但因意識混亂或有精神與智能障礙，而無法進行有效溝通和判斷時，由有同意權之人為之。前項有同意權人為配偶及直系親屬。

3. 見證人

   見證人簽名：____________________________日期：________年____月____日

   身分證字號：　　　　　　　　　　聯絡電話：

   通訊地址：

   * 受試者、法定代理人或有同意權之人皆無法閱讀時，應由見證人在場參與所有有關受試者同意之討論。並確定受試者、法定代理人或有同意權之人之同意完全出於其自由意願後，應於受試者同意書簽名並載明日期。試驗相關人員不得為見證人。

中國醫藥大學暨附設醫院
受試者同意書
（成年安全確認第三劑組）

流程表:

安全確認組

**訪視**	**1**[l]	**2**[l]		**3**		**4**		**5/提早退出試驗**	**6**[t]		**7**		**長期性追蹤**
**檢測項目**	**篩選**	**第一次接種**		**第二次接種**		**追蹤**[f]		**個別解盲**	**第三次接種**[g]		**追蹤**[g]		**第 12 個月追蹤**[h]
**天數**	**-28~-1**	**1**	**8, 15, 22**	**29 ±3 天**	**36, 43**	**57 ±3 天**	**64, 71, 78 ,85**	**197 ±15 天**	**197~242**	**第六次訪視後 7 天**	**第六次訪視後 14 天 ±3 天**	**253, 309**	**365 ±45 天**
獲得受試者同意書	X								**X**[g]				
納入/排除條件	X	X											
隨機分派		X											
**接種評估**				X					**X**[g]				
基本資料	X												
醫療病史	X	X											
身體檢查[a]	X	X		X		X		X	**X**[g]		**X**[g]		**X**
生命徵象	X	X		X		X		X	**X**[g]		**X**[g]		
心電圖	X												
**實驗室檢測 (安全性)**													
血液常規檢測[i]	X					X			**X**[g]		**X**[g]		
血液生化學檢測[i]	X					X			**X**[g]		**X**[g]		
免疫檢測[i]	X					X			**X**[g]		**X**[g]		

中國醫藥大學暨附設醫院

受試者同意書

（成年安全確認第三劑組）

訪視	1[1]	2[1]		3		4		5/提早退出試驗	6[t]		7		長期性追蹤
檢測項目	篩選	第一次接種		第二次接種		追蹤[f]		個別解盲	第三次接種[g]		追蹤[g]		第 12 個月追蹤[h]
天數	-28~-1	1	8, 15, 22	29 ±3 天	36, 43	57 ±3 天	64, 71, 78 ,85	197 ±15 天	197~242	第六次訪視後 7 天	第六次訪視後 14 天 ±3 天	253, 309	365 ±45 天
懷孕檢測[b]		X		X					X[g]				
尿液常規檢測[b]	X	X[n]		X[n]		X[n]		X[n]	X[g, n]		X[g, n]		
**實驗室檢測(探索性試驗)**									**X**		**X**		X
**備血**	**X**					**X**							
**免疫原性**[c]									**X**[g]		**X**[g]		**X**[g]
疫苗接種		X		X					**X**[g]				
指導使用電子日誌卡		X		X					**X**[g]				
電話安全性追蹤[c]			X		X		X			**X**[p, g]		X	
不良事件/特殊不良事件[m]/醫療需求不良事件/嚴重不良事件		X[j]	X[j]	X[j]	X[j]	X[j]	X[j]	X[k]	**X**[k, g]	**X**[k, g]	**X**[k ,g]	X[k]	X[k]
新型冠種病毒感染監測		X	X	X	X	X[d]	X[d]	X[d]	**X**[d, g]	**X**[d, g]	**X**[d, g]	X[d]	X[d]
併用藥物		X		X		X		X[e]	**X**[e, g]		**X**[e, g]		

a: 身高與體重僅在第一次訪視測量。
b: 於第 1, 29 天使用尿液懷孕檢測。若尿液檢測為陽性，應以血清懷孕檢測再次確認。以血清懷孕檢測替代尿液檢測將不視為試驗偏差。蛋白尿將透過尿液常規檢測確認，做為基礎值。
c: 所有受試者將進行電話安全追蹤以監測非預期性不良事件，包括特殊不良事件，和監測新型冠狀病毒感染的症狀。
d: 受試者將在每周(為每 7 天)由手機接獲一條提示以規律監測新型冠狀病毒感染症狀或病徵。記錄疑似新冠病毒感染所使用的藥物至新冠病毒感染頁面。
e: 只記錄醫療需求不良事件和嚴重不良事件之併用藥物。
f: 第二次接種疫苗後第 28 天
g: **針對同意並進行施打第三劑試驗疫苗的疫苗組受試者**
h: 第二次接種疫苗後第十二個月(第 365 天)
i: 安全性實驗室數值包括全血液計數(血紅素、血比容、紅血球計數)、白血球計數、血小板計數、肌酸酐、丙胺酸轉胺酶、天門冬胺酸轉胺酶、總膽紅素、直接膽紅素、高靈敏度 C 反應性蛋白、抗核抗體
j: 主動收集時期
k: 被動監測時期
l: 第一次訪視與第二次訪視可為同一次訪視。
m: 包括接種最後一劑疫苗後 12 個月，可能的免疫媒介醫療狀況(PIMMC)或任何定義為可能的特殊不良事件。新冠病毒感染的併發症也將視為疾病增強事件，將被記錄與通報為特殊不良事件。
n: 若受試者有≥第三級以上的高血壓，受試者將或熱是否有蛋白尿存在或惡化。
o: 備血將被冷凍存放做為 UBI 新型冠狀病毒酵素結合免疫吸附分析法，新型冠狀病毒確認酵素結合免疫吸附分析法，以及未來免疫學研究之用。
p: 若符合資格並接種第三劑疫苗的受試者將進行兩週的日誌卡追蹤紀錄，並將於接種後第 7 天進行電話安全性追蹤。
q: 第五次訪視和第六次訪視可為同一天。

中國醫藥大學暨附設醫院

受試者同意書

（成年免疫第三劑組）

黃高彬
2021.10.25

您被邀請參與此研究。此同意書主要是提供您本研究之相關資訊，以便您決定是否參加本研究。計畫主持人或其指定之研究人員會為您說明研究內容並回答您的疑問。您可以提出任何和此研究有關的問題，在您的問題尚未獲得滿意的答覆之前，請不要簽署此同意書。如果您願意參與本研究，此文件將視為您的同意紀錄。即使在您同意後，您可以隨時退出本研究不需任何理由。

計畫名稱<br>中文：一個評估 UB-612 疫苗對於新型冠狀病毒於青少年、成年和老年健康受試者的免疫原性、安全性與耐受性的第二期、安慰劑控制、隨機分派、觀察者盲性臨床試驗<br>英文：A Phase II, Placebo-controlled, Randomized, Observer-blind Study to Evaluate the Immunogenicity, Safety, and Tolerability of UB-612 Vaccine against COVID-19 in Adolescent, Younger and Elderly Adult Volunteers	
執行單位：中國醫藥大學附設醫院感染科、家庭醫學科	委託單位/藥廠：聯亞生技開發股份有限公司<br>研究經費來源：聯亞生技開發股份有限公司<br>受託研究機構：晉加股份有限公司
計畫主持人：黃高彬	職稱：主治醫師
協同主持人：林文元	職稱：主治醫師
協同主持人：林伯昌	職稱：主治醫師
緊急聯絡人：黃高彬	電話：0975-681-950
受試者姓名：<br>性別：<br>身分證字號：<br>通訊地址：	病歷號碼：<br>出生日期：<br>聯絡電話：
法定代理人或有同意權人之姓名：<br>性別：<br>身分證字號：<br>通訊地址：	與受試者關係：<br>出生日期：<br>聯絡電話：
（一）試驗簡介：<br>1. 本品/技術資料：	

新型冠狀病毒(SARS-CoV-2)於2019年12月起造成中國湖北省武漢市發現多起病毒性肺炎群聚，隨後於2020年1月底台灣出現第一起境外移入確診個案。此疾病在全球擴散，世界衛生組織宣布將此疫情為「國際關注公共衛生緊急事件」。截至2020年底，全球僅有數間疫苗公司，如美國輝瑞藥廠等，取得緊急使用授權上市。

UB-612疫苗為聯亞生技開發股份有限公司所開發新型冠狀病毒預防性疫苗，疫苗含病毒棘狀融合蛋白和胜肽片段，可產生高親和力抗體與新型冠狀病毒結合，並誘發細胞免疫反應，進而達到預防新型冠狀病毒的感染。

UB-612第一期延伸性試驗顯示，接種第三劑UB-612疫苗可以誘發極高的中和抗體，在目前變種病毒的威脅之下，施打第三劑加強免疫反應，已是許多國家的選擇。

2. 本品上市狀況：

本品仍應用於人體試驗，尚未在我國上市。

3. 本試驗使用的UB-612疫苗對新型冠狀病毒的預防效果仍未確認。

4. 您簽署這份受試者同意書是由於您願意接種第三劑UB-612疫苗。

(二)試驗目的：

<u>主要試驗目的</u>

- 評估UB-612疫苗誘發的新型冠狀病毒中和抗體效價。
- 評估接種UB-612疫苗後的安全性和耐受性。

<u>次要試驗目的</u>

- 評估在試驗期間對於新型冠狀病毒的免疫反應。
- 評估三批獨立批次疫苗的批次免疫一致性。

<u>探索性試驗目的</u>

- 評估UB-612疫苗誘發的T細胞功能。
- 評估UB-612疫苗在年輕受試者的安全性和免疫原性。
- 評估UB-612疫苗的療效。
- 描述UB-612疫苗於確診和/或嚴重感染新型冠狀病毒案例之血液學反應。
- 評估針對SARS-CoV-2抗原的抗體反應。

中國醫藥大學暨附設醫院
受試者同意書
（成年免疫第三劑組）

(三)試驗之主要納入與排除條件：

中國醫藥大學暨附設醫院執行本研究計畫的醫師或相關研究人員將會與您討論有關參加本研究的必要條件。請您配合必須誠實告知我們您過去的健康情形，若您有不符合參加本研究的情況，將不能參加本研究計畫。

1. 參加本研究計畫的主要條件:

☐(1) 您為納入試驗時20~85歲之間健康男性或未懷孕的女性受試者。

☐(2) 您為具生育能力的女性與男性應於首次接種疫苗至最後一次疫苗後3個月同意進行有效的避孕方式。可接受的有效避孕方式包括:

☐a. 男性或女性以手術方法絕育、植入式避孕、或子宮避孕器。

☐b. 注射避孕、避孕藥、避孕貼片、避孕環加上一種屏障避孕法*。

☐c. 合併使用兩種屏障避孕法*。

*有效的屏障避孕法為避孕隔膜、男性或女性保險套、避孕海綿或殺精劑(含可殺精化學物質的藥膏或凝膠)。

☐(3) 您能理解受試者同意書內容的說明與可能的風險，提供簽名的受試者同意書。

☐(4) 您能夠理解與遵從本試驗程序與能夠參與每次訪視。

☐(5) 您的耳溫≤ 38.0°C。

☐(6) 您依據醫療病史、身體檢查和試驗主持人的臨床判斷為健康受試者**可符合納入試驗資格。經試驗主持人判斷，即便您的病史穩定或且控制良好，但伴隨病情惡化而有提高嚴重新型冠狀病毒感染的風險。

**健康受試者有先前存在的穩定疾病者可以納入試驗，定義為該疾病在納入試驗前12週內沒有惡化至需要治療或住院的顯著變化和在納入試驗6個月內沒有惡化至需要治療或住院的顯著變化。

2. 若您有下列任一情況，您將無法參加本研究計畫:

☐(1) 您有接種疫苗後需要醫療介入的過敏性休克、蕁麻疹或其他顯著不良反應的病史。

☐(2) 您在篩選時或接種每劑疫苗前已懷孕女性或懷孕檢測為陽性的女性。

☐(3) 您為正在哺乳的女性，或計畫從接種第一劑疫苗至最後一劑疫苗後60天哺乳的女性。

☐(4) 您在接種第一劑疫苗前3天內，經試驗主持人判斷，患有任何急性疾病。

☐(5) 您在接種第一劑疫苗前1個月內有重大手術。

☐(6) 您是已知為人類免疫缺乏病毒抗體陽性。

☐(7) 您是已知為活動性B型肝炎或C型肝炎。活動性肝炎定義為肝臟轉胺酶(天門冬胺酸轉胺酶和/或丙胺酸轉胺酶)大於3倍正常值上限和/或總膽紅素大於3倍正常值上限。

☐(8) 您是已知曾暴露於新型冠狀病毒，或曾接受預防新型冠狀病毒、中東呼吸症候群冠狀病毒、嚴重急性呼吸道症候群的試驗或已上市產品。

☐(9) 您有格林-巴利症候群的病史。

☐(10) 您在簽署受試者同意書前12周內參與其他的臨床試驗。

☐(11) 您為免疫缺乏/失調疾病，無論是否由基因缺陷、免疫缺乏症或免疫抑制療法所造成。

☐(12) 您計畫或正在進行抗癌症治療。

☐(13) 您患有血小板異常或其他凝血異常可能造成注射之禁忌症。

☐(14) 您在接種第一劑疫苗前6個月長期接受(≥14天連續使用)免疫抑制劑、皮質類固醇(相當於一天使用≥ 20 mg強的松(prednisone))或細胞毒性治療。

☐(15) 您在接種第一劑疫苗前4個月接受免疫球蛋白和/或任何血液製劑的治療。

☐(16) 您在接種試驗疫苗前14天接種任何季流感疫苗或新型流感疫苗，或前28天接種其他疫苗。

☐(17) 您預期在接種試驗疫苗後14天接種任何季流感疫苗或新型流感疫苗，或後28天接種其他疫苗。

☐(18) 您使用短期(<14天使用)全身性類固醇。應於中斷使用全身性類固醇至少28天後才可使用試驗疫苗。吸入/噴霧性、關節注射、囊內或局部(皮膚或眼用)類固醇可允許使用。

☐(19) 您在篩選期前3個月失血或捐血超過500毫升，或預計在試驗期間內捐血或輸血。

☐(20) 經試驗主持人判斷，您有任何醫療疾病或狀況，可能會影響試驗結果或參與試驗可能會對受試者引發額外風險。

☐(21) 您是直接參與本試驗執行的試驗主持人所屬機構的試驗團隊、試驗委託者或受託

研究機構(CRO)的員工。

（四）試驗方法及相關檢驗：

這是一個第二期、觀察者盲性、多中心、隨機分派、安慰劑控制試驗，以評估青少年，成年和老年受試者使用兩劑UB-612疫苗的免疫原性，耐受度和安全性。有一部份的受試者使用UB-612疫苗，而另外一部份的受試者則使用「安慰劑」。所謂「安慰劑」是不含有效成份的疫苗。至於誰使用試驗用藥或誰使用「安慰劑」，則像丟銅板或擲骰子一樣由機率決定，不管是您或是研究醫師都不知道您使用了那一種藥，只有分發跟施打疫苗的試驗人員才知道您使用哪一種疫苗，這叫做觀察者盲性。

總計約有3850位合格成年受試者組成核心組用於申請緊急使用授權，另外大約有385位青少年受試者組成補充組申請額外適應症。所有受試者將以6:1的比例，隨機分派至兩劑100微克劑量組別和安慰劑組，包括462位大於18歲至小於65歲可評估的受試者進入批次分析組。對於免疫分析，至少包括350位可評估的成年受試者(年齡大於18歲至小於65歲)和154位可評估的老年受試者(年齡≥65歲)進行描述性分析。免疫原性的受試者將會先納入試驗。所有的受試者將會納入安全性分析，其中至少770位隨機分派的受試者為≥65歲的分層。青少年組將在核心組招募完畢後，再開始納入試驗。約有385位青少年受試者將以6:1的比例隨機分派，其中包括154位可評估的青少年受試者將收集免疫原性數據，並和成年及老年受試者數據進行比較。

您若參與這個試驗，則為有進行免疫分析檢測的免疫原性或批次一致組。

試驗總共有8個訪視。若您參與本試驗，則至少包括第一次訪視(篩選訪視)、第二次訪視（第1天，基礎值，隨機分派，第一次接種疫苗)、第三次訪視(第29天，第二次接種疫苗)、第四次訪視(第57天)、第五次訪視(第197天)。

在第五次訪視時，預計將進行個別解盲。若個別解盲後，得知您為施打疫苗的受試者，且你有意願且符合資格接種第三劑疫苗，將進入第六次訪視(第197~242天，第三次接種疫苗)，第七次訪視(接種疫苗後第14天)，及第八次訪視(第365天)。

整個試驗期間，預期您將參與試驗最長達13個月。

注意事項

1. 如果您同意參加本試驗，研究人員會請您簽署本份受試者同意書，並確認您符合參加本試驗的條件。
2. 從您參與試驗的當天開始，每次訪視都將有合格的試驗人員執行試驗流程與聯繫。

3. 若您有任何符合新型冠狀病毒感染的定義(您曾於過去 7 天內有出國，或是接觸疑似或確認武漢肺炎之病人，而有下列症狀: 發燒、開始咳嗽或惡化、開始呼吸急促或惡化、寒顫、開始肌肉疼痛或惡化、喉嚨痛、腹瀉、嘔吐、開始味覺/嗅覺異常)，請依照中央疫情指揮中心規定進行自主健康管理或至指定院所進行篩檢。
4. 若您在試驗期間感染新型冠狀病毒，將依法通報主管機關。

您是否同意? □是 □否

簽名：__________日期：____________

5. 於試驗期間，您不論任何理由提前退出試驗，試驗研究人員都將安排您完成最後一次的訪視之所有試驗項目。您有權利拒絕此項安排，您的決定不會引起任何影響日後醫師對您的醫療照護。

試驗步驟

第一次訪視(第-28~-1 天) -篩選訪視

在試驗醫師或試驗研究人員為您提供足夠的試驗資訊，並確保您有充分的時間考慮以及詢問任何問題後，您願意讓您參與本試驗，並由您簽署本受試者同意書。在確認您已完成受試者同意書簽署並且您也保有一份副本後，試驗醫師或試驗研究人員將會進行以下試驗程序：

(1) 記錄您簽署受試者同意書的日期
(2) 為您指定一組受試者篩選編號
(3) 確認您是否符合本試驗的納入排除條件
(4) 收集您的個人基本資料（例如生日、年齡及性別）
(5) 記錄您的醫療/用藥病史
(6) 進行身體檢查，包括身高體重
(7) 確認生命徵象
(8) 進行心電圖檢測
(9) 收集尿液檢體進行尿液檢測
(10) 收集血液檢體(共 15.5 毫升)，進行下列檢測:
- 常規血液檢測
- 血液生化學檢測
- 免疫學檢測(抗核抗體)

- 備血(作為測量新型冠狀病毒血清抗體和相關研究之用)

第二次訪視(第 1 天)-基礎值，確認符合試驗條件，第一次接種疫苗

(1) 再度確認您是否符合本試驗的納入排除條件
(2) 隨機分派，給予您一組隨機分派號碼
(3) 記錄您的醫療/用藥病史
(4) 進行身體檢查
(5) 確認生命徵象
(6) 進行尿液懷孕檢測（具有生育能力女性）
(7) 若您有產生第三級以上的高血壓，您將被收集尿液檢體以檢測是否有蛋白尿存在或惡化
(8) 收集血液檢體(若不進行 T 細胞檢測，共 20 毫升)，進行下列檢測:
  - 免疫原性檢測，包括 Anti-S1-RBD 免疫球蛋白 G 濃度，和新型冠狀病毒中和抗體效價
  - 若您願意，將進行 T 細胞檢測(需額外抽血 56 毫升)
(9) 進行第一次疫苗接種(注射部位為非慣用手，採用肌肉注射方式)。接種疫苗後，受試者應留在試驗地點至少 30 分鐘，監測生命徵象和急性過敏症狀。
(10) 將詳細地指導您如何填寫電子日誌卡(包括注射後 7 天內預期性不良事件，14 天內的皮膚過敏反應日誌卡)
(11) 收集併用藥物/治療

第一次電話安全性追蹤(第 8, 15, 22 天)

將與您電話聯繫，以追蹤未預期不良事件和新型冠狀病毒感染症狀。

第三次訪視(第 29± 3 天)-第二次接種疫苗

(1) 進行第二次接種評估(有可能會延遲接種時間)
(2) 進行身體檢查
(3) 確認生命徵象
(4) 進行尿液懷孕檢測（具生育能力的女性）
(5) 若您有產生第三級以上的高血壓，您將被收集尿液檢體以檢測是否有蛋白尿存在或惡化。
(6) 若您為批次一致/免疫原組，將收集血液檢體(共 5 毫升)，進行下列檢測:
  - 特定抗原 Anti-S1-RBD 抗體
(7) 進行第二次疫苗接種。
(8) 接種疫苗後，受試者應留在試驗地點至少 30 分鐘，監測生命徵象和急性過敏症狀。

(9) 將詳細地指導您如何填寫電子日誌卡(包括注射後 7 天內預期性不良事件，14 天內的皮膚過敏反應日誌卡)

(10) 收集併用藥物/治療

(11) 記錄上一次訪視至此次訪視之間的不良事件、嚴重不良事件型或新型冠狀病毒感染症狀

第二次電話安全性追蹤(第 36, 43 天)

將與您電話聯繫，以追蹤未預期不良事件和新型冠狀病毒感染症狀。

第四次訪視(第 57±3 天) –追蹤訪視

(1) 進行身體檢查

(2) 確認生命徵象

(3) 若您有產生第三級以上的高血壓，您將被收集尿液檢體以檢測是否有蛋白尿存在或惡化。

(4) 收集血液檢體(若不進行 T 細胞檢測，共 35.5 毫升) ，進行下列檢測:

- 免疫原性檢測，包括 Anti-S1-RBD 免疫球蛋白 G 濃度、和新型冠狀病毒中和抗體效價
- 若您願意，將進行 T 細胞檢測(需額外抽血 56 毫升)
- 常規血液檢測
- 血液生化學檢測
- 免疫學檢測(抗核抗體)
- 備血(作為測量新型冠狀病毒血清抗體和相關研究之用)

(5) 收集併用藥物/治療

(6) 記錄上一次訪視至此次訪視之間的不良事件、嚴重不良事件或新型冠狀病毒感染症狀

(7) 在此次訪視後，您將每周接獲訊息提醒，以定期監測新型冠狀病毒感染症狀

第三次電話安全性追蹤(第 64, 71, 78, 85 天)

將與您電話聯繫，以追蹤安全性及新型冠狀病毒感染症狀。

第五次訪視(第 197±15 天) –個別解盲

(1) 進行身體檢查

(2) 確認生命徵象

(3) 若您有產生第三級以上的高血壓，您將被收集尿液檢體以檢測是否有蛋白尿存在或惡化。

(4) 將替您個別解盲，告知您接種到疫苗或安慰劑。

(5) 收集血液檢體(共 20 毫升) ，進行下列檢測:

- 免疫原性檢測，包括 Anti-S1-RBD 免疫球蛋白 G 濃度、和新型冠狀病毒中和抗體效價

(6) 收集併用藥物/治療

(7) 記錄上一次訪視至此次訪視之間的不良事件、嚴重不良事件或新型冠狀病毒感染症狀

第六次訪視(第 197~242 天，第三次接種疫苗)

(1) 記錄您簽署受試者同意書的日期

(2) 確認您是否符合接種第三劑疫苗的資格，包括第三劑接種疫苗的禁忌症，或有延遲第三劑接種時間的條件

(3) 進行身體檢查

(4) 確認生命徵象

(5) 若您有產生第三級以上的高血壓，您將被收集尿液檢體以檢測是否有蛋白尿存在或惡化

(6) 收集血液檢體(**若不進行 T 細胞檢測，**共 20.5 毫升)，進行下列檢測:

- 常規血液檢測
- 血液生化學檢測
- 免疫學檢測(抗核抗體)
- 探索性試驗免疫反應檢測
- **若您願意，將進行 T 細胞檢測(需額外抽血 56 毫升)**

您是否同意? □是 □否

簽名：___________日期：_____________

(7) 進行尿液懷孕檢測（具有生育能力女性）

(8) 進行第三次疫苗接種。

(9) 接種疫苗後，受試者應留在試驗地點至少 30 分鐘，監測生命徵象和急性過敏症狀。將詳細地指導您如何填寫電子日誌卡(包括注射後 7 天內預期性不良事件，14 天內的皮膚過敏反應日誌卡)

(10) 收集併用藥物/治療

(11) 進行新型冠狀病毒監測

(12) 記錄上一次訪視至此次訪視之間的不良事件、嚴重不良事件型或新型冠狀病毒感染症狀

第六次訪視後第 7 天電話安全性追蹤

將與您電話聯繫，以追蹤未預期不良事件和新型冠狀病毒感染症狀。此外，還將監測皮膚過敏反應，或其他非預期的過敏反應。若您有發生任何第三級以上的過敏事件，試驗人員可能將安排您額外的回診。

第七次訪視 (第六次訪視後第 14± 3 天)

(1) 進行身體檢查
(2) 確認生命徵象
(3) 若您有產生第三級以上的高血壓，您將被收集尿液檢體以檢測是否有蛋白尿存在或惡化。
(4) 收集血液檢體(**若不進行 T 細胞檢測，**共 20.5 毫升)，進行下列檢測:
- 常規血液檢測
- 血液生化學檢測
- 免疫學檢測(抗核抗體)
- 探索性試驗免疫反應檢測
- **若您願意，將進行 T 細胞檢測(需額外抽血 56 毫升)**

(5) 收集併用藥物/治療
(6) 進行新型冠狀病毒監測
(7) 記錄上一次訪視至此次訪視之間的不良事件、嚴重不良事件型或新型冠狀病毒感染症狀

追蹤期電話安全性追蹤(第 253, 309 天)

第七次訪視後將每兩個月與您電話聯繫，以追蹤安全性及新型冠狀病毒感染症狀。

第八次訪視(第 365±45 天) –第 12 個月追蹤

(1) 確認生命徵象
(2) 收集血液檢體(共 20 毫升) ，進行下列檢測:
- 免疫原性檢測，包括 Anti-S1-RBD 免疫球蛋白 G 濃度、和新型冠狀病毒中和抗體效價

(3) 記錄上一次訪視至此次訪視之間的不良事件，包括特殊不良事件、醫療不良事件、嚴重不良事件或新型冠狀病毒感染症狀

受試者之檢體(含其衍生物)之保存、使用與再利用：

1. 檢體及剩餘檢體之保存與使用

(1) 檢體(含其衍生物)之保存與使用

為研究所需，我們所蒐集您的檢體，將依本研究計畫使用，檢體將保存於聯亞生技開發股份有限公司(試驗委託者)，直至20年保存期限屆滿，我們將依法銷毀。為了保護您的個人隱私，我們將以一個試驗編號來代替您的名字及相關個人資料，以確認您的檢體及與相關資料受到完整保密。如果您對檢體的使用有疑慮，或您有任何想要銷毀檢體的需求，請立即與我們聯絡(聯絡人：黃高彬醫師電話：0975-681-950)，我們即會將您的檢體銷毀。您也可以聯繫中國醫藥大學暨附設醫院研究倫理委員會(電話：04-22052121轉 1925、1926)，以協助您解決檢體在研究使用上的任何爭議。

(2) 剩餘檢體(含其衍生物)之再利用

您的生物檢體將會以專屬號碼進行編碼並在聯亞生技開發股份有限公司(試驗委託者)的控管下儲存最長20年，以研究UB-612 疫苗反應者的生物標記，及改善治療方式。

所有新的研究計畫都要再經由中國醫藥大學暨附設醫院研究倫理委員會審議通過，倫理審查委員會若認定新的研究超出您同意的範圍，將要求我們重新得到您的同意。

是否同意剩餘檢體保留提供未來新型冠狀病毒感染研究之用，並授權中國醫藥大學暨附設醫院研究倫理委員會審議是否需要再取得您的同意(擇一)

☐ 不同意保存我的剩餘檢體，試驗結束後請銷毀

☐ 同意以非去連結之方式保存我的剩餘檢體，逾越原同意使用範圍時，需再次得到我的同意才可使用我的檢體進行新的研究

2. 檢體及剩餘檢體之部分類型(檢體類型可依計畫書內容自行增減)

(1) 一般生化、血液檢驗/病毒檢測檢體

在試驗期間，會將您的檢體送往聯亞生技開發股份有限公司(試驗委託者)委託的中央實驗室中國醫藥大學暨附設醫院，此機構地址為台中市北區育德路2號，和大安聯合醫事檢驗所，此機構地址為台北市大安區復興南路二段151巷33號，中央實驗室會在分析後立即將分析結果提供給試驗中心，若有剩餘的檢體，將儲存直到至少完成臨床試驗報告為止，最長將保存20年。

(2) 抗體/細胞免疫試驗

在試驗期間，會將您的檢體送往聯亞生技開發股份有限公司(試驗委託者)分析實驗室。完成試驗後，若有剩餘檢體，將儲存直到至少完成臨床試驗報告為止，最長將保存20年。

(3) 中和試驗(neutralization test, NT)

在試驗期間，會將您的檢體送往聯亞生技開發股份有限公司(試驗委託者)委託的中央實驗室中央研究院進行處置、處理與進一步分析。此機構地址為台北市南港區研究院路二段128號。完成試驗後，若有剩餘檢體，將儲存直到至少完成臨床試驗報告為止，最長將保存20年。

(4) 遺傳學檢體

在試驗期間，若發生嚴重不良反應或特定不良反應，您的檢體將用於HLA分型檢驗，會將您的檢體送往聯亞生技開發股份有限公司(試驗委託者)委託的中央實驗室有勁基因股份有限公司分析，此機構地址為新北市樹林區復興路376-5號，中央實驗室不會將分析結果提供給試驗中心，若有剩餘的檢體，將會儲存直到檢驗結果複驗完畢即銷毀，不會長期儲存。

(5) 探索性試驗檢體

在試驗期間，會將您的檢體送往聯亞生技開發股份有限公司(試驗委託者)委託的實驗室(表一)進行處理或進一步分析。完成試驗後，若有剩餘檢體，將儲存直到至少完成臨床試驗報告為止，最長將保存20年。

表一、實驗室名稱與機構地址

實驗室名稱	機構地址
聯亞生技開發(股)公司	新竹縣竹北市生醫路二段 6-1 號 5 樓
Viroclinics	Rotterdam Science Tower, Marconistraat 16, 3029 AK Rotterdam, The Netherlands(荷蘭)
DASA	Jonas Cruz de Araujo, Diagnostics da America S/A, Surubiju Avenue, 1890, Barueri, SP, Brazil(巴西), 06455-040
PHE Porton Down	Salisbury Wiltshire SP4 0JG, England(英國)
UTMB	University of Texas Medical Branch 301 University Boulevard Keiller Building, Room 2.150 Galveston, Texas, USA(美國
Virology	University of São Paulo, Brazil Rua Dr EnnEn de Carvalho Aguiar 470, CEP 05403-000 (巴西)

VRDL	California Department of Public Health, 850 Marina Bay Parkway, Richmond, CA 94804, USA(美國)
NEXELIS	525 Boul. Cartier Ouest Laval, Qulbec, Canada, H7V 3S8(加拿大)
Vaccinology and Immunology Infection, Immunity & Inflammation Dept UCL GOS Institute of Childe Health	UCL Great Ormond Street Institute of Child Health 30 Guilford Street London WC1N 1EH, England(英國)
VisMederi	VisMederi Srl, Strada del Petriccio e Belriguardo, 35, 53100 Siena, Italy(義大利)

(五)可能產生之副作用、發生率及處理方法：

1. 與試驗藥物相關的風險（本試驗疫苗的副作用）：

<u>冠狀病毒疫苗的開發</u>

過去針對與SARS-CoV-2病毒相同屬於人類冠狀病毒的SARS-CoV(嚴重急性呼吸綜合症冠狀病毒(SARS冠狀病毒))的疫苗研究發現，接種過SARS-CoV疫苗的小鼠在暴露到SARS-CoV後會發生過度免疫反應而產生病變，因此不得不停止這種疫苗的開發。所以，成功的人類冠狀病毒疫苗不只要產生可以抑制病毒的免疫反應，更要避免過度免疫產生的副作用。

<u>疫苗相關的風險: 第一期臨床試驗</u>

接種疫苗可能會出現注射部位的不良反應(例如疼痛、硬化腫脹、皮疹發紅、過敏反應、蜂窩性組織炎)，或全身性不良反應(例如發燒、腹瀉、疲倦、噁心/嘔吐、厭食、咽喉痛、頭痛、咳嗽、關節痛、非注射部位疼痛、非注射部位搔癢、皮膚和黏膜異常、急性過敏反應、昏厥、急性支氣管痙攣、呼吸困難)。

第一期臨床試驗已經有60位受試者接種兩劑疫苗(含10微克、30微克、100微克融合蛋白)，安全實驗室數值並沒有顯示有任何的臨床顯著不正常數值，也沒有發生任何第三級以上與疫苗相關的預期性不良事件。大部分的預期性不良事件都是輕微的，大約於2天之內症狀都會緩解。也沒有任何的嚴重不良事件或特殊不良事件被通報。

<u>疾病增強(disease enhancement) 的風險</u>

SARS-CoV-2候選疫苗也可能會有引發疾病增強(disease enhancement) 的風險，包括抗體依賴性增強(antibody-dependent enhancement)或疫苗相關聯的增強的呼吸道疾病(vaccine-associated enhanced respiratory disease)。在先前研發SARS疫苗時，在數個SARS-CoV動物攻毒試驗(包括鼠類、雪貂、猴類)當中，有發現疾病增強的現象。疾病增強反應的免疫病理現象包括TH2偏向及嗜酸性白血球的肺部浸潤。但是目前已發表的新

型冠狀病毒肺炎疫苗研究，仍尚未發現類似的疾病增強現象。

本試驗疫苗於數個藥理試驗呈現不一致的TH1/TH2 (輔助型T細胞1/輔助型T細胞2)免疫反應偏向，試驗結果並未一致偏向TH2，而由小鼠之SARS-CoV-2動物攻毒試驗結果顯示，本試驗疫苗誘發疾病增強之風險不高。依據文獻指出，組成複雜或容易引起非中和抗體之抗原，如不活化病毒或整片段之蛋白(包含S蛋白與N蛋白)，與易引起偏向Th2免疫反應之佐劑成分，如鋁製佐劑，皆較有可能引起疾病增強。本試驗疫苗的主要抗原為S蛋白上之RBD 區域，已有多篇文獻指出，針對S-RBD設計之SARS與MERS疫苗從未於試驗動物模型上引發疾病增強現象。本試驗疫苗雖使用易引起偏向Th2免疫反應之佐劑，但由動物實驗證實，也同時引起偏向Th1之反應，因此發生疾病增強應屬低風險。且已於多種動物模型中證實，能誘發高效價之中和抗體，於細胞培養中亦能有效抑制新冠病毒感染。

建議您在有效疫苗上市前或本試驗疫苗的產品資訊有進一步更新前，盡量避免暴露於可能感染病毒的環境。研究團隊將會在試驗中執行相關安全性監測。若有任何關於本試驗疫苗與疾病增強風險相關之任何最新資訊，將即時更新並提供給您。

<u>疫苗佐劑相關的風險</u>

本試驗疫苗所使用的佐劑含Adju-Phos®，是屬於一種磷酸鋁類的佐劑。磷酸鋁類佐劑已經使用超過半個世紀，具有相當的安全性。由於此類佐劑可誘導免疫反應，因此可能會造成局部發炎反應，例如在注射部位產生輕微而短暫的疼痛、發紅以及腫脹。

2. 與試驗/研究過程相關的風險：

<u>抽血</u>

本試驗需要抽血檢驗。抽血可能引起一些不適和瘀血。整個試驗期間13個月，共需抽血157毫升。若您願意抽血檢驗T細胞檢測，則會**於該次訪視每次額外抽血56毫升**。若您罹患新型冠狀病毒感染，可能將於每次額外訪視抽血30毫升。

在接種疫苗過程中，可能會出現一些尚未在已完成試驗中發現的副作用。一般而言，接種某一新疫苗總是會有一定的風險，但是計畫主持人會採取一切措施預防風險的發生。**計畫主持人鼓勵您報告您遇到的任何不適。**

（六）其他替代療法及說明：

您不是非參加不可，若不參加研究，由於目前尚未有疫苗可用來預防新型冠狀病毒感染，因此預防措施與其他呼吸道感染相同，包括：勤洗手、減少觸摸眼口鼻、注意咳嗽禮節、妥善處理口鼻分泌物等，避免出入公共場所，並不要接觸野生動物。

如果您對於本試驗疫苗有任何的疑問，您可以提出來向您的試驗醫師討論。

(七)試驗預期效益：

依據臨床前試驗結果，預期本試驗疫苗對您可能可以產生抗體，預防新型冠狀病毒感染，但因每個人體質不同也有可能不會產生療效，故參加本試驗可能不會有直接的好處。

但是您參加本試驗，可協助我們獲得更多資訊，以瞭解UB-612疫苗的安全性與免疫力。

(八)試驗進行中受試者之禁忌、限制與應配合之事項：

禁止使用的藥物

以下藥物請勿在試驗期間使用：

- 直到試驗第197天禁止使用免疫抑制劑、或細胞毒性治療
- 到試驗第197天禁止使用免疫球蛋白和/或任何血液製劑
- 整個試驗期間禁止使用試驗產品(包括藥物或疫苗)
- 到試驗第197天禁止使用全身性皮質類固醇(相當於一天使用≥ 20 mg強的松(prednisone))
- 接種試驗疫苗後14天禁止接種任何季流感疫苗或新型流感疫苗，或後28天禁止接種其他非試驗疫苗。整個試驗期間禁止使用任何已上市的新型冠狀病毒疫苗產品。

允許使用的藥物

若您的藥物或治療必須常規使用，經試驗醫師判斷不會影響本試驗疫苗的免疫原性、臨床療效與安全性，則可以正常使用。您有任何關於在試驗期間可允許使用何種藥物或治療的問題，請詢問您的試驗醫師。

懷孕或母乳哺乳的風險

目前未知本試驗疫苗對於未出生胎兒的影響，因此：

- 您為具生育能力的女性受試者 (除非手術絕育或停經)，或您為男性受試者應於接種疫苗至最後一次疫苗後 3 個月同意進行有效的避孕方式，同意進行有效的避孕方式(例如子宮內節育器、荷爾蒙療法或避孕套)。
- 若您為具生育能力的女性，將請您進行懷孕檢測，結果必須為陰性，方可參與試驗。

- 若您為懷孕的女性，將被告知不可參與本試驗。
- 若您在試驗期間懷孕，請盡速通知試驗人員，並且停止施打本疫苗。
- 基於安全性考量，若您為女性受試者而在試驗期間懷孕，或您為男性受試者而您的性伴侶在試驗期間懷孕(將請您的懷孕性伴侶需簽署另外一份同意書)，您與您的胎兒將會被追蹤監測至分娩，除非另有醫學指示。

您應向您的配偶或性伴侶告知您有參與此試驗與相關風險:

簽名：__________日期：____________________

（九）機密性：

中國醫藥大學附設醫院將依法把任何可辨識您的身分之紀錄與您的個人隱私資料視為機密來處理，不會公開。研究人員將以一個研究代碼代表您的身分，此代碼不會顯示您的姓名、國民身分證統一編號、住址等可識別資料。如果發表試驗/研究結果，您的身分仍將保密。您亦瞭解若簽署同意書即同意您的原始醫療紀錄可直接受監測者、稽核者、研究倫理委員會及主管機關檢閱，以確保臨床試驗/研究過程與數據符合相關法律及法規要求，上述人員並承諾絕不違反您的身分之機密性。除了上述機構依法有權檢視外，我們會小心維護您的隱私。由於試驗藥物可能同時申請美國臨床試驗，依美國藥品管理規定，試驗結果將公佈於公開的臨床試驗資訊網站：Clinicaltrials.gov (美國)，但您的個人資料仍將保密，該網站只會有試驗之結果摘要，您可以在任何時候搜尋該網站。

在試驗/研究期間，依據計畫類型與您所授權的內容，我們將會蒐集與您有關的病歷資料、醫療紀錄、量表、問卷等資料與資訊，並以一個編號來代替您的名字及相關個人資料。前述資料若為紙本型式，將會與本同意書分開存放於研究機構之上鎖櫃中；若為電子方式儲存或建檔以供統計與分析之用，將會存放於設有密碼與適當防毒軟體之專屬電腦內。這些研究資料與資訊將會保存至藥品於我國上市後至少兩年，若試驗疫苗終止研發則保存至試驗正式停止後至少二年，至多將保存至疫苗上市後或試驗正式停止後二年。上述資料與資訊若傳輸至國外分析與統計，您仍會獲得與本國法規相符之保障，計畫主持人與相關團隊將盡力確保您的個人資料獲得妥善保護。

（十）損害補償與保險：

1. 如依本研究所訂臨床試驗計畫，因發生不良反應造成損害，由聯亞生技開發股份

有限公司負補償責任。但本受試者同意書上所記載之可預期不良反應，不予補償。

2. 如依本研究所訂臨床試驗計畫，因而發生不良反應或損害，贊助廠商將依法負責損害賠償責任。本醫院願意提供專業醫療照顧及醫療諮詢。您不必負擔治療不良反應或損害之必要醫療費用。
3. 除前二項補償及醫療照顧外，本研究不提供其他形式之補償。若您不願意接受這樣的風險，請勿參加試驗。
4. 您不會因為簽署本同意書，而喪失在法律上的任何權利。
5. 本研究有投保責任保險。

（十一）受試者權利：

1. 試驗過程中，與您的健康或是疾病有關，可能影響您繼續接受臨床試驗意願的任何重大發現，都將即時提供給您。
2. 如果您在試驗過程中對試驗工作性質產生疑問，對身為患者之權利有意見或懷疑因參與研究而受害時，可與本院之研究倫理委員會聯絡請求諮詢，其電話號碼為：04-22052121轉1925、1926。
3. 為進行試驗工作，您必須接受黃高彬醫師的照顧。如果您現在或於試驗期間有任何問題或狀況，請不必客氣，可與在中國醫藥大學附設醫院兒童感染科的黃高彬醫師聯絡（24小時聯繫電話：0975-681-950）。
4. 參加試驗研究計畫之補助：本計畫將在每次訪視提供交通費及營養費給您，新增的兩個診次(第六次返診、第七次返診)將各提供費用1500元；整個試驗預計給予您21000元。若您願意參與T細胞檢測研究，將在該次訪視另外提供營養費500元給您。
5. 本同意書一式2份，醫師已將同意書副本交給您，並已完整說明本研究之性質與目的。醫師已回答您有關藥品與研究的問題。

（十二）試驗之退出與中止：

您可自由決定是否參加本試驗；試驗過程中也可隨時撤銷同意，退出試驗，不需任何理由，且不會引起任何不愉快或影響其日後醫師對您的醫療照顧。

計畫主持人或贊助廠商亦可能於必要時中止該試驗之進行。

（十三）簽名：

1. 計畫主持人、或協同主持人已詳細解釋有關本研究計畫中上述研究方法的性質與目的，及可能產生的危險與利益。

   計畫主持人/協同主持人簽名：____________日期：________年____月____日
2. 受試者已詳細瞭解上述研究方法及其所可能產生的危險與利益，有關本試驗計畫

的疑問，業經試驗主持人詳細予以解釋。本人同意接受為臨床試驗計畫的自願受試者。

受試者簽名：______________________________日期：_________年____月____日

法定代理人簽名：__________________________日期：_________年____月____日

* 受試者為無行為能力(未滿七歲之未成年人者或禁治產人)，由法定代理人為之；禁治產人，由監護人擔任其法定代理人。

* 受試者為限制行為人者(滿七歲以上之未成年人)，應得法定代理人之同意。

有同意權人簽名：__________________________日期：_________年____月____日

* 受試者雖非無行為能力或限制行為能力者，但因意識混亂或有精神與智能障礙，而無法進行有效溝通和判斷時，由有同意權之人為之。前項有同意權人為配偶及直系親屬。

3. 見證人

見證人簽名：_______________________________日期：_________年____月____日

身分證字號：　　　　　　　　　　　　　聯絡電話：

通訊地址：

* 受試者、法定代理人或有同意權之人皆無法閱讀時，應由見證人在場參與所有有關受試者同意之討論。並確定受試者、法定代理人或有同意權之人之同意完全出於其自由意願後，應於受試者同意書簽名並載明日期。試驗相關人員不得為見證人。

中國醫藥大學暨附設醫院
受試者同意書
（成年免疫第三劑組）

流程表:

<u>批次分析與免疫分析組</u>

訪視	1[m]	2[m]		3		4		5/提早退出試驗	6[t]		7		長期性追蹤
檢測項目	篩選	第一次接種		第二次接種		追蹤[g]		個別解盲	第三次接種[h]		追蹤[h]		第12個月追蹤[i]
天數	-28~-1	1	8, 15, 22	29 ±3天	36, 43	57 ±3天	64, 71, 78,85	197 ±15天	197~242	第六次訪視後7天	第六次訪視後14天 ±3天	253, 309	365 ±45天
獲得受試者同意書	X								X[h]				
納入/排除條件	X	X											
隨機分派		X											
接種評估				X					X[h]				
基本資料	X												
醫療病史	X	X											
身體檢查[a]	X	X		X		X		X	X[h]		X[h]		
生命徵象	X	X		X		X		X	X[h]		X[h]		X
心電圖	X												
**實驗室檢測 (安全性)**													
血液常規檢測[j]	X					X			X[h]		X[h]		
血液生化學檢測[j]	X					X			X[h]		X[h]		
免疫檢測[j]	X					X			X[h]		X[h]		
懷孕檢測[b]		X		X					X[h]				

中國醫藥大學暨附設醫院
受試者同意書
（成年免疫第三劑組）

訪視	1[m]	2[m]		3		4		5/提早退出試驗	6[t]		7		長期性追蹤
檢測項目	篩選	第一次接種		第二次接種		追蹤[g]		個別解盲	第三次接種[h]		追蹤[h]		第 12 個月追蹤[i]
天數	-28~-1	1	8, 15, 22	29 ±3 天	36, 43	57 ±3 天	64, 71, 78,85	197 ±15 天	197~242	第六次訪視後 7 天	第六次訪視後 14 天 ±3 天	253, 309	365 ±45 天
尿液常規檢測[b]	X	X[q]		X[q]		X[q]		X[q]	X[h, q]		X[h, q]		
**實驗室檢測(免疫原性)**													
免疫原性[c]		X		X[n]		X		X					X
T 細胞反應(可選擇[o])		X				X							
**實驗室檢測(探索性試驗)**													
T 細胞功能性檢測(可選擇[u])									X[h]		X[h]		
備血	X					X							
免疫原性[c]									X[h]		X[h]		
疫苗接種		X		X					X[h]				
指導使用電子日誌卡		X		X					X[h]				
電話安全性追蹤[d]			X		X		X			X[s,h]		X	
不良事件/特殊不良事件[p]/醫		X[k]	X[k]	X[k]	X[k]	X[k]	X[k]	X[l]	X[l, h]	X[l, h]	X[l, h]	X[l]	X[l]

訪視	1[m]	2 [m]		3		4		5/提早退出試驗	6[t]		7		長期性追蹤
檢測項目	篩選	第一次接種		第二次接種		追蹤 [g]		個別解盲	第三次接種 [h]		追蹤 [h]		第 12 個月追蹤 [i]
天數	-28~-1	1	8, 15, 22	29 ±3 天	36, 43	57 ±3 天	64, 71, 78,85	197 ±15 天	197~242	第六次訪視後 7 天	第六次訪視後 14 天 ±3 天	253, 309	365 ±45 天
療需求不良事件/嚴重不良事件													
新型冠種病毒感染監測		X	X	X	X	X [e]	X [e]	X [e]	X [e, h]	X [e, h]	X [e, h]	X [e]	X [e]
併用藥物		X		X		X		X [f]	X [f, h]		X [f, h]		

a: 身高與體重僅在第一次訪視測量。
b:於第 1, 29 天使用尿液懷孕檢測。若尿液檢測為陽性，應以血清懷孕檢測再次確認。以血清懷孕檢測替代尿液檢測將不視為試驗偏差。蛋白尿將透過尿液常規檢測確認，做為基礎值。
c: 針對 Anti-S1-RBD 免疫球蛋白 G 濃度和新型冠狀病毒中和抗體效價，以及抑制 S1-RBD: ACE2 的抗體效價。
d: 所有受試者將進行電話安全追蹤以監測非預期性不良事件，包括特殊不良事件，和監測新型冠狀病毒感染的症狀。
e: 受試者將在每周(為每 7 天)由手機接獲一條提示以規律監測新型冠狀病毒感染症狀或病徵。記錄疑似新冠病毒感染所使用的藥物至新冠病毒感染頁面。
f: 只記錄醫療需求不良事件和嚴重不良事件之併用藥物。
g: 第二次接種疫苗後第 28 天
h: 僅針對同意並進行施打第三劑試驗疫苗的疫苗組受試者
i: 第二次接種疫苗後第十二個月(第 365 天)
j: 安全性實驗室數值包括全血液計數(血紅素、血比容、紅血球計數)、白血球計數、血小板計數、肌酸酐、丙胺酸轉胺酶、天門冬胺酸轉胺酶、總膽紅素、直接膽紅素、高靈敏度 C 反應性蛋白、抗核抗體
k: 主動收集時期
l: 被動監測時期
m: 第一次訪視與第二次訪視可為同一次訪視。
n: 僅測量 Anti-S1-RBD IgG 濃度
o: 將在選定的試驗地點納入至少 100 位>18-<65 歲的受試者。
p : 包括接種最後一劑疫苗後 12 個月，可能的免疫媒介醫療狀況(PIMMC)或任何定義為可能的特殊不良事件。新冠病毒感染的併發症也將視為疾病增強事件，將被記錄與通報為特殊不良事件。
q: 若受試者有≥第三級以上的高血壓，受試者將確認是否有蛋白尿存在或惡化。
r: 備血將被冷凍存放做為 UBI 新型冠狀病毒酵素結合免疫吸附分析法，新型冠狀病毒確認酵素結合免疫吸附分析法，以及未來免疫學研究之用。
s: 若符合資格並接種第三劑疫苗的受試者將進行兩週的日誌卡追蹤紀錄，並將於接種後第 7 天進行電話安全性追蹤。
t: 第五次訪視和第六次訪視可為同一天。

中國醫藥大學暨附設醫院

受試者同意書

（成年免疫第三劑組）

u: 在選定之試驗地點中，將邀請大約 30 位年齡 >18-<65 歲的受試者和大約 30 位年齡 ≥65 歲的受試者。在適用的情況下，優先邀請曾進行過第 57 天 T 細胞功能評估的受試者。

中國醫藥大學暨附設醫院
受試者同意書
（成年安全確認第三劑組）

黃高彬
2021.10.25

您被邀請參與此研究。此同意書主要是提供您本研究之相關資訊，以便您決定是否參加本研究。計畫主持人或其指定之研究人員會為您說明研究內容並回答您的疑問。您可以提出任何和此研究有關的問題，在您的問題尚未獲得滿意的答覆之前，請不要簽署此同意書。如果您願意參與本研究，此文件將視為您的同意紀錄。即使在您同意後，您可以隨時退出本研究不需任何理由。

<table>
<tr><td colspan="2">計畫名稱
中文：一個評估 UB-612 疫苗對於新型冠狀病毒於青少年、成年和老年健康受試者的免疫原性、安全性與耐受性的第二期、安慰劑控制、隨機分派、觀察者盲性臨床試驗
英文：A Phase II, Placebo-controlled, Randomized, Observer-blind Study to Evaluate the Immunogenicity, Safety and Tolerability of UB-612 Vaccine against COVID-19 in Adolescent, Younger and Elderly Adult Volunteers</td></tr>
<tr><td>執行單位：中國醫藥大學附設醫院感染科、家庭醫學科</td><td>委託單位/藥廠：聯亞生技開發股份有限公司
研究經費來源：聯亞生技開發股份有限公司
受託研究機構：晉加股份有限公司</td></tr>
<tr><td>計畫主持人：黃高彬</td><td>職稱：主治醫師</td></tr>
<tr><td>協同主持人：林文元</td><td>職稱：主治醫師</td></tr>
<tr><td>協同主持人：林伯昌</td><td>職稱：主治醫師</td></tr>
<tr><td>緊急聯絡人：黃高彬</td><td>電話：0975-681-950</td></tr>
<tr><td>受試者姓名：
性別：
身分證字號：
通訊地址：</td><td>病歷號碼：
出生日期：
聯絡電話：</td></tr>
<tr><td>法定代理人或有同意權人之姓名：
性別：
身分證字號：
通訊地址：</td><td>與受試者關係：
出生日期：
聯絡電話：</td></tr>
<tr><td colspan="2">(一)試驗簡介：
1. 本品/技術資料：</td></tr>
</table>

中國醫藥大學暨附設醫院
受試者同意書
（成年安全確認第三劑組）

新型冠狀病毒(SARS-CoV-2)於2019年12月起造成中國湖北省武漢市發現多起病毒性肺炎群聚，隨後於2020年1月底台灣出現第一起境外移入確診個案。此疾病在全球擴散，世界衛生組織宣布將此疫情為「國際關注公共衛生緊急事件」。截至2020年底，全球僅有數間疫苗公司，如美國輝瑞藥廠等，取得緊急使用授權上市。

UB-612疫苗為聯亞生技開發股份有限公司所開發新型冠狀病毒預防性疫苗，疫苗含病毒棘狀融合蛋白和胜肽片段，可產生高親和力抗體與新型冠狀病毒結合，並誘發細胞免疫反應，進而達到預防新型冠狀病毒的感染。

UB-612第一期延伸性試驗顯示，接種第三劑UB-612疫苗可以誘發極高的中和抗體，在目前變種病毒的威脅之下，施打第三劑加強免疫反應，已是許多國家的選擇。

2. 本品上市狀況：

本品仍應用於人體試驗，尚未在我國上市。

3. 本試驗使用的UB-612疫苗對新型冠狀病毒的預防效果仍未確認。

4. 您簽署這份受試者同意書是由於您願意接種第三劑UB-612疫苗。

（二）試驗目的：

主要試驗目的

- 評估UB-612疫苗誘發的新型冠狀病毒中和抗體效價。
- 評估接種UB-612疫苗後的安全性和耐受性。

次要試驗目的

- 評估在試驗期間對於新型冠狀病毒的免疫反應。
- 評估三批獨立批次疫苗的批次免疫一致性。

探索性試驗目的

- 評估UB-612疫苗誘發的T細胞功能。
- 評估UB-612疫苗在年輕受試者的安全性和免疫原性。
- 評估UB-612疫苗的療效。
- 描述UB-612疫苗於確診和/或嚴重感染新型冠狀病毒案例之血液學反應。
- 評估針對SARS-CoV-2抗原的抗體反應。

(三)試驗之主要納入與排除條件：

中國醫藥大學暨附設醫院執行本研究計畫的醫師或相關研究人員將會與您討論有關參加本研究的必要條件。請您配合必須誠實告知我們您過去的健康情形，若您有不符合參加本研究的情況，將不能參加本研究計畫。

1. 參加本研究計畫的主要條件:

☐(1) 您為納入試驗時20~85歲之間健康男性或未懷孕的女性受試者。

☐(2) 您為具生育能力的女性與男性應於首次接種疫苗至最後一次疫苗後3個月同意進行有效的避孕方式。可接受的有效避孕方式包括:

☐a. 男性或女性以手術方法絕育、植入式避孕、或子宮避孕器。

☐b.注射避孕、避孕藥、避孕貼片、避孕環加上一種屏障避孕法*。

☐c.合併使用兩種屏障避孕法*。

*有效的屏障避孕法為避孕隔膜、男性或女性保險套、避孕海綿或殺精劑(含可殺精化學物質的藥膏或凝膠)。

☐(3) 您能理解受試者同意書內容的說明與可能的風險，提供簽名的受試者同意書。

☐(4) 您能夠理解與遵從本試驗程序與能夠參與每次訪視。

☐(5) 您的耳溫≤ 38.0°C。

☐(6) 您依據醫療病史、身體檢查和試驗主持人的臨床判斷為健康受試者**可符合納入試驗資格。經試驗主持人判斷，即便您的病史穩定或且控制良好，但伴隨病情惡化而有提高嚴重新型冠狀病毒感染的風險。

**健康受試者有先前存在的穩定疾病者可以納入試驗，定義為該疾病在納入試驗前12週內沒有惡化至需要治療或住院的顯著變化和在納入試驗6個月內沒有惡化至需要治療或住院的顯著變化。

2. 若您有下列任一情況，您將無法參加本研究計畫:

☐(1) 您有接種疫苗後需要醫療介入的過敏性休克、蕁麻疹或其他顯著不良反應的病史。

☐(2) 您在篩選時或接種每劑疫苗前已懷孕女性或懷孕檢測為陽性的女性。

☐(3) 您為正在哺乳的女性，或計畫從接種第一劑疫苗至最後一劑疫苗後60天哺乳的女性。

☐(4) 您在接種第一劑疫苗前3天內，經試驗主持人判斷，患有任何急性疾病。

☐(5) 您在接種第一劑疫苗前1個月內有重大手術。

☐(6) 您是已知為人類免疫缺乏病毒抗體陽性。

☐(7) 您是已知為活動性B型肝炎或C型肝炎。活動性肝炎定義為肝臟轉胺酶(天門冬胺酸轉胺酶和/或丙胺酸轉胺酶)大於3倍正常值上限和/或總膽紅素大於3倍正常值上限。

☐(8) 您是已知曾暴露於新型冠狀病毒，或曾接受預防新型冠狀病毒、中東呼吸症候群冠狀病毒、嚴重急性呼吸道症候群的試驗或已上市產品。

☐(9) 您有格林-巴利症候群的病史。

☐(10) 您在簽署受試者同意書前12周內參與其他的臨床試驗。

☐(11) 您為免疫缺乏/失調疾病，無論是否由基因缺陷、免疫缺乏症或免疫抑制療法所造成。

☐(12) 您計畫或正在進行抗癌症治療。

☐(13) 您患有血小板異常或其他凝血異常可能造成注射之禁忌症。

☐(14) 您在接種第一劑疫苗前6個月長期接受(≥14天連續使用)免疫抑制劑、皮質類固醇(相當於一天使用≥ 20 mg強的松(prednisone))或細胞毒性治療。

☐(15) 您在接種第一劑疫苗前4個月接受免疫球蛋白和/或任何血液製劑的治療。

☐(16) 您在接種試驗疫苗前14天接種任何季流感疫苗或新型流感疫苗，或前28天接種其他疫苗。

☐(17) 您預期在接種試驗疫苗後14天接種任何季流感疫苗或新型流感疫苗，或後28天接種其他疫苗。

☐(18) 您使用短期(<14天使用)全身性類固醇。應於中斷使用全身性類固醇至少28天後才可使用試驗疫苗。吸入/噴霧性、關節注射、囊內或局部(皮膚或眼用)類固醇可允許使用。

☐(19) 您在篩選期前3個月失血或捐血超過500毫升，或預計在試驗期間內捐血或輸血。

☐(20) 經試驗主持人判斷，您有任何醫療疾病或狀況，可能會影響試驗結果或參與試驗可能會對受試者引發額外風險。

☐(21) 您是直接參與本試驗執行的試驗主持人所屬機構的試驗團隊、試驗委託者或受託

研究機構(CRO)的員工。

(四)試驗方法及相關檢驗：

這是一個第二期、觀察者盲性、多中心、隨機分派、安慰劑控制試驗，以評估青少年，成年和老年受試者使用兩劑UB-612疫苗的免疫原性，耐受度和安全性。有一部份的受試者使用UB-612疫苗，而另外一部份的受試者則使用「安慰劑」。所謂「安慰劑」是不含有效成份的疫苗。至於誰使用試驗用藥或誰使用「安慰劑」，則像丟銅板或擲骰子一樣由機率決定，不管是您或是研究醫師都不知道您使用了那一種藥，只有分發跟施打疫苗的試驗人員才知道您使用哪一種疫苗，這叫做觀察者盲性。

總計約有3850位合格成年受試者組成核心組用於申請緊急使用授權，另外大約有385位青少年受試者組成補充組申請額外適應症。所有受試者將以6:1的比例，隨機分派至兩劑100微克劑量組別和安慰劑組，包括462位大於18歲至小於65歲可評估的受試者進入批次分析組。對於免疫分析，至少包括350位可評估的成年受試者(年齡大於18歲至小於65歲)和154位可評估的老年受試者(年齡≥65歲)進行描述性分析。免疫原性的受試者將會先納入試驗。所有的受試者將會納入安全性分析，其中至少770位隨機分派的受試者為≥65歲的分層。青少年組將在核心組招募完畢後，再開始納入試驗。約有385位青少年受試者將以6:1的比例隨機分派，其中包括154位可評估的青少年受試者將收集免疫原性數據，並和成年及老年受試者數據進行比較。

您若參與這個試驗，則為進行安全性確認的安全確認組。

試驗總共有8個訪視。若您參與本試驗，則至少包括第一次訪視(篩選訪視)、第二次訪視（第1天，基礎值，隨機分派，第一次接種疫苗)、第三次訪視(第29天，第二次接種疫苗)、第四次訪視(第57天)、第五次訪視(第197天)。

在第五次訪視時，預計將進行個別解盲。若個別解盲後，得知您為施打疫苗的受試者，且你有意願且符合資格接種第三劑疫苗，將進入第六次訪視(第197~242天，第三次接種疫苗)，第七次訪視(接種疫苗後第14天)，及第八次訪視(第365天)。

整個試驗期間，預期您將參與試驗最長達13個月。

<u>注意事項</u>

1. 如果您同意參加本試驗，研究人員會請您簽署本份受試者同意書，並確認您符合參加本試驗的條件。
2. 從您參與試驗的當天開始，每次訪視都將有合格的試驗人員執行試驗流程與聯繫。

3. 若您有任何符合新型冠狀病毒感染的定義(您曾於過去 7 天內有出國，或是接觸疑似或確認武漢肺炎之病人，而有下列症狀: 發燒、開始咳嗽或惡化、開始呼吸急促或惡化、寒顫、開始肌肉疼痛或惡化、喉嚨痛、腹瀉、嘔吐、開始味覺/嗅覺異常)，請依照中央疫情指揮中心規定進行自主健康管理或至指定院所進行篩檢。
4. 若您在試驗期間感染新型冠狀病毒，將依法通報主管機關。

您是否同意? □是 □否

簽名：__________日期：__________

5. 於試驗期間，您不論任何理由提前退出試驗，試驗研究人員都將安排您完成最後一次的訪視之所有試驗項目。您有權利拒絕此項安排，您的決定不會引起任何影響日後醫師對您的醫療照護。

試驗步驟

第一次訪視(第-28~-1 天) -篩選訪視

在試驗醫師或試驗研究人員為您提供足夠的試驗資訊，並確保您有充分的時間考慮以及詢問任何問題後，您願意讓您參與本試驗，並由您簽署本受試者同意書。在確認您已完成受試者同意書簽署並且您也保有一份副本後，試驗醫師或試驗研究人員將會進行以下試驗程序：

(1) 記錄您簽署受試者同意書的日期
(2) 為您指定一組受試者篩選編號
(3) 確認您是否符合本試驗的納入排除條件
(4) 收集您的個人基本資料（例如生日、年齡及性別）
(5) 記錄您的醫療/用藥病史
(6) 進行身體檢查，包括身高體重
(7) 確認生命徵象
(8) 進行心電圖檢測
(9) 收集尿液檢體進行尿液檢測
(10) 收集血液檢體(共 15.5 毫升)，進行下列檢測:
- 常規血液檢測
- 血液生化學檢測
- 免疫學檢測(抗核抗體)

- 備血(作為測量新型冠狀病毒血清抗體和相關研究之用)

第二次訪視(第 1 天)-基礎值，確認符合試驗條件，第一次接種疫苗

(1) 再度確認您是否符合本試驗的納入排除條件
(2) 隨機分派，給予您一組隨機分派號碼
(3) 記錄您的醫療/用藥病史
(4) 進行身體檢查
(5) 確認生命徵象
(6) 進行尿液懷孕檢測（具有生育能力女性）
(7) 若您有產生第三級以上的高血壓，您將被收集尿液檢體以檢測是否有蛋白尿存在或惡化
(8) 進行第一次疫苗接種(注射部位為非慣用手，採用肌肉注射方式)。接種疫苗後，受試者應留在試驗地點至少 30 分鐘，監測生命徵象和急性過敏症狀。
(9) 將詳細地指導您如何填寫電子日誌卡(包括注射後 7 天內預期性不良事件,14 天內的皮膚過敏反應日誌卡)
(10) 收集併用藥物/治療

第一次電話安全性追蹤(第 8, 15, 22 天)

將與您電話聯繫，以追蹤未預期不良事件和新型冠狀病毒感染症狀。

第三次訪視(第 29± 3 天)-第二次接種疫苗

(1) 進行第二次接種評估(有可能會延遲接種時間)
(2) 進行身體檢查
(3) 確認生命徵象
(4) 進行尿液懷孕檢測（具生育能力的女性）
(5) 若您有產生第三級以上的高血壓，您將被收集尿液檢體以檢測是否有蛋白尿存在或惡化。
(6) 進行第二次疫苗接種。
(7) 接種疫苗後,受試者應留在試驗地點至少 30 分鐘,監測生命徵象和急性過敏症狀。
(8) 將詳細地指導您如何填寫電子日誌卡(包括注射後 7 天內預期性不良事件，14 天內的皮膚過敏反應日誌卡)
(9) 收集併用藥物/治療
(10) 記錄上一次訪視至此次訪視之間的不良事件、嚴重不良事件型或新型冠狀病毒感染症狀

第二次電話安全性追蹤(第 36, 43 天)

將與您電話聯繫，以追蹤未預期不良事件和新型冠狀病毒感染症狀。

第四次訪視(第 57±3 天) –追蹤訪視

(1) 進行身體檢查
(2) 確認生命徵象
(3) 若您有產生第三級以上的高血壓，您將被收集尿液檢體以檢測是否有蛋白尿存在或惡化。
(4) 收集血液檢體(共 15.5 毫升) ，進行下列檢測:
- 常規血液檢測
- 血液生化學檢測
- 免疫學檢測(抗核抗體)
- 備血(作為測量新型冠狀病毒血清抗體和相關研究之用)

(5) 收集併用藥物/治療
(6) 記錄上一次訪視至此次訪視之間的不良事件、嚴重不良事件或新型冠狀病毒感染症狀
(7) 在此次訪視後，您將每周接獲訊息提醒，以定期監測新型冠狀病毒感染症狀至第 197 天。

第三次電話安全性追蹤(第 64, 71, 78, 85 天)

將與您電話聯繫，以追蹤安全性及新型冠狀病毒感染症狀。

第五次訪視(第 197±15 天) –個別解盲

(1) 進行身體檢查
(2) 確認生命徵象
(3) 若您有產生第三級以上的高血壓，您將被收集尿液檢體以檢測是否有蛋白尿存在或惡化。
(4) 將替您個別解盲，告知您接種到疫苗或安慰劑。
(5) 收集併用藥物/治療
(6) 記錄上一次訪視至此次訪視之間的不良事件、嚴重不良事件或新型冠狀病毒感染症狀

第六次訪視（第 197~242 天，第三次接種疫苗）

(1) 記錄您簽署受試者同意書的日期
(2) 確認您是否符合接種第三劑疫苗的資格，包括第三劑接種疫苗的禁忌症，或有延遲第三劑接種時間的條件
(3) 進行身體檢查

(4) 確認生命徵象

(5) 若您有產生第三級以上的高血壓，您將被收集尿液檢體以檢測是否有蛋白尿存在或惡化

(6) 收集血液檢體(**若不進行 T 細胞檢測，**共 20.5 毫升)，進行下列檢測:

- 常規血液檢測
- 血液生化學檢測
- 免疫學檢測(抗核抗體)
- 探索性試驗免疫反應檢測
- **若您願意，將進行 T 細胞檢測(需額外抽血 56 毫升)**

您是否同意? □是 □否

簽名：＿＿＿＿＿＿日期：＿＿＿＿＿＿＿

(7) 進行尿液懷孕檢測（具有生育能力女性）

(8) 進行第三次疫苗接種。

(9) 接種疫苗後，受試者應留在試驗地點至少 30 分鐘，監測生命徵象和急性過敏症狀。將詳細地指導您如何填寫電子日誌卡(包括注射後 7 天內預期性不良事件，14 天內的皮膚過敏反應日誌卡)

(10) 收集併用藥物/治療

(11) 進行新型冠狀病毒監測

(12) 記錄上一次訪視至此次訪視之間的不良事件、嚴重不良事件型或新型冠狀病毒感染症狀

<u>第六次訪視後第 7 天電話安全性追蹤</u>

將與您電話聯繫，以追蹤未預期不良事件和新型冠狀病毒感染症狀。此外，還將監測皮膚過敏反應，或其他非預期的過敏反應。若您有發生任何第三級以上的過敏事件，試驗人員可能將安排您額外的回診。

<u>第七次訪視 (第六次訪視後第 14± 3 天)</u>

(1) 進行身體檢查

(2) 確認生命徵象

(3) 若您有產生第三級以上的高血壓，您將被收集尿液檢體以檢測是否有蛋白尿存在或惡化。

(4) 收集血液檢體(**若不進行 T 細胞檢測，**共 20.5 毫升)，進行下列檢測:

- 常規血液檢測

- 血液生化學檢測
- 免疫學檢測(抗核抗體)
- 探索性試驗免疫反應檢測
- **若您願意，將進行 T 細胞檢測(需額外抽血 56 毫升)**

(5) 收集併用藥物/治療

(6) 進行新型冠狀病毒監測

(7) 記錄上一次訪視至此次訪視之間的不良事件、嚴重不良事件型或新型冠狀病毒感染症狀

追蹤期電話安全性追蹤(第 253, 309 天)

第七次訪視後將每兩個月與您電話聯繫，以追蹤安全性及新型冠狀病毒感染症狀。

第八次訪視**(第 365±45 天) –第 12 個月追蹤**

(1) 確認生命徵象

(2) 收集血液檢體(共 10 毫升)，進行下列檢測:

- 探索性試驗免疫反應檢測

(3) 記錄上一次訪視至此次訪視之間的不良事件，包括特殊不良事件、醫療不良事件、嚴重不良事件或新型冠狀病毒感染症狀

受試者之檢體(含其衍生物)之保存、使用與再利用：

1. 檢體及剩餘檢體之保存與使用

(1) 檢體(含其衍生物)之保存與使用

為研究所需，我們所蒐集您的檢體，將依本研究計畫使用，檢體將保存於聯亞生技開發股份有限公司(試驗委託者)，直至 20 年保存期限屆滿，我們將依法銷毀。為了保護您的個人隱私，我們將以一個試驗編號來代替您的名字及相關個人資料，以確認您的檢體及與相關資料受到完整保密。如果您對檢體的使用有疑慮，或您有任何想要銷毀檢體的需求，請立即與我們聯絡(聯絡人：黃高彬醫師電話：0975-681-950)，我們即會將您的檢體銷毀。您也可以聯繫中國醫藥大學暨附設醫院研究倫理委員會(電話：04-22052121 轉 1925、1926)，以協助您解決檢體在研究使用上的任何爭議。

(2) 剩餘檢體(含其衍生物)之再利用

您的生物檢體將會以專屬號碼進行編碼並在聯亞生技開發股份有限公司(試驗委託

者)的控管下儲存最長20年，以研究UB-612 疫苗反應者的生物標記，及改善治療方式。

所有新的研究計畫都要再經由中國醫藥大學暨附設醫院研究倫理委員會審議通過，倫理審查委員會若認定新的研究超出您同意的範圍，將要求我們重新得到您的同意。

是否同意剩餘檢體保留提供未來新型冠狀病毒感染研究之用，並授權中國醫藥大學暨附設醫院研究倫理委員會審議是否需要再取得您的同意(擇一)

☐ 不同意保存我的剩餘檢體，試驗結束後請銷毀

☐ 同意以非去連結之方式保存我的剩餘檢體，逾越原同意使用範圍時，需再次得到我的同意才可使用我的檢體進行新的研究

2. 檢體及剩餘檢體之部分類型(檢體類型可依計畫書內容自行增減)

(1) 一般生化、血液檢驗/病毒檢測檢體

在試驗期間，會將您的檢體送往聯亞生技開發股份有限公司(試驗委託者)委託的中央實驗室中國醫藥大學暨附設醫院，此機構地址為台中市北區育德路2號，和大安聯合醫事檢驗所，此機構地址為台北市大安區復興南路二段151巷33號，中央實驗室會在分析後立即將分析結果提供給試驗中心，若有剩餘的檢體，將儲存直到至少完成臨床試驗報告為止，最長將保存20年。

(2) 抗體/細胞免疫試驗

在試驗期間，會將您的檢體送往聯亞生技開發股份有限公司(試驗委託者)分析實驗室。完成試驗後，若有剩餘檢體，將儲存直到至少完成臨床試驗報告為止，最長將保存20年。

(3) 中和試驗(neutralization test, NT)

在試驗期間，會將您的檢體送往聯亞生技開發股份有限公司(試驗委託者)委託的中央實驗室中央研究院進行處置、處理與進一步分析。此機構地址為台北市南港區研究院路二段 128 號。完成試驗後，若有剩餘檢體，將儲存直到至少完成臨床試驗報告為止，最長將保存 20 年。

(4) 遺傳學檢體

在試驗期間，若發生嚴重不良反應或特定不良反應，您的檢體將用於 HLA 分型檢驗，會將您的檢體送往聯亞生技開發股份有限公司(試驗委託者)委託的中央實驗室有勁

基因股份有限公司分析，此機構地址為新北市樹林區復興路 376-5 號，中央實驗室不會將分析結果提供給試驗中心，若有剩餘的檢體，將會儲存直到檢驗結果複驗完畢即銷毀，不會長期儲存。

(5) 探索性試驗檢體

在試驗期間，會將您的檢體送往聯亞生技開發股份有限公司(試驗委託者)委託的相關實驗室(表一)進行處理或進一步分析。完成試驗後，若有剩餘檢體，將儲存直到至少完成臨床試驗報告為止，最長將保存 20 年。

表一、實驗室名稱與機構地址

實驗室名稱	機構地址
聯亞生技開發(股)公司	新竹縣竹北市生醫路二段 6-1 號 5 樓
Viroclinics	Rotterdam Science Tower, Marconistraat 16, 3029 AK Rotterdam, The Netherlands(荷蘭)
DASA	Jonas Cruz de Araujo, Diagnostics da America S/A, Surubiju Avenue, 1890, Barueri, SP, Brazil(巴西), 06455-040
PHE Porton Down	Salisbury Wiltshire SP4 0JG, England(英國)
UTMB	University of Texas Medical Branch 301 University Boulevard Keiller Building, Room 2.150 Galveston, Texas, USA(美國
Virology	University of São Paulo, Brazil Rua Dr EnnEn de Carvalho Aguiar 470, CEP 05403-000 (巴西)
VRDL	California Department of Public Health, 850 Marina Bay Parkway, Richmond, CA 94804, USA(美國)
NEXELIS	525 Boul. Cartier Ouest Laval, Qulbec, Canada, H7V 3S8(加拿大)
Vaccinology and Immunology Infection, Immunity & Inflammation Dept UCL GOS Institute of Childe Health	UCL Great Ormond Street Institute of Child Health 30 Guilford Street London WC1N 1EH, England(英國)
VisMederi	VisMederi Srl, Strada del Petriccio e Belriguardo, 35, 53100 Siena, Italy(義大利)

（五）可能產生之副作用、發生率及處理方法：

1. 與試驗藥物相關的風險（本試驗疫苗的副作用）：

冠狀病毒疫苗的開發

過去針對與SARS-CoV-2病毒相同屬於人類冠狀病毒的SARS-CoV(嚴重急性呼吸綜合症

冠狀病毒(SARS冠狀病毒))的疫苗研究發現，接種過SARS-CoV疫苗的小鼠在暴露到SARS-CoV後會發生過度免疫反應而產生病變，因此不得不停止這種疫苗的開發。所以，成功的人類冠狀病毒疫苗不只要產生可以抑制病毒的免疫反應，更要避免過度免疫產生的副作用。

疫苗相關的風險: 第一期臨床試驗

接種疫苗可能會出現注射部位的不良反應(例如疼痛、硬化腫脹、皮疹發紅、過敏反應、蜂窩性組織炎)，或全身性不良反應(例如發燒、腹瀉、疲倦、噁心/嘔吐、厭食、咽喉痛、頭痛、咳嗽、關節痛、非注射部位疼痛、非注射部位搔癢、皮膚和黏膜異常、急性過敏反應、昏厥、急性支氣管痙攣、呼吸困難)。

第一期臨床試驗已經有60位受試者接種兩劑疫苗(含10微克、30微克、100微克融合蛋白)，安全實驗室數值並沒有顯示有任何的臨床顯著不正常數值，也沒有發生任何第三級以上與疫苗相關的預期性不良事件。大部分的預期性不良事件都是輕微的，症狀在大約於2天之內都會緩解。試驗中也沒有任何的嚴重不良事件或特殊不良事件被通報。

疾病增強(disease enhancement) 的風險

SARS-CoV-2候選疫苗也可能會有引發疾病增強(disease enhancement) 的風險，包括抗體依賴性增強(antibody-dependent enhancement)或疫苗相關聯的增強的呼吸道疾病(vaccine-associated enhanced respiratory disease)。在先前研發SARS疫苗時，在數個SARS-CoV動物攻毒試驗(包括鼠類、雪貂、猴類)當中，有發現疾病增強的現象。疾病增強反應的免疫病理現象包括TH2偏向及嗜酸性白血球的肺部浸潤。但是目前已發表的新型冠狀病毒肺炎疫苗研究，仍尚未發現類似的疾病增強現象。

本試驗疫苗於數個藥理試驗呈現不一致的TH1/TH2 (輔助型T細胞1/輔助型T細胞2)免疫反應偏向，試驗結果並未一致偏向TH2，而由小鼠之SARS-CoV-2動物攻毒試驗結果顯示，本試驗疫苗誘發疾病增強之風險不高。依據文獻指出，組成複雜或容易引起非中和抗體之抗原，如不活化病毒或整片段之蛋白(包含S蛋白與N蛋白)，與易引起偏向Th2免疫反應之佐劑成分，如鋁製佐劑，皆較有可能引起疾病增強。本試驗疫苗的主要抗原為S蛋白上之RBD 區域，已有多篇文獻指出，針對S-RBD設計之SARS與MERS疫苗從未於試驗動物模型上引發疾病增強現象。本試驗疫苗雖使用易引起偏向Th2免疫反應之佐劑，但由動物實驗證實，也同時引起偏向Th1之反應，因此發生疾病增強應屬低風險。且已於多種動物模型中證實，能誘發高效價之中和抗體，於細胞培養中亦能有效抑制新冠病毒感染。

建議您在有效疫苗上市前或本試驗疫苗的產品資訊有進一步更新前，盡量避免暴露於可能感染病毒的環境。研究團隊將會在試驗中執行相關安全性監測。若有任何關於本試驗

疫苗與疾病增強風險相關之任何最新資訊，將即時更新並提供給您。

<u>**疫苗佐劑相關的風險**</u>

本試驗疫苗所使用的佐劑含Adju-Phos®，是屬於一種磷酸鋁類的佐劑。磷酸鋁類佐劑已經使用超過半個世紀，具有相當的安全性。由於此類佐劑可誘導免疫反應，因此可能會造成局部發炎反應，例如在注射部位產生輕微而短暫的疼痛、發紅以及腫脹。

2. 與試驗/研究過程相關的風險：

<u>**抽血**</u>

本試驗需要抽血檢驗。抽血可能引起一些不適和瘀血。整個試驗期間13個月，共需抽血 82毫升。**若您願意抽血檢驗T細胞檢測，則會於特定訪視每次額外抽血56毫升。**若您罹患新型冠狀病毒感染，可能將於每次額外訪視抽血30毫升。

在接種疫苗過程中，可能會出現一些尚未在已完成試驗中發現的副作用。一般而言，接種某一新疫苗總是會有一定的風險，但是計畫主持人會採取一切措施預防風險的發生。**計畫主持人鼓勵您報告您遇到的任何不適。**

(六)其他替代療法及說明：

您不是非參加不可，若不參加研究，由於目前尚未有疫苗可用來預防新型冠狀病毒感染，因此預防措施與其他呼吸道感染相同，包括：勤洗手、減少觸摸眼口鼻、注意咳嗽禮節、妥善處理口鼻分泌物等，避免出入公共場所，並不要接觸野生動物。

如果您對於本試驗疫苗有任何的疑問，您可以提出來向您的試驗醫師討論。

(七)試驗預期效益：

依據臨床前試驗結果，預期本試驗疫苗對您可能可以產生抗體，預防新型冠狀病毒感染，但因每個人體質不同也有可能不會產生療效，故參加本試驗可能不會有直接的好處。

但是您參加本試驗，可協助我們獲得更多資訊，以瞭解UB-612疫苗的安全性與免疫力。

(八)試驗進行中受試者之禁忌、限制與應配合之事項：

<u>**禁止使用的藥物**</u>

以下藥物請勿在試驗期間使用：

- 直到試驗第57天禁止使用免疫抑制劑、或細胞毒性治療

- 到試驗第57天禁止使用免疫球蛋白和/或任何血液製劑
- 整個試驗期間禁止使用試驗產品(包括藥物或疫苗)
- 到試驗第57天禁止使用全身性皮質類固醇(相當於一天使用≥ 20 mg強的松(prednisone))
- 接種試驗疫苗後14天禁止接種任何季流感疫苗或新型流感疫苗，或後28天禁止接種其他非試驗疫苗。整個試驗期間禁止使用任何已上市的新型冠狀病毒疫苗產品。

允許使用的藥物

若您的藥物或治療必須常規使用，經試驗醫師判斷不會影響本試驗疫苗的安全性，則可以正常使用。您有任何關於在試驗期間可允許使用何種藥物或治療的問題，請詢問您的試驗醫師。

懷孕或母乳哺乳的風險

目前未知本試驗疫苗對於未出生胎兒的影響，因此：

- 您為具生育能力的女性受試者 (除非手術絕育或停經)，或您為男性受試者應於接種疫苗至最後一次疫苗後 3 個月同意進行有效的避孕方式，同意進行有效的避孕方式(例如子宮內節育器、荷爾蒙療法或避孕套)。
- 若您為具生育能力的女性，將請您進行懷孕檢測，結果必須為陰性，方可參與試驗。
- 若您為懷孕的女性，將被告知不可參與本試驗。
- 若您在試驗期間懷孕，請盡速通知試驗人員，並且停止施打本疫苗。
- 基於安全性考量，若您為女性受試者而在試驗期間懷孕，或您為男性受試者而您的性伴侶在試驗期間懷孕(將請您的懷孕性伴侶需簽署另外一份同意書)，您與您的胎兒將會被追蹤監測至分娩，除非另有醫學指示。

您應向您的配偶或性伴侶告知您有參與此試驗與相關風險:

簽名：__________日期：__________________

(九)機密性：

中國醫藥大學附設醫院將依法把任何可辨識您的身分之紀錄與您的個人隱私資料視為機密來處理，不會公開。研究人員將以一個研究代碼代表您的身分，此代碼不會顯示您的

中國醫藥大學暨附設醫院
受試者同意書
（成年安全確認第三劑組）

姓名、國民身分證統一編號、住址等可識別資料。如果發表試驗/研究結果，您的身分仍將保密。您亦瞭解若簽署同意書即同意您的原始醫療紀錄可直接受監測者、稽核者、研究倫理委員會及主管機關檢閱，以確保臨床試驗/研究過程與數據符合相關法律及法規要求，上述人員並承諾絕不違反您的身分之機密性。除了上述機構依法有權檢視外，我們會小心維護您的隱私。由於試驗藥物可能同時申請美國臨床試驗，依美國藥品管理規定，試驗結果將公佈於公開的臨床試驗資訊網站：Clinicaltrials.gov (美國)，但您的個人資料仍將保密，該網站只會有試驗之結果摘要，您可以在任何時候搜尋該網站。

在試驗/研究期間，依據計畫類型與您所授權的內容，我們將會蒐集與您有關的病歷資料、醫療紀錄、量表、問卷等資料與資訊，並以一個編號來代替您的名字及相關個人資料。前述資料若為紙本型式，將會與本同意書分開存放於研究機構之上鎖櫃中；若為電子方式儲存或建檔以供統計與分析之用，將會存放於設有密碼與適當防毒軟體之專屬電腦內。這些研究資料與資訊將會保存至藥品於我國上市後至少兩年，若試驗疫苗終止研發則保存至試驗正式停止後至少二年，至多將保存至疫苗上市後或試驗正式停止後二年。上述資料與資訊若傳輸至國外分析與統計，您仍會獲得與本國法規相符之保障，計畫主持人與相關團隊將盡力確保您的個人資料獲得妥善保護。

（十）損害補償與保險：

1. 如依本研究所訂臨床試驗計畫，因發生不良反應造成損害，由聯亞生技開發股份有限公司負補償責任。但本受試者同意書上所記載之可預期不良反應，不予補償。
2. 如依本研究所訂臨床試驗計畫，因而發生不良反應或損害，贊助廠商將依法負責損害賠償責任。本醫院願意提供專業醫療照顧及醫療諮詢。您不必負擔治療不良反應或損害之必要醫療費用。
3. 除前二項補償及醫療照顧外，本研究不提供其他形式之補償。若您不願意接受這樣的風險，請勿參加試驗。
4. 您不會因為簽署本同意書，而喪失在法律上的任何權利。
5. 本研究有投保責任保險。

（十一）受試者權利：

1. 試驗過程中，與您的健康或是疾病有關，可能影響您繼續接受臨床試驗意願的任何重大發現，都將即時提供給您。
2. 如果您在試驗過程中對試驗工作性質產生疑問，對身為患者之權利有意見或懷疑因參與研究而受害時，可與本院之研究倫理委員會聯絡請求諮詢，其電話號碼為：04-22052121轉1925、1926。
3. 為進行試驗工作，您必須接受黃高彬醫師的照顧。如果您現在或於試驗期間有任

何問題或狀況，請不必客氣，可與在中國醫藥大學附設醫院兒童感染科的黃高彬醫師聯絡（24小時聯繫電話：0975-681-950）。

4. 參加試驗研究計畫之補助：本計畫將在每次訪視提供交通費及營養費給您，新增的兩個診次(第六次返診、第七次返診)將各提供費用1000元；整個試驗預計給予您14000元。**若您願意參與T細胞檢測研究，將在該次訪視另外提供營養費500元給您。**

5. 本同意書一式2份，醫師已將同意書副本交給您，並已完整說明本研究之性質與目的。醫師已回答您有關藥品與研究的問題。

（十二）試驗之退出與中止：

您可自由決定是否參加本試驗；試驗過程中也可隨時撤銷同意，退出試驗，不需任何理由，且不會引起任何不愉快或影響其日後醫師對您的醫療照顧。

計畫主持人或贊助廠商亦可能於必要時中止該試驗之進行。

（十三）簽名：

1. 計畫主持人、或協同主持人已詳細解釋有關本研究計畫中上述研究方法的性質與目的，及可能產生的危險與利益。

   計畫主持人/協同主持人簽名：____________日期：________年____月____日

2. 受試者已詳細瞭解上述研究方法及其所可能產生的危險與利益，有關本試驗計畫的疑問，業經試驗主持人詳細予以解釋。本人同意接受為臨床試驗計畫的自願受試者。

   受試者簽名：____________________________日期：________年____月____日

   法定代理人簽名：________________________日期：________年____月____日

   * 受試者為無行為能力(未滿七歲之未成年人者或禁治產人)，由法定代理人為之；禁治產人，由監護人擔任其法定代理人。

   * 受試者為限制行為人者(滿七歲以上之未成年人)，應得法定代理人之同意。

   有同意權人簽名：________________________日期：________年____月____日

   * 受試者雖非無行為能力或限制行為能力者，但因意識混亂或有精神與智能障礙，而無法進行有效溝通和判斷時，由有同意權之人為之。前項有同意權人為配偶及直系親屬。

3. 見證人

   見證人簽名：_____________________________日期：________年____月____日

   身分證字號：　　　　　　　　　　　　聯絡電話：

   通訊地址：

中國醫藥大學暨附設醫院

受試者同意書

（成年安全確認第三劑組）

* 受試者、法定代理人或有同意權之人皆無法閱讀時，應由見證人在場參與所有有關受試者同意之討論。並確定受試者、法定代理人或有同意權之人之同意完全出於其自由意願後，應於受試者同意書簽名並載明日期。試驗相關人員不得為見證人。

中國醫藥大學暨附設醫院
受試者同意書
（成年安全確認第三劑組）

流程表:

安全確認組

訪視	**1**[l]	**2**[l]		*3*		*4*		*5/提早退出試驗*	*6*[t]		*7*		長期性追蹤
檢測項目	篩選	第一次接種		*第二次接種*		*追蹤*[f]		*個別解盲*	*第三次接種*[g]		*追蹤*[g]		第 **12** 個月追蹤[h]
天數	**-28~-1**	*1*	*8, 15, 22*	*29 ±3 天*	*36, 43*	*57 ±3 天*	*64, 71, 78 ,85*	*197 ±15 天*	*197~242*	*第六次訪視後 7 天*	*第六次訪視後 14 天 ±3 天*	*253, 309*	**365 ±45** 天
獲得受試者同意書	X								X[g]				
納入/排除條件	X	X											
隨機分派		X											
接種評估				X					X[g]				
基本資料	X												
醫療病史	X	X											
身體檢查[a]	X	X		X		X		X	X[g]		X[g]		X
生命徵象	X	X		X		X		X	X[g]		X[g]		
心電圖	X												
**實驗室檢測 (安全性)**													
血液常規檢測[i]	X					X			X[g]		X[g]		
血液生化學檢測[i]	X					X			X[g]		X[g]		
免疫檢測[i]	X					X			X[g]		X[g]		

訪視	**1** [l]	**2** [l]		*3*		*4*		*5/提早退出試驗*	*6*[t]		*7*		長期性追蹤
檢測項目	篩選	第一次接種		*第二次接種*		*追蹤* [f]		*個別解盲*	*第三次接種* [g]		*追蹤* [g]		第 **12** 個月追蹤 [h]
天數	**-28~-1**	*1*	*8, 15, 22*	*29 ±3 天*	*36, 43*	*57 ±3 天*	*64, 71, 78 ,85*	*197 ±15 天*	*197~242*	*第六次訪視後 7 天*	*第六次訪視後 14 天 ±3 天*	*253, 309*	**365 ±45** 天
懷孕檢測 [b]		X		X					X[g]				
尿液常規檢測 [b]	X	X [n]		X [n]		X [n]		X [n]	X[g, n]		X[g, n]		
**實驗室檢測(探索性試驗)**									X		X		X
**T** 細胞功能性檢測(可選擇 [r])									**X** [g]		**X**[g]		
備血	X					X							
免疫原性 [c]									X [g]		X [g]		X [g]
疫苗接種		X		X					X [g]				
指導使用電子日誌卡		X		X					X [g]				
電話安全性追蹤 [c]			X		X		X			X[p, g]		X	
不良事件/特殊不良事件 [m]/醫療需求不良事件/嚴重不良事件		X [j]	X [j]	X [j]	X [j]	X [j]	X [j]	X [k]	X [k, g]	X [k, g]	X [k ,g]	X [k]	X [k]

中國醫藥大學暨附設醫院

受試者同意書

（成年安全確認第三劑組）

訪視	1[l]	2[l]		3		4		5/提早退出試驗	6[t]		7		長期性追蹤
檢測項目	篩選	第一次接種		第二次接種		追蹤[f]		個別解盲	第三次接種[g]		追蹤[g]		第 12 個月追蹤[h]
天數	-28~-1	1	8, 15, 22	29 ±3 天	36, 43	57 ±3 天	64, 71, 78 ,85	197 ±15 天	197~242	第六次訪視後 7 天	第六次訪視後 14 天 ±3 天	253, 309	365 ±45 天
新型冠種病毒感染監測		X	X	X	X	X[d]	X[d]	X[d]	X[d, g]	X[d, g]	X[d, g]	X[d]	X[d]
併用藥物		X		X		X		X[e]	X[e, g]		X[e, g]		

a: 身高與體重僅在第一次訪視測量。
b: 於第 1, 29 天使用尿液懷孕檢測。若尿液檢測為陽性，應以血清懷孕檢測再次確認。以血清懷孕檢測替代尿液檢測將不視為試驗偏差。蛋白尿將透過尿液常規檢測確認，做為基礎值。
c: 所有受試者將進行電話安全追蹤以監測非預期性不良事件，包括特殊不良事件，和監測新型冠狀病毒感染的症狀。
d: 受試者將在每周(為每 7 天)由手機接獲一條提示以規律監測新型冠狀病毒感染症狀或病徵。記錄疑似新冠病毒感染所使用的藥物至新冠病毒感染頁面。
e: 只記錄醫療需求不良事件和嚴重不良事件之併用藥物。
f: 第二次接種疫苗後第 28 天
g: 針對同意並進行施打第三劑試驗疫苗的疫苗組受試者
h: 第二次接種疫苗後第十二個月(第 365 天)
i: 安全性實驗室數值包括全血液計數(血紅素、血比容、紅血球計數)、白血球計數、血小板計數、肌酸酐、丙胺酸轉胺酶、天門冬胺酸轉胺酶、總膽紅素、直接膽紅素、高靈敏度 C 反應性蛋白、抗核抗體
j: 主動收集時期
k: 被動監測時期
l: 第一次訪視與第二次訪視可為同一次訪視。
m: 包括接種最後一劑疫苗後 12 個月，可能的免疫媒介醫療狀況(PIMMC)或任何定義為可能的特殊不良事件。新冠病毒感染的併發症也將視為疾病增強事件，將被記錄與通報為特殊不良事件。
n: 若受試者有≥第三級以上的高血壓，受試者將或熱是否有蛋白尿存在或惡化。
o: 備血將被冷凍存放做為 UBI 新型冠狀病毒酵素結合免疫吸附分析法，新型冠狀病毒確認酵素結合免疫吸附分析法，以及未來免疫學研究之用。
p: 若符合資格並接種第三劑疫苗的受試者將進行兩週的日誌卡追蹤紀錄，並將於接種後第 7 天進行電話安全性追蹤。
q: 第五次訪視和第六次訪視可為同一天。
r: 在選定之試驗地點中，將邀請大約 30 位年齡 >18-<65 歲的受試者和大約 30 位年齡 ≥65 歲的受試者。在適用的情況下，優先邀請曾進行過第 57 天 T 細胞功能評估的受試者。
